# Expedient synthesis of the heneicosasaccharyl mannose capped arabinomannan of the *Mycobacterium tuberculosis* cellular envelope by glycosyl carbonate donors†
†Electronic supplementary information (ESI) available: Experimental procedures, compound characterization data and spectral charts. See DOI: 10.1039/c6sc04866h



**DOI:** 10.1039/c6sc04866h

**Published:** 2016-11-15

**Authors:** Maidul Islam, Ganesh P. Shinde, Srinivas Hotha

**Affiliations:** a Department of Chemistry , Indian Institute of Science Education and Research , Pune – 411 008 , India . Email: s.hotha@iiserpune.ac.in

## Abstract

The global incidence of tuberculosis is increasing at an alarming rate, and *Mycobacterium tuberculosis* (Mtb) is the causative agent for tuberculosis, a disease with high mortality. Lipoarabinomannan (LAM) is one of the major components of the Mtb cellular envelope and is an attractive scaffold for developing anti-tubercular drugs, vaccines and diagnostics. Herein, a highly convergent strategy is developed to synthesize heneicosasaccharyl arabinomannan for the first time. The arabinomannan synthesized in this endeavour has several 1,2-*trans* or α-Ara*f* linkages and three 1,2-*cis* or β-Ara*f* linkages end capped with 1,2-*trans* or α-Man*p* linkages. All the key glycosidations were performed with alkynyl carbonate glycosyl donors under [Au]/[Ag] catalysis conditions, which gave excellent yields and stereoselectivity even for the reactions between complex and branched oligosaccharides. The resultant allyl oligosaccharide was globally deprotected to obtain the heneicosasaccharyl arabinomannan as a propyl glycoside. In summary, heneicosasaccharyl mannose capped arabinomannan synthesis was achieved in 56 steps with 0.016% overall yield.

## Abstract 2

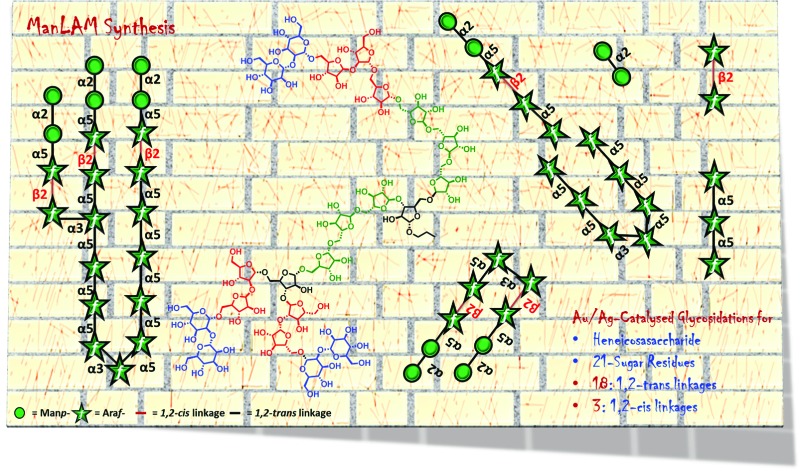
Herein, a highly convergent strategy is developed to synthesize heneicosasaccharyl arabinomannan for the first time.

## Introduction

The world-wide resurgence of mycobacterial infections coupled with the emergence of multi- and extreme drug resistance have placed tuberculosis (TB) as a major public health concern.^1–3^ The only available protection against tuberculosis is the BCG vaccine; however, multi-centered clinical trials demonstrated variable efficacy.^4^ Tuberculosis infections are caused by *Mycobacterium tuberculosis* (Mtb), which has a thick waxy cell wall making it impervious to drugs.^5,6^ As a consequence, patients suffering from TB are prescribed a long regimen of multiple drugs. Therefore, TB is an ever growing challenge, and novel strategies to diagnose, control or eradicate it are in great demand.

The chemical structure of the waxy cellular envelope has been identified as a unique glycocalyx comprising mycolic acids, Ara*f*, Gal*f*, Man*p*, Rha*p* and inositols.^7–10^ ‡
‡Ara*f* means arabinofuranose; Man*p* means mannospyranose, Im. means imidazole, DMAP means 4-*N*,*N*′-dimethylaminopyridine, THF means tetrahydrofuran, and 4 Å MS means 4 Å molecular sieves. Further investigations revealed that the glycocalyx contains trehalose lipids, lipoarabinomannan (LAM), arabinogalactan (AG) and peptidoglycan.^7–10^ Of these, the structure of LAM was noticed to have a key C-3 branched arabinan domain with many α-1 → 5-linked d-Ara*f*s, and a few β-1 → 2-linked d-Ara*f*s capped with α-1 → 2 linked d-Man*p*s at the non-reducing end.^7–10,13^ It has been well established that mannose capped LAM (ManLAM) is prevalent in more pathogenic mycobacterial species such as *M. tuberculosis*, *M. leprae*, *M. bovis*.^11–13^ ManLAM has been shown to inhibit the production of tumor necrosis factor-α (TNF-α) and interleukin-12 (IL-12) by human dendritic cells and macrophages *in vitro* to modulate *M. tuberculosis* induced macrophage apoptosis.^14,15^ Quite recently, a rapid point of care diagnostic kit was developed exploiting the antigenic properties of ManLAM.^16–18^ In another study LAM was investigated as a candidate vaccine for mycobacterial diseases. Thus, the non-reducing end portion of LAM is beneficial to various immunological studies, diagnostics and the development of carbohydrate-based tuberculosis vaccines.

**Fig. 1:**
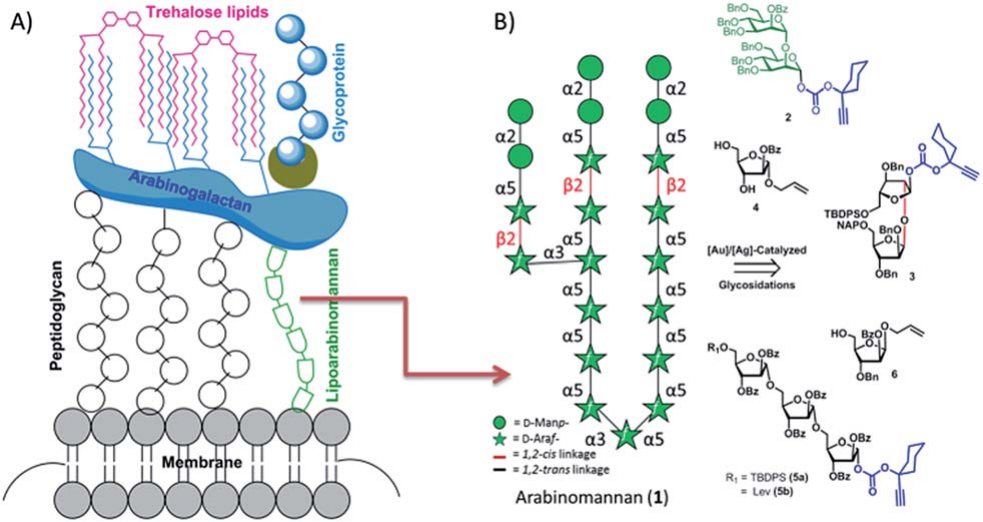
Cartoon representation of the *Mycobacterium tuberculosis* cell wall (A) and the retrosynthesis of heneicosasaccharyl mannose capped arabinan (B).

Owing to the significance of ManLAM, the synthesis of ManLAM and arabinan fragments has been attempted over the last two decades.^19–27,44^ A recent investigation by Guo's group verified the synthetic and immunological potential of protein conjugated ManLAM fragments.^28^ However, far too little attention has been paid to synthesizing the large oligomers in ManLAM. The main aim of the current research has therefore been to synthesize naturally occurring large oligosaccharide portions of ManLAM such as arabinomannan **1** (Fig. 1) to facilitate vaccine and diagnostic development.

## Results and discussion

The arabinomannan moiety (**1**) offers many complexities and challenges in its synthesis in terms of the type and number of linkages and the asymmetric branching.Careful retrosynthetic disconnection revealed that the target molecule, as a propyl glycoside, can be synthesized conveniently using two disaccharides (**2**, **3**), two monosaccharides (**4**, **6**) and a trisaccharide (**5**) (Fig. 1). Apart from their continued interest,^29–34^ alkynyl carbonate donors are chosen as they undergo catalytic activation in the presence of gold and silver salts, and are therefore fast and high yielding.^35^ The neighboring group or reciprocal donor acceptor selectivity assisted synthesis of 1,2-*trans* or α-linkages and stereoelectronics guided synthesis of 1,2-*cis* or β-Ara*f* was envisioned.^36^ An allyl moiety was strategically placed at the anomeric position since it is stable and orthogonal to -OTBDPS, -OBz, and -ONAP protecting groups and can be converted to a hemiacetal *en route* to glycosyl donor preparation. In addition, an allyl moiety can also be exploited for the conjugation of proteins and biomolecules.^37–40^

**Scheme 1:**
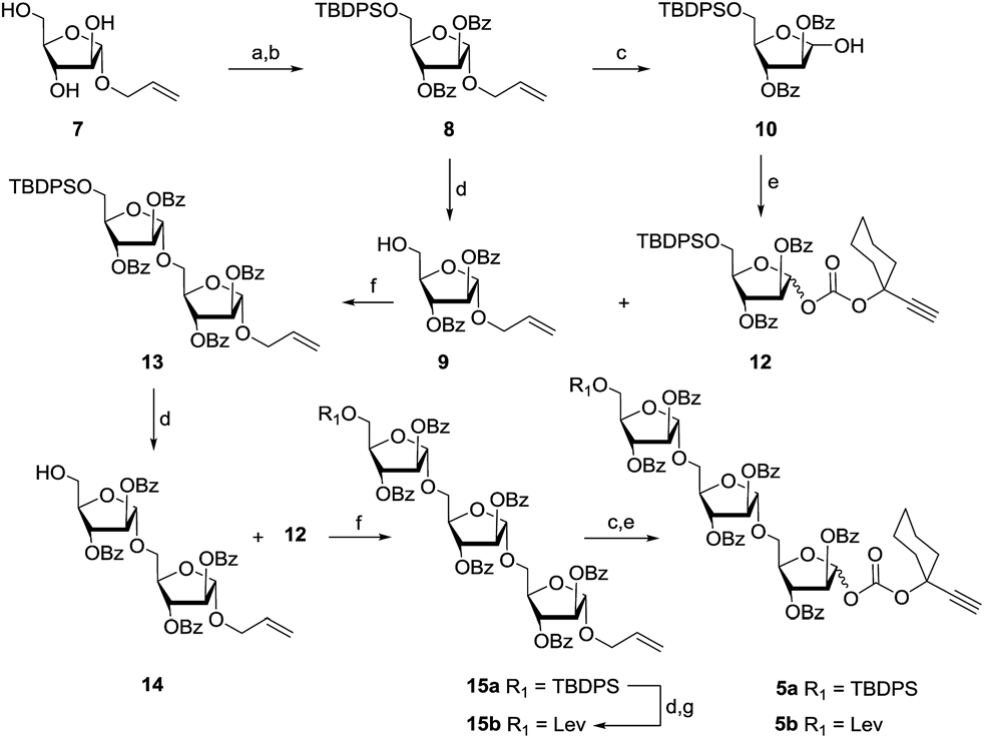
Reagents: (a) TBDPS–Cl, Im., DMF, 0 °C, 1 h, 82%; (b) BzCl, pyridine, DMAP, 0–25 °C, 5 h, 93%; (c) PdCl_2_, CH_2_Cl_2_–MeOH (1 : 4), 25 °C, 4 h, 85%; (d) HF·py, pyridine, 0–25 °C, 5 h, 93% for **9**, 91% for **14**, and 90% for **15b**; (e) 1-ethynyl cyclohexyl(4-nitrophenyl)carbonate (**11**), CH_2_Cl_2_, DMAP, 0–25 °C, 3 h, 85% for **12**, 83% for **5a** and 85% for **5b** over two steps; (f) 8 mol% chloro[tris(2,4-di-*tert*-butylphenyl)phosphite] gold, 8 mol% AgOTf, CH_2_Cl_2_, 4 Å MS powder, 25 °C, 15 min, 95% for **13** and 92% for **15a**; (g) levulinic acid, DIC, DMAP, CH_2_Cl_2_, 0–25 °C, 2 h, 95%.

Our synthetic endeavour started with the preparation of a triarabinofuranosyl carbonate donor (Scheme 1). Easily accessible allyl arabinofuranoside **7** ^41^ was converted into fully protected compound **8** by first protecting the C-5-OH as a silyl ether using TBDPS–Cl, followed by the protection of the remaining hydroxyls as benzoates with BzCl/py/DMAP. Compound **8** serves as a common building block for the synthesis of both glycosyl donors and acceptors. Accordingly, compound **8** has been split into two portions and one portion was converted into the glycosyl acceptor **9** by treatment with HF·py in THF whereas the second portion was converted into hemiacetal **10** using PdCl_2_; subsequently, this was transformed to glycosyl donor **12** by reacting it with easily available ethynyl cyclohexyl(4-nitrophenyl)carbonate **11** (Scheme 1).

The first [Au]/[Ag]-glycosidation between acceptor **9** and donor **12** was successfully performed to afford disaccharide **13** in excellent yield.^35^ In continuation, a lone silyl ether was deprotected under HF·py conditions to afford acceptor **14** which was glycosylated again with the glycosyl donor **12** under gold/silver catalytic conditions to obtain trisaccharide **15a** as an allyl glycoside. Cleavage of the silyl ether and protection as a levulinoate resulted in the other required trisaccharide **15b**. Trisaccharides **15a** and **15b** were respectively converted easily into the triarabinofuranosyl carbonate donors **5a** and **5b** (Scheme 1).^46^

**Scheme 2:**
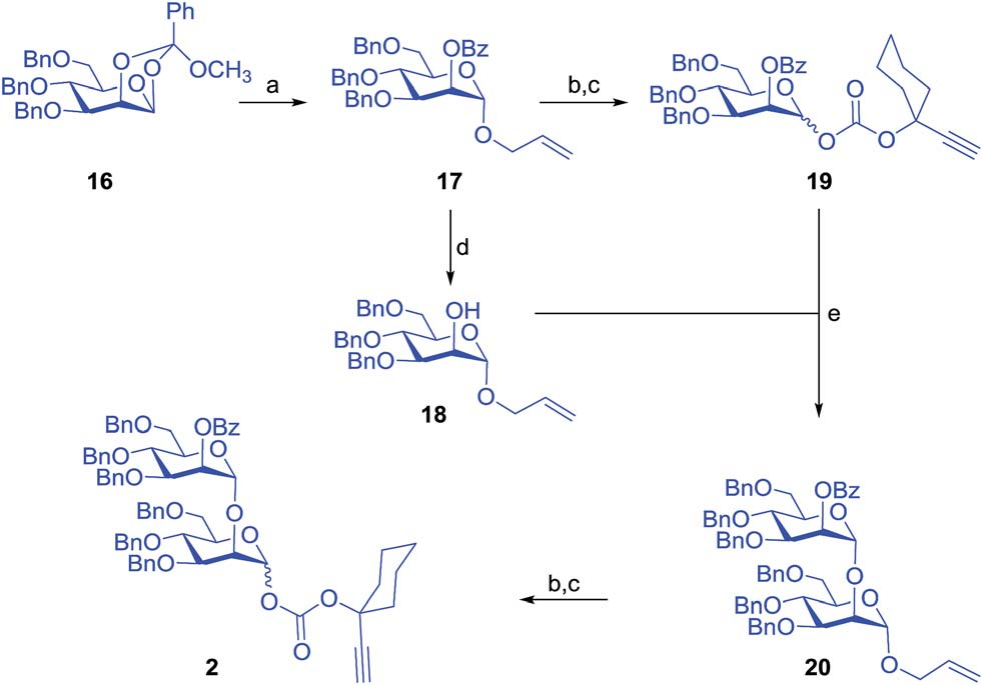
Reagents: (a) PTSA (0.2 eq.), allyl alcohol, CH_2_Cl_2_, 4 Å MS powder, 25 °C, 1 h, 86%; (b) PdCl_2_, CH_2_Cl_2_–MeOH (1 : 4), 25 °C, 4 h, 90% towards **19**; (c) **11**, CH_2_Cl_2_, DMAP, 0–25 °C, 3 h, 78% for **19** and 83% for **2** over two steps; (d) NaOMe, MeOH, 25 °C, 1 h, 94%; (e) 8 mol% chloro[tris(2,4-di-*tert*-butylphenyl)phosphite] gold, 8 mol% AgOTf, CH_2_Cl_2_, 4 Å MS powder, 25 °C, 15 min, 93%.

In parallel, allyl mannopyranoside **17** was synthesized from known mannopyranosyl 1,2-orthoester **16** ^27^ under acidic conditions. Subsequently, allyl glycoside **17** was split into two portions and one part was subjected to saponification under Zemplén conditions^42^ to afford the acceptor **18**, and the other part was converted into the glycosyl donor **19** in two easy steps. The gold/silver assisted glycosidation between the donor **19** and the acceptor **18** went uneventfully affording 93% of the disaccharide **20** which was subsequently converted into the other required building block **2** in two steps (Scheme 2).^46^

The synthesis of the next important 1,2-*cis* disaccharide **3** was initiated with the saponification of compound **8** under Zemplén conditions (NaOMe/MeOH); the subsequent conversion of particular groups to benzyl ethers afforded compound **21**. Moving on, the cleavage of the silyl ether using HF·py and protection of the resulting hydroxyl group as a NAP ether went effortlessly by employing NAP-Br to afford NAP-protected allyl glycoside **22**. Hydrolysis of the allyl glycoside using PdCl_2_ and its conversion to the corresponding donor **23** was achieved in very high yield. In parallel, compound **24** was protected as benzyl ether **25** using NaH/BnBr/DMF and the opening of the isopropylidene moiety was performed in the presence of allyl alcohol under acidic conditions to afford an α,β-mixture of glycosides.

**Scheme 3:**
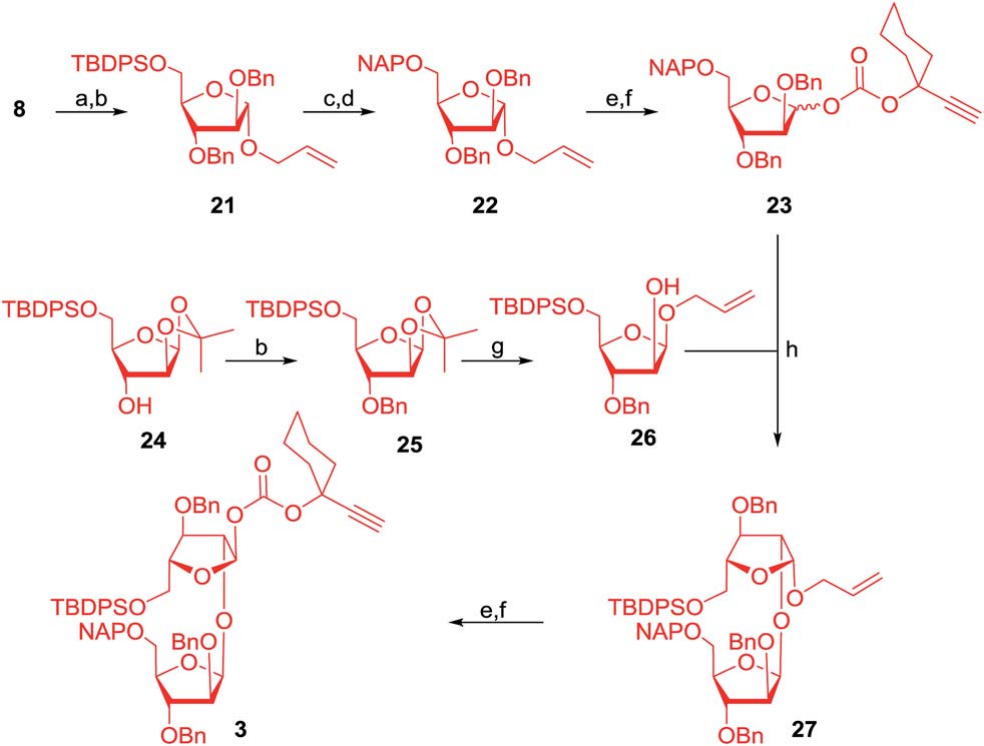
Reagents: (a) NaOMe, MeOH, 25 °C, 1 h, 90%; (b) NaH, BnBr, TBAI, DMF, 0–25 °C, 1 h, 91% for **21** and 93% for **25**; (c) HF·py, pyridine, 0–25 °C, 5 h, 92%; (d) NaH, NAPBr, TBAI, DMF, 0–25 °C, 2 h, 90%; (e) PdCl_2_, CH_2_Cl_2_–MeOH (1 : 4), 25 °C, 4 h; (f) **11**, DMAP, CH_2_Cl_2_, 0–25 °C, 3 h, 82% for **23** and 78% for **3** over two steps; (g) PTSA (0.2 eq.), allyl alcohol, CH_2_Cl_2_, 50 °C, 2 h, 45%; (h) 8 mol% chloro[tris(2,4-di-*tert*-butylphenyl)phosphite] gold, 8 mol% AgOTf, CH_2_Cl_2_, 4 Å MS powder, –78 °C, 5 h, 92%.

Earlier studies from our group demonstrated that the reciprocal donor acceptor selectivity depends on the reactivity of the nucleophile and the stereoelectronics around the C-2 position of the glycosyl acceptor.^36^ Accordingly, the allyl glycosides were separated by flash silica gel column chromatography and this isolated the required 1,2-*cis* disposed allyl glycoside **26**. Glycosyl donor **23** and acceptor **26** were subjected to [Au]/[Ag]-catalysed glycosidation conditions to afford the 1,2-*cis* or β-disaccharide **27** in 92% yield as a single diastereomer further verifying our earlier results.^36,46^ Subsequently, the disaccharide **27** was converted into the required glycosyl donor **3** in two steps *viz.* the Pd-catalysed hydrolysis of the allyl glycoside to a hemiacetal followed by its conversion to the ethynylcyclohexyl carbonate by treating it with compound **11** and DMAP. Orthogonally cleavable TBDPS and NAP ethers were selected for installing the mannopyranosyl disaccharide at a later stage (Scheme 3).

Synthesis of another monosaccharide **4** commenced with the saponification of easily accessible compound **28** ^43^ under Zemplén conditions (NaOMe/MeOH) followed by its conversion to disilyl ether **29** using TBDPSCl/Im./DMAP resulting in 81% yield over two steps. Acid mediated opening of the 1,2-orthoester in the presence of an allyl alcohol afforded the allyl glycoside in 80% yield. Cleavage of the silyl ethers was achieved using HF·py to afford the allyl glycoside **4**, and the resulting C-5 hydroxyl group was protected as its silyl ether to afford the glycosyl acceptor **31**.

**Scheme 4:**
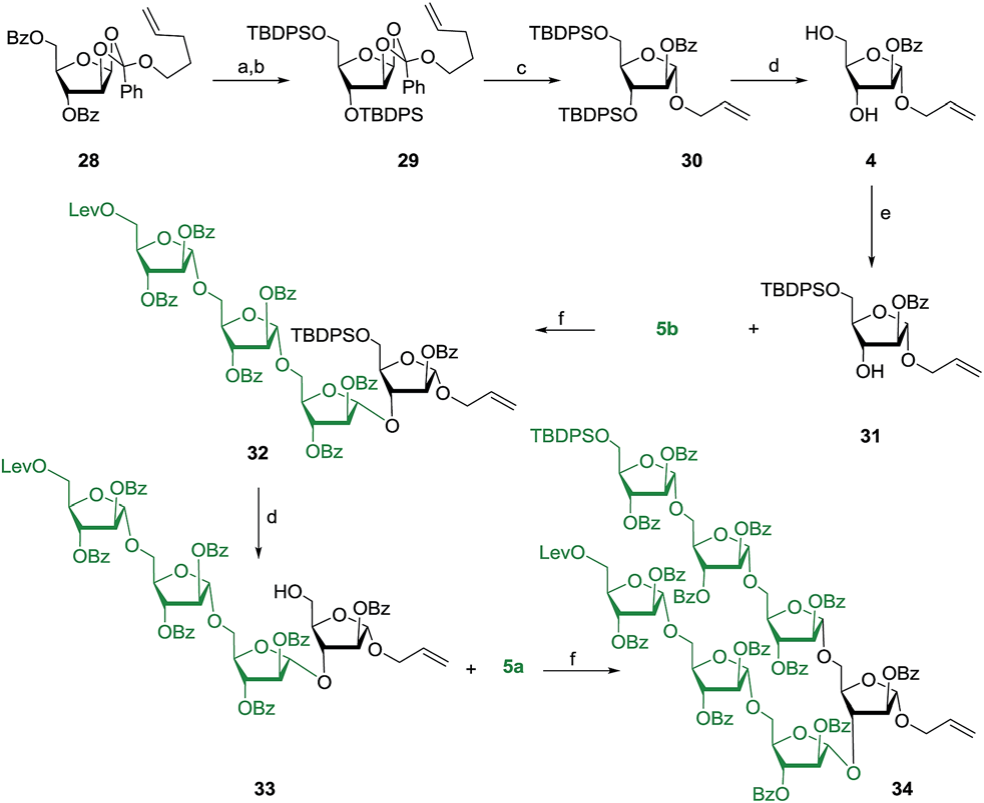
Reagents: (a) NaOMe, MeOH, 25 °C, 1 h, 95%; (b) TBDPS–Cl (2.5 eq.), Im., DMF, 0–25 °C, 2 h, 85%; (c) PTSA (0.2 eq.), excess allyl alcohol, CH_2_Cl_2_, 4 Å MS powder, 25 °C, 1 h, 80%; (d) HF·py, pyridine, 0–25 °C, 6 h, 90% for **4** and 90% for **33**; (e) TBDPS–Cl, Im., DMF, 0 °C, 1 h, 80%; (f) 8 mol% chloro[tris(2,4-di-*tert*-butylphenyl)phosphite] gold, 8 mol% AgOTf, CH_2_Cl_2_, 4 Å MS powder, 25 °C, 20 min, 95% for **32** and 92% for **34**.

Synthesis of enough quantities of all of the identified major partners drove the assembly of ManLAM. Accordingly, the glycosyl acceptor **31** and the glycosyl donor **5b** were first glycosylated under gold–silver catalysed glycosidation conditions to afford the tetrasaccharide **32** in excellent yield.^46^ Deprotection of the silyl ether using HF·py to obtain **33** and subsequent treatment with glycosyl donor **5a**, with 8 mol% each of AgOTf and a gold–phosphite catalyst in CH_2_Cl_2_, afforded the required heptasaccharide **34** in 92% yield (Scheme 4).

**Scheme 5:**
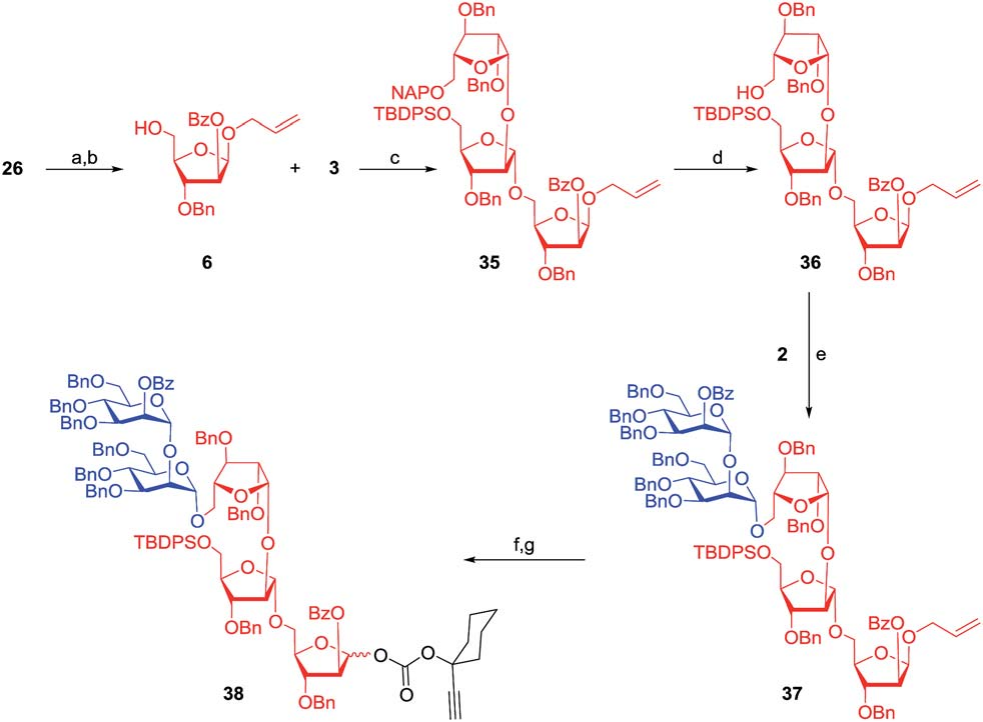
Reagents: (a) BzCl, pyridine, DMAP, 0–25 °C, 5 h, 93%; (b) HF·py, pyridine, 0–25 °C, 5 h, 94%; (c) 8 mol% chloro[tris(2,4-di-*tert*-butylphenyl)phosphite] gold, 8 mol% AgOTf, CH_2_Cl_2_, 4 Å MS powder, –78 °C, 5 h, 80% (overall yield 90% with α : β = 8 : 1); (d) DDQ, CH_2_Cl_2_–MeOH (1 : 4), 25 °C, 4 h, 82%; (e) 8 mol% chloro[tris(2,4-di-*tert*-butylphenyl)phosphite] gold, 8 mol% AgOTf, CH_2_Cl_2_, 4 Å MS powder, 25 °C, 15 min, 76%; (f) PdCl_2_, CH_2_Cl_2_–MeOH (1 : 4), 25 °C, 4 h; (g) **11**, DMAP, CH_2_Cl_2_, 0–25 °C, 3 h, 75% over two steps.

Protection of the C-2 hydroxyl group of compound **26** as a benzoate followed by the unblocking of the C-5 hydroxyl group by the addition of HF·py resulted in the glycosyl acceptor **6**. Subsequently, glycosyl acceptor **6** was treated with the donor **3** under gold–silver catalysis conditions to afford the required trisaccharide **35**.^35^ The diastereoselectivity of the reaction was noticed to be temperature dependent. The diastereomeric ratio swung in favour of the desired α-isomer as the temperature of the reaction was lowered. The best 8 : 1 ratio (α : β) in favour of the desired isomer with an overall yield of 90% (which translates to 80% of the trisaccharide **35**) was accomplished at –78 °C and that might be due to the presence of a bulky 5-*O*-TBDPS moiety (Scheme 5).^36^ The naphthyl moiety was deprotected using DDQ and CH_2_Cl_2_–MeOH (1 : 4) at 25 °C to obtain the acceptor **36** which was further treated with the mannopyranosyl donor **2** to obtain the pentasaccharide **37** in 76% yield. A two step conversion transformed allyl glycoside **37** into the glycosyl donor **38** in 75% yield (Scheme 5).

A glycosidation reaction between donor **3** and acceptor **4** afforded another pentasaccharide **39** as an allyl glycoside in 77% yield in the presence of 8 mol% each of Au–phosphite and AgOTf. The stereochemical outcome of glycosidation was noticed to be temperature dependent. Careful analysis of the glycosidation revealed that the mixture of glycosides (α : β = 8 : 1) results from the C-5 position of the acceptor, but not from the C-3 position of acceptor **4**.

**Scheme 6:**
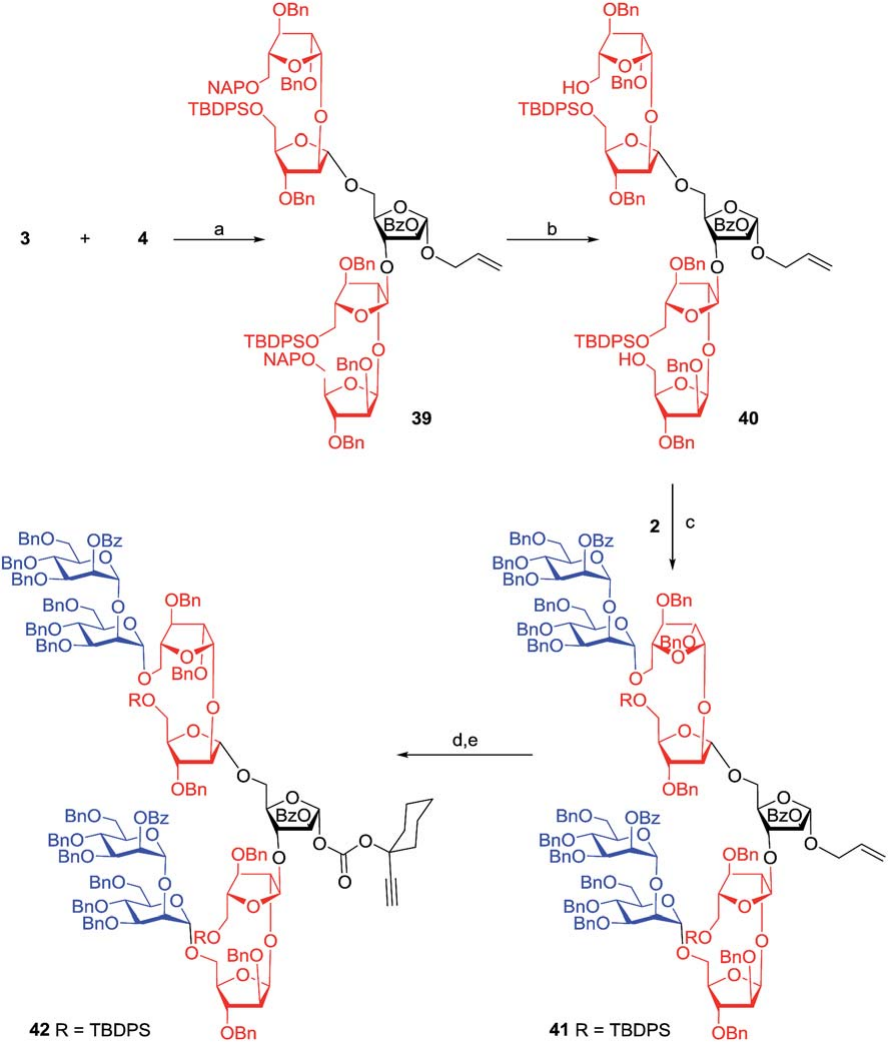
Reagents: (a) 8 mol% chloro[tris(2,4-di-*tert*-butylphenyl)phosphite] gold, 8 mol% AgOTf, CH_2_Cl_2_, 4 Å MS powder, –78 °C, 5 h, 77% (overall yield 87% with α : β = 8 : 1); (b) DDQ, CH_2_Cl_2_–MeOH (1 : 4), 25 °C, 4 h, 75%; (c) 8 mol% chloro[tris(2,4-di-*tert*-butylphenyl)phosphite] gold, 8 mol% AgOTf, CH_2_Cl_2_, 4 Å MS powder, 25 °C, 15 min, 60%; (d) PdCl_2_, CH_2_Cl_2_–MeOH (1 : 4), 25 °C, 4 h; (e) **11**, DMAP, CH_2_Cl_2_, 0–25 °C, 3 h, 76% over two steps.

Gratifyingly, the mixture of pentasaccharides could be separated using flash silica gel column chromatography to obtain 77% of the desired pentasaccharide **39**.^15^ Deprotection of the NAP-ether was achieved to afford the diol **40** which upon treatment with mannopyranosyl donor **2** gave nonasaccharide **41**. In the ^13^C NMR spectrum of the nonasaccharide **41**, resonances due to nine anomeric carbons were noticed at *δ* = 98.6, 98.8, 99.7, 99.8, 100.0, 100.5, 105.1, 105.5, and 106.8 ppm. Subsequently, the nonasaccharide was converted into the corresponding carbonate glycosyl donor **42** in 76% yield (Scheme 6).

**Scheme 7:**
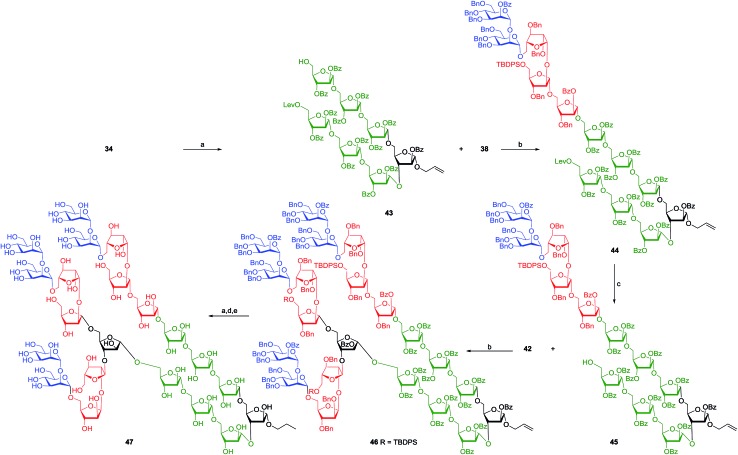
Reagents: (a) HF·py, pyridine, 0–25 °C, 5 h, 83% for **43** and 80% for **47**; (b) 8 mol% chloro[tris(2,4-di-*tert*-butylphenyl)phosphite] gold, 8 mol% AgOTf, CH_2_Cl_2_, 4 Å MS powder, 25 °C, 30 min, 85% for **44** and 80% for **46**; (c) hydrazine acetate, THF–MeOH (4 : 1), 25 °C, 45 min, 80%; (d) NaOMe, MeOH, 25 °C, 15 h, 87%; (e) Pd(OH)_2_, CH_3_OH–THF–H_2_O (4 : 3 : 3), H_2_, 36 h, 78%.

The final assembly of heneicosaarabinomannan **1** started with the deprotection of silyl ether **34** using HF·py to afford alcohol **43** which was glycosylated with donor **38** using 8 mol% each of gold–phosphite and AgOTf to afford dodecassacharide **44** in 85% yield. Twelve characteristic resonances due to anomeric carbons were noticed in the anomeric region (*δ* = 98.7–106.2 ppm) of the NMR spectrum.^46^ The lone levulinoate was hydrolysed with hydrazine acetate in THF–MeOH to afford the required glycosyl acceptor **45**.^45^ The final glycosidation between acceptor **45**, containing twelve saccharide residues, and the glycosyl donor **42**, containing nine carbohydrate residues, was performed in the presence of 8 mol% each of Au–phosphite and AgOTf resulting in the formation of the fully protected heneicosaarabinomannan **46** in 80% yield. In the ^13^C NMR spectrum of arabinomannan **46**, resonances due to the anomeric carbons were noticed as two sets centred on *δ* = 98.8–100.7 ppm and *δ* = 105.1–107.3 ppm for 21-anomeric carbons (Scheme 7).^4^ Oligosaccharide **46** can be subjected to a variety of reactions in order to attach biomolecules; however, the global deprotection of compound **46** was considered to show that the molecule is stable under the conditions employed for the cleavage of protecting groups. Cleavage of the three silyl ethers was carried out using the HF·py, Zemplén debenzoylation resulted in the saponification of eighteen benzoates and the final hydrogenolysis with Pd(OH)_2_/H_2_ caused the deprotection of twenty eight benzyl ethers and the reduction of the olefin as well affording the heneicosaarabinomannan (**47**) as its propyl glycoside.

## Conclusions

In summary, the execution of this highly convergent and modular strategy has led to the first synthesis of a branched, hybrid and complex arabinomannan (containing 15-Ara*f* and 6-Man*p*-residues) from the *Mycobacterium tuberculosis* cell wall in sufficient amounts for biological explorations with an overall yield of 0.016%. Stable alkynyl carbonate glycosyl donors are shown to be versatile glycosyl donors for the synthesis of large oligosaccharides. [Au]/[Ag]-catalytic conditions were employed for all the key glycosylations. 1,2-*cis* Ara*f* and some 1,2-*trans* Ara*f* linkages were installed taking advantage of reciprocal donor–acceptor selectivity. Taken together, the synthesis uses stable reactants and catalytic quantities of noble metal salts; therefore, it is experimentally less demanding and operationally convenient.
