# Supplementary material for: Expedient synthesis of the heneicosasaccharyl mannose capped arabinomannan of the Mycobacterium tuberculosis cellular envelope by glycosyl carbonate donors
Source: Chem Sci. 2016 Nov 15;8(3):2033–8. doi: 10.1039/c6sc04866h (PMC5398307; doi:10.1039/c6sc04866h)

Electronic Supplementary Material (ESI) for Chemical Science.
This journal is © The Royal Society of Chemistry 2016

Expedient Synthesis of Heneicosasaccharyl Mannose Capped Arabinomannnan of Mycobacterium tuberculosis Cellular Envelope by Glycosyl Carbonate Donors

Maidul Islam, Ganesh P. Shinde and Srinivas Hotha*

Department of Chemistry, Indian Institute of Science Education and Research, Dr. Homi Bhabha Road, Pune – 411 008

Ph. +91 20 2590 8015, E-mail: s.hotha@iiserpune.ac.in

Supporting Information

Contents

	Page No
Materials and Methods	S2
Experimental Procedures	S3-S11
Compound Characterization Data	S12-S29
^{1}H-, ^{13}C-, 2D-NMR Spectral Charts	S30-S157

Experimental Section

Materials and Methods

Unless otherwise noted, materials were obtained from commercial suppliers and were used without further purification. Unless otherwise reported all reactions were performed under argon atmosphere. Removal of solvent in vacuo refers to distillation using a rotary evaporator attached to an efficient vacuum pump. Products obtained as solids or syrups were dried under high vacuum. Yields in the glycosidation reaction are compute based on the quantity of acceptor. Analytical thin-layer chromatography was performed on pre-coated silica plates (F_{254}, 0.25 mm thickness); compounds were visualized by UV light or by staining with anisaldehyde spray. Optical rotations were measured on a digital polarimeter. IR spectra were recorded on a FT-IR spectrometer. NMR spectra were recorded either on a 400 or a 500 MHz with $CDCl_3$ as the solvent and TMS as the internal standard. High resolution mass spectroscopy was performed using ESI-TOF or MALDI-TOF mass analyzer. Low resolution mass spectroscopy (LRMS) was performed on UPLC-MS with TLC interface.

Characterization data for carbonate glycosyl donors was provided as the ratio of diastereomers are not required to be separated. The out come of the glycosylation does not depend on the initial ratio of glycosyl donor. However, for confirmation, we have provided spectra charts of corresponding glycosyl carbonates which show that the donors that we are prepared are pure.

The diastereoselectivity of glycosidation reactions was confirmed from the ratio of integration in the ^{1}H NMR and corresponding ^{13}C NMR spectra. In the ^{13}C NMR spectrum, resonances due to the α-anomeric arabinofuranosidic linkages are identified at δ 103-109 ppm whereas β-anomeric arabinofuranosidic linkages are noticed at δ 97-102 ppm. 1,2-*trans* or α-anomeric mannopyranosidic linkages are identified based on the $^{1}J_{C\text{-}H}$ values in the 2D-NMR spectrum which showed ~170Hz.

Experimental procedures

Procedure A: *Synthesis of ethynyl cyclohexyl glycosyl carbonate donors* (**2**,**3**,**5a**,**5b**,**12**,**19**,**23**,**38**,**42**): To a rapidly stirring CH_2Cl_2 solution of arabinofuranosyl or mannopyranosyl hemiacetal (1.0 mmol) and DMAP (1.5 mmol) at 25 ^{0}C, ethynyl cyclohexyl (4-nitrophenyl) carbonate (**11**) (1.2 mmol) was added and continued stirring at 25 ^{0}C for 2 h. After complete consumption of the hemiacetal, the reaction mixture was concentrated in vacuo and purified by silica gel column chromatography (n-hexane/EtOAc) to afford ethynyl cyclohexyl glycosyl carbonate donors containing trace quantity of the 4-nitro phenol. Eluents containing the compound are concentrated and redissolved in CH_2Cl_2 and washed with sat. aq. $NaHCO_3$ solution to get **2**,**3**,**5a**,**5b**,**12**,**19**,**23**,**38**,**42** in 85-90% yield.

Procedure B: G*lycosylation using ethynyl cyclohexyl glycosyl carbonate donors*: To a solution of acceptor (**4**,**6**,**9**,**14**,**18**,**26**,**31**,**33**,**36**,**40**,**43**,**45**) (1.0 mmol) and donor (**2**,**3**,**5a**,**5b**,**12**,**19**,**23**,**38**,**42**) (1.0 mmol) in anhydrous CH_2Cl_2 (15 mL) was added freshly activated 4Å MS powder (0.400 g) at 25 ^{0}C. After stirring at 25 °C for 10 minutes chloro[tris(2,4-di-*tert*-butylphenyl)phosphite]gold(I) (8mol%) and AgOTf (8mol%) were added to the reaction mixture and stirred for 15-25 minutes if the reaction was carried out at 25 °C and 5-8 h for reactions at -78 °C, the reaction was neutralized by Et_3N and filtered through a bed of Celite®. The filtrate was concentrated in vacuo and the residue was purified by silica gel column chromatography using *n*-hexane/EtOAc as mobile phase to afford glycosides in 80-95% yield.

Procedure C: *Protection of alcohol using TBDPS-Cl*:

A solution of the allylarabinofuranoside (1 mmol) and imidazole (1.3 mmol) in anhydrous DMF (5 mL) was cooled at 0 °C and *tert*-butyldiphenylchlorosilane (1.2 mmol) was added dropwise under nitrogen atmosphere. The reaction mixture was gradually warmed up to 25 ^{o}C and stirred for 2 h. After the completion, the reaction mixture was quenched by adding ice cold water. The whole mixture was transferred to a separatory funnel and extracted with ethyl acetate (3x25 mL). The combined organic layers was washed with cold water and brine solution. Organic layer was dried over anhydrous Na_2SO_4, the organic layer was concentrated *in vacuo* and the residue was purified by silica gel column chromatography (ethyl acetate/hexanes) to obtain the desired product.

Procedure D: *Deprotection of the TBDPS-ethers:*

HF·py (3 mmol) was added dropwise under inert atmosphere to a solution of silyl ether (1 mmol) in pyridine (3 times to the volume of HF·py) at 0 ^{o}C. The reaction mixture was stirred for 5 h at 25 ^{o}C, 6*N* HCl was added to quench the reaction at 0 ^{o}C, diluted with EtOAc (25 mL) and washed successively with ice cold water (2x10 mL), saturated solution of $NaHCO_3$ (aq), and brine solution. Organic layer was dried anhydrous Na_2SO_4, concentrated *in vacuo*

to obtain a residue that was purified by silica gel column chromatography (ethyl acetate/hexanes) to furnish the alcohol.

Procedure E: *Protection of alcohols as benzyl ethers:*

To a solution of the alcohol (1 mmol) in anhydrous DMF (5 mL) was cooled to 0 °C under nitrogen atmosphere and NaH (60% oil dispersion, 1.3mmol per alcohol) was added and stirred for 10 minute at 0 ^{o}C, benzyl bromide (1.2 mmol per alcohol) was added dropwise and stirred for 2 h at 25 ^{o}C. The reaction mixture was poured into cold water with vigorous shaking, extraction with ethyl acetate (3x25 mL) and combined EtOAc layers was washed with cold water, brine solution, and dried over Na_2SO_4. The EtOAc solution was decanted and evaporated *in vacuo* to obtain a reddish brown coloured residue that was purified by column chromatography (ethyl acetate/hexanes) to obtain the benzyl ether(s) as pale yellow coloured syrup**.**

Procedure F: *Synthesis of Allyl glycoside from 1,2-orthoester:*

Ally alcohol (4 mmol) followed by PTSA (0.2 mmol) were added to a vigorously stirred solution of orthoester (1 mmol) in freshly prepared anhydrous CH_2Cl_2 (10 mL) at 25 ^{o}C. After completion of the reaction, Et_3N (2 mL) was added and the solvent was evaporated *in vacuo* and the crude compound was purified by conventional silica gel column chromatography (ethyl acetate/hexanes) to obtain the desired allyl glycoside**.**

procedure G: *Deprotection of allyl glycosides:*

To the solution of the allyl glycoside (1 mmol) in CH_2Cl_2 (10 mL), $PdCl_2$ (0.2mmol) in MeOH (40mL) was added in five portions at 25 ^{o}C and stirred for 4-5 h. After completion of the reaction, excess amount of Et_3N was added and the solid residue was filtered off through a pad of Celite®. The solvent was evaporated *in vacuo* and the crude compound was purified by conventional silica gel column chromatography (ethyl acetate/hexanes) to obtain the desired hemiacetal**.**

Procedure H: *Protection of levulinoate:*

A solution of the alcohol (1 mmol), DMAP (0.5 mmol) and levulinic acid (1.3 mmol) in dry CH_2Cl_2 (10 mL) was cooled to 0 °C and *N*,*N*'-Diisopropylcarbodiimide (1.5 mmol) was added dropwise under nitrogen atmosphere. The reaction mixture was gradually warmed to room temperature and stirred for 2 h. After completion of the reaction, CH_2Cl_2 was evaporated under reduced pressure and the residue was purified by silica gel column chromatography (ethyl acetate/hexanes) to furnish the Lev ester.

Procedure I: *Deprotection of levulinoate:*

Hydrazine acetate (4 mmol) in THF (4 mL) was added to a solution of levulinoate (1 mmol) in MeOH (1 mL) at 25 ^{o}C and stirred for 45 minutes. The reaction was quenched by adding ice cold 1N HCl solution and washed successively with ethyl acetate (3x25 mL), water, saturated solution of $NaHCO_3$ (aq), and brine solution. After drying over Na_2SO_4, the organic layer was concentrated under diminished pressure and the residue was purified by column chromatography (ethyl acetate/hexanes) to afford the alcohol.

Procedure J: *Protection of alcohols as NAPethers:* [A. B. Smith, K. Basu, T. Bosanac, *J. Am. Chem. Soc.*, 2007, **129**, 14872]

To a solution of the alcohol (1 mmol) in anhydrous DMF (5 mL) was cooled to 0 °C under nitrogen atmosphere and NaH (60% oil dispersion, 1.3mmol per alcohol) was added and stirred for 10 minute at 0 ^{o}C, 2-bromomethyl naphthalene (1.2 mmol per alcohol) was added dropwise and stirred for 2 h at 25 ^{o}C. The reaction mixture was poured into cold water with vigorous shaking, extraction with ethyl acetate (3x25 mL) and combined EtOAc layers was washed with cold water, brine solution, and dried over Na_2SO_4. The EtOAc solution was decanted and evaporated *in vacuo* to obtain a reddish brown coloured residue that was purified by column chromatography (ethyl acetate/hexanes) to obtain the benzyl ether(s) as pale yellow coloured syrup**.**

Procedure K*: Deprotection of NAP ethers:* [A. B. Smith, K. Basu, T. Bosanac, *J. Am. Chem. Soc.*, 2007, **129**, 14872]

2,3-Dichloro-5,6-dicyano-1,4-benzoquinone (2 mmol) was added to a rapidly stirred solution of alcohol (1 mmol) in CH_2Cl_2-MeOH (1:4) at 25 ^{o}C. After 4 h, the reaction mixture was quenched by the addition of Et_3N (reaction mixture turns black from brown) and solvent was evaporated under reduced pressure and the residue was purified by silica gel column chromatography (ethyl acetate/hexanes) to furnish the alcohol as a pale yellow coloured syrup.

Procedure L: *Synthesis of 1,2-acetonide of arabinose:*

2,2-Dimethoxy propane (1.25 mmol) and PTSA (0.2 mmol) were added to a vigorously stirring solution of *C*-5-*O*-TBDPS arabinofuranoside (1 mmol), activated 4Å MS in anhydrous CH_2Cl_2 (50 mL) and stirred for 24 h at 25 ^{o}C. After completion of the reaction, excess Et_3N was added, the solid residue was filtered off through Celite® bed. The solvent was evaporated *in vacuo* under reduced pressure and the crude compound was purified by column chromatography (ethyl acetate/hexanes) to obtain the desired ara*f*-1,2-acetonite**.**

Procedure M: *Synthesis of benzoates:*

A solution of the alcohol (1 mmol) in anhydrous pyridine (10 mL) and DMAP (0.1 mmol) was cooled to 0 °C under nitrogen atmosphere and benzoyl chloride (1.2 mmol per alcohol) was added dropwise and stirred for 5 h at 25 ^{o}C. The reaction mixture was poured into cold water with vigorous shaking, neutralized by the addition of 6N HCl solution, extracted with ethyl acetate (3x50 mL) and combined EtOAc layers was washed with cold water, sat. $NaHCO_3$, brine solution, and dried over Na_2SO_4. The EtOAc solution was decanted and evaporated *in vacuo* to obtain a reddish brown coloured residue that was purified by column chromatography (ethyl acetate/hexanes) to obtain the required benzoate.

Procedure N: *Saponification of benzoates:*

Freshly prepared NaOMe (0.1 mmol per benzoate) was added to a solution of the benzoate (1 mmol) in MeOH (10 mL) and stirred for 8 h at 25 ^{o}C. The reaction mixture was concentrated *in vacuo* to obtain a residue that was purified by column chromatography (ethyl acetate/hexanes) to obtain alcohol.

Procedure O: *Hydrogenolysis of compound* **46***:*

To a solution of the compound **46** (30 mg, 5.4 µmol) in 2 mL of MeOH-THF-H_2O (4:3:3) under Hydrogen atmosphere (balloon pressure) was added 10% $Pd(OH)_2$(2mg, 1.0 µmol) and stirred for 36 h at 25 ^{o}C. The reaction mixture was filtered through a pad of Celite® and the filtrate was evaporated *in vacuo* first and subsequently subjected to lyophilisation to afford the target compound **47** (14 mg, 90%) as a white solid.

Procedure P: *Synthesis of allyl arabinofuranoside from 1,2-acetonide:*

Allyl alcohol (2.12 mL, 30.8 mmol) was added to a solution of acetonide **25** (8.0 g, 15.4 mmol) in CH_2Cl_2 (60 mL), stirred at 60 ^{o}C. After 5 min, PTSA (0.53 g, 3.1 mmol) was added and continued stirring for additional 1 h, the reaction mixture was neutralized by the addition of Et_3N (excess), diluted with water and extracted with CH_2Cl_2 (3x50 mL). Combined CH_2Cl_2 layers was washed with a brine solution, dried over anhydrous sodium sulphate, decanted and concentrated *in vacuo* to obtain a residue that was partially purified by silica gel column chromatography (ethyl acetate/hexanes) to obtain α/β-allyl furanosides which were separated by flash chromatography by obtain diastereomerically pure **26** (3.6 g, 45%) as a pale yellow syrup.

Schemes Annotated with Procedures and Yields

Scheme 1

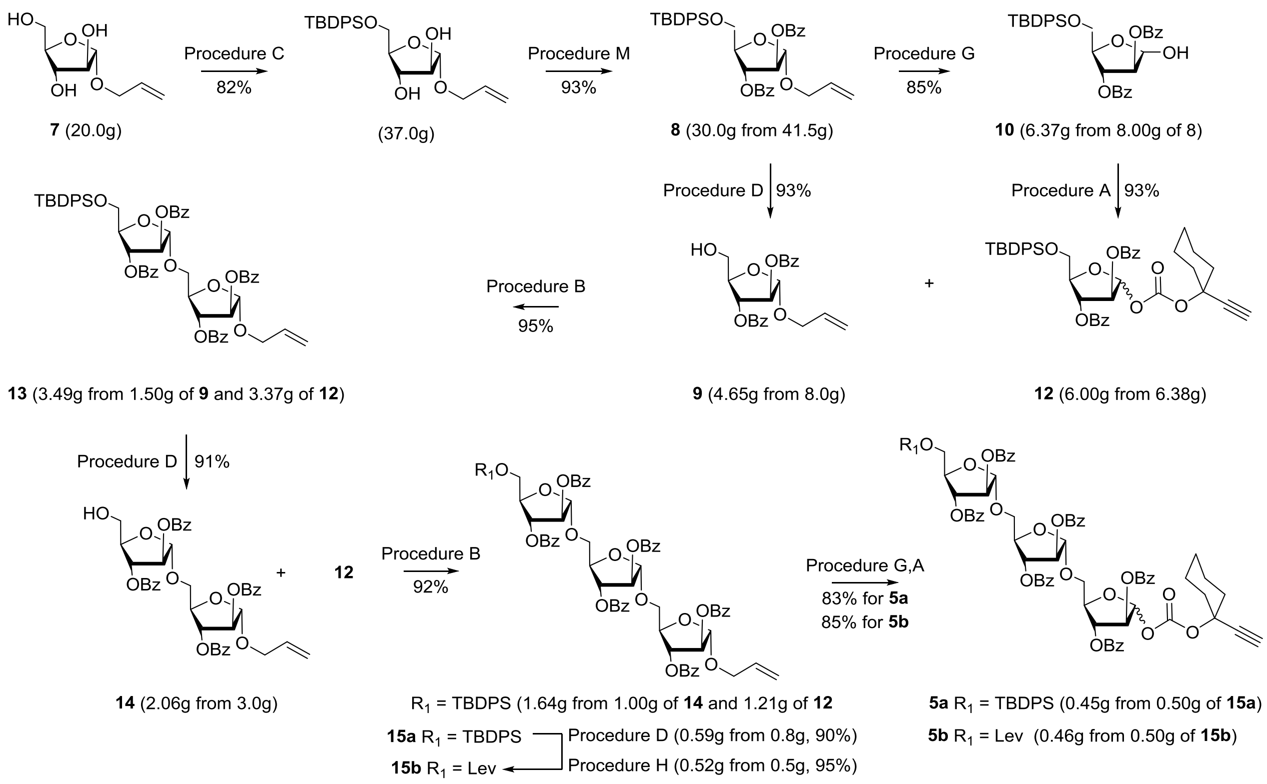

Scheme 2

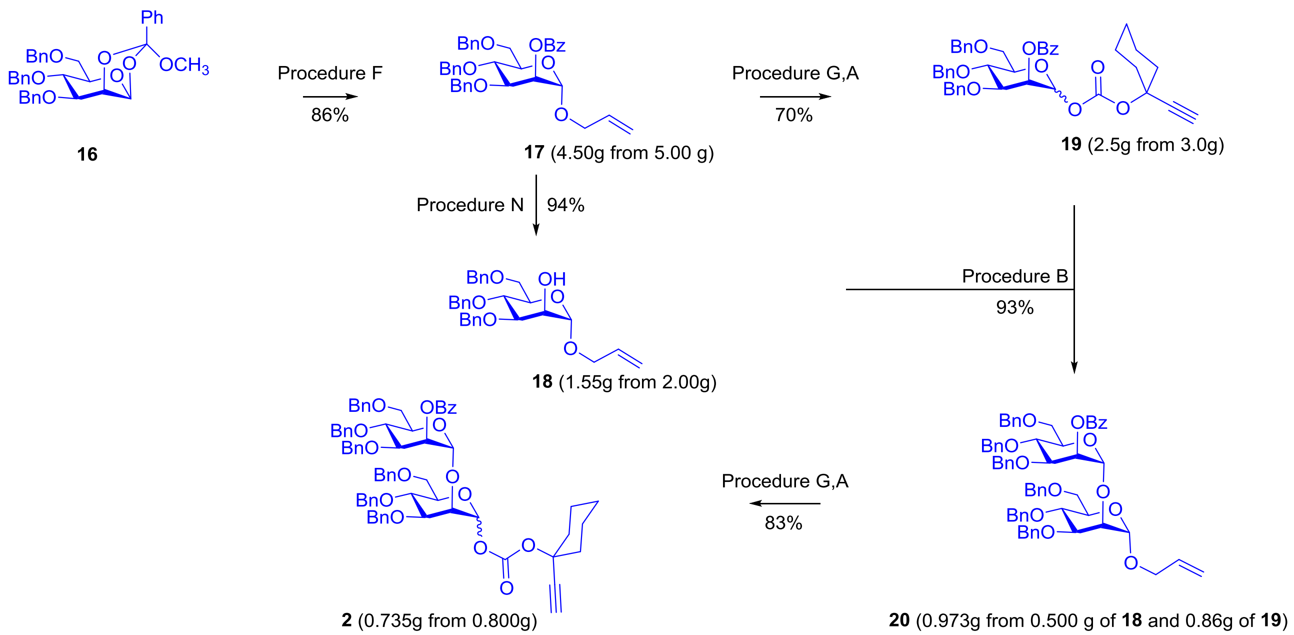

Scheme 3

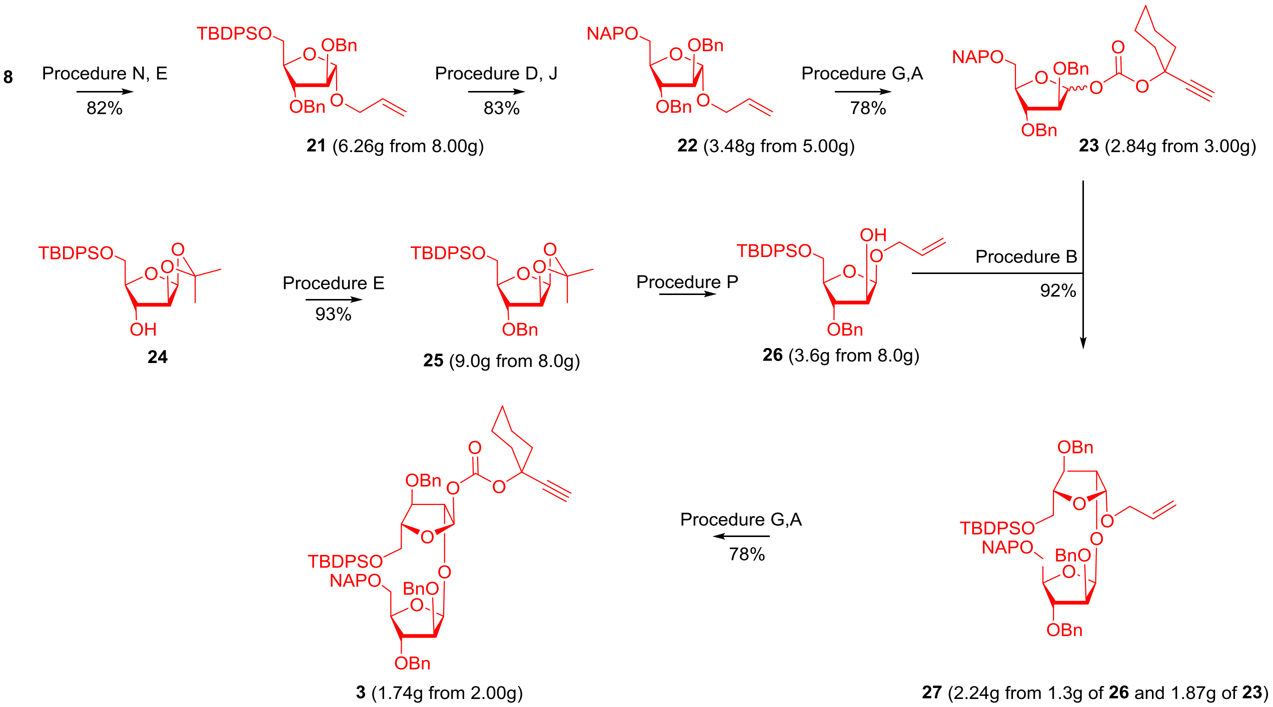

Scheme 4

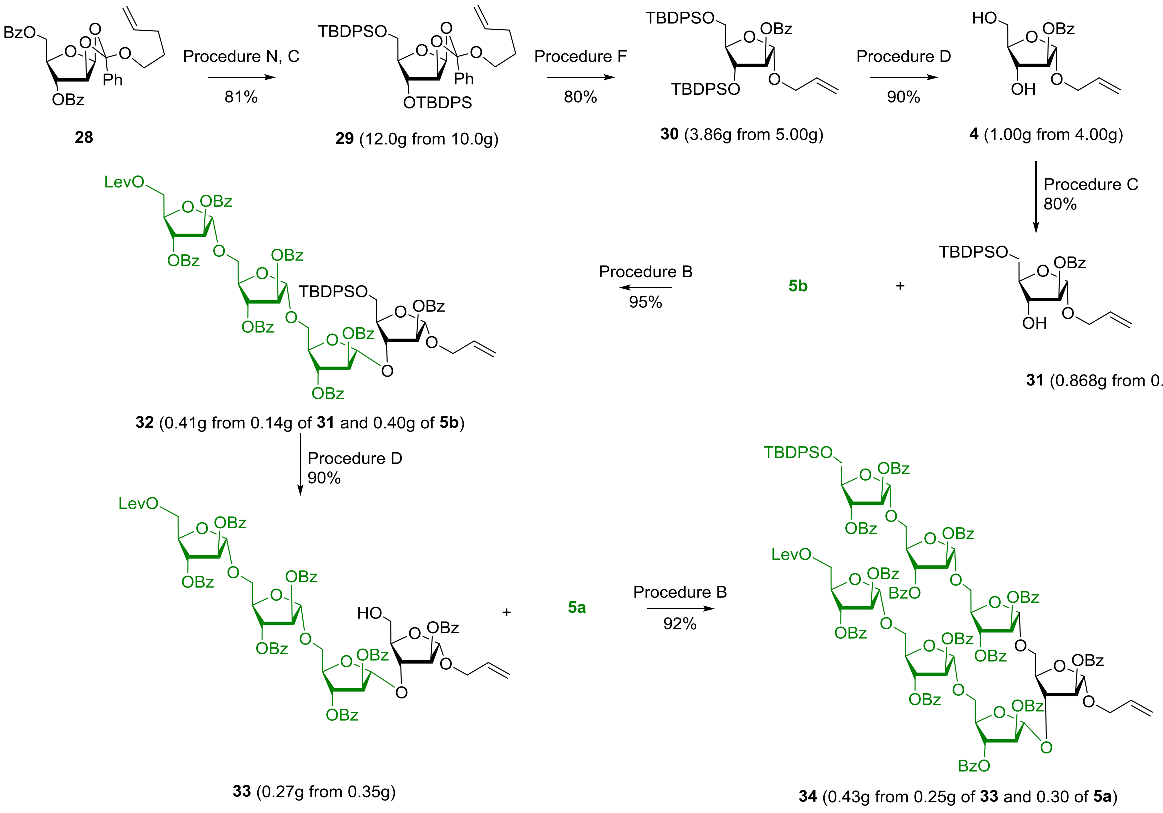

Scheme 5

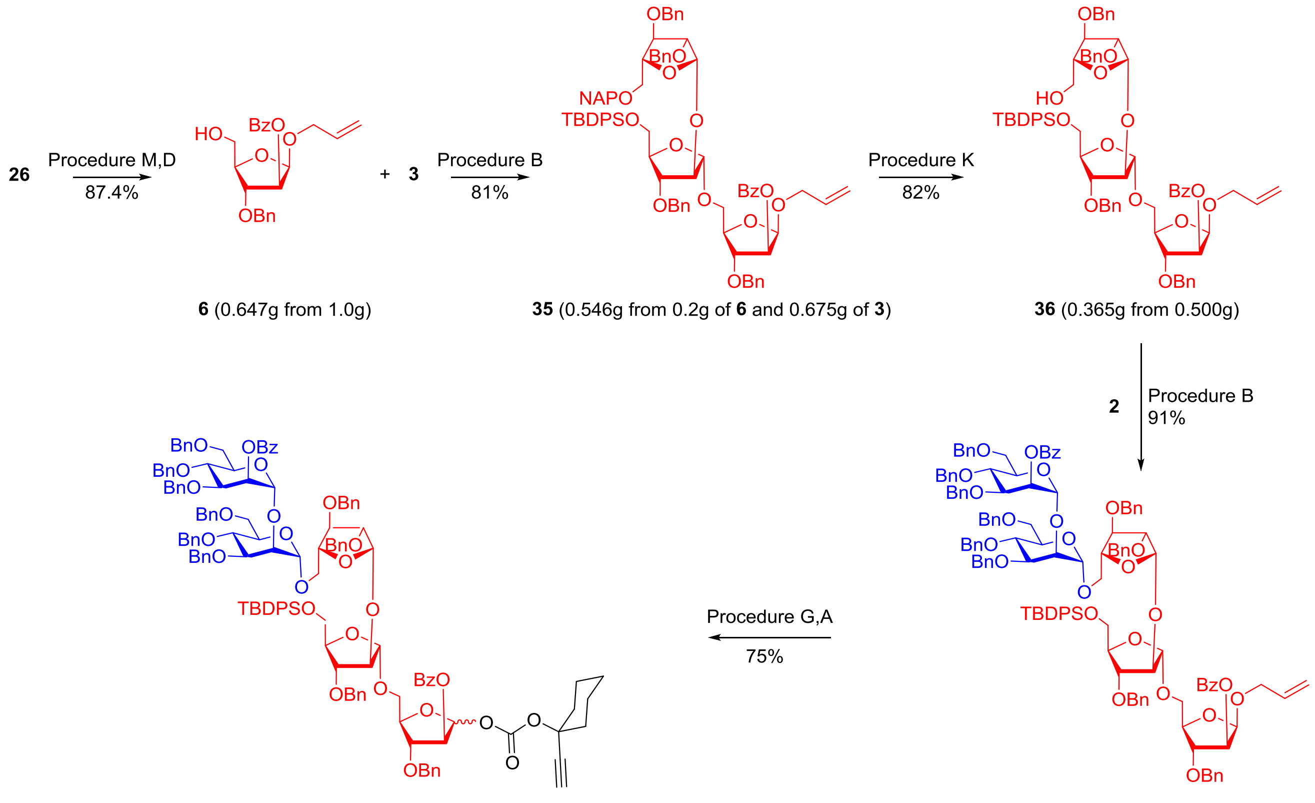

6 (0.647g from 1.0g)

35 (0.546g from 0.2g of 6 and 0.675g of 3)

36 (0.365g from 0.500g)

37 (0.334g from 0.2g of 36 and 0.235g of 2)

38 (0.236g from 0.300g)

Scheme 6

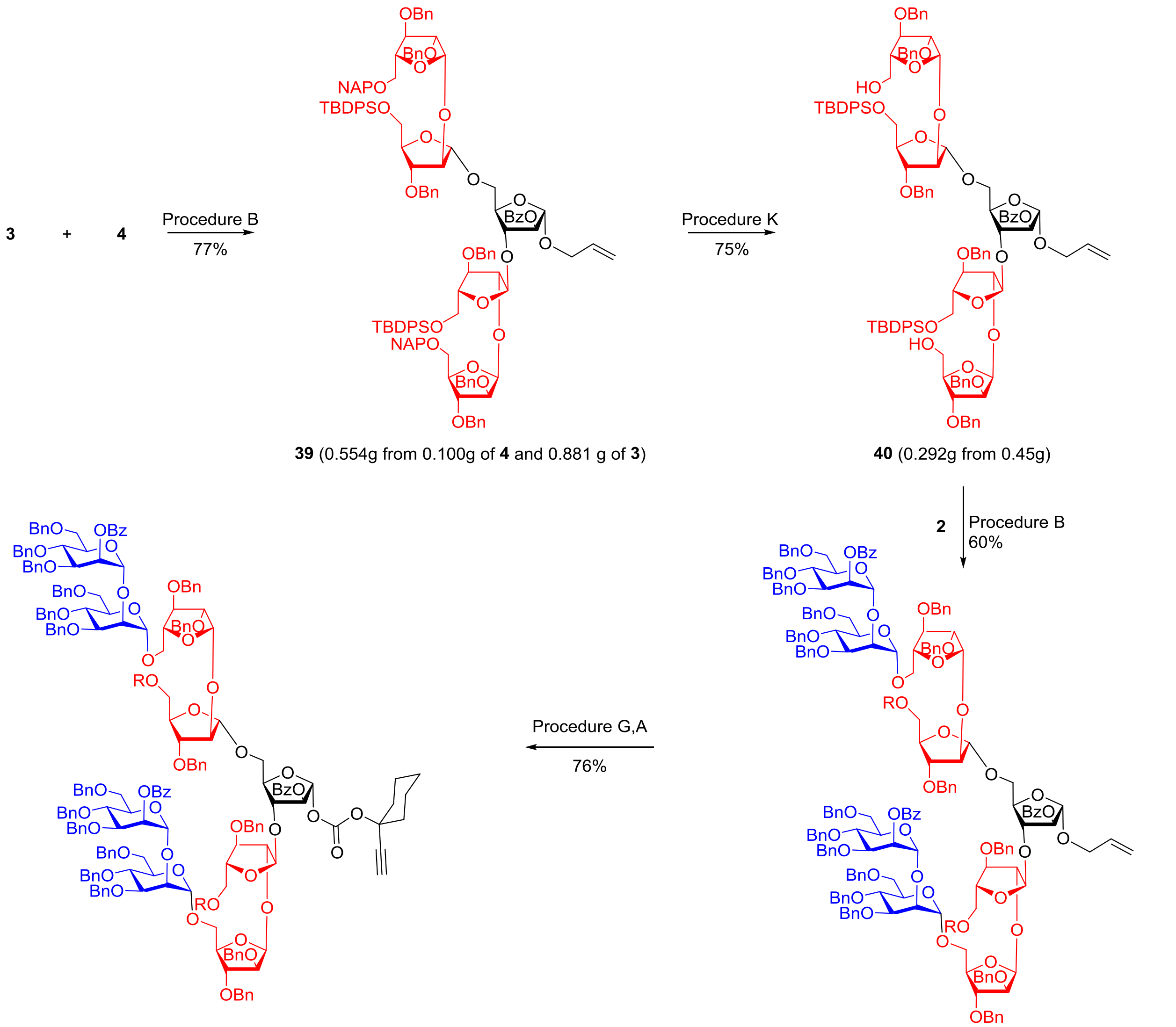

39 (0.554g from 0.100g of **4** and 0.881 g of **3**)

40 (0.292g from 0.45g)

42 R = TBDPS (0.156g from 0.200g)

41 R = TBDPS (0.246g from 0.200g of **40** and 0.309g of **2**)

Scheme 7

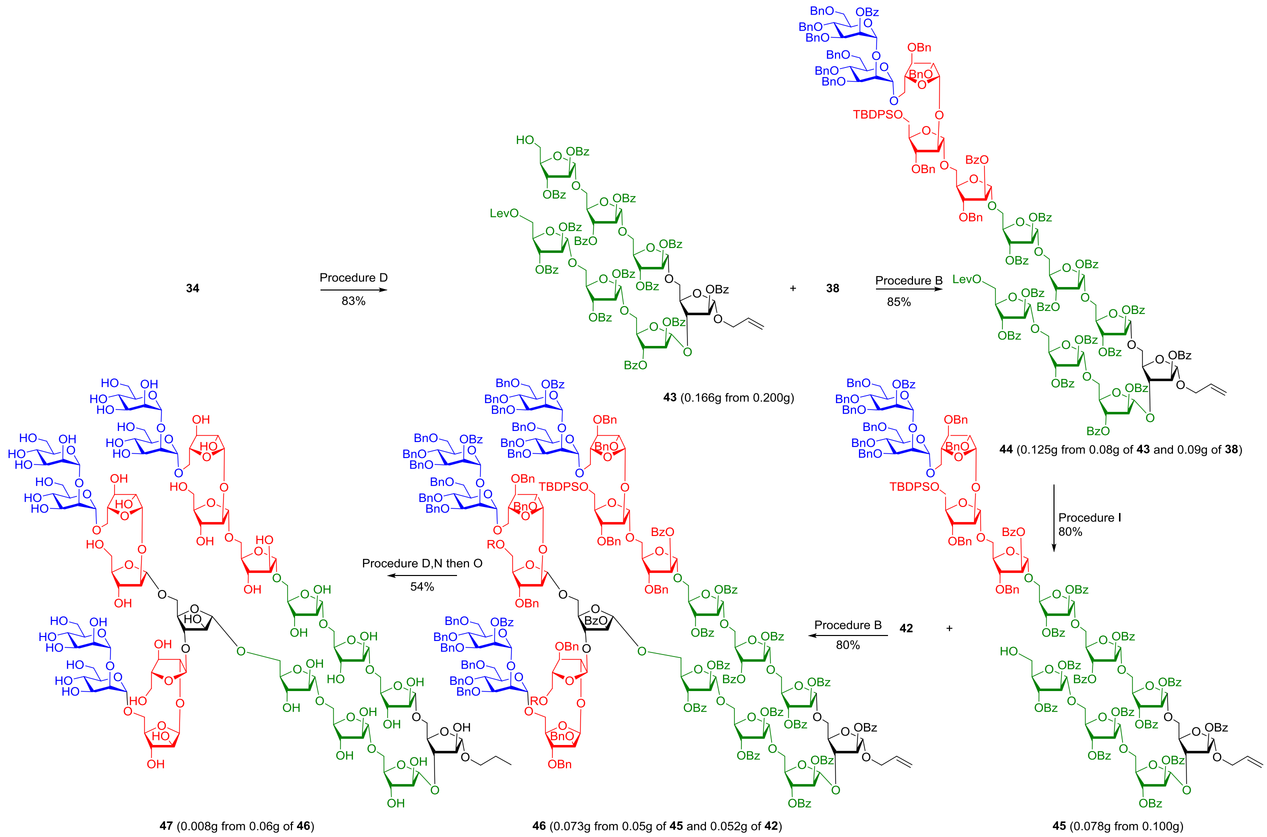
34
Procedure D
83%
43 (0.166g from 0.200g)
+
38
Procedure B
85%
44 (0.125g from 0.08g of 43 and 0.09g of 38)
Procedure I
80%
45 (0.078g from 0.100g)
42
+
Procedure B
80%
46 (0.073g from 0.05g of 45 and 0.052g of 42)
Procedure D,N then O
54%
47 (0.008g from 0.06g of 46)

Compound Characterization Data

Allyl 2,3-di-*O*-benzoyl-5-*O*-^{t}butyldiphenylsilyl-α-D-arabinofuranoside (**8**): Yield: (76% over two steps from **7**); $[\alpha]_D^{25}$ = -17.5 (*c* = 1.0, $CHCl_3$); ^{1}H NMR (399.78 MHz, $CDCl_3$): δ 1.05 (s, 9H), 4.01 (d, *J* = 4.8 Hz, 2H), 4.11 (ddt, *J* = 13.1, 6.1, 1.3 Hz, 1H), 4.29 (ddt, *J* = 13.1, 5.0, 1.5 Hz, 1H), 4.40 (q, *J* = 4.7 Hz, 1H), 5.20 – 5.24 (m, 1H), 5.26 (s, 1H), 5.37 (dq, *J* = 17.1, 1.6 Hz, 1H), 5.49 (d, *J* = 1.5 Hz, 1H), 5.62 (dd, *J* = 5.2, 1.4 Hz, 1H), 5.90 – 6.02 (m, 1H), 7.30 – 7.35 (m, 4H), 7.35 – 7.41 (m, 4H), 7.42 – 7.48 (m, 2H), 7.52 – 7.61 (m, 2H), 7.71 (ddd, *J* = 7.8, 3.0, 1.5 Hz, 4H), 7.96 – 8.01 (m, 2H), 8.04 – 8.08 (m, 2H); ^{13}C NMR (100.53 MHz, $CDCl_3$): δ 19.4, 26.9(3C), 63.7, 68.0, 77.6, 82.6, 83.1(2C), 105.1, 117.5, 127.8(3C), 128.5(3C), 129.4, 129.6, 129.8(2C), 130.1(3C), 133.3, 133.4(3C), 134.0, 135.8(6C), 165.6, 165.8; IR (CHCl3); 3439, 3029, 2930, 1599, 1454, 1214, 701 cm^{-1}; HRMS (TOF) m/z $[M + Na]^+$ calcd for $C_{38}H_{40}O_7NaSi$, 659.2441, found 659.2445.

Allyl 2,3-di-*O*-benzoyl-α-D-arabinofuranoside (**9**): Yield: (93%); $[\alpha]_D^{25}$ = -36.4 (*c* = 1.0, $CHCl_3$); ^{1}H NMR (399.78 MHz, $CDCl_3$): δ 2.69 (s, 1H), 4.03 (d, *J* = 12.9 Hz, 2H), 4.11 (ddt, *J* = 13.1, 5.9, 1.3 Hz, 1H), 4.29 (ddt, *J* = 13.1, 4.9, 1.5 Hz, 1H), 4.36 (q, *J* = 4.1 Hz, 1H), 5.21 (dd, *J* = 10.4, 1. 5 Hz, 1H), 5.30 (s, 1H), 5.37 (dq, *J* = 17.2, 1.6 Hz, 1H), 5.43 – 5.50 (m, 1H), 5.57 (d, *J* = 1.3 Hz, 1H), 5.88 – 6.00 (m, 1H), 7.43 (td, *J* = 7.7, 4.5 Hz, 4H), 7.52 – 7.62 (m, 2H), 7.87 – 8.17 (m, 4H); ^{13}C NMR (100.53 MHz, $CDCl_3$): δ 62.3 , 67.9 , 77.8 , 82.0 , 83.8(2C) , 104.7 , 117.4 , 128.5 , 128.5(2C), 129.1 , 129.2, 129.9 (2C), 129.9(2C), 133.5, 133.6, 133.7, 165.4, 166.2; IR (CHCl3): 3437, 3068, 2927, 1599, 1452, 1262, 1106, 709 cm^{-1}; HRMS (TOF) m/z $[M + Na]^+$ calcd. for $C_{22}H_{22}O_7Na$, 421.1263, found 421.1265.

Allyl 2,3-di-*O*-benzoyl-5-*O*-(2,3-di-*O*-benzoyl-5-*O*-^{t}butyldiphenylsilyl-α-D-arabinofuranosyl)-α-D-arabinofuranoside (**13**): Yield: (95%); $[\alpha]_D^{25}$ = -5.4 (*c* = 1.0, $CHCl_3$); ^{1}H NMR (399.78MHz, $CDCl_3$): δ 1.04 (s, 9H), 3.97 (dd, *J* = 11.2, 2.9 Hz, 1H), 4.02 (dd, *J* = 4.4, 1.8 Hz, 2H), 4.11 (ddt, *J* = 13.2, 5.9, 1.3 Hz, 1H), 4.23 (dd, *J* = 11.2, 4.6 Hz, 1H), 4.30 (ddt, *J* = 13.2, 4.9, 1.5 Hz, 1H), 4.51 – 4.59 (m, 2H), 5.20 (dd, *J* = 10.4, 1.5 Hz, 1H), 5.30 (s, 1H), 5.36 (dq, *J* = 17.3, 1.6 Hz, 1H), 5.41 (s, 1H), 5.58 (d, *J* = 1.2 Hz, 1H), 5.61 (d, *J* = 1.2

Hz, 1H), 5.67 (t, *J* = 5.6 Hz, 2H), 5.88 – 6.03 (m, 1H), 7.28 – 7.47 (m, 16H), 7.51 – 7.58 (m, 2H), 7.72 (ddd, *J* = 7.8, 3.6, 1.7 Hz, 4H), 7.92 – 8.02 (m, 6H), 8.07 (dd, *J* = 8.3, 1.2 Hz, 2H); ^{13}C NMR (100.53 MHz, $CDCl_3$): δ 19.4, 26.8(3C), 63.5, 66.1, 67.8(2C), 67.8, 77.5 (2C), 82.0, 82.0, 82.2, 83.3, 104.9, 106.0, 117.4, 127.7(3C), 128.3(2C), 128.4(2C), 128.5(2C), 128.5(2C), 129.1, 129.3, 129.3, 129.4, 129.7(2C), 129.8(2C), 129.9(2C), 130.0(2C), 130.0(2C), 133.2, 133.3, 133.3, 133.4, 133.4(2C), 133.8, 135.7(2C), 135.7(2C), 165.3, 165.4, 165.6, 165.8; IR ($CHCl_3$): 3430, 3029, 2929, 1599, 1444, 1214, 699 cm^{-1}; HRMS (TOF) m/z $[M + Na]^+$ calcd for $C_{57}H_{56}O_{13}NaSi$, 999.3387, found 999.3392.

Allyl 2,3-di-*O*-benzoyl-5-*O*-(2,3-di-*O*-benzoyl-α-D-arabinofuranosyl)-α-D-arabinofuranoside (**14**): Yield: (91%); $[\alpha]_D^{25}$ = -10.8 (c = 1.0, $CHCl_3$); ^{1}H NMR (399.78MHz, $CDCl_3$): δ 2.47 (s, 1H), 3.97 (dd, *J* = 11.2, 2.8 Hz, 2H), 4.09 (ddt, *J* = 13.2, 6.0, 1.4 Hz, 1H), 4.21 (dd, *J* = 11.2, 4.6 Hz, 1H), 4.28 (ddt, *J* = 13.2, 4.9, 1.6 Hz, 1H), 4.48 (dt, *J* = 4.7, 2.4 Hz, 1H), 4.51 (q, *J* = 4.2 Hz, 1H), 5.20 (dq, *J* = 10.5, 1.4 Hz, 1H), 5.29 (s, 1H), 5.33 (q, *J* = 1.6 Hz, 1H), 5.37 (q, *J* = 1.6 Hz, 1H), 5.42 (s, 1H), 5.43 – 5.46 (m, 1H), 5.57 (d, *J* = 1.3 Hz, 1H), 5.64 (d, *J* = 5.1 Hz, 1H), 5.66 (d, *J* = 1.3 Hz, 1H), 5.87 – 6.00 (m, 1H), 7.24 – 7.33 (m, 2H), 7.37 – 7.47 (m, 7H), 7.48 – 7.54 (m, 1H), 7.57 (t, *J* = 7.4 Hz, 2H), 7.94 (dd, *J* = 8.3, 1.2 Hz, 2H), 8.00 – 8.10 (m, 6H); ^{13}C NMR (100.53 MHz, $CDCl_3$): δ 62.4, 66.2, 67.9, 77.4, 77.8, 81.7, 82.0(2C), 83.8(2C), 104.9, 105.8, 117.4, 128.4(2C), 128.5(2C), 128.6(3C), 129.1, 129.1, 129.2, 129.3, 129.9(4C), 129.9(2C), 130.0(2C), 133.4, 133.5, 133.5, 133.6, 133.8, 165.2, 165.5, 165.8, 166.1; IR ($CHCl_3$): 3459, 3067, 2928, 1599, 1452, 1281, 1106, 706 cm^{-1}; HRMS (TOF) m/z $[M + Na]^+$ calcd for $C_{41}H_{38}O_{13}Na$, 761.2209, found 761.2211.

Allyl 2,3-di-*O*-benzoyl-5-*O*-(2,3-di-*O*-benzoyl-5-*O*-(2,3-di-*O*-benzoyl-5-*O*-^{t}butyldiphenylsilyl-α-D-arabinofuranosyl)-α-D-arabinofuranosyl)-α-D-arabinofuranoside (**15a**): Yield: (92%); $[\alpha]_D^{25}$ = -0.8 (c = 1.0, $CHCl_3$); ^{1}H NMR (399.78 MHz, $CDCl_3$): δ 1.02 (s, 9H), 3.91 – 4.01 (m, 4H), 4.09 (ddt, *J* = 13.2, 6.0, 1.4 Hz, 1H), 4.21 (ddd, *J* = 11.0, 6.4, 4.5 Hz, 2H), 4.28 (ddt, *J* = 13.2, 4.9, 1.6 Hz, 1H), 4.48 (dt, *J* = 4.7, 2.3 Hz, 1H), 4.51 (q, *J* = 4.7 Hz, 1H), 4.66 (q, *J* = 4.2 Hz, 1H), 5.17 – 5.24 (m, 1H), 5.28 (s, 1H), 5.35 (dd, *J* = 17.2, 1.7 Hz, 1H), 5.41 (d, *J* = 3.9 Hz, 2H), 5.57 (d, *J* = 1.3 Hz, 1H), 5.58 (d, *J* = 1.3 Hz, 1H), 5.62 –

5.67 (m, 4H), 5.93 (dddd, *J* = 17.2, 10.6, 5.9, 4.9 Hz, 1H), 7.25 – 7.40 (m, 17H), 7.41 – 7.57 (m, 7H), 7.70 (ddd, *J* = 7.9, 4.2, 1.7 Hz, 4H), 7.89 – 8.08 (m, 12H); ^{13}C NMR (100.53 MHz, $CDCl_3$): δ 19.4, 26.9(3C), 63.5, 66.0, 66.0, 67.9, 77.4(3C), 81.6, 82.0(2C), 82.2, 82.3, 83.3(2C), 104.9, 105.9, 106.1, 117.4, 127.8(3C), 128.3(2C), 128.4 (2C), 128.4(2C), 128.5(2C), 128.5(2C), 128.6(2C), 129.1, 129.2, 129.3, 129.3, 129.4(2C), 129.7(2C), 129.9(4C), 129.9(4C), 130.0(2C), 130.0(2C), 133.1, 133.3, 133.3, 133.4(2C), 133.4, 133.5, 133.5, 133.9, 135.7(2C), 135.7(2C), 165.2, 165.3, 165.5, 165.6, 165.7, 165.8; IR ($CHCl_3$): 3468, 3049, 2938, 1601, 1450, 1230, 1109, 697 cm^{-1}; HRMS (TOF) m/z [M + Na]$^+$ calcd for $C_{76}H_{72}O_{19}NaSi$, 1339.4334, found 1339.4347.

Allyl 2,3-di-*O*-benzoyl-5-*O*-(2,3-di-*O*-benzoyl-5-*O*-(2,3-di-*O*-benzoyl-5-*O*-(4-oxopentanoyl)-α-D-arabinofuranosyl)-α-D-arabinofuranosyl)-α-D-arabinofuranoside (**15b**): Yield: (88% over two steps); $[\alpha]_D^{25}$ = 0.1 (*c* = 1.0, $CHCl_3$); ^{1}H NMR (399.78MHz, $CDCl_3$): δ 2.12 (s, 3H), 2.55 – 2.61 (m, 2H), 2.67 – 2.73 (m, 2H), 3.95 (ddd, *J* = 13.8, 11.3, 2.8 Hz, 2H), 4.09 (ddt, *J* = 13.2, 6.0, 1.4 Hz, 1H), 4.21 (dt, *J* = 11.0, 4.8 Hz, 2H), 4.26 (dt, *J* = 4.9, 1.6 Hz, 1H), 4.30 (dt, *J* = 4.9, 1.6 Hz, 1H), 4.40 (dd, *J* = 11.5, 5.0 Hz, 1H), 4.47 (td, *J* = 4.6, 2.9 Hz, 2H), 4.54 – 4.61 (m, 1H), 4.63 (dt, *J* = 5.8, 2.8 Hz, 1H), 5.20 (dq, *J* = 10.5, 1.4 Hz, 1H), 5.28 (s, 1H), 5.35 (dq, *J* = 17.2, 1.7 Hz, 1H), 5.39 – 5.42 (m, 2H), 5.45 (s, 1H), 5.56 (d, *J* = 1.3 Hz, 1H), 5.60 (d, *J* = 1.1 Hz, 1H), 5.62 – 5.67 (m, 2H), 5.94 (dddd, *J* = 17.1, 10.6, 5.9, 4.9 Hz, 1H), 7.27 (t, *J* = 7.7 Hz, 4H), 7.36 – 7.47 (m, 10H), 7.51 (tdd, *J* = 8.3, 3.5, 2.2 Hz, 2H), 7.58 (dtt, *J* = 10.2, 7.0, 1.3 Hz, 2H), 7.89 – 7.94 (m, 4H), 7.99 – 8.09 (m, 8H); ^{13}C NMR (100.53 MHz, $CDCl_3$): δ 27.9, 29.9, 40.0, 63.7, 66.0, 66.2, 67.8, 77.4(3C), 77.7, 81.2, 81.5, 81.7, 82.0(2C), 82.1, 104.9, 105.8, 106.0, 117.4, 128.4(2C), 128.4(2C), 128.5(2C), 128.6(4C), 128.6(2C), 129.0, 129.1(2C), 129.2, 129.2, 129.3, 129.8(2C), 129.9(6C), 129.9(2C), 130.0(2C), 133.3, 133.3, 133.5(2C), 133.7, 133.8, 165.1, 165.2, 165.5, 165.7(2C), 165.8, 172.5, 206.3; IR ($CHCl_3$): 3462, 3051, 2939, 1767, 1599, 1451, 1234, 1110, 697 cm^{-1}; HRMS (TOF) m/z [M + Na]$^+$ calcd for $C_{65}H_{60}O_{21}Na$, 1199.3524, found 1199.3525.

Allyl 2-*O*-benzoyl-3,4,6-tri-*O*-benzyl-α-D-mannopyranoside (**17**): Yield: (86%); $[\alpha]_D^{25}$ = -8.3 (*c* = 1.0, $CHCl_3$); ^{1}H NMR (399.78MHz, $CDCl_3$): δ 3.75 – 3.82 (m, 1H), 3.86 – 3.93 (m, 2H), 4.02 (ddt, *J* = 12.8, 6.0, 1.3 Hz, 1H), 4.06 – 4.16 (m, 2H), 4.20 (ddt, *J* = 12.8, 5.1, 1.5 Hz,

1H), 4.51 – 4.60 (m, 3H), 4.74 (d, *J* = 12.0 Hz, 1H), 4.80 (d, *J* = 11.2 Hz, 1H), 4.87 (d, *J* = 10.7 Hz, 1H), 5.02 (d, *J* = 1.9 Hz, 1H), 5.20 (dq, *J* = 10.6, 1.3 Hz, 1H), 5.28 (dq, *J* = 17.0, 1.6 Hz, 1H), 5.64 (dd, *J* = 2.8, 2.1 Hz, 1H), 5.84 – 5.95 (m, 1H), 7.17 – 7.20 (m, 2H), 7.22 – 7.25 (m, 3H), 7.26 – 7.27 (m, 2H), 7.28 – 7.29 (m, 1H), 7.31 (dq, *J* = 3.3, 1.4 Hz, 3H), 7.32 – 7.34 (m, 1H), 7.34 – 7.40 (m, 5H), 7.55 (ddt, *J* = 8.7, 7.0, 1.3 Hz, 1H), 8.04 – 8.10 (m, 2H); ^{13}C NMR (100.53 MHz, $CDCl_3$): δ 68.4, 69.2(2C), 71.7, 71.8, 73.6, 74.5, 75.5, 78.4(2C), 97.1, 118.0, 127.7(3C), 127.7, 127.8, 128.2(3C), 128.4(2C), 128.5(3C), 128.5, 128.5, 130.0, 130.1(3C), 133.3, 133.6, 138.2, 138.5, 138.6, 165.9; IR ($CHCl_3$): 3460, 3030, 2920, 1600, 1453, 1203, 1058, 696 cm^{-1}; HRMS (TOF) m/z [M + Na]$^+$ calcd for $C_{37}H_{38}O_7Na$, 617.2515, found 617.2509.

Allyl 3,4,6-tri-O-benzyl-α-D-mannopyranoside (**18**): Yield: (90%); $[\alpha]_D^{25}$ = 57.0 (*c* = 1.0, $CHCl_3$); ^{1}H NMR (399.78MHz, $CDCl_3$): δ 2.54 (s, 1H), 3.67 – 3.82 (m, 3H), 3.82 – 3.93 (m, 2H), 3.98 (dd, *J* = 12.9, 6.1 Hz, 1H), 4.05 (s, 1H), 4.17 (dd , *J* = 12.9, 5.1 Hz, 1H), 4.51 (t, *J* = 11.8 Hz, 2H), 4.62 – 4.74 (m, 3H), 4.82 (d, *J* = 10.8 Hz, 1H), 4.95 (d, *J* = 1.3 Hz, 1H), 5.14 – 5.20 (m, 1H), 5.25 (dd, *J* = 17.2, 1.5 Hz, 1H), 5.87 (ddt, *J* = 16.6, 10.6, 5.6 Hz, 1H), 7.17 (dd, *J* = 5.0, 2.6 Hz, 2H), 7.31 (ddd, *J* = 16.4, 12.1, 6.9 Hz, 13H); ^{13}C NMR (100.53 MHz, $CDCl_3$): δ 68.1, 68.5, 69.02, 71.2, 72.1, 73.5, 74.4, 75.3, 80.3(2C), 98.5, 117.6, 127.7, 127.8, 127.9(2C), 128.0(2C), 128.0, 128.0, 128.4(2C), 128.5(2C), 128.6(2C), 133.8, 138.0, 138.3, 138.4; IR ($CHCl_3$): 3460, 3030, 2920, 1600, 1453, 1203, 1058, 696 cm^{-1}; HRMS (TOF) m/z [M + Na]$^+$ calcd for $C_{30}H_{34}O_6Na$, 513.2253, found 513.2261.

Allyl 2-*O*-(2-*O*-benzoyl-3,4,6-tri-*O*-benzyl-α-D-mannopyranosyl)-3,4,6-tri-O-benzyl-α-D-mannopyranoside (**20**): Yield: (93%); $[\alpha]_D^{25}$ = 2.8 (*c* = 1.0, $CHCl_3$); ^{1}H NMR (399.78 MHz, $CDCl_3$): δ 3.69 – 3.80 (m, 4H), 3.87 (dd, *J* = 11.4, 7.0 Hz, 3H), 3.96 (dd, *J* = 9.2, 2.1 Hz, 1H), 4.00 – 4.06 (m, 3 H), 4.11 (dt, *J* = 8.4, 5.3 Hz, 2H), 4.42 – 4.51 (m, 2H), 4.55 (dd, *J* = 11.7, 6.1 Hz, 3H), 4.64 – 4.79 (m, 5H), 4.86 (dd, *J* = 10.8, 5.9 Hz, 2H), 4.95 (s, 1H), 5.13 (d, *J* = 10.4 Hz, 1H), 5.20 (t, *J* = 8.6 Hz, 2H), 5.78 (m, 1H), 5.80 – 5.91 (m, 1H), 7.14 – 7.22 (m, 11H), 7.23 – 7.29 (m, 12H), 7.35 (dq, *J* = 12.4, 7.1, 6.6 Hz, 9H), 7.55 (t, *J* = 7.4 Hz, 1H), 8.07 (d, *J* = 8.1 Hz, 2H); ^{13}C NMR (100.53 MHz, $CDCl_3$): δ 68.0, 69.2, 69.3, 69.4, 71.8, 72.0, 72.1,

72.3, 73.4, 73.5, 74.5, 74.8, 75.2, 75.3, 75.3, 78.3, 79.9(2C), 98.0, 99.7, 117.3, 127.5, 127.6, 127.6(7C), 127.7, 128.0(2C), 128.2(2C), 128.2(2C), 128.3(2C), 128.4(2C), 128.4(4C), 128.4(4C), 128.5(2C), 130.1(3C), 130.1, 133.1, 133.9, 138.2, 138.4, 138.5(2C), 138.6, 138.6, 165.5; IR ($CHCl_3$): 3455, 3041, 2931, 1599, 1453, 1211, 1058, 697 cm^{-1}; HRMS (TOF) m/z $[M + Na]^+$ calcd for $C_{64}H_{66}O_{12}Na$, 1049.4452, found 1049.4449.

Allyl 2,3-di-*O*-benzyl-5-*O*-^{t}butyldiphenylsilyl-α-D-arabinofuranoside (**21**): Yield: (81% over two steps); $[\alpha]_D^{25}$ = 37.9 (*c* = 1.0, $CHCl_3$); ^{1}H NMR (399.78 MHz, $CDCl_3$): δ 0.97 (s, 9H), 3.71 – 3.77 (m, 2H), 3.90 – 3.95 (m, 1H), 3.95 – 3.97 (m, 1H), 3.99 (dd, *J* = 2.9, 1.3 Hz, 1H), 4.08 – 4.11 (m, 1H), 4.12 – 4.18 (m, 1H), 4.38 – 4.51 (m, 4H), 5.02 (d, *J* = 1.0 Hz, 1H), 5.11 (dq, *J* = 10.3, 1.3 Hz, 1H), 5.23 (dq, *J* = 17.3, 1.7 Hz, 1H), 5.79 – 5.92 (m, 1H), 7.15 – 7.22 (m, 6H), 7.23 – 7.29 (m, 8H), 7.30 – 7.36 (m, 2H), 7.60 (td, *J* = 7.4, 6.8, 1.4 Hz, 4H); ^{13}C NMR (100.53 MHz, $CDCl_3$): δ 19.5, 27.0(3C), 64.0, 68.3, 72.0, 72.2, 82.5, 83.4(2C), 88.5, 105.4, 117.4, 127.7(3C), 127.8(3C), 127.9(2C), 127.9(2C), 128.5(2C), 128.5(2C), 129.8(2C), 133.6, 134.5, 135.8(2C), 135.8(2C), 137.8, 138.1; IR ($CHCl_3$): 3034, 2928, 1458, 1230, 1103, 698 cm^{-1}; HRMS (TOF) m/z $[M + Na]^+$ calcd for $C_{38}H_{44}O_5NaSi$, 631.2855, found 631.2855.

Allyl 2,3-di-*O*-benzyl-5-*O*-(naphthalen-1-yl methyl)-α-D-arabinofuranoside (**22**): Yield: (81% over two steps); $[\alpha]_D^{25}$ = 43.4 (*c* = 1.0, $CHCl_3$); ^{1}H NMR (399.78 MHz, $CDCl_3$): δ 3.65 (qd, *J* = 10.7, 4.5 Hz, 2H), 3.94 (dd, *J* = 6.7, 3.1 Hz, 1H), 3.97 – 4.04 (m, 1H), 4.06 (dd, *J* = 3.1, 1.2 Hz, 1H), 4.21 – 4.28 (m, 2H), 4.47 (dd, *J* = 11.9, 3.3 Hz, 2H), 4.55 (d, *J* = 11.7 Hz, 2H), 4.67 – 4.78 (m, 2H), 5.11 (s, 1H), 5.16 – 5.21 (m, 1H), 5.30 (dq, *J* = 17.2, 1.6 Hz, 1H), 5.92 (dddd, *J* = 16.9, 10.9, 6.2, 5.0 Hz, 1H), 7.18 – 7.25 (m, 5H), 7.27 – 7.34 (m, 5H), 7.41 – 7.50 (m, 3H), 7.74 – 7.84 (m, 4H); ^{13}C NMR (100.53 MHz, $CDCl_3$): δ 68.2, 69.9, 72.1, 72.3, 73.6, 80.9, 83.7(2C), 88.5, 105.5, 117.4, 126.0(2C), 126.2, 126.7, 127.8(2C), 127.9(2C), 128.0, 128.0(2C), 128.3, 128.4(2C), 128.5(2C), 133.1, 133.4, 134.4, 135.7, 137.7, 138.0; IR ($CHCl_3$): 3029, 2917, 1455, 1213, 698 cm^{-1}; HRMS (TOF) m/z $[M + Na]^+$ calcd for $C_{33}H_{34}O_5Na$, 533.2303, found 533.2306.

Allyl 3-*O*-benzyl-5-*O*-^{t}butyldiphenylsilyl-β-D-arabinofuranoside (**26**): Yield: (45%); $[\alpha]_D^{25}$ = -23.8 (*c* = 1.0, $CHCl_3$); ^{1}H NMR (399.78 MHz, $CDCl_3$): δ 1.06 (s, 9H), 2.63 (d, *J* = 9.5 Hz, 1H), 3.75 (m, 2H), 3.98 (q, *J* = 6.2 Hz, 2H), 4.08 (q, *J* = 5.5 Hz, 1H), 4.18 (dd, *J* = 12.8, 5.2 Hz, 1H), 4.27 (dt, *J* = 9.8, 5.3 Hz, 1H), 4.62 (d, *J* = 11.8 Hz, 1H), 4 .76 (d, *J* = 11.8 Hz, 1H), 5.01 (d, *J* = 4.7 Hz, 1H), 5.13 (d, *J* = 10.4 Hz, 1H), 5.18 (d, *J* = 17.2 Hz, 1H), 5.80 (ddt, *J* = 16.4, 10.6, 5.7 Hz, 1H), 7.28 (dd, *J* = 14.5, 4.1 Hz, 5H), 7.34 – 7.44 (m, 6H), 7.68 (d, *J* = 7.0 Hz, 4H); ^{13}C NMR (100.53 MHz, $CDCl_3$): δ 19.4, 27.0(3C), 65.5, 68.8, 72.0, 78.3, 82.6, 84.7(2C), 100.7, 117.7, 127.7, 127.7(2C), 127.8(2C), 128.5, 129.8, 129.8, 133.4, 133.5, 133.8, 135.7(6C), 138.2; IR ($CHCl_3$): 3451, 3030, 2929, 1455, 1213, 697 cm^{-1}; HRMS (TOF) m/z $[M + Na]^+$ calcd for $C_{31}H_{38}O_5NaSi$, 541.2386, found 541.2390.

Allyl 2-O-(2,3-di-*O*-benzyl-5-*O*-(naphthalen-1-yl methyl)-β-D-arabinofuranosyl)-3-*O*-benzyl-5-*O*-^{t}butyldiphenylsilyl-β-D-arabinofuranoside (**27**): Yield: (92%) $[\alpha]_D^{25}$ = -69.7 (*c* = 1.0, $CHCl_3$); ^{1}H NMR (399.78 MHz, $CDCl_3$): δ 1.04 (s, 9H) , 3.58 (dd, *J* = 9.7, 4.5 Hz, 1H), 3.65 (td, *J* = 8.3, 7.0, 2.5 Hz, 1H), 3.71 – 3.76 (m, 2H), 3.90 (dd, *J* = 12.1, 6.0 Hz, 1H), 4.06 – 4.22 (m, 5H), 4.27 (td, *J* = 7.1, 6.2, 3.1 Hz, 1H), 4.41 (dd, *J* = 12.3, 2.2 Hz, 1H), 4.44 – 4.51 (m, 2H), 4.53 – 4.63 (m, 4H), 4.70 (dd, *J* = 11.9, 2.4 Hz, 1H), 4.80 (dd, *J* = 11.4, 2.2 Hz, 1H), 5.05 (d, *J* = 10.3 Hz, 1H), 5.12 – 5.19 (m, 3H), 5.69 – 5.81 (m, 1H), 7.23 (s, 10H), 7.29 – 7.38 (m, 11H), 7.39 (d, *J* = 2.0 Hz, 1H), 7.41 – 7.45 (m, 2H), 7.59 (s, 1H), 7.63 – 7.69 (m, 4H), 7.71 – 7.80 (m, 3H); 13C NMR (100.53 MHz, CDCl3): δ 19.4, 27.0(3C), 66.0, 68.2, 71.8, 72.3, 72.4, 72.8, 73.3, 79.5, 81.0(2C), 82.3, 82.9, 83.2, 84.0, 98.7, 99.0, 117.7, 125.8, 125.8, 126.1, 126.4, 127.6, 127.7(2C), 127.7, 127.8, 127.8(4C), 128.0, 128.2(2C), 128.4(2C), 128.4(2C), 128.5(2C), 129.8(2C), 133.1, 133.4, 133.5(2C), 134.0(2C), 135.7(6C), 135.8, 138.1, 138.3, 138.4; IR ($CHCl_3$): 3032, 2931, 1458, 1230, 1103, 698 cm^{-1}; HRMS (TOF) m/z $[M + Na]^+$ calcd for $C_{61}H_{66}O_9NaSi$, 993.4374, found 993.4379.

Allyl 2-O-benzoyl-α-D-arabinofuranoside (**4**): Yield: (90%); $[\alpha]_D^{25}$ = 99.3 (*c* = 1.0, $CHCl_3$); ^{1}H NMR (399.78 MHz, $CDCl_3$): δ 2.24 (s, 1H), 3.51 (s, 1H), 3.80 (d, *J* = 11.8 Hz, 1H), 3.90 – 3.98 (m, 1H), 4.08 (ddt, *J* = 12.9, 6.2, 1.3 Hz, 1H), 4.22 (dd, *J* = 6.1, 2.5 Hz, 2H), 4.29 (ddt, *J* = 12.9, 5.1, 1.4 Hz, 1H), 5.1 3 (d, *J* = 1.5 Hz, 1H), 5.23 (dd, *J* = 10.4, 1.3 Hz, 1H), 5.30 (s,

1H), 5.34 (dq, *J* = 17.2, 1.6 Hz, 1H), 5.87 – 6.01 (m, 1H), 7.44 (t, *J* = 7.7 Hz, 2H), 7.59 (t, *J* = 7.4 Hz, 1H), 7.99 – 8.03 (m, 2H); ^{13}C NMR (100.53 MHz, $CDCl_3$): δ 62.0, 68.4, 76.6, 84.2(2C), 86.3, 104.6, 118.0, 128.7, 129.1, 129.9(2C), 133.7, 133.8, 166.8; IR ($CHCl_3$): 3427, 3074, 2929, 1601, 1451, 1203, 1105, 712 cm^{-1}; HRMS (TOF) m/z $[M + Na]^+$ calcd for $C_{15}H_{18}O_6Na$, 317.1007, found 317.1006.

Allyl 2-*O*-benzoyl-5-*O*-^{t}butyldiphenylsilyl-α-D-arabinofuranoside (**31**): Yield: (30%); $[\alpha]_D^{25}$ = 47.5 (*c* = 1.0, $CHCl_3$); ^{1}H NMR (399.78 MHz, $CDCl_3$): δ 1.04 (s, 9H), 3.09 (d, *J* = 4.1 Hz, 1H), 3.89 (d, *J* = 4.2 Hz, 2H), 4.07 (dd, *J* = 12.9, 6.2 Hz, 1H), 4.21 – 4.31 (m, 3H), 5.14 (s, 1H), 5.22 (dd, *J* = 10.4, 1.4 Hz, 1H), 5.28 (s, 1H), 5.33 (dt, *J* = 17.2, 1.5 Hz, 1H), 5.94 (dddd, J = 16.7, 10.5, 6.1, 5.2 Hz, 1H), 7.31 – 7.44 (m, 8H), 7.56 (d, *J* = 7.4 Hz, 1H), 7.68 (dd, *J* = 7.9, 1.4 Hz, 4H), 7.91 – 7.98 (m, 2H); ^{13}C NMR (100.53 MHz, $CDCl_3$): δ 19.4, 26.9(3C), 63.7, 68.3, 77.0, 84.8(2C), 85.8, 104.6, 117.9, 127.8(2C), 127.8(2C), 128.6(2C), 129.3, 129.8(2C), 129.9(2C), 133.4, 133.6, 133.9, 135.7(2C), 135.7(2C), 166.7; IR ($CHCl_3$): 3435, 3027, 2924, 1599, 1454, 1213, 1102, 700 cm^{-1}; HRMS (TOF) m/z $[M + Na]^+$ calcd for $C_{31}H_{36}O_6NaSi$, 555.2178, found 555.2180.

Allyl 2-*O*-benzoyl- 3-*O*-(2,3-di-*O*-benzoyl-5-*O*-(2,3-di-*O*-benzoyl-5-*O*-(2,3-di-*O*-benzoyl-5-*O*-(4-oxopentanoyl)-α-D-arabinofuranosyl)-α-D-arabinofuranosyl)-α-D-arabinofuranosyl)-5-O-tbutyldiphenylsilyl-α-D-arabinofuranoside (**32**): Yield: (95%); $[\alpha]_D^{25}$ = 13.7 (*c* = 1.0, $CHCl_3$); ^{1}H NMR (399.78 MHz, $CDCl_3$): δ 0.99 (s, 9H), 2.11 (s, 3H), 2.58 (td, *J* = 7.2, 6.6, 2.2 Hz, 2H), 2.67 – 2.73 (m, 2H), 3.69 (d, *J* = 10.9 Hz, 1H), 3.89 (d, *J* = 3.7 Hz, 2H), 3.94 (d, *J* = 10.3 Hz, 1H), 4.11 (td, *J* = 10.9, 9.4, 4.6 Hz, 2H), 4.19 (dd, *J* = 11.9, 3.1 Hz, 2H), 4.25 – 4.34 (m, 2H), 4.39 (dd, *J* = 11.2, 4.5 Hz, 1H), 4.53 – 4.62 (m, 4H), 5.18 (d, *J* = 10.4 Hz, 1H), 5.25 (s, 1H), 5.35 (s, 1H), 5.38 – 5.41 (m, 2H), 5.42 (s, 1H), 5.44 (s, 1H), 5.57 – 5.64 (m, 5H), 5.69 (d, *J* = 4.3 Hz, 1H), 5.95 (ddt, *J* = 16.7, 10.7, 5.4 Hz, 1H), 7.20 – 7.24 (m, 2H), 7.25 – 7.32 (m , 6H), 7.36 (q, *J* = 7.3 Hz, 10H), 7.43 (d, *J* = 7.4 Hz, 2H), 7.45 – 7.51 (m, 4H), 7.52 – 7.60 (m, 3H), 7.62 – 7.66 (m, 4H), 7.83 (d, *J* = 8.2 Hz, 2H), 7.89 (d, *J* = 8.2 Hz, 2H), 8.01 (ddd, *J* = 26.8, 16.6, 8.0 Hz, 10H); ^{13}C NMR (100.53 MHz, $CDCl_3$): δ 19.4 , 26.8(3C), 27.9, 29.9, 38.0, 63.2, 63.7, 65.5, 66.1, 67.8(2C), 77.0, 77.3, 77.7, 80.1, 81.2,

81.5(2C), 81.6, 82.1, 82.9, 82.9, 83.5, 105.0, 105.2, 106.0, 106.0, 117.3, 127.7(2C), 127.8(2C), 128.3(2C), 128.4(2C), 128.4(2C), 128.6(6C), 128.6(2C), 129.0, 129.1, 129.1, 129.2, 129.2, 129.4, 129.5, 129.7, 129.8(2C), 129.8, 129.9(5C), 123.0(8C), 133.2 (2C), 133.3(2C), 133.4 (2C), 133.7, 134.2, 135.6(2C), 135.7(2C), 165.1, 165.1, 165.2, 165.6, 165.6, 165.7, 165.7, 172.5, 206.3; IR ($CHCl_3$): 3419, 3035, 2920, 1752,1601, 1455, 1210, 1100, 693 cm^{-1}; HRMS (TOF) m/z $[M + Na]^+$ calcd for $C_{93}H_{90}O_{26}NaSi$, 1674.5420, found 1674.5425.

Allyl 2-*O*-benzoyl- 3-*O*-(2,3-di-*O*-benzoyl-5-*O*-(2,3-di-*O*-benzoyl-5-*O*-(2,3-di-*O*-benzoyl-5-*O*-(4-oxopentanoyl)-α-D-arabinofuranosyl)-α-D-arabinofuranosyl)-α-D-arabinofuranosyl)-α-D-arabinofuranoside (**33**): Yield: (90%); $[\alpha]_D^{25}$ = 12.3 (*c* = 1.0, $CHCl_3$); ^{1}H NMR (399.78 MHz, $CDCl_3$): δ 2.12 (s, 3H), 2.58 (td, *J* = 7.5, 6.8, 2.6 Hz, 2H), 2.67 – 2.72 (m, 2H), 3.82 (d, *J* = 11.4 Hz, 1H), 3.90 – 3.99 (m, 3H), 4.06 – 4.21 (m, 3H), 4.23 – 4.33 (m, 2H), 4.35 – 4.47 (m, 3H), 4.53 – 4.62 (m, 3H), 5.15 – 5.20 (m, 1H), 5.23 (s, 1H), 5.33 (q, *J* = 1.6 Hz, 1H), 5.36 (s, 1H), 5.38 – 5.41 (m, 2H), 5.43 (s, 1H), 5.56 (d, *J* = 4.8 Hz, 1H), 5.59 (s, 3H), 5.63 (d, *J* = 3.6 Hz, 2H), 5.92 (dddd, *J* = 16.7, 10.6, 6.0, 4.9 Hz, 1H), 7.2 7 (q, *J* = 8.2 Hz, 4H), 7.40 (tdd, *J* = 7.4, 6.2, 5.5, 2.8 Hz, 9H), 7.43 – 7.48 (m, 4H), 7.49 – 7.55 (m, 2H), 7.55 – 7.61 (m, 2H), 7.91 (ddd, *J* = 8.4, 3.6, 1.2 Hz, 4H), 7.98 – 8.08 (m, 10H); ^{13}C NMR (100.53 MHz, $CDCl_3$): δ 27.9, 29.9, 38.0, 62.0, 63.7, 66.1, 66.4, 67.9, 77.2, 77.3, 77.7, 80.8, 81.2, 81.5, 81.5, 81.9, 82.0, 82.5, 83.1, 83.3(2C), 105.0, 105.6, 105.8, 106.0, 117.5, 128.4(5C), 128.6 (8C), 128.6, 129.0, 129.1(2C), 129.1, 129.2, 129.3, 129.4, 129.9(8C), 130.0(5C), 133.3, 133.4, 133.5(4C), 133.7, 133.9, 165.2, 165.3, 165.4, 165.6, 165.7, 165.7, 165.7, 172.6, 206.4; IR ($CHCl_3$): 3426, 3030, 2929, 1741,1599, 1455, 1210, 1100, 693 cm^{-1}; HRMS (TOF) m/z $[M + Na]^+$ calcd for $C_{77}H_{72}O_{26}Na$, 1435.4209, found 1435.4193.

Allyl 2-O-benzoyl- 3-O-(2,3-di-O-benzoyl-5-O-(2,3-di-O-benzoyl-5-O-(2,3-di-O-benzoyl-5-O-(4-oxopentanoyl)-α-D-arabinofuranosyl)-α-D-arabinofuranosyl)-α-D-arabinofuranosyl)-5-*O*-(2,3-di-*O*-benzoyl-5-*O*-(2,3-di-*O*-benzoyl-5-*O*-(2,3-di-*O*-benzoyl-5-*O*-(^{t}butyldiphenylsilyl)-α-D-arabinofuranosyl)-α-D-arabinofuranosyl)-α-D-arabinofuranosyl)-α-D-arabinofuranoside (**34**): Yield: (92%); $[\alpha]_D^{25}$ = 13.9 (*c* = 1.0, $CHCl_3$);

^{1}H NMR (399.78 MHz, $CDCl_3$): δ 1.01 (s, 9H), 2.10 (s, 3H), 2.57 (dd, *J* = 6.3, 1.9 Hz, 2H), 2.65 – 2.70 (m, 2H), 3.79 – 3.92 (m, 5H), 3.95 – 3.99 (m, 2H), 4.00 – 4.09 (m, 2H), 4.14 – 4.28 (m, 5H), 4.35 – 4.44 (m, 3H), 4.46 – 4.54 (m, 3H), 4.59 (ddd, *J* = 11.8, 7.7, 3.6 Hz, 4H), 5.14 (dd, *J* = 10.5, 1.5 Hz, 1H), 5.23 (s, 1H), 5.29 – 5.34 (m, 3H), 5.36 (s, 1H), 5.38 (d, *J* = 5.2 Hz, 2H), 5.41 (s, 1H), 5.45 (d, *J* = 1.5 Hz, 1H), 5.52 (s, 1H), 5.56 (d, *J* = 1.3 Hz, 1H), 5.58 (d, *J* = 1.2 Hz, 2H), 5.60 (d, *J* = 1.1 Hz, 1H), 5.62 (d, *J* = 3.5 Hz, 5H), 5.64 (d, *J* = 5.0 Hz, 1H), 5.69 (d, *J* = 4.6 Hz, 1H), 5.84 – 5.95 (m, 1H), 7.22 – 7.30 (m, 11H), 7.30 – 7.35 (m, 10H), 7.36 – 7.39 (m, 10H), 7.40 – 7.43 (m, 3H), 7.43 – 7.48 (m, 6H), 7.48 – 7.52 (m, 2H), 7.55 (d, *J* = 11.7 Hz, 2H), 7.69 (ddd, *J* = 7.9, 4.1, 1.6 Hz, 4H), 7.84 (dd, *J* = 8.3, 1.2 Hz, 2H), 7.86 – 7.87 (m, 2H), 7.87 – 7.89 (m, 3H), 7.91 (dd, *J* = 6.4, 1.2 Hz, 2H), 7.94 (dd, *J* = 2.7, 1.3 Hz, 2H), 7.96 – 7.97 (m, 5H), 7.98 – 7.99 (m, 5H), 8.00 – 8.06 (m, 6H); ^{13}C NMR (100.53 MHz, $CDCl_3$):) δ 19.4, 26.8(3C), 27.9, 29.8, 37.9, 63.5, 63.7, 65.6, 65.7, 65.8, 66.0, 67.8(2C), 77.0, 77.2, 77.3, 77.3, 77.4(2C), 77.7(2C), 80.7, 81.2, 81.5(3C), 81.6(2C), 82.0, 82.2, 82.2, 82.6, 82.8, 82.9, 83.2, 105.1, 105.4, 105.8, 105.9, 105.9, 106.0, 106.0, 117.4, 127.7(7C), 128.3(2C), 128.3(2C), 128.3(2C), 128.4(2C), 128.4(3C), 128.5(3C), 128.5(3C), 128.6(2C), 129.0, 129.1, 129.1, 129.1(2C), 129.1, 129.2, 129.2, 129.3(2C), 129.3, 129.4, 129.7(2C), 129.8(2C), 129.8(9C), 129.9(12C), 129.9(5C), 130.0(2C), 133.1, 133.2, 133.2, 133.3, 133.3(5C), 133.4(3C), 133.6, 133.9, 135.7(3C), 135.7(3C), 165.1, 165.1, 165.1, 165.2, 165.2, 165.2, 165.5, 165.6, 165.6(4C), 165.7, 172.5, 206.3; IR ($CHCl_3$): 3016, 2918, 1750, 1600, 1454, 1267, 1106, 708 cm^{-1}; HRMS (TOF) m/z $[M + Na]^+$ calcd for $C_{150}H_{138}O_{44}NaSi$, 2694.8261, found 2694.7383.

Allyl 2-*O*-benzoyl-3-*O*-benzyl-β-D-arabinofuranoside (**6**): Yield: (87% over two steps); $[\alpha]_D^{25}$ = -115.7 (*c* = 1.0, $CHCl_3$); ^{1}H NMR (399.78 MHz, $CDCl_3$):) δ 2.49 (s, 1H), 3.65 (ddd, *J* = 12.1, 8.2, 4.1 Hz, 1H), 3.79 (dt, *J* = 11.8, 2.5 Hz, 1H), 4.00 (ddt, *J* = 12.9, 5.8, 1.3 Hz, 1H), 4.15 – 4.21 (m, 2H), 4.58 – 4.7 3 (m, 3H), 5.09 (dq, *J* = 10.7, 1.2 Hz, 1H), 5.18 (dq, *J* = 17.1, 1.5 Hz, 1H), 5.23 (dd, *J* = 6.8, 4.9 Hz, 1H), 5.42 (d, *J* = 4.7 Hz, 1H), 5.68 – 5.82 (m, 1H), 7.24 – 7.35 (m, 5H), 7.43 – 7.49 (m, 2H), 7.59 (tt, *J* = 7.0, 1.3 Hz, 1H), 8.02 – 8.08 (m, 2H); ^{13}C NMR (100.53 MHz, $CDCl_3$): δ 63.5, 69.8, 72.6, 79.7, 79.8, 82.2(2C), 100.0, 117.9, 127.8, 128.0, 128.6(3C), 129.4, 129.9(3C), 133.5(2C), 137.6, 166.0; IR ($CHCl_3$): 3463, 3041, 2929,

1599, 1453, 1276, 1108, 704 cm^{-1}; HRMS (TOF) m/z [M + Na]$^+$ calcd for $C_{22}H_{24}O_6Na$, 407.1470, found 407.1465.

Allyl 2-*O*-benzoyl-3-*O*-benzyl-5-*O*-(2-*O*-(2,3-di-*O*-benzyl-5-*O*-(naphthalen-1-yl methyl)-β-D-arabinofuranosyl)-3-*O*-benzyl-5-*O*-^{t}butyldiphenylsilyl-α-D-arabinofuranosyl)-β-D-arabinofuranoside (**35**): Yield: (81%); $[\alpha]_D^{25}$ = -0.3 (*c* = 1.0, $CHCl_3$); ^{1}H NMR (399.78 MHz, $CDCl_3$): δ 1.06 (s, 9H), 3.55 – 3.63 (m, 3H), 3.77 – 3.86 (m, 2H), 3.93 (d, *J* = 15.1 Hz, 2H), 4.07 – 4.29 (m, 7H), 4.40 – 4.51 (m, 4H), 4.52 (s, 1H), 4.57 (d, *J* = 3.2 Hz, 2H), 4.60 – 4.69 (m, 5H), 4.97 – 5.05 (m, 2H), 5.10 (d, *J* = 4.3 Hz, 2H), 5.22 (d, *J* = 4.6 Hz, 1H), 5.40 (d, *J* = 4.6 Hz, 1H), 5.72 (ddd, *J* = 14.5, 10.5, 4.5 Hz, 1H), 7.18 – 7.22 (m, 6H), 7.24 (dd, *J* = 5.7, 3.3 Hz, 7H), 7.27 – 7.37 (m, 12H), 7.37 (s, 2H), 7.43 (t, *J* = 3.5 Hz, 4H), 7.53 – 7.62 (m, 2H), 7.65 – 7.73 (m, 5H), 7.74 – 7.81 (m, 2H), 8.04 (dd, *J* = 6.4, 2.1 Hz, 2H); ^{13}C NMR (100.53 MHz, $CDCl_3$): δ 19.4, 27.0(3C), 64.0, 68.5, 68.9, 72.3, 72.3(2C), 72.4, 72.6, 73.3, 79.7, 79.8, 80.2, 82.3, 82.8, 83.2(2C), 83.9, 84.1, 85.8, 99.6, 100.2, 106.0, 117.1, 125.7, 125.9, 126.1, 126.4, 127.6(3C), 127.8(10C), 127.9, 128.0, 128.1(2C), 128.2, 128.4(2C), 128.4(2C), 128.5(2C), 128.6(2C), 129.6, 129.8(2C), 129.9(2C), 133.0, 133.3, 133.3, 133.5, 133.5, 133.9, 135.5, 135.7(6C), 137.8, 137.9, 138.1, 138.2, 166.0; IR ($CHCl_3$): 3057, 2927, 1598, 1456, 1268, 1107, 698 cm^{-1}; HRMS (TOF) m/z [M + Na]$^+$ calcd for $C_{80}H_{84}O_{14}NaSi$, 1319.5527, found 1319.5518.

Allyl 2-*O*-benzoyl-3-*O*-benzyl-5-*O*-(2-*O*-(2,3-di-*O*-benzyl-5-*O*-(2-*O*-(2-*O*-benzoyl-3,4,6-tri-*O*-benzyl-α-D-mannopyranosyl)-3,4,6-tri-*O*-benzyl-α-D-mannopyranosyl)-β-D-arabinofuranosyl)-3-*O*-benzyl-5-*O*-^{t}butyldiphenylsilyl-α-D-arabinofuranosyl)-β-D-arabinofuranoside (**37**): Yield: (76%); $[\alpha]_D^{25}$ = -37.9 (*c* = 1.0, $CHCl_3$); ^{1}H NMR (399.78MHz, $CDCl_3$): δ 1.03 (s, 9H), 3.53 – 3.66 (m, 4H), 3.70 – 3.87 (m, 8H), 3.88 – 4.01 (m, 4H), 4.02 – 4.13 (m, 6H), 4.14 – 4.25 (m, 3H), 4.39 (dd, *J* = 9.8, 3.2 Hz, 4H), 4.44 – 4.55 (m, 7H), 4.56 – 4.64 (m, 6H), 4.64 – 4.75 (m, 3H), 4.83 (dd, *J* = 10.9, 3.9 Hz, 2H), 4.96 (dd, *J* = 18.4, 1.6 Hz, 2H), 5.00 – 5.09 (m, 2H), 5.11 – 5.23 (m, 3H), 5.37 (d, *J* = 4.5 Hz, 1H), 5.64 – 5.74 (m, 1H), 5.76 (s, 1H), 7.13 – 7.22 (m, 17H), 7.25 (dtd, *J* = 8.6, 5.0, 4.4, 1.9 Hz, 30H), 7.33 – 7.37 (m, 10H), 7.42 (t, *J* = 7.7 Hz, 3H), 7.50 – 7.58 (m, 2H), 7 .66 (td, *J* = 8.1, 1.5 Hz, 4H), 7.98 – 8.08

(m, 4H); ^{13}C NMR (100.53 MHz, $CDCl_3$): δ 19.4, 27.0(3C), 64.0, 68.6, 69.1(3C), 71.7, 72.2(3C), 72.2, 72.4, 72.4, 72.7, 73.4(4C), 74.3, 74.4, 75.2, 75.2, 75.3, 78.3, 79.3, 79.7, 79.8, 78.0, 82.2, 82.6, 83.7, 83.9(3C), 85.9, 98.6, 99.6, 99.7, 100.4, 105.8, 117.0, 127.4(3C), 127.4, 127.5(5C), 127.5(3C), 127.6, 127.8(6C), 128.0(3C), 128.1(3C), 128.1(3C), 128.2(3C), 128.3(4C), 128.3(3C), 128.4(4C), 128.4(3C), 128.4, 128.4, 128.5(5C), 128.5, 128.6, 129.6, 129.8(4C), 129.9(3C), 130.1(3C), 130.1, 133.1, 133.3, 133.5, 133.5, 133.9, 135.7, 135.8, 137.7, 138.0, 138.0, 138.0, 138.2, 138.3, 138.6, 138.6, 138.6, 138.6, 165.4, 166.0; IR ($CHCl_3$): 3045, 2929, 1599, 1433, 1253, 1107, 704 cm^{-1}; HRMS (TOF) m/z $[M + Na]^+$ calcd for $C_{130}H_{136}O_{25}NaSi$, 2148.9071, found 2148.9023.

Allyl 2-*O*-benzoyl-3,5-di-*O*-(2-*O*-(2,3-di-*O*-benzyl-5-*O*-(naphthalen-1-yl methyl)-β-D-arabinofuranosyl)-3-*O*-benzyl-5-*O*-^{t}butyldiphenylsilyl-α-D-arabinofuranosyl)-α-D-arabinofuranoside (**39**): Yield: (77%); $[\alpha]_D^{25}$ = -12.5 (*c* = 1.0, $CHCl_3$); ^{1}H NMR (399.78 MHz, $CDCl_3$): δ 0.94 (s, 9H), 1.00 (s, 9H), 3.50 – 3.60 (m, 4H), 3.72 (ddd, *J* = 15.6, 8.5, 3.5 Hz, 4H), 3.78 (d, *J* = 10.9 Hz, 2H), 3.96 (s, 1H), 3.99 (dd, *J* = 4.5, 1.8 Hz, 2H), 4.03 – 4.14 (m, 8H), 4.14 – 4.22 (m, 3H), 4.27 (s, 2H), 4.40 (dt, *J* = 9.2, 3.1 Hz, 4H), 4.44 (s, 1H), 4.45 – 4.50 (m, 3H), 4.52 (s, 1H), 4.54 (d, *J* = 4.1 Hz, 2H), 4.57 (d, *J* = 3.1 Hz, 1H), 4.58 – 4.60 (m, 2H), 4.62 (d, *J* = 3.5 Hz, 1H), 4.64 – 4.70 (m, 2H), 5.06 (d, *J* = 1.2 Hz, 1H), 5.13 (dd, *J* = 10.5, 1.5 Hz, 1H), 5.16 (d, *J* = 4.4 Hz, 1H), 5.19 (s, 1H), 5.26 (dd, *J* = 17.2, 1.6 Hz, 1H), 5.32 (d, *J* = 5.4 Hz, 3H), 5.86 (ddt, *J* = 16.7, 10.6, 5.6 Hz, 1H), 7.14 – 7.17 (m, 5H), 7.19 (dd, *J* = 5.9, 3.5 Hz, 6H), 7.22 (s, 10H), 7.24 – 7.26 (m, 7H), 7.26 – 7.30 (m, 11H), 7.33 (dd, *J* = 7.5, 1.9 Hz, 4H), 7.37 – 7.46 (m, 6H), 7.60 (ddd, *J* = 13.8, 7.8, 2.5 Hz, 11H), 7.65 – 7.73 (m, 4H), 7.74 – 7.79 (m, 2H), 7.92 – 8.01 (m, 2H); ^{13}C NMR (100.53 MHz, $CDCl_3$): δ 19.3, 19.4, 26.9(3C), 27.0(3C), 63.8, 63.9, 66.3, 67.8, 72.2, 72.3, 72.3, 72.4(3C), 72.6, 72.6, 73.2, 73.3, 77.4, 80.1, 80.1, 81.5, 82.1, 82.3, 82.8, 83.1, 83.5, 83.6, 83.7(2C), 83.9, 84.1, 85.1, 85.8, 99.9, 100.2, 105.1, 105.6, 106.9, 117.6, 125.7, 125.7, 125.9, 125.9, 126.1, 126.1, 126.3, 126.4, 127.5, 127.5(2C), 127.6, 127.7(3C), 127.8(17C), 127.9, 128.0(2C), 128.0(2C), 128.2(3C), 128.2(2C), 128.3(2C), 128.4(3C), 128.4(6C), 128.5(3C), 128.5(2C), 129.5, 129.7(2C), 129.9, 133.0, 133.3, 133.4, 133.5, 133.7, 134.0, 135.7(3C), 135.7(6C), 135.8(3C), 137.8, 138.0,

138.2, 138.3, 138.3, 138.4, 165.8; IR ($CHCl_3$): 3032, 2929, 1601, 1452, 1228, 1107, 695 cm^{-1}; HRMS (TOF) m/z $[M + Na]^+$ calcd for $C_{131}H_{138}O_{22}NaSi_2$, 2141.9115, found 2141.8357.

Allyl 2-*O*-benzoyl-3,5-di-*O*-(2-*O*-(2,3-di-*O*-benzyl-β-D-arabinofuranosyl)-3-*O*-benzyl-5-*O*-^{t}butyldiphenylsilyl-α-D-arabinofuranosyl)-α-D-arabinofuranoside (**40**): Yield: (75%); $[\alpha]_D^{25}$ = -7.8 (*c* = 1.0, $CHCl_3$); ^1H NMR (399.78 MHz, $CDCl_3$): δ 0.97 (s, 9H), 1.02 (s, 9H), 2.33 (d, *J* = 23.3 Hz, 2H), 3.47 – 3.61 (m, 3H), 3.67 (d, *J* = 11.4 Hz, 3H), 3.76 (d, *J* = 11.1 Hz, 3H), 3.90 (s, 1H), 3.96 – 4.03 (m, 4H), 4.06 – 4.14 (m, 3H), 4.15 – 4.21 (m, 3H), 4.22 – 4.31 (m, 4H), 4.36 – 4.43 (m, 2H), 4.44 – 4.50 (m, 3H), 4.51 – 4.65 (m, 6H), 4.67 – 4.77 (m, 3H), 5.07 (s, 1H), 5.12 – 5.19 (m, 2H), 5.20 – 5.22 (m, 1H), 5.23 – 5.36 (m, 4H), 5.88 (ddt, *J* = 15.4, 10.4, 4.9 Hz, 1H), 7.18 – 7.37 (m, 44H), 7.45 – 7.51 (m, 1H), 7.56 – 7.67 (m, 8H), 7.9 6 (d, *J* = 8.3 Hz, 2H); ^{13}C NMR (100.53 MHz, $CDCl_3$): δ 19.3, 19.4, 26.9(3C), 27.0(3C), 63.5, 63.6, 63.7, 63.8, 66.6, 67.9, 72.2, 72.4, 72.4, 72.4, 72.7, 72.7, 80.8(3C), 81.5, 81.8, 82.0, 82.1, 82.2, 82.2, 82.8, 82.8, 83.5, 84.0, 84.1, 84.9, 85.5, 99.4, 99.7, 105.1, 105.2, 106.5, 117.7, 127.6, 127.7(2C), 127.8(11C), 127.8(11C), 128.0(3C), 128.2(2C), 128.4(2C), 128.4(2C), 128.5(3C), 128.5(4C), 128.6(4C), 129.4, 129.8(3C), 129.9(2C), 133.2, 133.4, 133.4, 133.5(2C), 133.9, 135.7, 135.7, 135.8, 137.7, 137.8, 137.9, 138.0, 138.2, 138.2, 165.7; IR ($CHCl_3$): 3461, 3031, 2926, 1597, 1454, 1267, 1106, 701 cm^{-1}; HRMS (ESI) m/z $[M + Na]^+$ calcd for $C_{109}H_{122}O_{22}NaSi_2$, 1861.7864, found 1861.7887.

Allyl 2-*O*-benzoyl-3,5-di-*O*-(2-*O*-(2,3-di-*O*-benzyl-5-*O*-(2-*O*-(2-*O*-benzoyl-3,4,6-tri-*O*-benzyl-α-D-mannopyranosyl)-3,4,6-tri-*O*-benzyl-α-D-mannopyranosyl)-β-D-arabinofuranosyl)-3-*O*-benzyl-5-*O*-^{t}butyldiphenylsilyl-α-D-arabinofuranosyl)-α-D-arabinofuranoside (**41**): Yield: (60%); $[\alpha]_D^{25}$ = -14.9 (*c* = 1.0, $CHCl_3$); ^1H NMR (399.78 MHz, $CDCl_3$): δ 0.93 (s, 9H), 0.98 (s, 9H), 3.49 – 3.65 (m, 7H), 3.75 (dd, *J* = 20.5, 10.0 Hz, 14H), 3.87 (d, *J* = 9.0 Hz, 2H), 3.97 (d, *J* = 26.5 Hz, 13H), 4.08 (s, 6H), 4.16 (s, 2H), 4.22 – 4.31 (m, 3H), 4.39 (d, *J* = 12.2 Hz, 9H), 4.48 (d, *J* = 11.0 Hz, 9H), 4.59 (dd, *J* = 25.9, 12.1 Hz, 15H), 4.72 (d, *J* = 11.1 Hz, 2H), 4.80 – 4.93 (m, 6H), 5.04 – 5.12 (m, 2H), 5.13 – 5.25 (m, 4H), 5.30 (d, *J* = 15.0 Hz, 3H), 5.76 (s, 1H), 5.85 (s, 1H), 7.08 – 7.33 (m, 100H), 7.35 (d, *J* = 6.9 Hz, 6H), 7.43 (s, 2H), 7.50 – 7.67 (m, 10H), 7.92 – 8.14 (m, 7H); ^{13}C NMR (10.53 MHz, $CDCl_3$): δ

19.3, 19.4, 26.9(3C), 27.0(3C), 63.7, 63.8, 65.3, 67.8, 69.0, 69.1(3C), 69.6, 69.7, 70.3, 71.5, 71.7(2C), 72.1, 72.3(2C), 72.3(3C), 72.4, 72.5, 73.4(5C), 73.8, 74.3, 74.3, 74.5, 74.5, 74.7, 75.1, 75.2, 75.2, 75.3(2C), 75.3, 76.9, 77.4, 78.3, 78.3, 79.2, 79.4, 80.0(2C), 81.7, 82.2, 82.4, 82.8, 83.4, 83.5, 83.8(2C), 83.8, 83.9, 84.3, 85.1, 86.1, 98.6, 98.8, 9 9.7, 99.8, 100.0, 100.5, 105.1, 105.5, 106.8, 117.6, 127.4(2C), 127.4(2C), 127.5(10C), 127.6(6C), 127.6, 127.7(16C), 128.0, 128.0(6C), 128.1(3C), 128.1(4C), 128.2(2C), 128.2(4C), 128.3(10C), 128.4(17C), 128.5(10C), 128.5(5C), 128.7, 129.4, 129.7(4C), 129.9(2C), 130.1(5C), 133.1, 133.1, 133.3, 133.4, 133.5(2C), 133.6, 134.0, 135.7(3C), 135.7(6C), 135.8(3C), 137.6, 137.7, 137.9, 138.0, 138.1(2C), 138.2(2C), 138.2, 138.3, 138.3, 138.6, 138.6(2C), 138.6, 138.7, 138.7(2C), 165.4, 165.7, 166.0; IR ($CHCl_3$): 3035, 2925, 1599, 1454, 1268, 1106, 699 cm^{-1}; MALDI (TOF) m/z $[M + K]^+$ calcd for $C_{231}H_{242}O_{44}KSi_2$, 3816.59, found 3816.96.

Allyl 2-*O*-benzoyl-3-*O*-(2,3-di-*O*-benzoyl-5-*O*-(2,3-di-*O*-benzoyl-5-*O*-(2,3-di-*O*-benzoyl-5-*O*-(4-oxopentanoyl)-α-D-arabinofuranosyl)-α-D-arabinofuranosyl)-α-D-arabinofuranosyl)-5-*O*-(2,3-di-*O*-benzoyl-5-*O*-(2,3-di-*O*-benzoyl-5-*O*-(2,3-di-*O*-benzoyl-α-D-arabinofuranosyl)-α-D-arabinofuranosyl)-α-D-arabinofuranosyl)-α-*D*-arabinofuranoside (**43**): Yield: (80%); $[\alpha]_D^{25}$ = 12.2 (*c* = 1.0, $CHCl_3$); ^1H NMR (399.78 MHz, $CDCl_3$): δ 2.10 (s, 3H), 2.53 – 2.60 (m, 2H), 2.68 (t, *J* = 6.4 Hz, 2H), 3.78 – 3.94 (m, 6H), 4.03 (dq, *J* = 15.5, 5.3, 4.6 Hz, 3H), 4.13 – 4.19 (m, 4H), 4.23 (dd, *J* = 13.3, 4.8 Hz, 1H), 4.38 (dd, *J* = 9.0, 5.0 Hz, 3H), 4.43 – 4.49 (m, 3H), 4.51 – 4.60 (m, 4H), 5.11 – 5.17 (m, 1H), 5.22 (s, 1H), 5.30 (d, *J* = 6.0 Hz, 2H), 5.35 – 5.41 (m, 6H), 5.43 (s, 1H), 5.50 (s, 1H), 5.58 (d, *J* = 6.5 Hz, 3H), 5.61 (s, 6H), 5.68 (d, *J* = 4.5 Hz, 1H), 5.89 (ddt, *J* = 16.6, 10.6, 5.4 Hz, 1H), 7.18 – 7.29 (m, 8H), 7.32 – 7.50 (m, 28H), 7.53 – 7.59 (m, 2H), 7.81 – 7.93 (m, 10H), 7.95 – 8.06 (m, 17H); ^{13}C NMR (100.53 MHz, $CDCl_3$): δ 27.9, 29.9, 38.0, 62.4, 63.7, 65.6, 65.7, 66.0, 66.0, 66.1, 67.8(2C), 77.0, 77.2, 77.3, 77.4, 77.7, 77.8(2C), 80.7, 81.2, 81.5(3C), 81.6(2C), 81.8, 82.1, 82.1, 82.6, 82.8, 82.9, 83.7, 105.1, 105.4, 105.8(2C), 105.9, 105.9, 106.0, 117.4, 128.3(2C), 128.3(2C), 128.4(3C), 128.5(2C), 128.5(2C), 128.6(4C), 128.6(7C), 128.6(4C), 129.0, 129.0, 129.1, 129.1(2C), 129.1, 129.1, 129.2, 129.2, 129.2, 129.3, 129.3, 129.3, 129.8, 129.8, 129.8, 129.9(10C), 129.9(9C), 133.2(2C), 133.3, 133.3, 133.4, 133.4(2C), 133.5(2C), 133.5(2C), 133.5(2C), 133.6, 133.7, 133.9(2C), 165.1(2C), 165.2(3C), 165.2,

165.6, 165.7(2C), 165.7, 165.7(2C), 166.1, 172.5, 206.4; IR ($CHCl_3$): 3039, 2925, 1752, 1599, 1448, 1231, 1106, 705 cm^{-1}; HRMS (TOF) m/z $[M + Na]^+$ calcd for $C_{134}H_{120}O_{44}Na$, 2455.7049, found 2455.5154.

Allyl 2-*O*-benzoyl-3-*O*-(2,3-di-*O*-benzoyl-5-*O*-(2,3-di-*O*-benzoyl-5-*O*-(2,3-di-*O*-benzoyl-5-*O*-(4-oxopentanoyl)-α-D-arabinofuranosyl)-α-D-arabinofuranosyl)-α-D-arabinofuranosyl)-5-*O*-(2,3-di-*O*-benzoyl-5-*O*-(2,3-di-*O*-benzoyl-5-*O*-(2,3-di-*O*-benzoyl-5-*O*-(2-*O*-benzoyl-3-*O*-benzyl-5-*O*-(2-*O*-(2,3-di-*O*-benzyl-5-*O*-(2-*O*-(2-*O*-benzoyl-3,4,6-tri-*O*-benzyl-α-D-mannopyranosyl)-3,4,6-tri-*O*-benzyl-α-D-mannopyranosyl)-β-D-arabinofuranosyl)-3-*O*-benzyl-5-*O*-^{t}butyldiphenylsilyl-α-D-arabinofuranosyl)-α-D-arabinofuranosyl)-α-D-arabinofuranosyl)-α-D-arabinofuranosyl)-α-D-arabinofuranosyl)-α-D-arabinofuranoside (**44**): Yield: (85%); $[\alpha]_D^{25}$ = 15.2 (*c* = 1.0, $CHCl_3$); ^1H NMR (399.78 MHz, $CDCl_3$): δ 0.99 (s, 9H), 2.10 (s, 3H), 2.56 (dd, *J* = 10.3, 4.4 Hz, 2H), 2.68 (t, *J* = 6.6 Hz, 2H), 3.44 – 3.57 (m, 2H), 3.62 (dd, *J* = 18.6, 8.4 Hz, 3H), 3.68 – 3.78 (m, 6H), 3.80 (s, 1H), 3.82 – 3.88 (m, 7H), 3.94 (dd, *J* = 14.2, 6.6 Hz, 4H), 4.01 (dt, *J* = 14.4, 5.0 Hz, 6H), 4.09 (s, 4H), 4.17 (d, *J* = 11.3 Hz, 4H), 4.23 (dd, *J* = 13.1, 5.0 Hz, 1H), 4.28 (s, 1H), 4.32 (s, 1H), 4.35 (s, 1H), 4.40 (d, *J* = 11.4 Hz, 7H), 4.44 (d, *J* = 4.0 Hz, 2H), 4.47 (d, *J* = 5.3 Hz, 2H), 4.48 – 4.53 (m, 3H), 4.56 (d, *J* = 10.5 Hz, 8H), 4.62 (d, *J* = 11.5 Hz, 2H), 4.72 (d, *J* = 11.0 Hz, 1H), 4.83 (d, *J* = 10.9 Hz, 2H), 4.90 (s, 1H), 4.99 (s, 1H), 5.07 (d, *J* = 3.4 Hz, 1H), 5.11 – 5.18 (m, 2H), 5.22 (s, 1H), 5.26 – 5.31 (m, 3H), 5.32 – 5.41 (m, 5H), 5.43 (s, 2H), 5.51 (s, 1H), 5.57 (s, 3H), 5.59 – 5.64 (m, 8H), 5.68 (d, *J* = 4.5 Hz, 1H), 5.76 (s, 1H), 5.89 (ddt, *J* = 15.5, 10.4, 5.4 Hz, 1H), 7.08 (d, *J* = 7.3 Hz, 3H), 7.11 (s, 2H), 7.14 (d, *J* = 4. 3 Hz, 7H), 7.17 – 7.20 (m, 13H), 7.21 – 7.27 (m, 29H), 7.28 – 7.30 (m, 7H), 7.32 (d, *J* = 4.0 Hz, 5H), 7.36 (dd, *J* = 11.6, 5.2 Hz, 21H), 7.40 (s, 2H), 7.43 – 7.50 (m, 10H), 7.55 (d, *J* = 8.4 Hz, 2H), 7.62 (t, *J* = 8.1 Hz, 4H), 7.85 (t, *J* = 9.5 Hz, 8H), 7.92 (d, *J* = 8.1 Hz, 2H), 7.94 – 8.00 (m, 13H), 8.02 (s, 1H), 8.03 – 8.08 (m, 6H); ^{13}C NMR (100.53 MHz, $CDCl_3$): δ 19.4, 27.0(3C), 27.9, 29.8, 38.0, 63.7, 63.9, 65.7, 65.7, 65.8, 65.8, 66.1, 67.8, 69.0, 69.1, 69.1, 69.6, 71.7, 72.1, 72.2(2C), 72.2(2C), 72.3, 72.3, 72.4, 72.4, 73.4(3C), 74.3, 74.5, 75.1, 75.2, 75.3, 76.9, 77.0, 77.3, 77.4, 77.7, 78.3, 79.4, 80.0, 80.8, 81.2, 81.5(3C), 81.7, 81.8, 82.0, 82.1, 82.2, 82.3(2C), 82.6, 82.8, 82.9(2C), 83.1, 83.2, 83.5, 83.8, 84.1, 86.0, 98.7, 99.7, 100.7, 105.1, 105.4, 105.9(2C), 106.0, 106.0,

106.0, 106.2(2C), 117.4, 127.3(3C), 127.5(8C), 127.6(4C), 127.7(2C), 127.7(5C), 127.8(7C), 127.9, 128.0(3C), 128.0(3C), 128.1(3C), 128.2(3C), 128.2(3C), 128.3(8C), 128.4(14C), 128.4(3C), 128.5(3C), 128.5(5C), 128.5(12C), 128.6(3C), 129.0, 129.1, 129.1, 129.1, 129.2, 129.2(2C), 129.2(2C), 129.3(3C), 129.4, 129.6, 129.7(2C), 129.9(16C), 129.9(7C), 130.0(4C), 130.1, 133.1(2C), 133.1, 133.2, 133.3, 133.4(3C), 133.4(3C), 133.5(2C), 133.5, 133.6, 134.0(2C), 135.7, 135.7(3C), 137.7(3C), 137.8, 138.0, 138.1, 138.2, 138.2, 138.5, 138.6, 138.6, 138.6, 165.1(2C), 165.2, 165.2(2C), 165.3, 165.3, 165.4, 165.6(3C), 165.7(3C), 165.7, 172.5, 206.3; IR ($CHCl_3$): 3033, 2928, 1730, 1620, 1453, 1263, 1105, 703 cm^{-1}; MALDI (TOF) m/z $[M + Na]^+$ calcd for $C_{261}H_{250}O_{68}NaSi$, 4524.5839, found 4524.9119.

Allyl 2-*O*-benzoyl-3-*O*-(2,3-di-*O*-benzoyl-5-*O*-(2,3-di-*O*-benzoyl-5-*O*-(2,3-di-*O*-benzoyl-α-D-arabinofuranosyl)-α-D-arabinofuranosyl)-α-D-arabinofuranosyl)-5-*O*-(2,3-di-*O*-benzoyl-5-*O*-(2,3-di-*O*-benzoyl-5-*O*-(2,3-di-*O*-benzoyl-5-*O*-(2-*O*-benzoyl-3-*O*-benzyl-5-*O*-(2-*O*-(2,3-di-*O*-benzyl-5-*O*-(2-*O*-(2-*O*-benzoyl-3,4,6-tri-*O*-benzyl-α-D-mannopyranosyl)-3,4,6-tri-*O*-benzyl-α-D-mannopyranosyl)-β-D-arabinofuranosyl)-3-*O*-benzyl-5-*O*-^{t}butyldiphenylsilyl-α-D-arabinofuranosyl)-α-D-arabinofuranosyl)-α-D-arabinofuranosyl)-α-D-arabinofuranosyl)-α-D-arabinofuranosyl)-α-D-arabinofuranoside (**45**): Yield: (80%); $[\alpha]_D^{25}$ = 16.3 (*c* = 1.0, $CHCl_3$); ^1H NMR (399.78 MHz, $CDCl_3$):) δ 0.99 (s, 9H), 3.52 (dd, *J* = 17.8, 8.7 Hz, 2H), 3.62 (dd, *J* = 18.7, 8.6 Hz, 3H), 3.67 – 3.80 (m, 7H), 3.84 (d, *J* = 7.4 Hz, 8H), 3.91 – 4.04 (m, 12H), 4.06 – 4.11 (m, 4H), 4.12 – 4.17 (m, 3H), 4.18 – 4.26 (m, 2H), 4.27 (s, 1H), 4.39 (d, *J* = 11.4 Hz, 8H), 4.45 (dd, *J* = 17.1, 5.1 Hz, 6H), 4.51 (d, *J* = 6.2 Hz, 2H), 4.54 – 4.64 (m, 8H), 4.72 (d, *J* = 11.0 Hz, 1H), 4.83 (dd, *J* = 10.9, 2.2 Hz, 2H), 4.90 (s, 1H), 4.98 (s, 1H), 5.07 (d, *J* = 3.9 Hz, 1H), 5.11 – 5.17 (m, 2H), 5.21 (s, 1H), 5.26 (s, 1H), 5.30 (s, 2H), 5.32 – 5.40 (m, 5H), 5.43 (s, 2H), 5.48 (s, 1H), 5.58 (dd, *J* = 15.0, 5.7 Hz, 9H), 5.63 (d, *J* = 4.5 Hz, 1H), 5.67 (d, *J* = 4.2 Hz, 1H), 5.76 (s, 1H), 5.88 (dq, *J* = 15.6, 4.9 Hz, 1H), 7.06 – 7.15 (m, 14H), 7.17 – 7.25 (m, 38H), 7.28 (d, *J* = 3.1 Hz, 10H), 7.31 (s, 3H), 7.36 (dt, *J* = 15.4, 7.9 Hz, 24H), 7.43 – 7.50 (m, 9H), 7.54 (t, *J* = 7.5 Hz, 2H), 7.61 (t, *J* = 8.1 Hz, 4H), 7.81 – 7.88 (m, 8H), 7.91 (d, *J* = 7.4 Hz, 2H), 7.95 – 8.02 (m, 16H), 8.04 – 8.08 (m, 5H); ^{13}C NMR (100.53 MHz, $CDCl_3$): δ 19.4, 27.0, 62.4, 64.0, 65.7(2C), 65.8, 66.0, 66.1, 67.8, 69.0, 69.1, 69.1, 69.6, 71.7(2C), 72.1, 72.2(2C), 72.3(2C), 72.3(2C), 72.3, 72.4, 72.5, 73.4,

73.4, 74.3, 74.5, 75.1, 75.2, 75.3, 77.0, 77.2, 77.2, 77.3, 77.4, 77.8, 78.3, 79.4, 80.0, 80.8, 81.6(3C), 81.7, 81.7, 81.8(2C), 82.0, 82.1, 82.3(2C), 82.6, 82.8(3C), 82.9, 83.2, 83.5, 83.8(2C), 83.8, 84.1, 86.0, 98.7, 99.7, 100.7, 105.1, 105.4, 105.9(4C), 106.0, 106.2(2C), 117.4, 127.4(3C), 127.5(8C), 127.6(5C), 127.7(3C), 127.7(3C), 127.8(2C), 127.8(3C), 127.9, 128.0 (3C), 128.0(3C), 128.1(3C), 128.2(3C), 128.3(2C), 128.3(5C), 128.3(3C), 128.4(8C), 128.4(10C), 128.4(2C), 128.5(4C), 128.5(6C), 128.6(10C), 129.1(2C), 129.1, 129.2, 129.2(2C), 129.3, 129.3(3C), 129.4, 129.6, 129.8(2C), 129.9(20C), 130.0(5C), 130.1(3C), 130.1, 133.1(2C), 133.2, 133.3, 133.3(2C), 133.4(3C), 133.5(4C), 133.6, 133.6, 134.0(2C), 135.7, 135.8, 137.7, 137.9, 138.0, 138.1, 138.2, 138.3, 138.6, 138.6, 138.6, 138.7, 165.2(2C), 165.2(3C), 165.3, 165.4, 165.5, 165.6(3C), 165.7, 165.7(2C), 166.1; IR ($CHCl_3$): 3430, 3030, 2934,, 1590, 1447, 1229, 1105, 694 cm^{-1}; MALDI (TOF) m/z $[M + Na]^+$ calcd for $C_{256}H_{244}O_{66}Na_2Si$, 4426.5471, found 4426.8154.

Allyl 2-*O*-benzoyl-3-*O*-(2,3-di-*O*-benzoyl-5-*O*-(2,3-di-*O*-benzoyl-5-*O*-(2,3-di-*O*-benzoyl-5-*O*-(2-*O*-benzoyl-3,5-di-*O*-(2-*O*-(2,3-di-*O*-benzyl-5-*O*-(2-*O*-(2-*O*-benzoyl-3,4,6-tri-*O*-benzyl-α-D-mannopyranosyl)-3,4,6-tri-*O*-benzyl-α-D-mannopyranosyl)-β-D-arabinofuranosyl)-3-*O*-benzyl-5-*O*-^{t}butyldiphenylsilyl-α-D-arabinofuranosyl)-α-D-arabinofuranosyl)-α-D-arabinofuranosyl)-α-D-arabinofuranosyl)-α-D-arabinofuranosyl)-5-*O*-(2,3-di-*O*-benzoyl-5-*O*-(2,3-di-*O*-benzoyl-5-*O*-(2,3-di-*O*-benzoyl-5-*O*-(2-*O*-benzoyl-3-*O*-benzyl-5-*O*-(2-*O*-(2,3-di-*O*-benzyl-5-*O*-(2-*O*-(2-*O*-benzoyl-3,4,6-tri-*O*-benzyl-α-D-mannopyranosyl)-3,4,6-tri-*O*-benzyl-α-D-mannopyranosyl)-β-D-arabinofuranosyl)-3-*O*-benzyl-5-*O*-^{t}butyldiphenylsilyl-α-D-arabinofuranosyl)-α-D-arabinofuranosyl)-α-D-arabinofuranosyl)-α-D-arabinofuranosyl)-α-D-arabinofuranosyl)-α-D-arabinofuranoside (**46**): Yield: (80%); $[\alpha]_D^{25}$ = 5.4 (*c* = 1.0, $CHCl_3$); ^1H NMR (399.78 MHz, $CDCl_3$): δ 0.88 (s, 9H), 0.95 (s, 9H), 0.98 (s, 9H), 3.44 – 3.54 (m, 5H), 3.56 (dd, *J* = 15.0, 6.6 Hz, 4H), 3.60 – 3.65 (m, 4H), 3.68 (dd, *J* = 21.6, 9.0 Hz, 9H), 3.72 – 3.80 (m, 11H), 3.84 (t, *J* = 12.2 Hz, 9H), 3.88 – 3.95 (m, 9H), 3.95 – 4.04 (m, 19H), 4.09 (d, *J* = 16.0 Hz, 12H), 4.13 – 4.24 (m, 5H), 4.27 (d, *J* = 6.7 Hz, 2H), 4.31 (dd, *J* = 10.8, 3.7 Hz, 5H), 4.33 – 4.36 (m, 4H), 4.36 – 4.40 (m, 11H), 4.43 (d, *J* = 8.5 Hz, 5H), 4.44 – 4.47 (m, 4H), 4.48 – 4.51 (m, 7H), 4.52 – 4.57 (m, 16H), 4.60 (dd, *J* = 13.6, 8.2 Hz, 5H), 4.71 (dd, *J* = 10.9, 5.7 Hz, 3H), 4.82 (dd, *J* = 10.4, 6.8 Hz, 6H), 4.85 – 4.89 (m, 3H), 4.98

(s, 1H), 5.06 (s, 2H), 5.09 – 5.18 (m, 6H), 5.20 (s, 2H), 5.26 (d, *J* = 3.6 Hz, 2H), 5.32 (dd, *J* = 21.2, 5.0 Hz, 4H), 5.37 (d, *J* = 11.7 Hz, 2H), 5.42 (s, 2H), 5.53 (d, *J* = 21.6 Hz, 4H), 5.57 – 5.63 (m, 6H), 5.67 (d, *J* = 3.9 Hz, 1H), 5.75 (s, 3H), 5.85 (ddq, *J* = 23.3, 12.6, 6.7, 5.9 Hz, 1H), 7.01 (dt, J = 12.1, 6.5 Hz, 2H), 7.06 (dd, *J* = 14.6, 7.1 Hz, 12H), 7.09 – 7.15 (m, 30H), 7.18 (dq, *J* = 14.1, 7.5, 6.8 Hz, 48H), 7.21 – 7.31 (m, 86H), 7.31 – 7.36 (m, 22H), 7.36 – 7.40 (m, 5H), 7.41 – 7.49 (m, 7H), 7.50 – 7.56 (m, 6H), 7.56 – 7.63 (m, 9H), 7.75 – 7.81 (m, 3H), 7.84 (dd, *J* = 13.0, 7.7 Hz, 4H), 7.88 – 7.93 (m, 4H), 7.97 (dd, *J* = 18.9, 7.6 Hz, 12H), 8.01 – 8.07 (m, 10H); ^{13}C NMR (100.53 MHz, $CDCl_3$): δ 19.4(3C), 26.9(9C), 63.6, 63.7(2C), 64.0(2C), 65.4, 65.6(2C), 65.6(2C), 65.7(2C), 65.7, 65.8(2C), 65.8(2C), 66.3, 67.8(3C), 69.0(2C), 69.0, 69.1(5C), 69.1, 69.6(2C), 69.7, 71.7(3C), 71.9, 72.0, 72.1, 72.3(7C), 72.3(2C), 72.4, 72.5, 73.4(3C), 73.4(3C), 73.4(4C), 74.3(2C), 74.3, 74.5(2C), 75.0(2C), 75.1, 75.2(2C), 75.3(2C), 75.3, 75.3(2C), 75.3(3C), 78.3, 78.3, 79.2, 79.4, 79.4, 80.0(3C), 81.0, 81.5, 81.6(3C), 81.7, 81.7, 81.8, 81.9, 82.0(2C), 82.3(2C), 82.3, 82.3(2C), 82.5, 82.5, 82.8(2C), 83.0, 83.0, 83.1, 83.4, 83.4, 83.5, 83.8(2C), 83.9, 84.2, 84.2, 84.4(2C), 85.6, 86.0, 86.3, 98.8(2C), 98.8, 99.7, 99.7(2C), 100.5, 100.7(2C), 105.1, 105.4, 105.9(4C), 106.0(2C), 106.2(2C), 106.4, 107.3, 117.4, 127.4(6C), 127.4, 127.5(2C), 127.5(6C), 127.5(12C), 127.5(12C), 127.6(10C), 127.7(3C), 127.7(10C), 127.8(7C), 127.8(7C), 127.8(6C), 127.9, 127.9(2C), 128.0(2C), 128.0(6C), 128.1(5C), 128.1(6C), 128.2(2C), 128.2(6C), 128.3(4C), 128.3(18C), 128.4(14C), 128.4(18C), 128.5(6C), 128.5(5C), 128.5(8C), 128.5(5C), 128.6(4C), 128.6(4C), 128.7, 129.1, 129.1, 129.2, 129.2, 129.2, 129.2(2C), 129.3(2C), 129.3, 129.4, 129.4, 129.5, 129.6(2C), 129.7(3C), 129.8(3C), 129.8(2C), 129.9(3C), 129.9(6C), 129.9(6C), 130.0(2C), 130.0(3C), 130.1(6C), 130.1(2C), 130.2, 133.0, 133.1, 133.1(2C), 133.2(2C), 133.3, 133.4(2C), 133.4(2C), 133.4(2C), 133.5(2C), 133.5, 133.5, 133.6, 134.0(2C), 135.6(2C), 135.7(3C), 135.7(3C), 135.8(3C), 137.7, 137.7, 137.9, 137.9, 138.0, 138.0, 138.1, 138.1, 138.1, 138.2, 138.2, 138.2, 138.2, 138.3(2C), 138.3, 138.6(2C), 138.6(3C), 138.6(2C), 138.6, 138.7(2C), 138.7, 138.7, 139.4, 165.2, 165.2, 165.2(3C), 165.3, 165.3, 165.4, 165.4, 165.5(2C), 165.6(2C), 165.7(4C), 165.7; IR ($CHCl_3$): 3035, 2926, 1600, 1444, 1266, 1106, 699 cm^{-1}; MALDI (TOF) m/z [M + Na]$^+$ calcd for $C_{484}H_{480}O_{109}NaSi_2$, 8147.1390, found 8144.5210.

Propyl 3-*O*-(5-*O*-(5-*O*-(5-*O*-(3,5-di-*O*-(2-*O*-(5-*O*-(2-*O*-(α-D-mannopyranosyl)-α-D-mannopyranosyl)-β-D-arabinofuranosyl)-α-D-arabinofuranosyl)-α-D-arabinofuranosyl)-α-D-arabinofuranosyl)-α-D-arabinofuranosyl)-α-D-arabinofuranosyl)-5-*O*-(5-*O*-(5-*O*-(5-*O*-(5-*O*-(2-*O*-(5-*O*-(2-*O*-(α-D-mannopyranosyl)-α-D-mannopyranosyl)-β-D-arabinofuranosyl)-α-D-arabinofuranosyl)-α-D-arabinofuranosyl)-α-D-arabinofuranosyl)-α-D-arabinofuranosyl)-α-D-arabinofuranosyl)-α-D-arabinofuranoside (**47**): Yield: (54% over three steps); $[\alpha]_D^{25}$ = 32.5 (5mg/ml, H_2O:MeOH=4:1); ^1H NMR (600.40 MHz, D_2O): 0.81 (t, *J* = 7.4 Hz, 3H), 1.51 (dd, *J* = 14.1, 7.1 Hz, 2H), 3.45 (dd, *J* = 16.3, 6.5 Hz, 1H), 3.52 (dd, *J* = 19.3, 9.6 Hz, 6H), 3.65 – 3.58 (m, 14H),3.68 (dd, *J* = 15.7, 8.4 Hz, 12H), 3.74 (dd, *J* = 11.1, 7.3 Hz, 10H), 3.79 (t, *J* = 9.7 Hz, 15H), 3.85 (dd, *J* = 9.9, 6.9 Hz, 4H), 3.92 (s, 15H), 4.06 – 3.97 (m, 23H), 4.08 (s, 3H), 4.12 (d, *J* = 3.9 Hz, 7H), 4.19 (d, *J* = 13.9 Hz, 3H), 4.93 (s, 3H), 4.96 (s, 1H), 4.99 (s, 6H), 5.02 (s, 1H), 5.06 (d, *J* = 6.5 Hz, 7H), 5.09 (s, 2H), 5.16 (s, 1H); ^{13}C NMR (150.99 MHz, D_2O): δ 9.8, 22.0, 58.9, 60.6, 60.6, 60.7(2C), 61.1(2C), 61.7, 66.1(2C), 66.2, 66.6, 66.6(2C), 66.6, 66.8, 66.8, 66.9(6C), 67.8, 68.2(3C), 69.9(3C), 70.0, 70.1(2C), 70.2(2C), 72.8(2C), 73.2(3C), 74.0(2C), 75.0(3C), 75.0, 75.1, 76.0(3C), 76.5, 76.6, 76.6, 76.7, 76.7, 76.7, 76.7, 78.7(2C), 79.1, 79.1(2C), 79.7(2C), 79.7(2C), 80.8, 80.8(4C), 80.8, 80.8, 81.0, 81.3, 81.6, 81.7, 82.1, 82.2, 82.3, 82.3(2C), 82.4, 83.1, 83.1(2C), 83.1, 86.9, 86.9, 87.0, 87.4, 98.2(3C), 100.4, 100.5, 100.7, 102.3(3C), 105.4, 105.5, 105.6, 107.2, 107.3, 107.4, 107.5(2C), 107.5(4C); HRMS (MALDI-TOF) m/z $[M + Na]^+$ calcd for $C_{114}H_{188}O_{91}Na^+$, 3037.00, found 3036.55.

^{1}H NMR Spectrum (399.78 MHz, $CDCl_3$) of Compound **8**

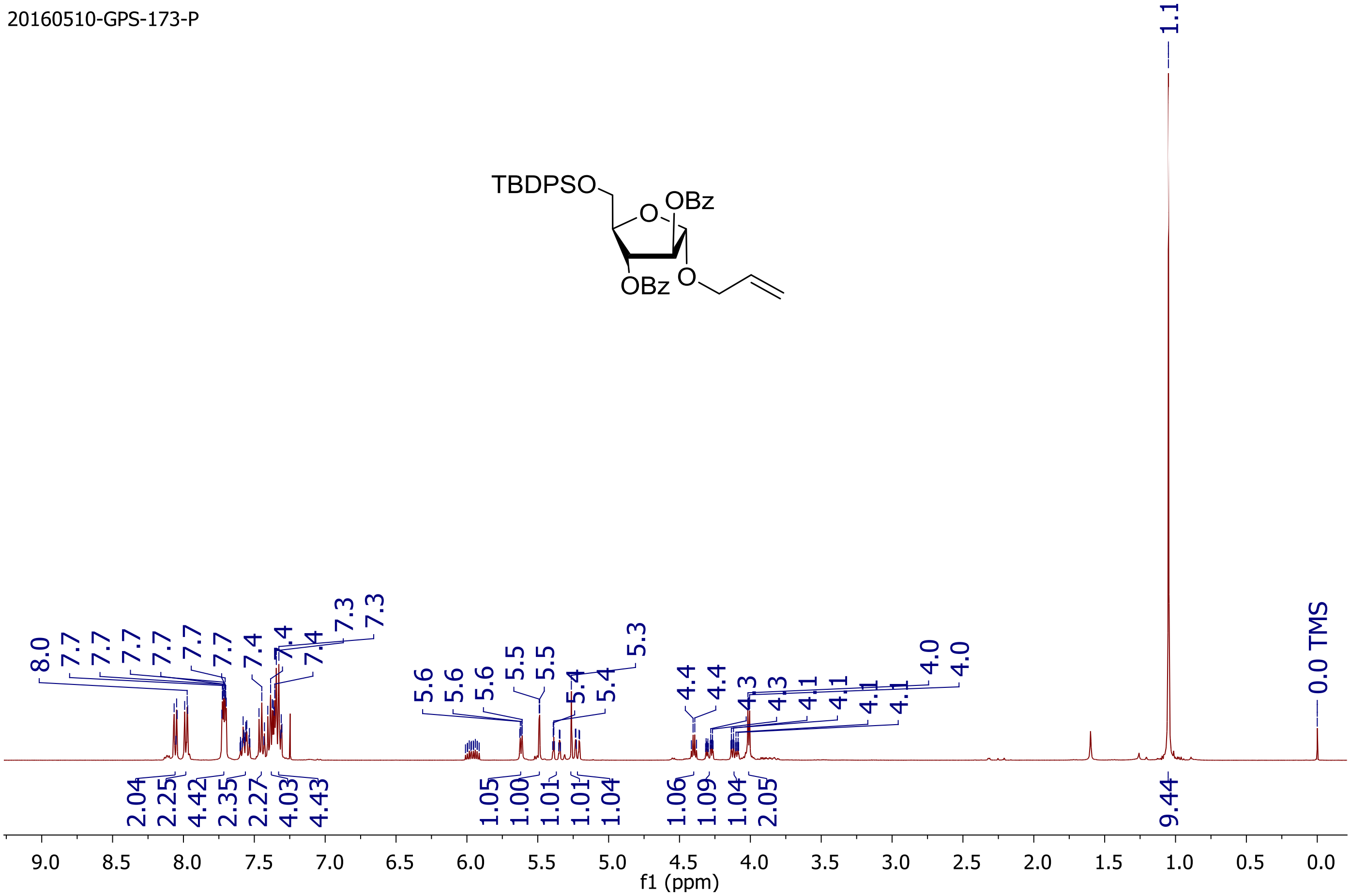

^{13}C NMR Spectrum (100.53 MHz, $CDCl_3$) of Compound **8**

20160510-GPS-173-P

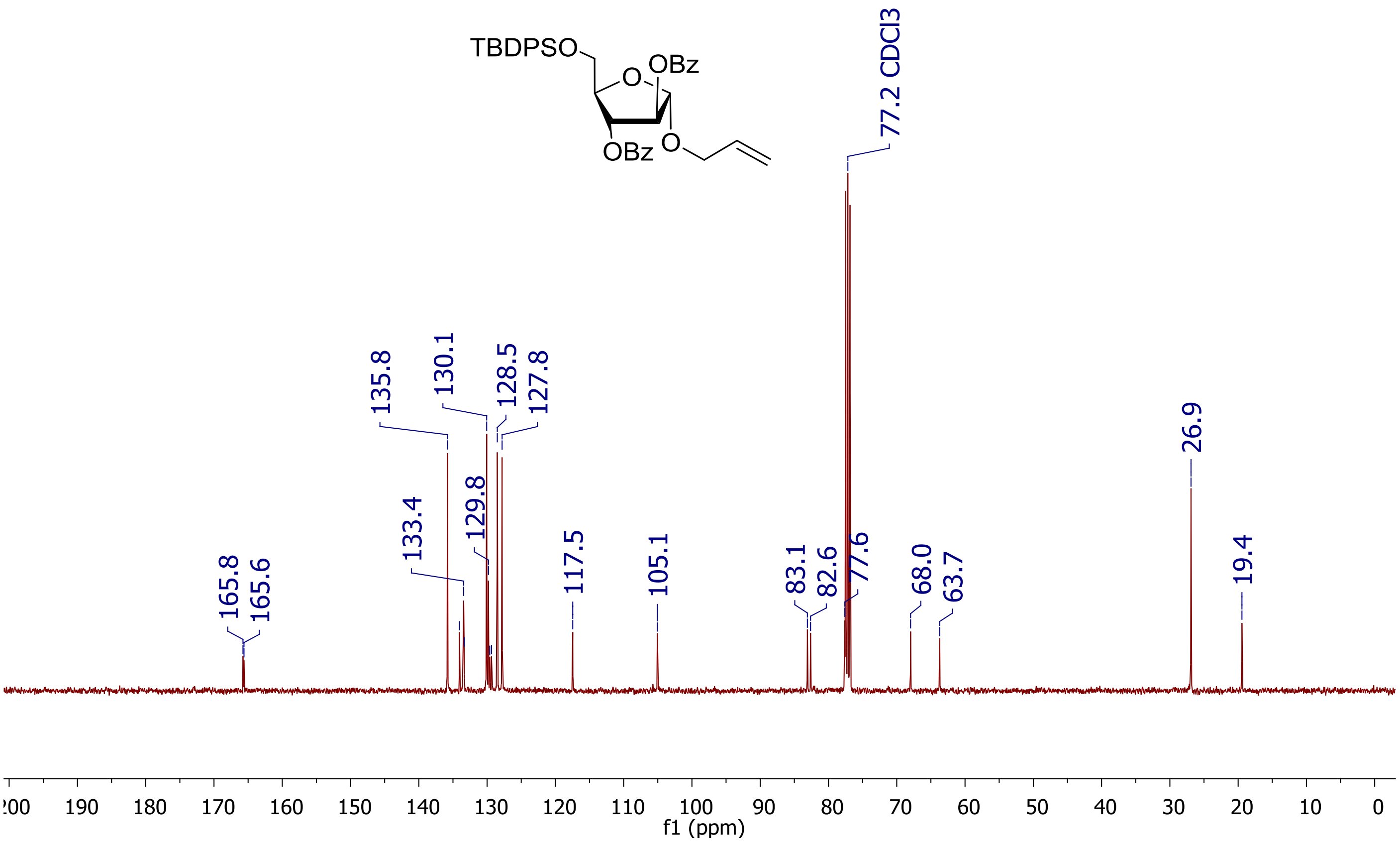

DEPT Spectrum (100.53 MHz, $CDCl_3$) of Compound **8**

20160510-GPS-173-P

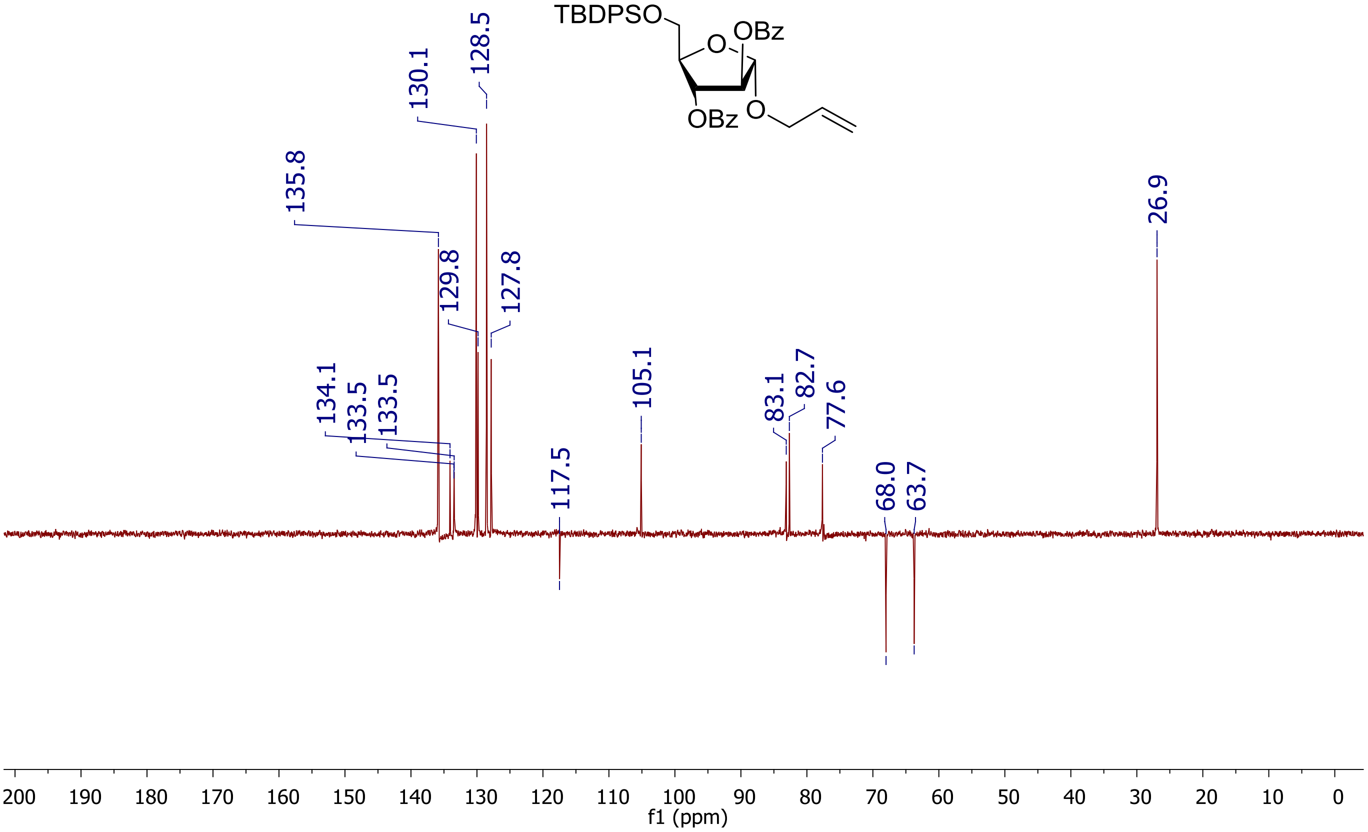

^{1}H NMR Spectrum (399.78 MHz, $CDCl_3$) of Compound **9**

20160416-MI-5-76A.12.fid
MI-5-76A

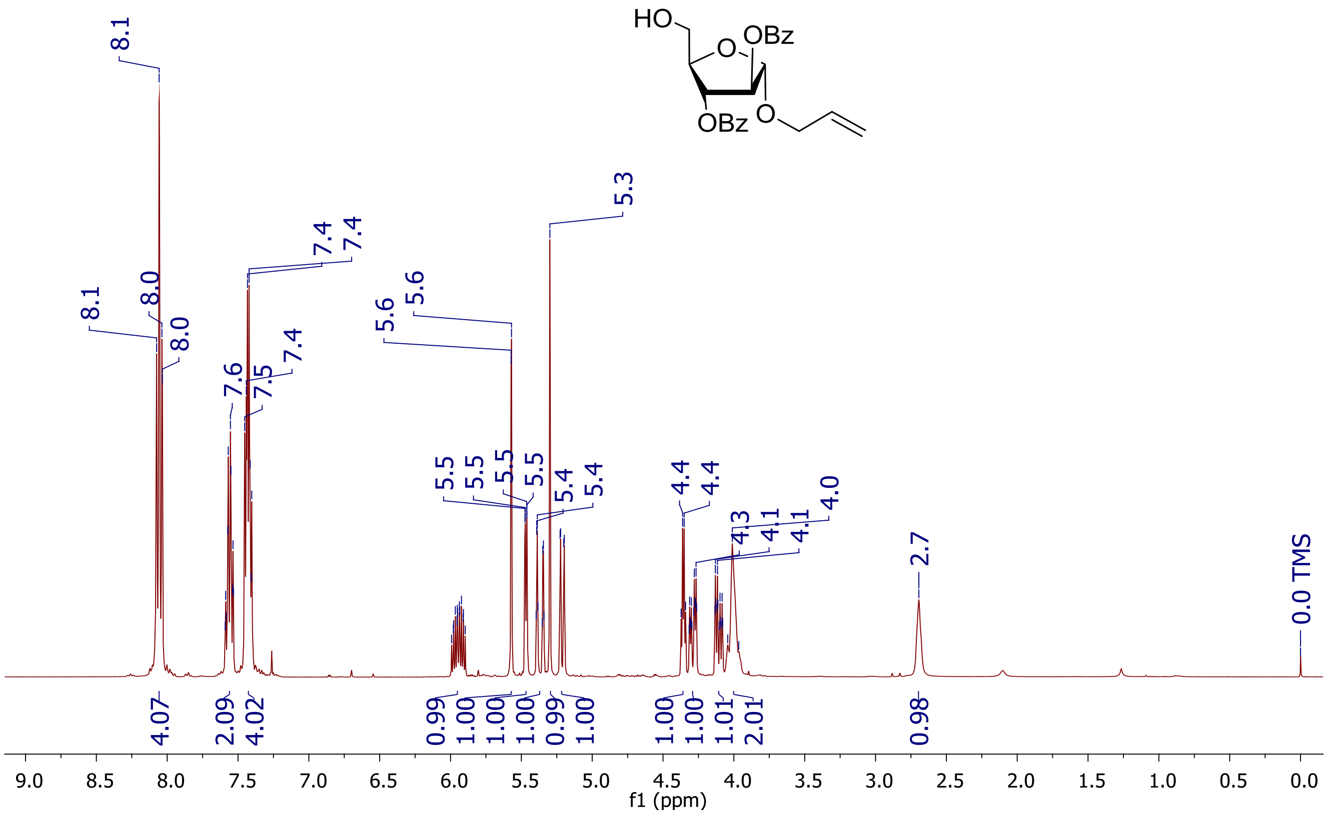

^{13}C NMR Spectrum (100.53 MHz, $CDCl_3$) of Compound **9**

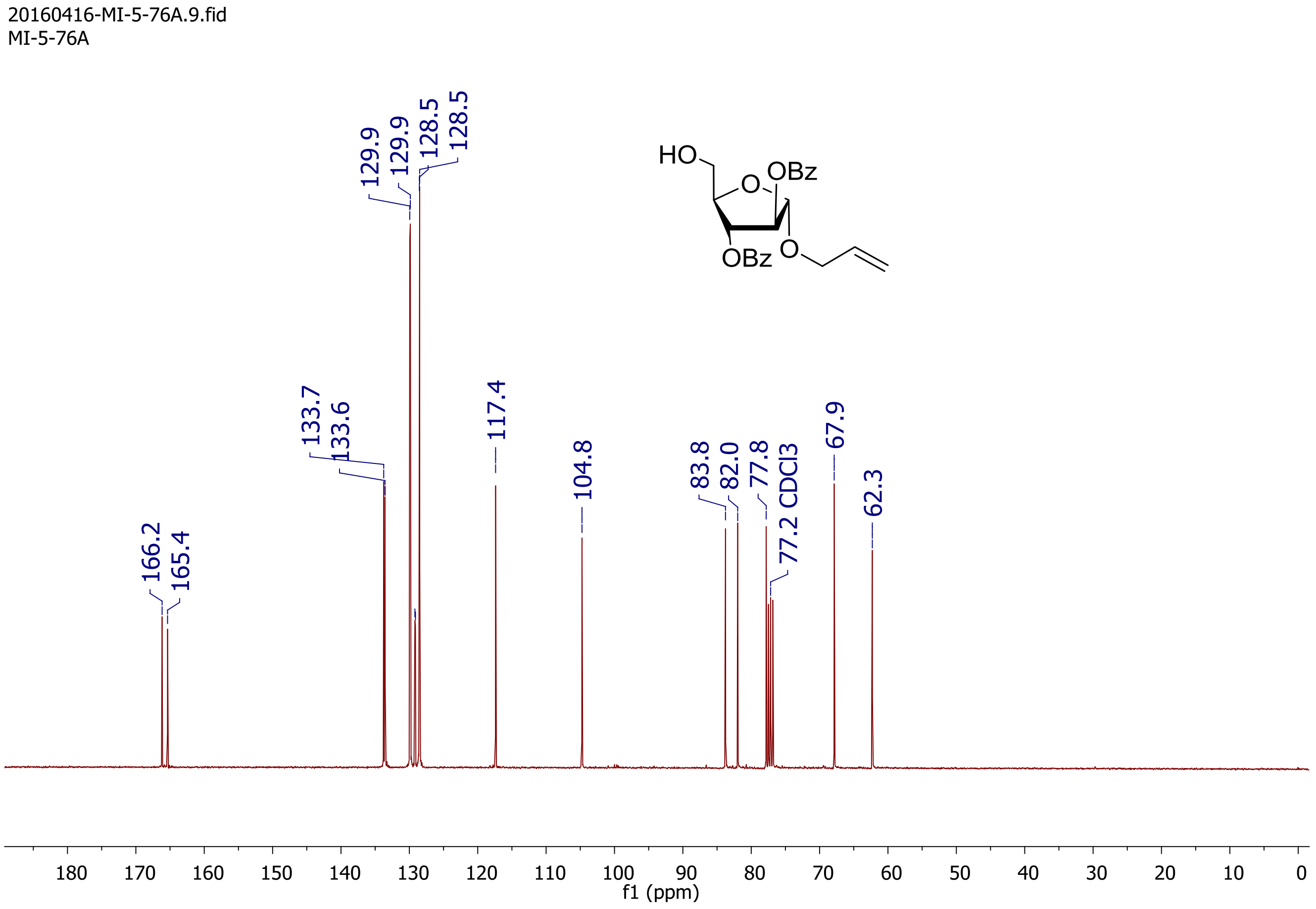

DEPT Spectrum (100.53 MHz, $CDCl_3$) of Compound **9**

20160416-MI-5-76A.11.fid
MI-5-76A

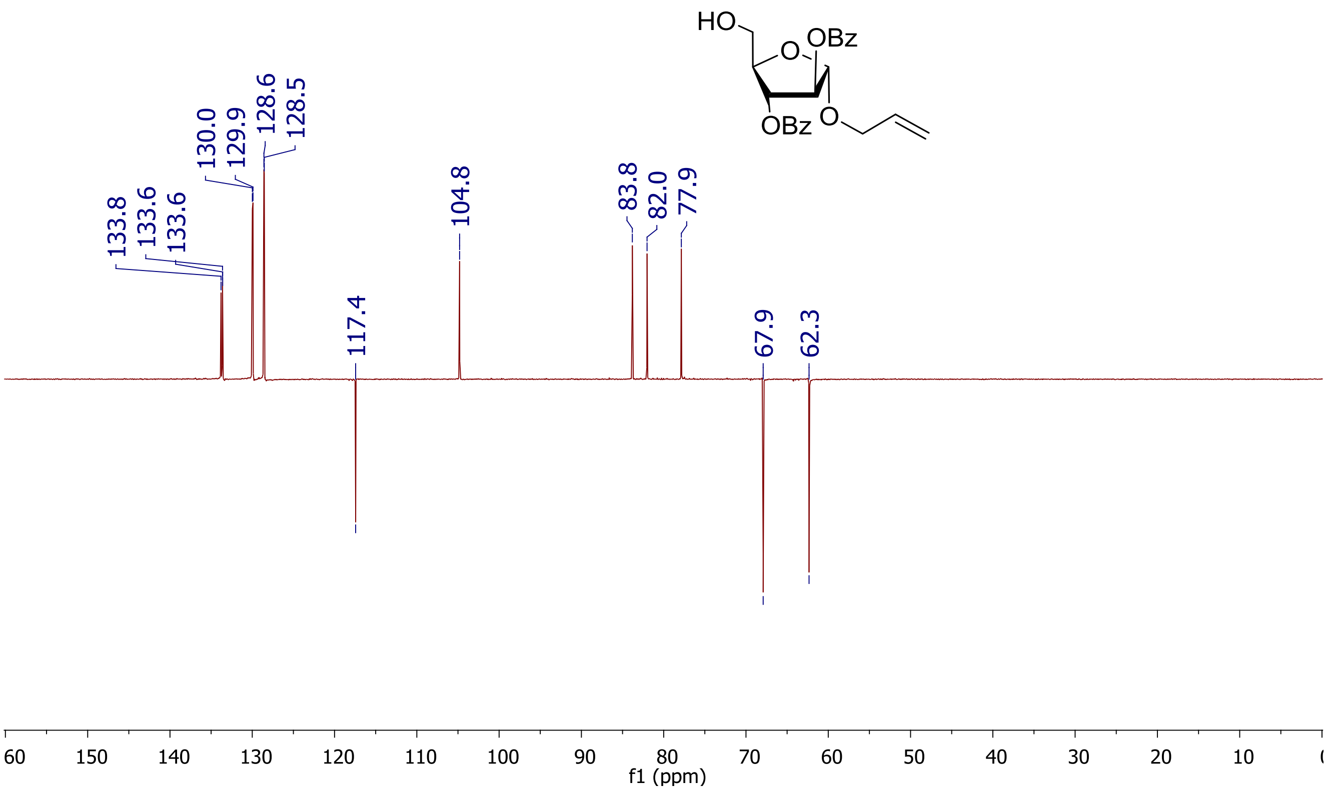

^{1}H NMR Spectrum (399.78 MHz, $CDCl_3$) of Compound **12**

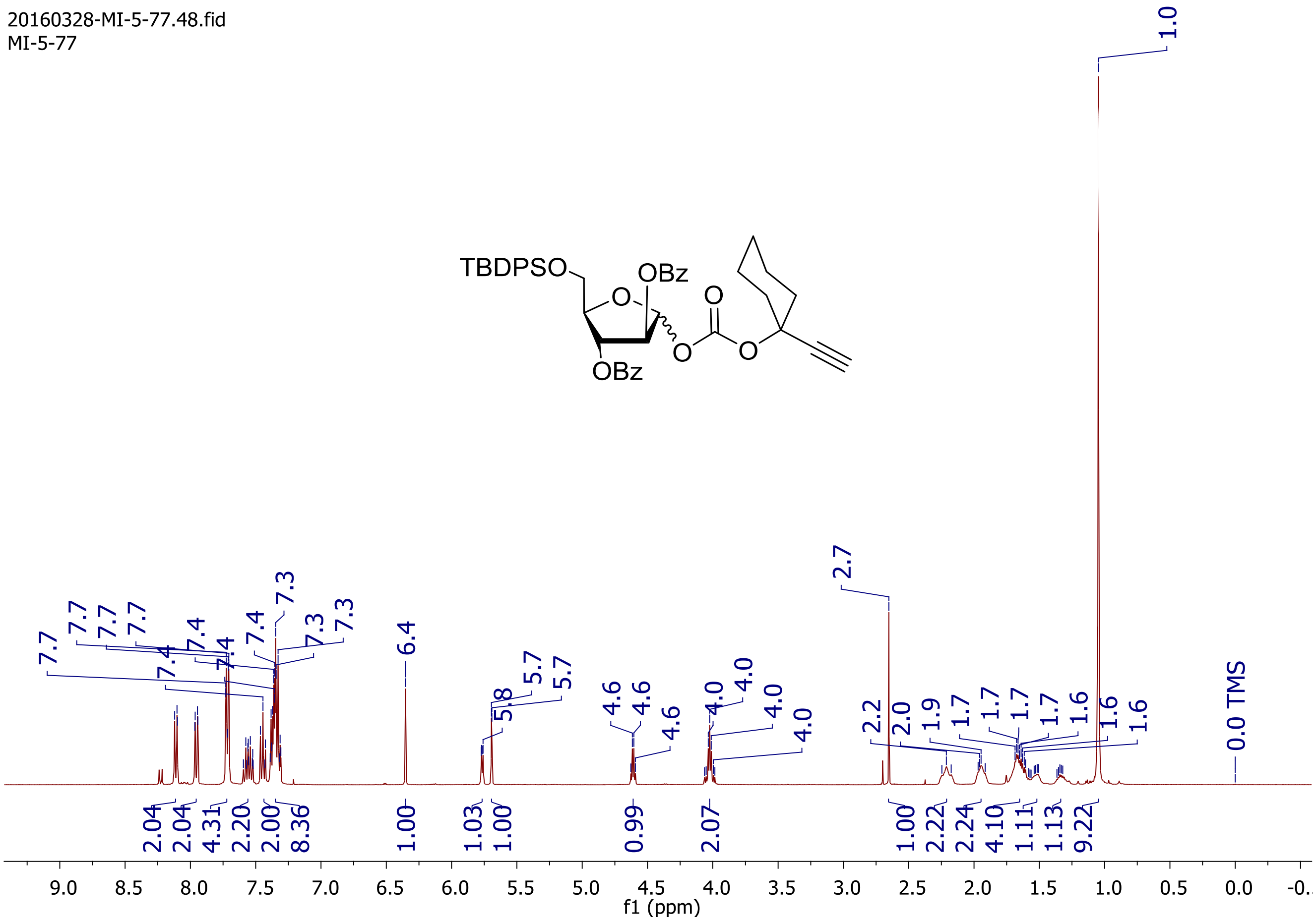

^{13}C NMR Spectrum (100.53 MHz, $CDCl_3$) of Compound **12**

20160328-MI-5-77.50.fid
MI-5-77

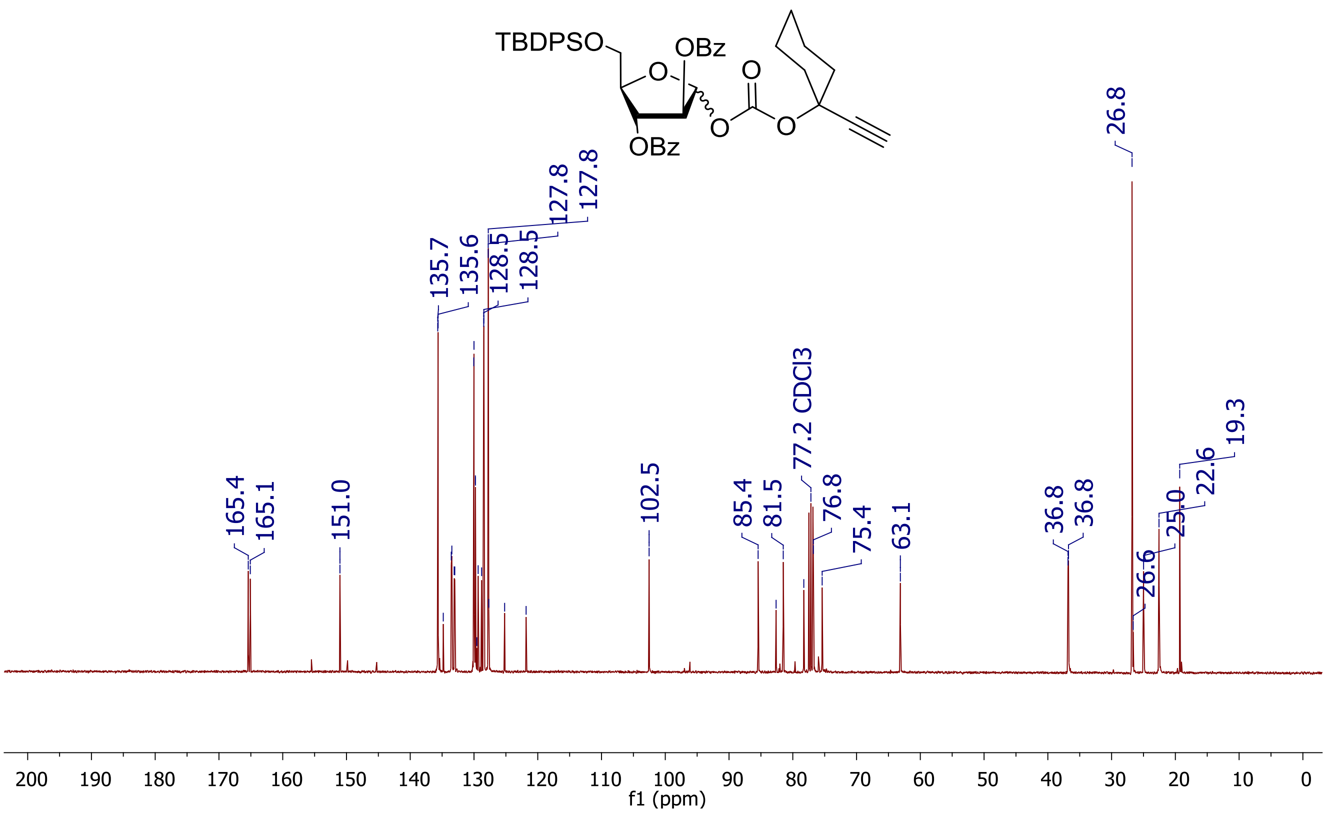

DEPT Spectrum (100.53 MHz, $CDCl_3$) of Compound **12**

20160328-MI-5-77.51.fid
MI-5-77

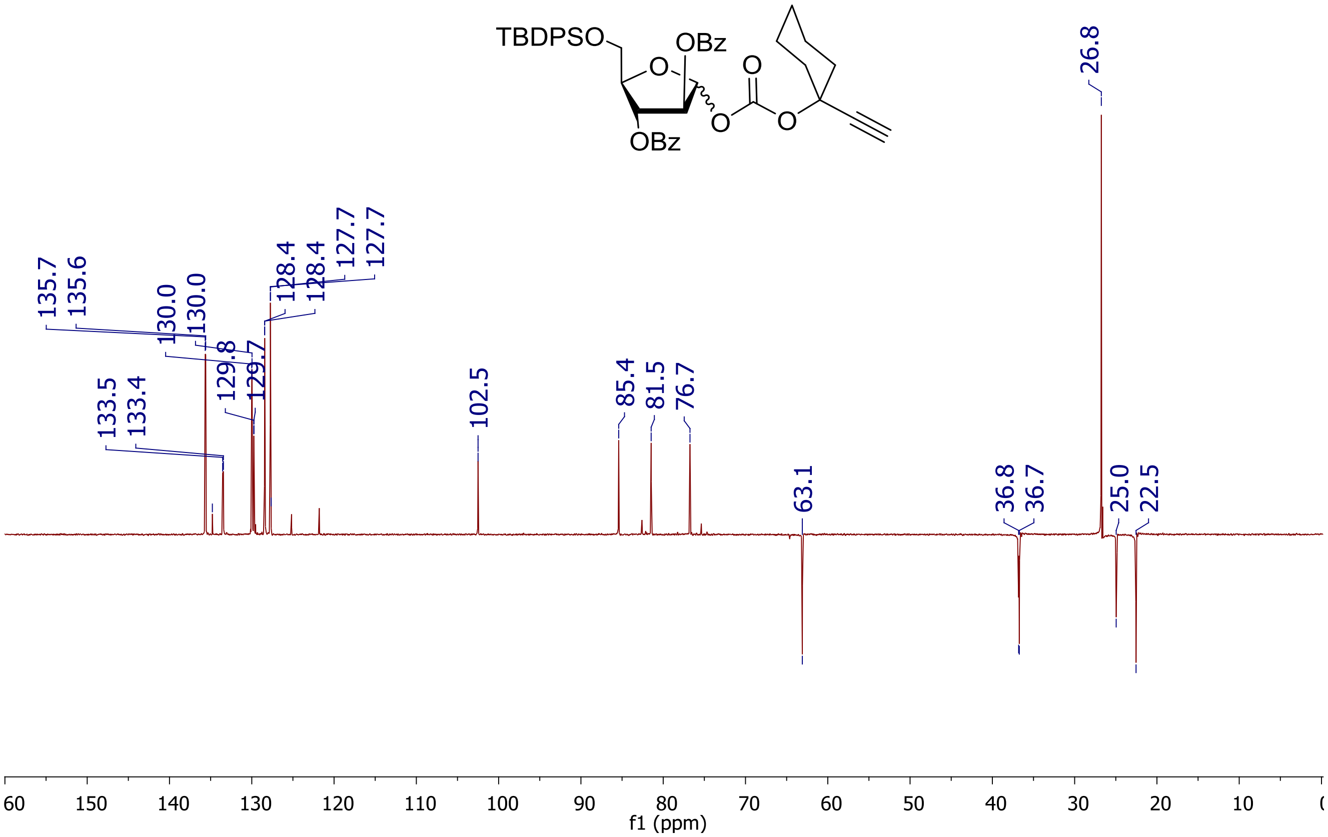

^{1}H NMR Spectrum (399.78 MHz, $CDCl_3$) of Compound **13**

20160328-MI-5-63/42
MI-5-63

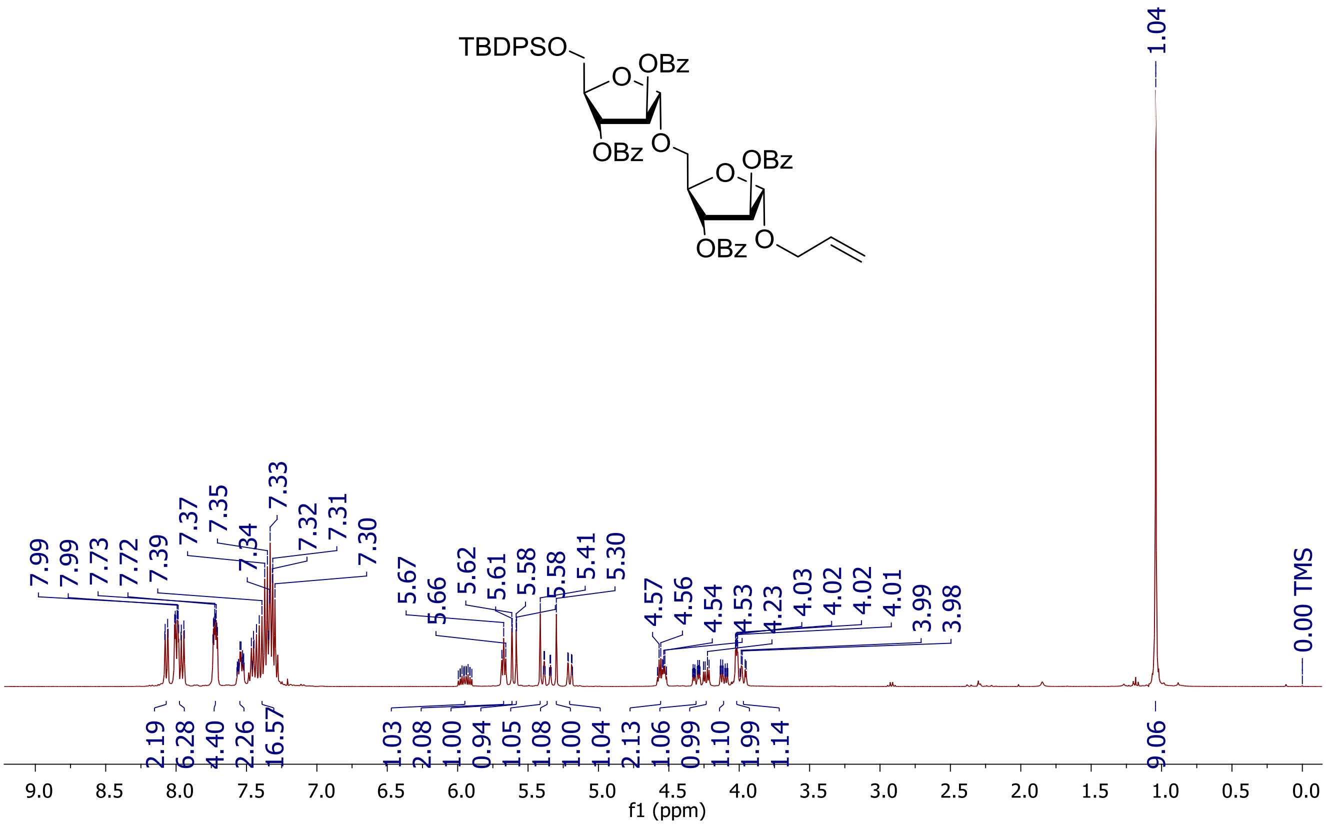

^{13}C NMR Spectrum (100.53 MHz, $CDCl_3$) of Compound **13**

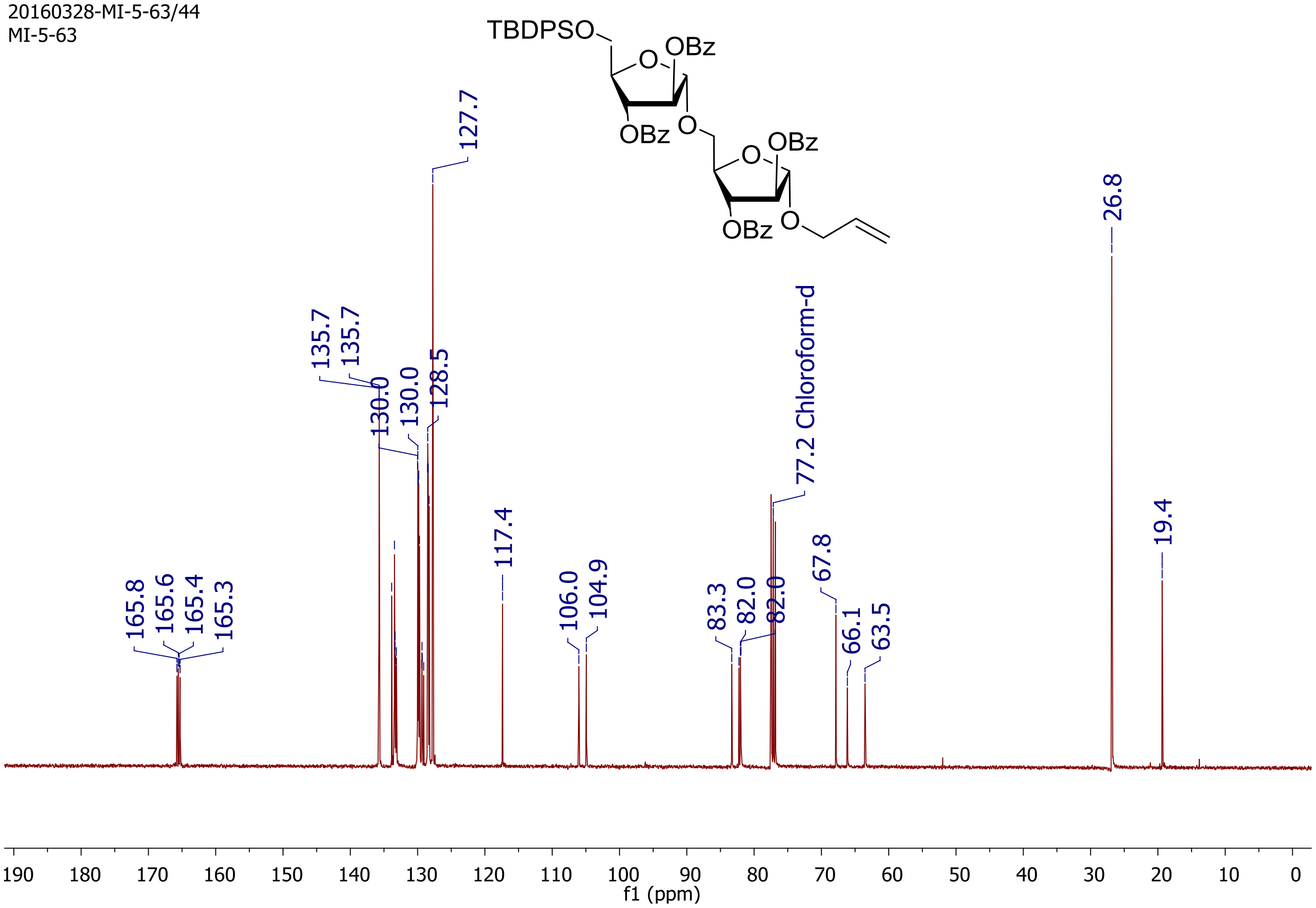

DEPT Spectrum (100.53 MHz, $CDCl_3$) of Compound **13**

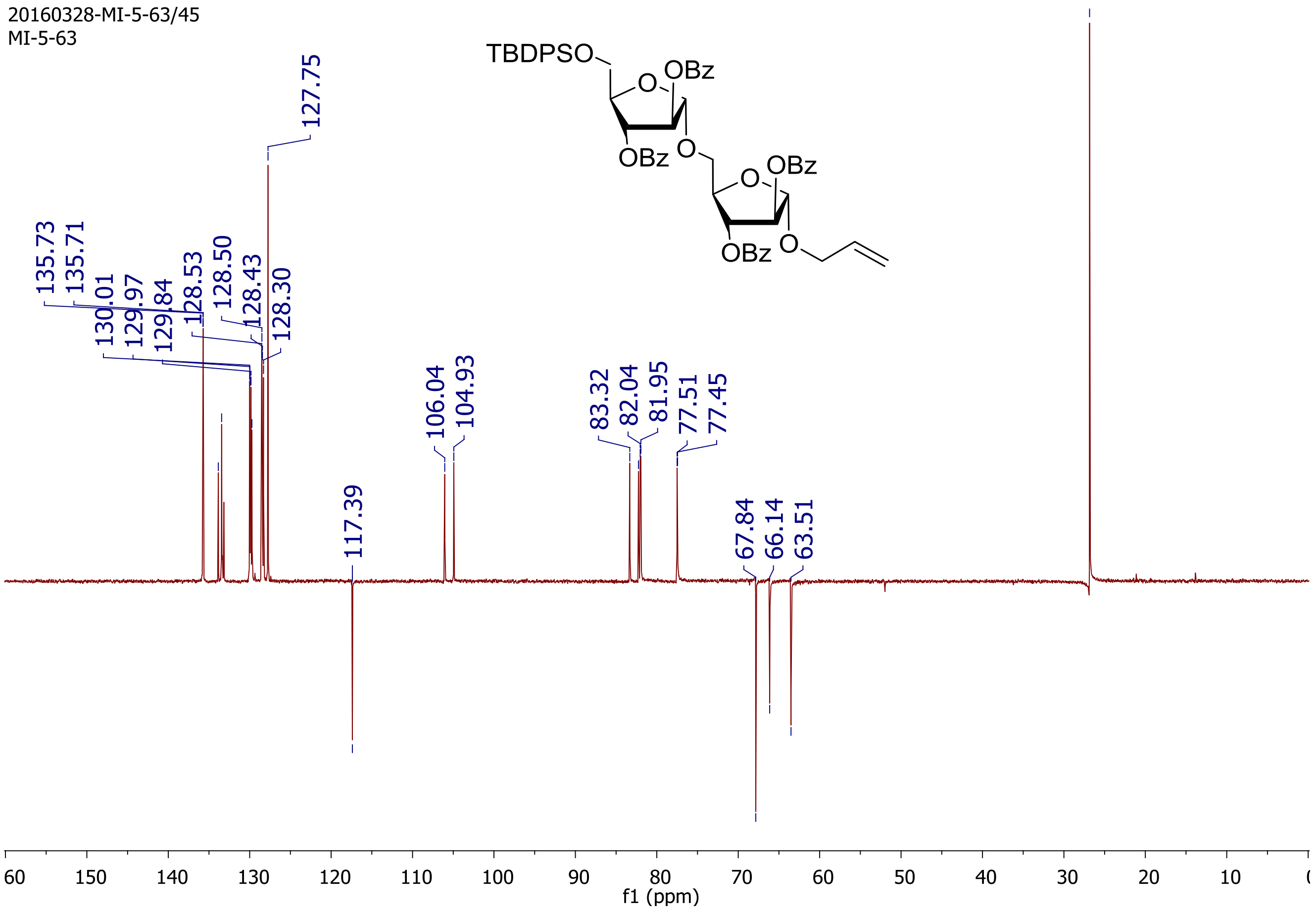

^{1}H NMR Spectrum (399.78 MHz, $CDCl_3$) of Compound **14**

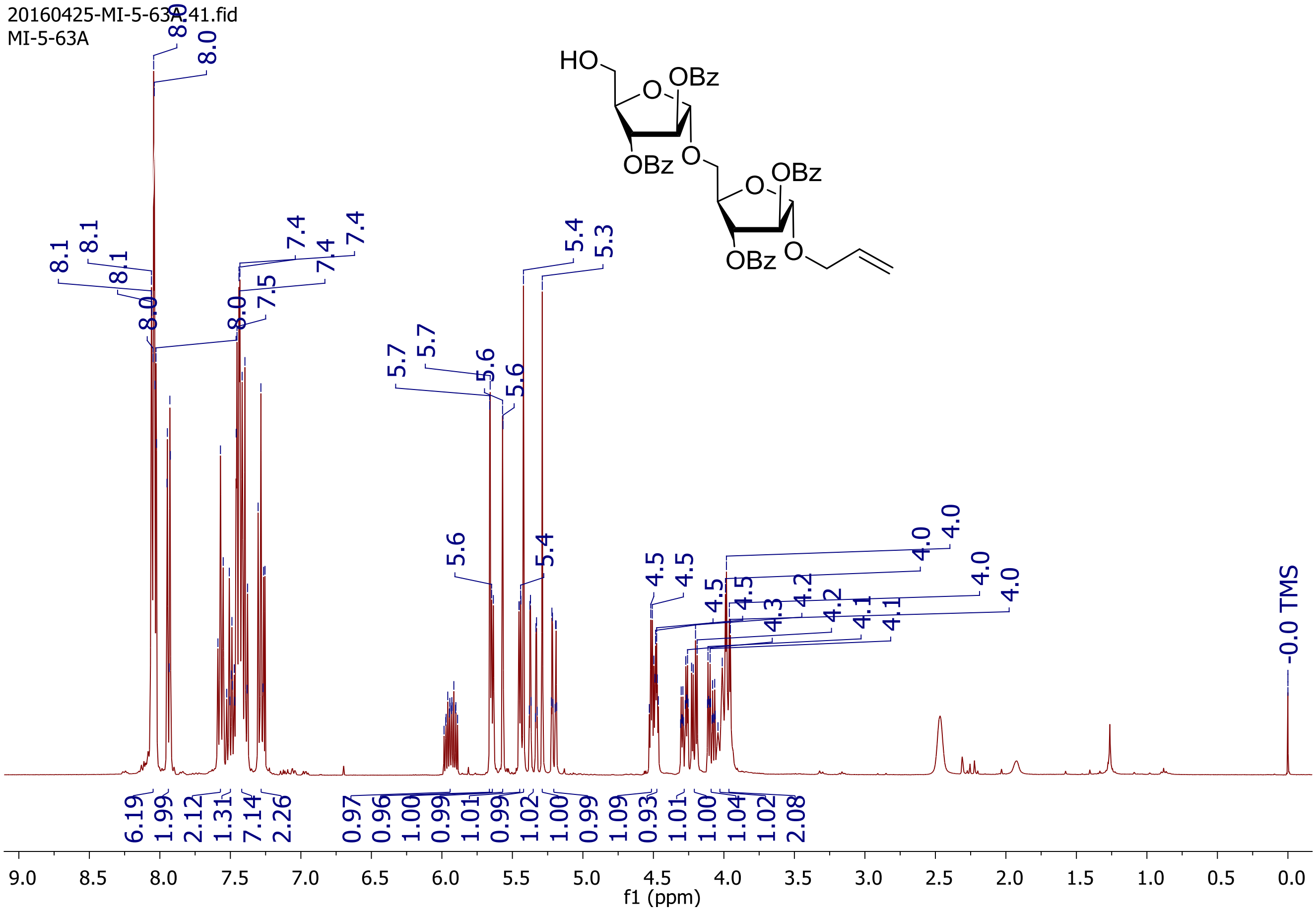

^{13}C NMR Spectrum (100.53 MHz, $CDCl_3$) of Compound **14**

20160425-MI-5-63A.43.fid
MI-5-63A

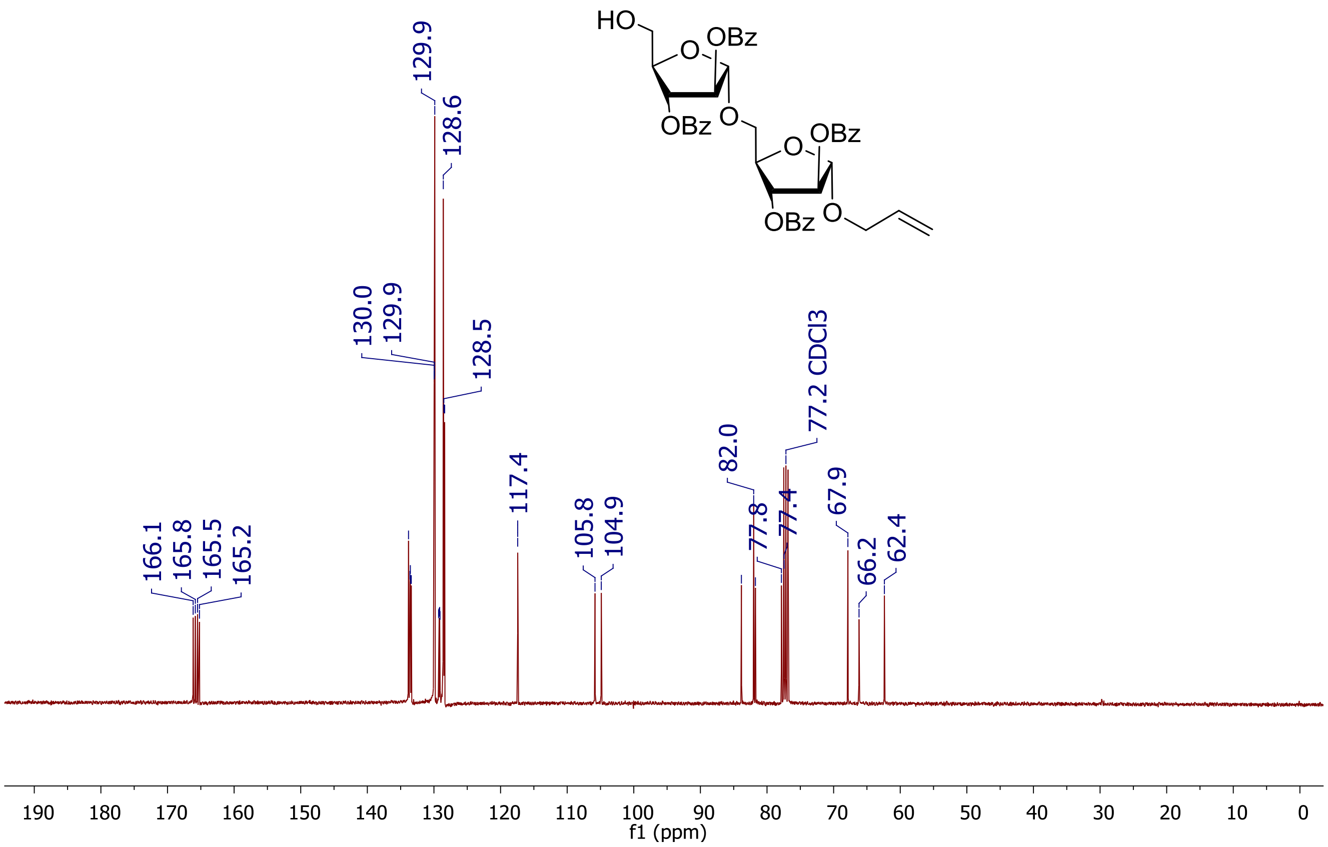

DEPT Spectrum (100.53 MHz, $CDCl_3$) of Compound **14**

20160425-MI-5-63A.44.fid
MI-5-63A

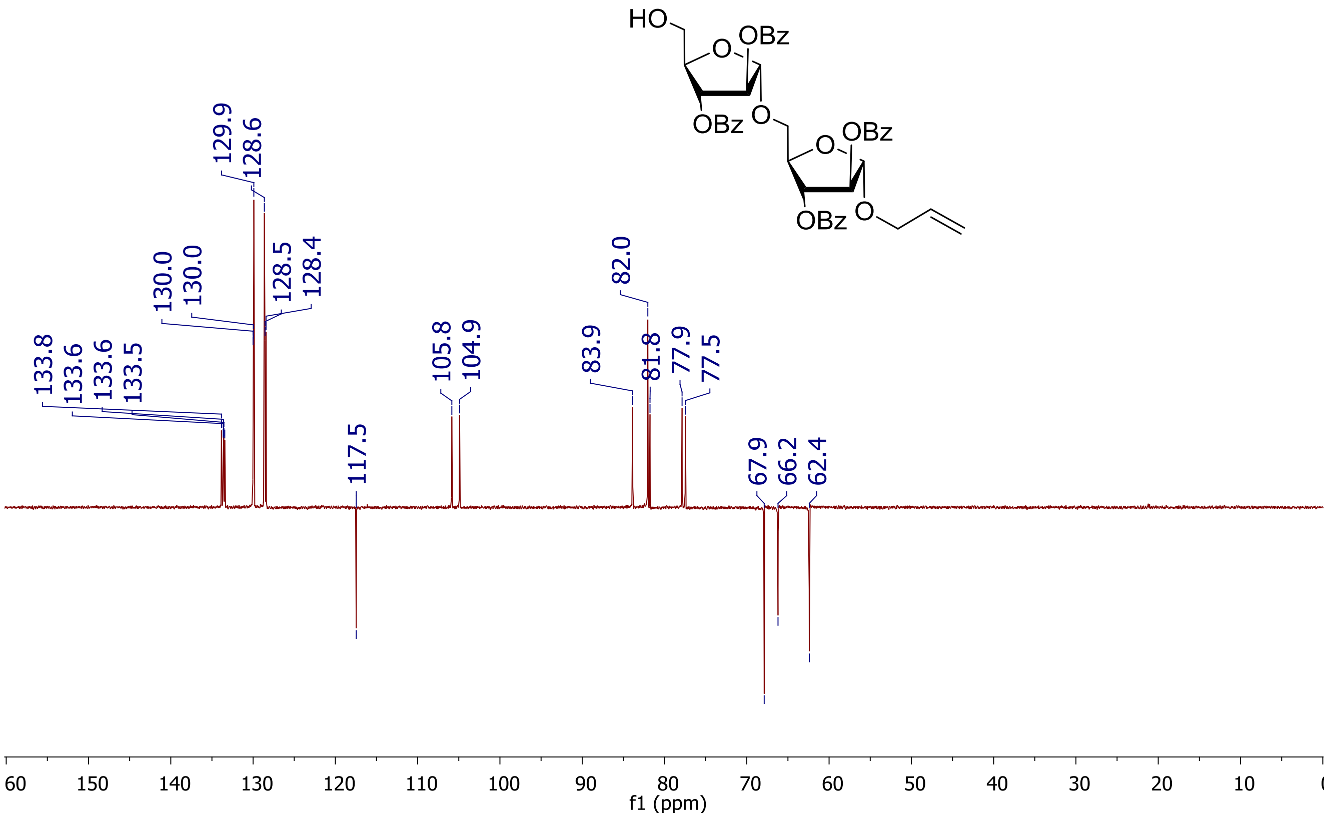

^1H NMR Spectrum (399.78 MHz, $CDCl_3$) of Compound **15a**

20160328-MI-5-78/45
MI-5-78

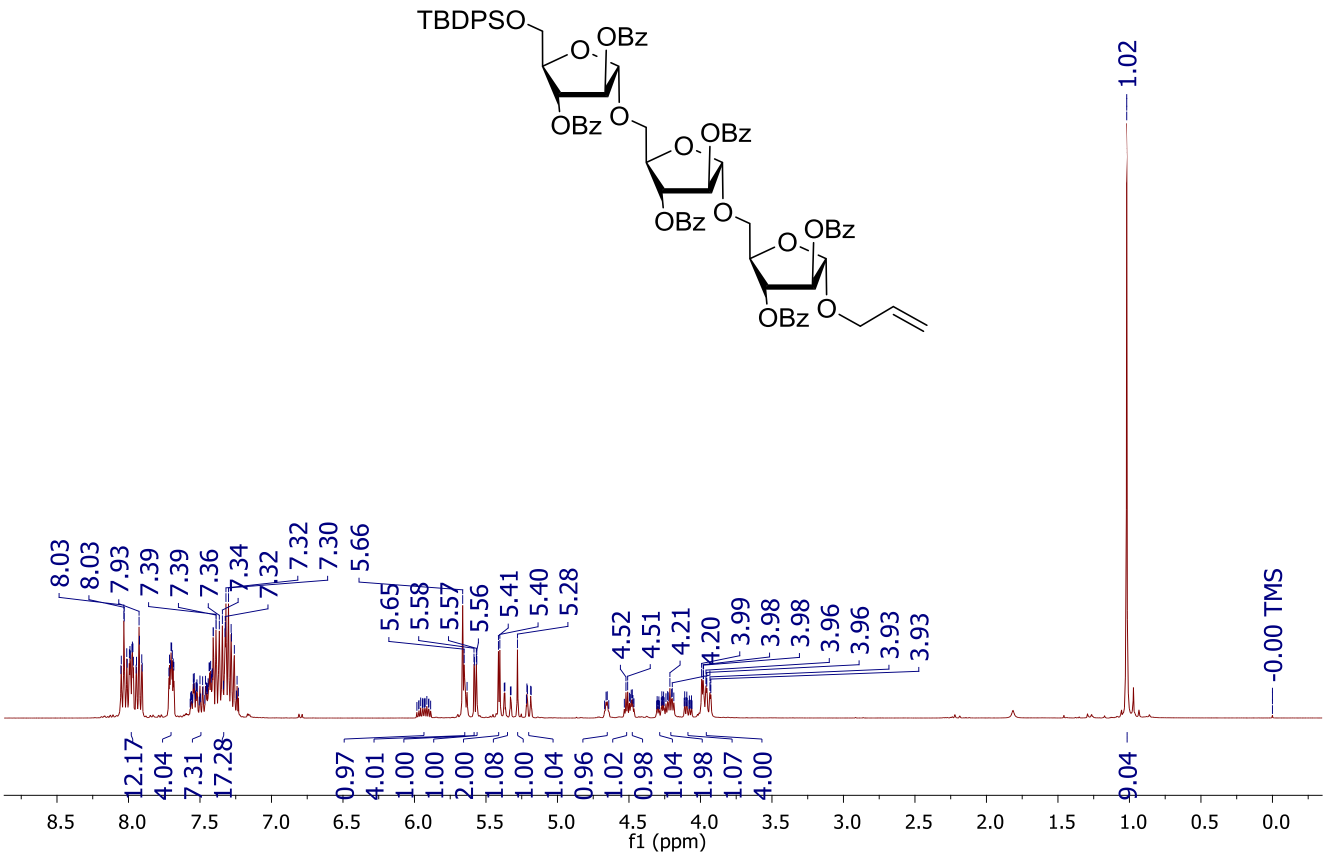

^{13}C NMR Spectrum (100.53 MHz, $CDCl_3$) of Compound **15a**

20160328-MI-5-78/47
MI-5-78

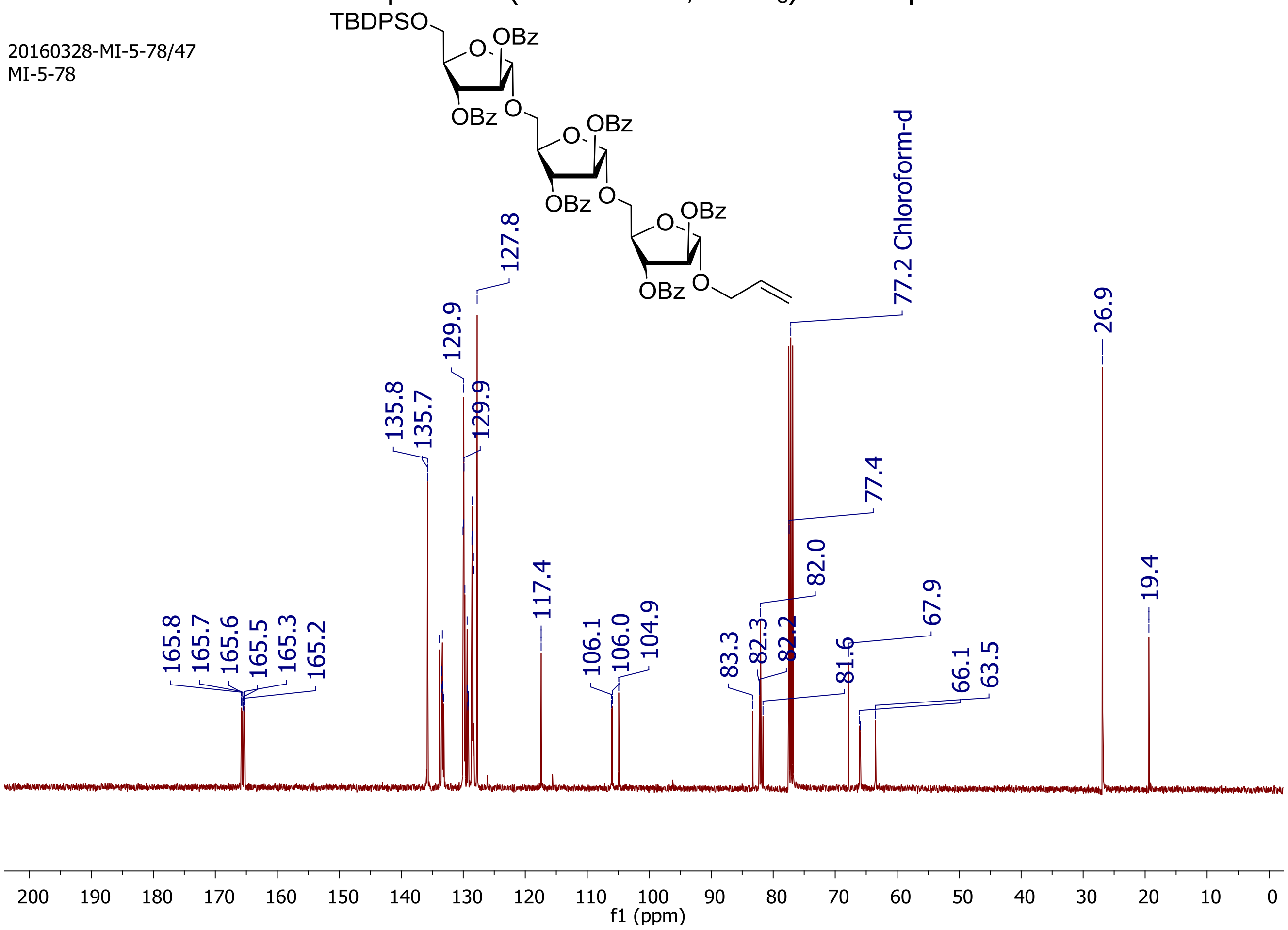

DEPT Spectrum (100.53 MHz, $CDCl_3$) of Compound **15a**

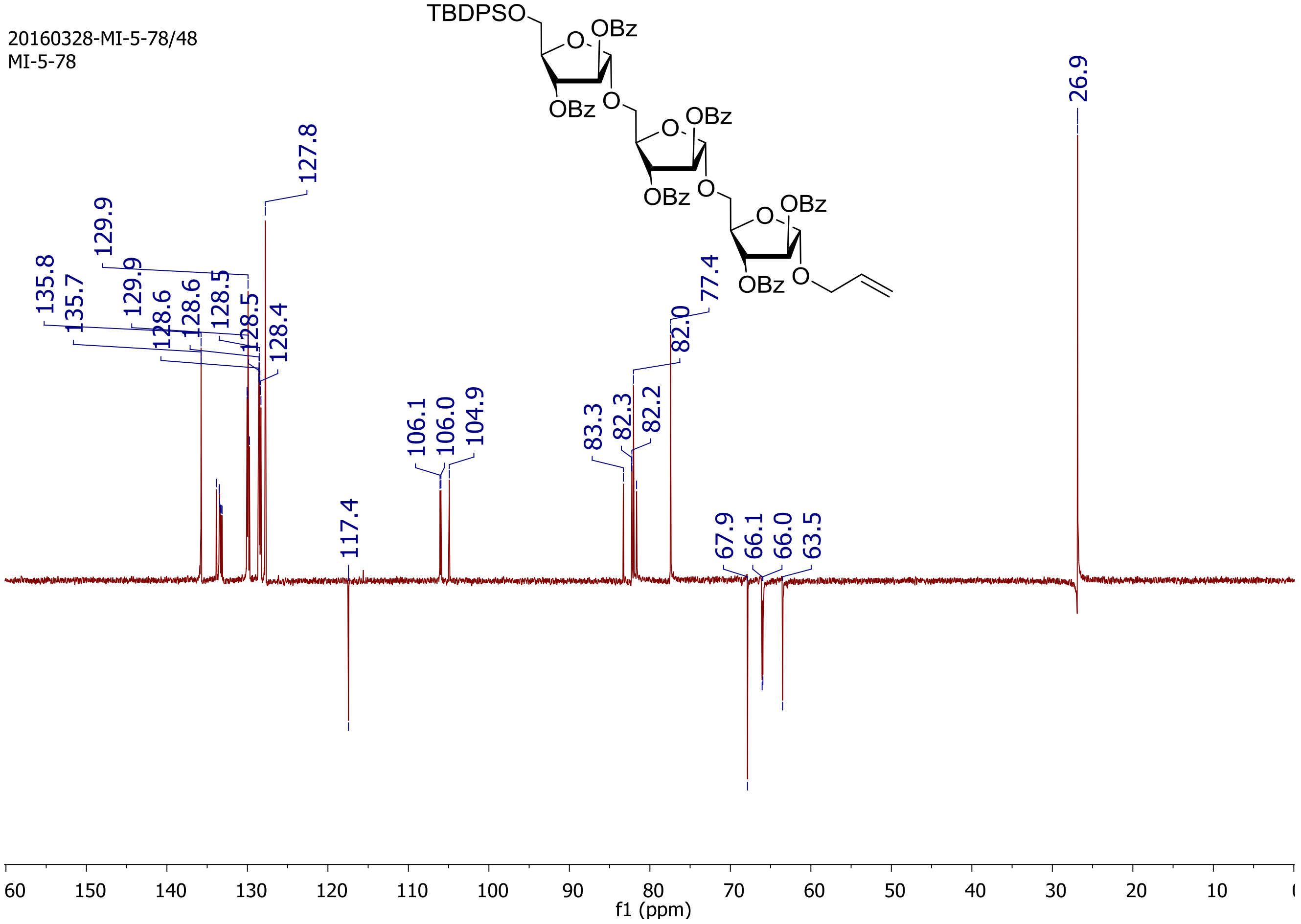

^{1}H NMR Spectrum (399.78 MHz, $CDCl_3$) of Compound **15b**

20160328-MI-5-79/38
MI-5-79

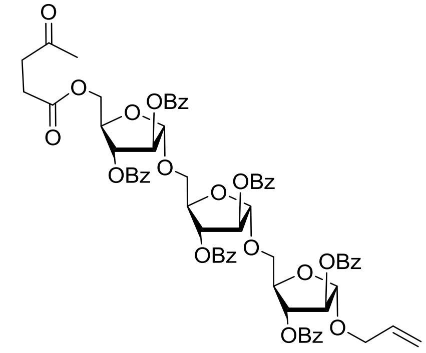

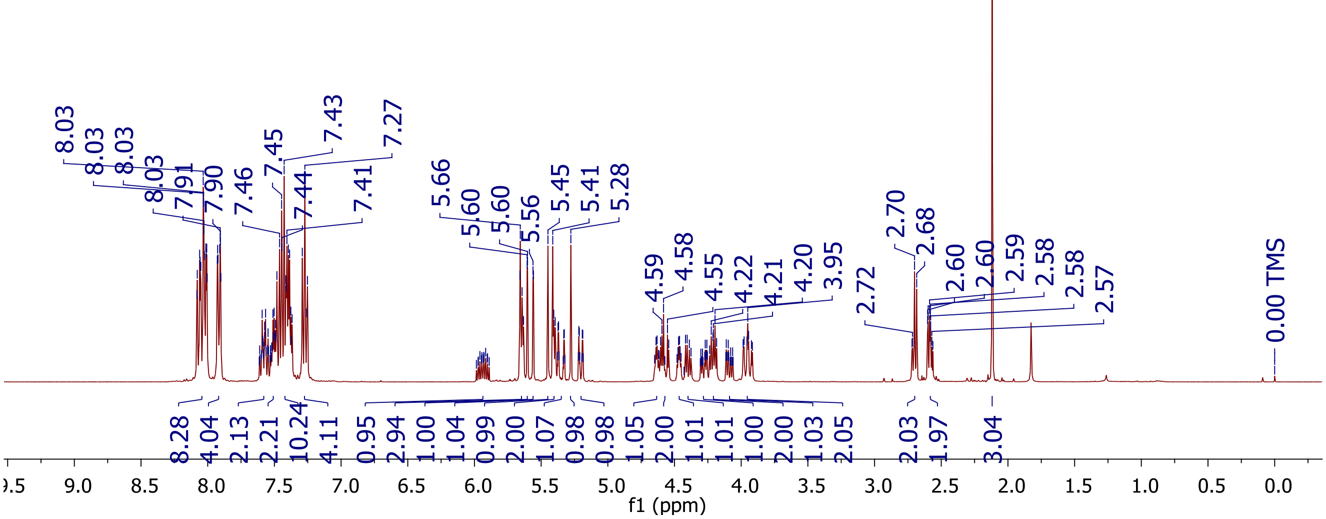

^{13}C NMR Spectrum (100.53 MHz, $CDCl_3$) of Compound **15b**

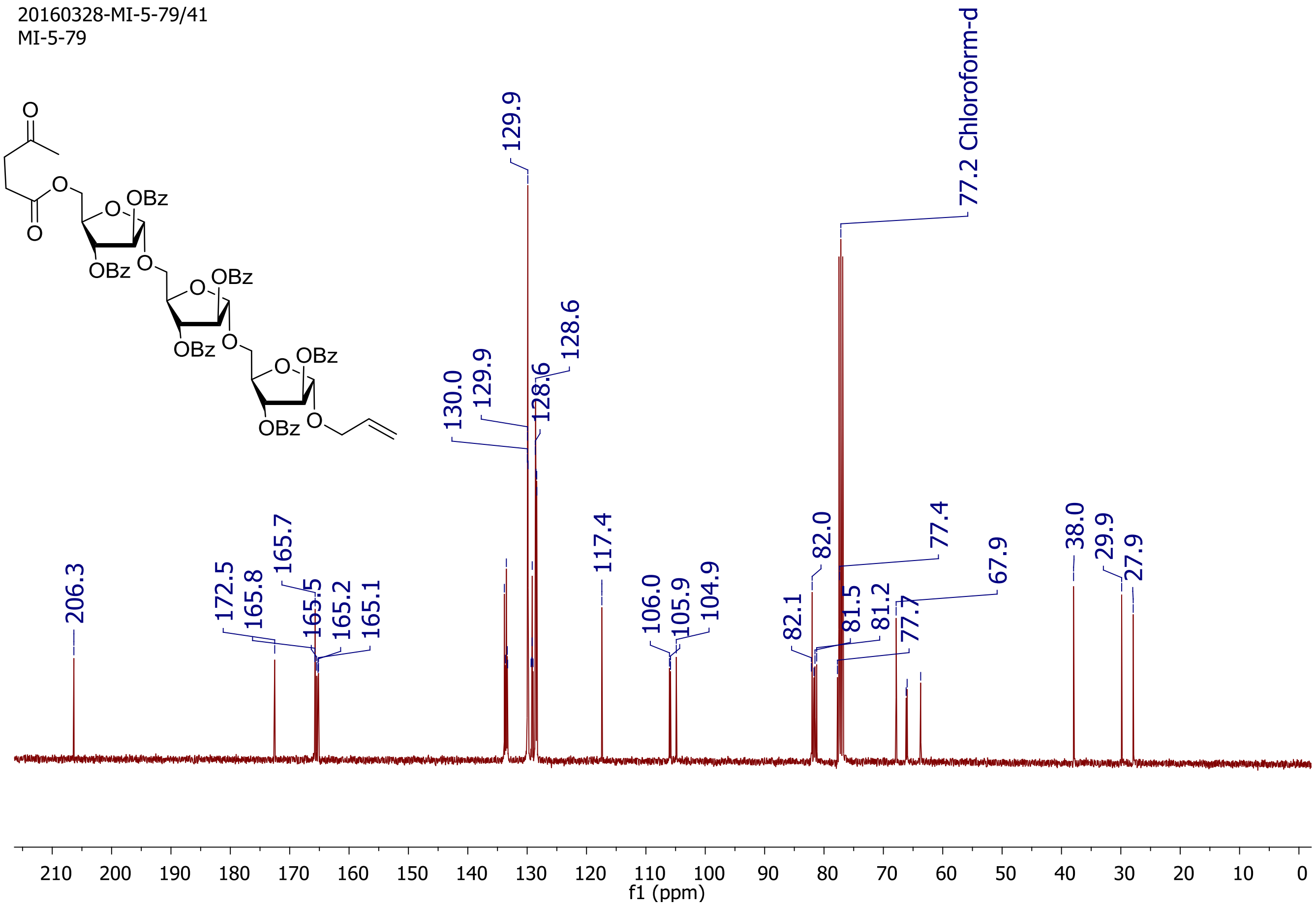

DEPT Spectrum (100.53 MHz, $CDCl_3$) of Compound **15b**

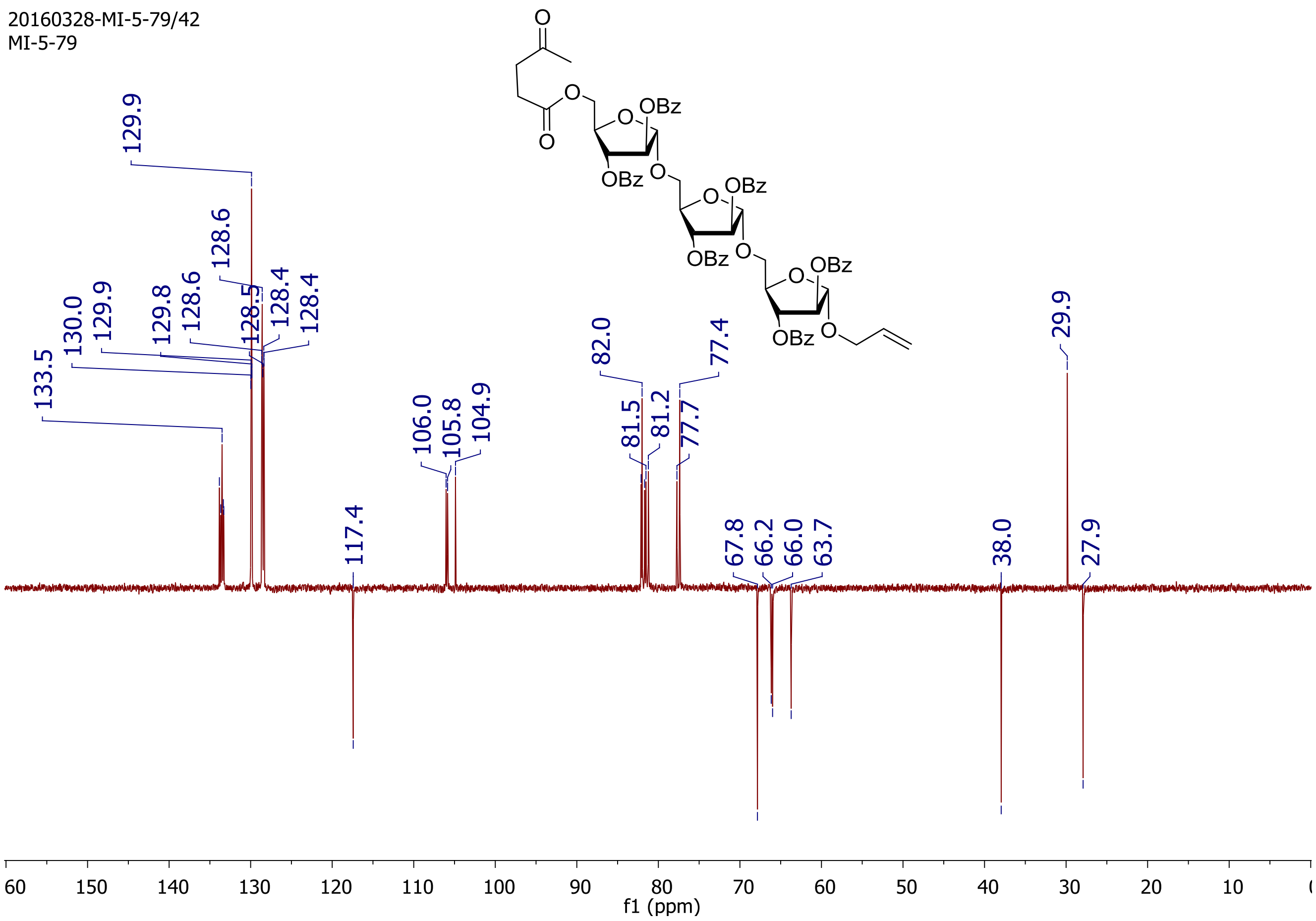

^{1}H NMR Spectrum (399.78 MHz, $CDCl_3$) of Compound **5a**

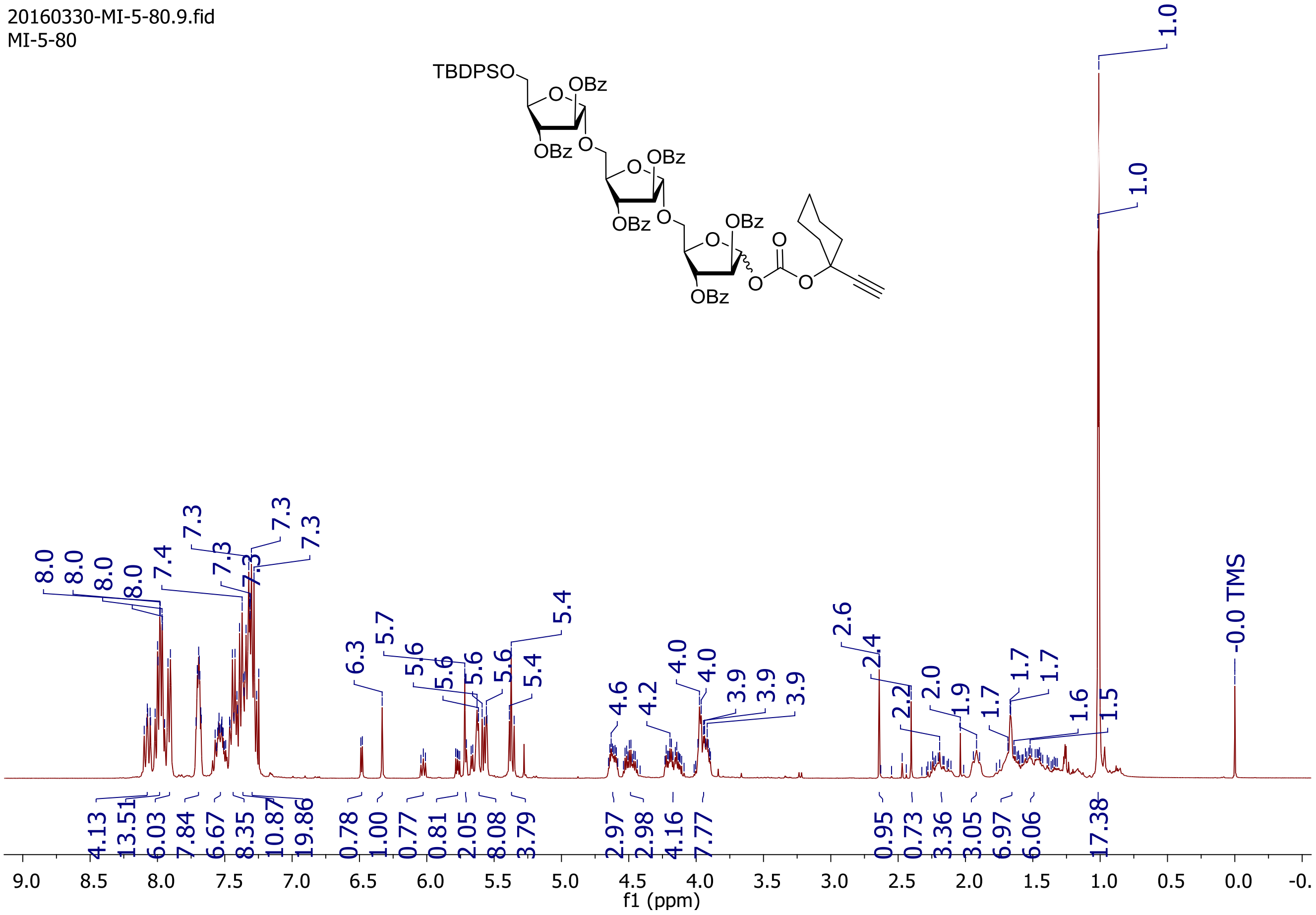

^{13}C NMR Spectrum (100.53 MHz, $CDCl_3$) of Compound **5a**

20160330-MI-5-80.11.fid
MI-5-80

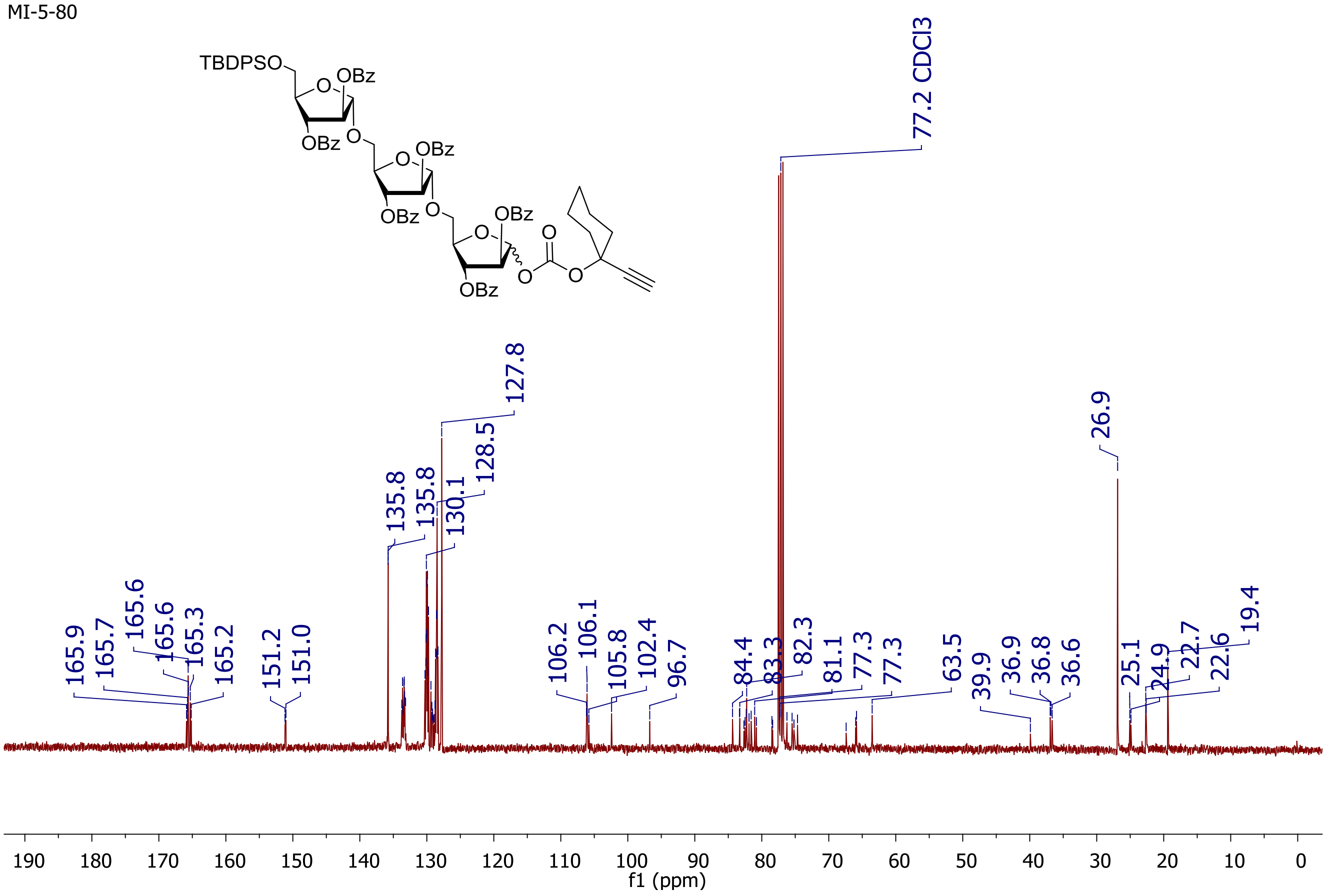

DEPT Spectrum (100.53 MHz, $CDCl_3$) of Compound **5a**

20160330-MI-5-80.12.fid
MI-5-80

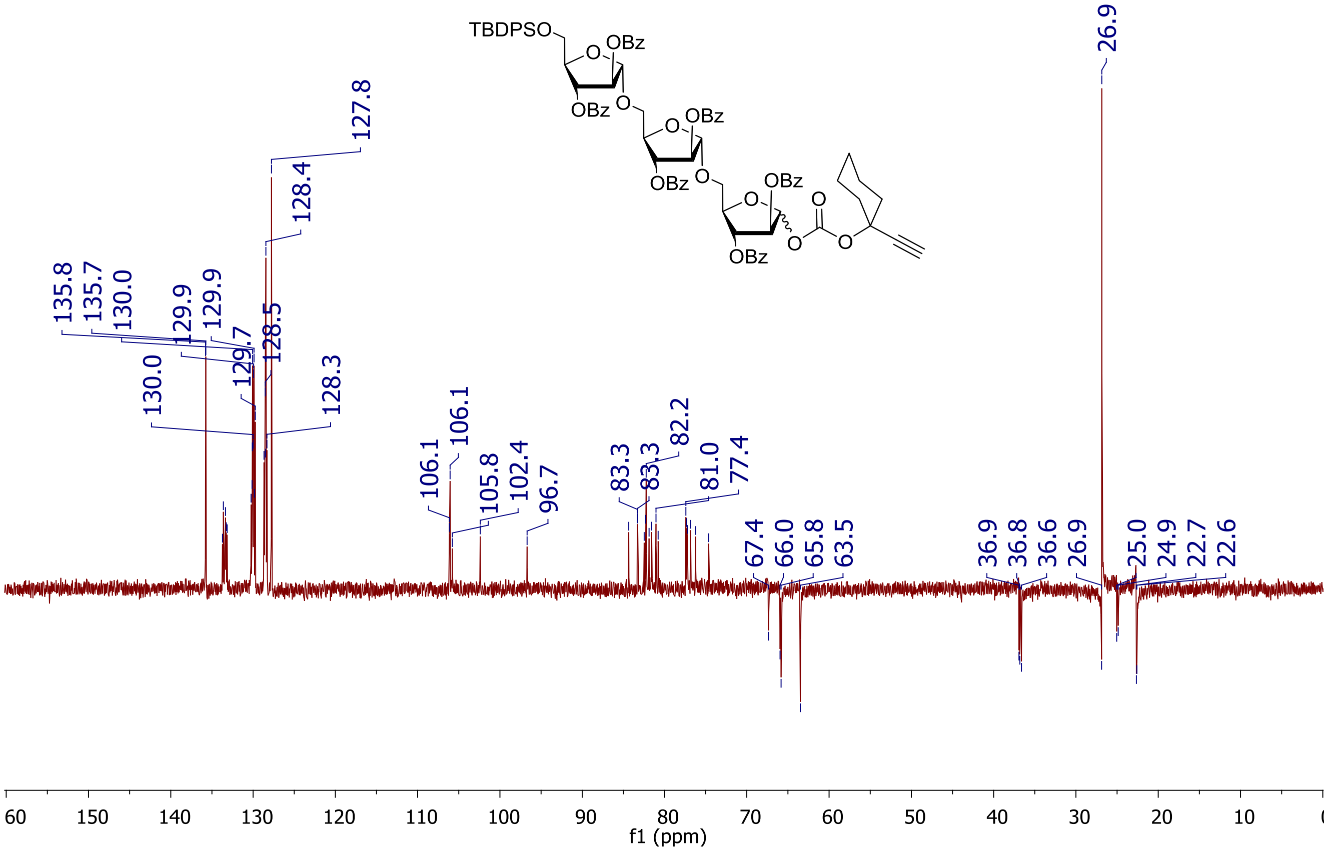

^{1}H NMR Spectrum (399.78 MHz, $CDCl_3$) of Compound **5b**

20160426-MI-5-81.1.fid
MI-5-81

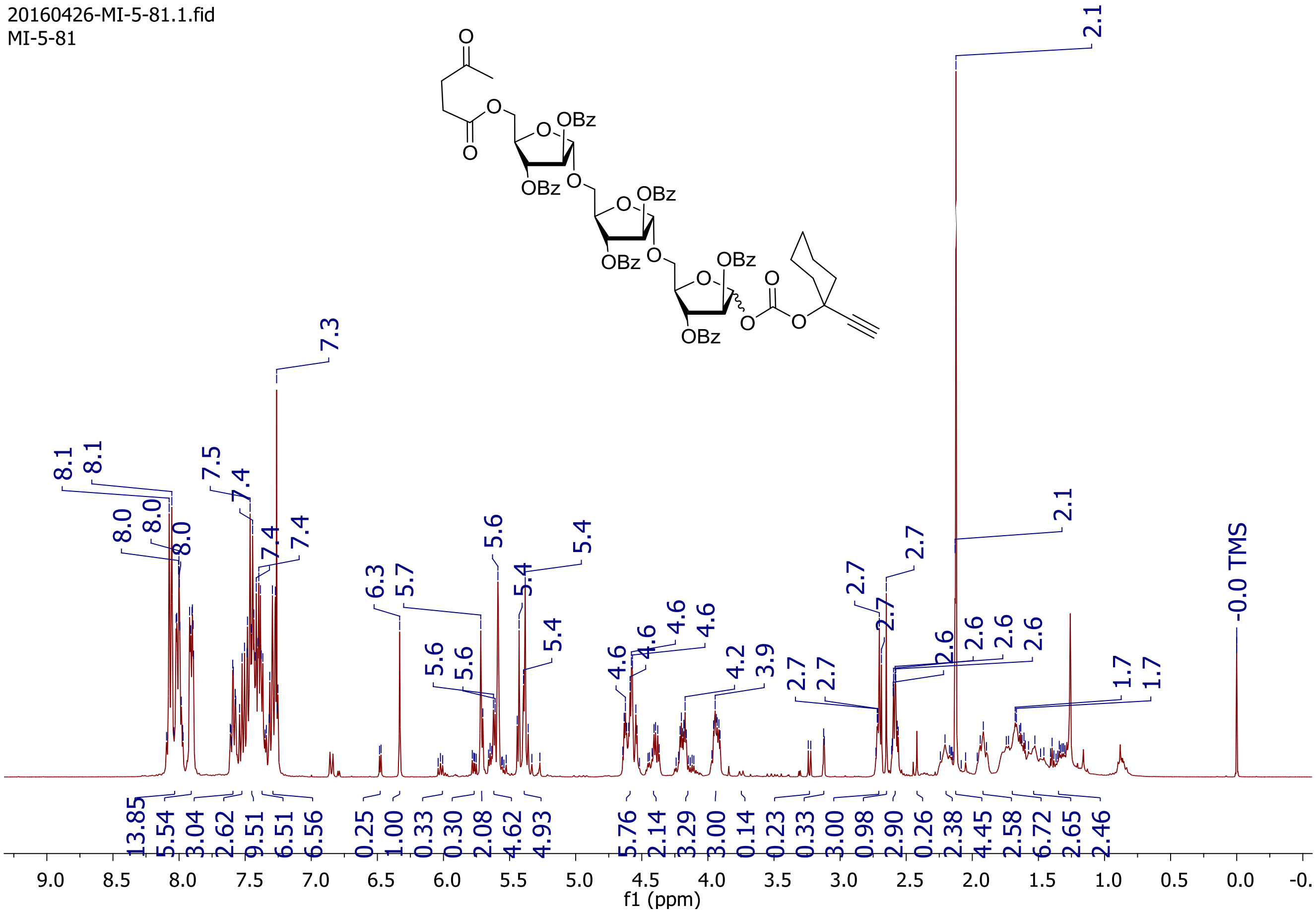

^{13}C NMR Spectrum (100.53 MHz, $CDCl_3$) of Compound **5b**

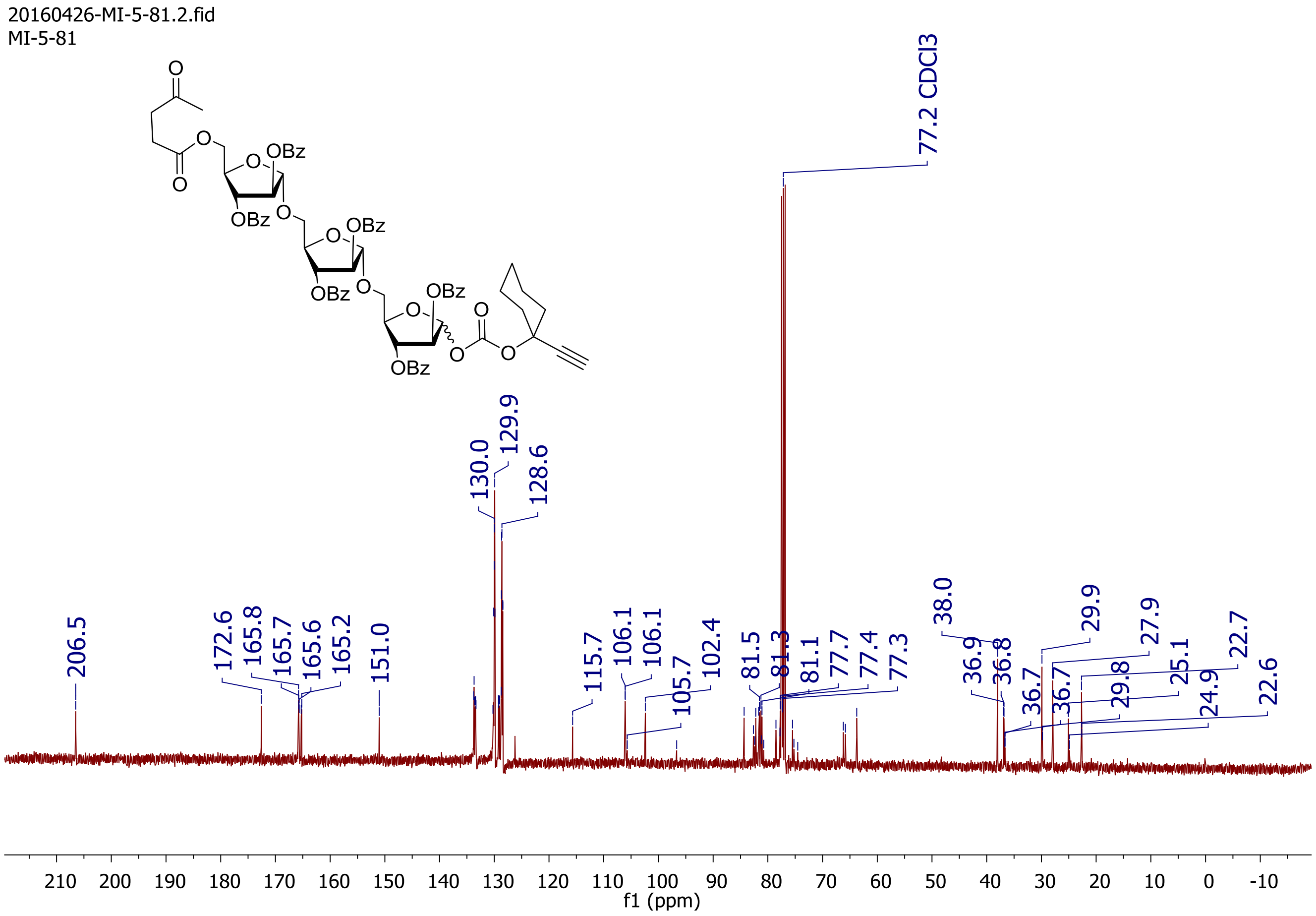

DEPT Spectrum (100.53 MHz, $CDCl_3$) of Compound **5b**

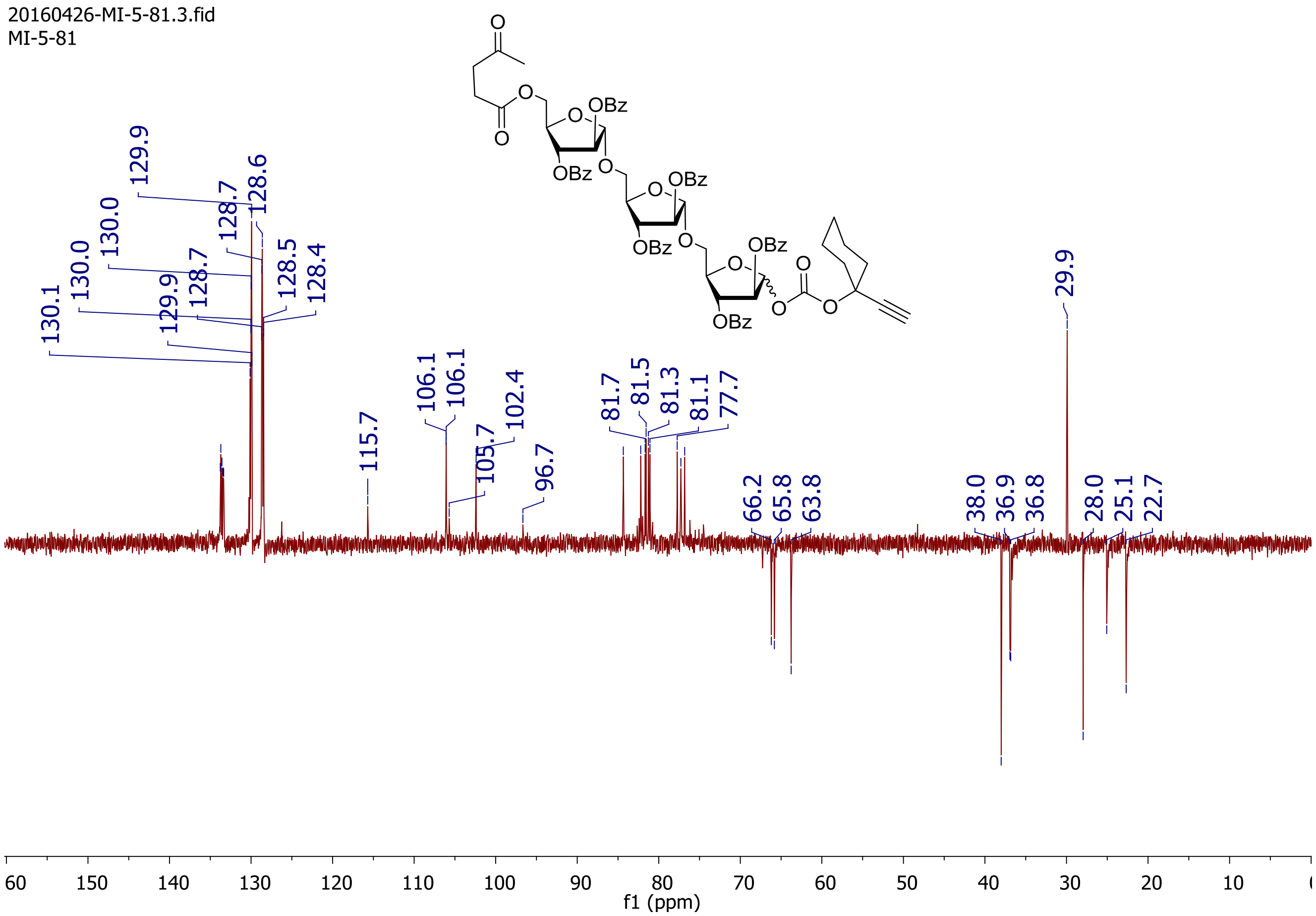

^{1}H NMR Spectrum (399.78 MHz, $CDCl_3$) of Compound **17**

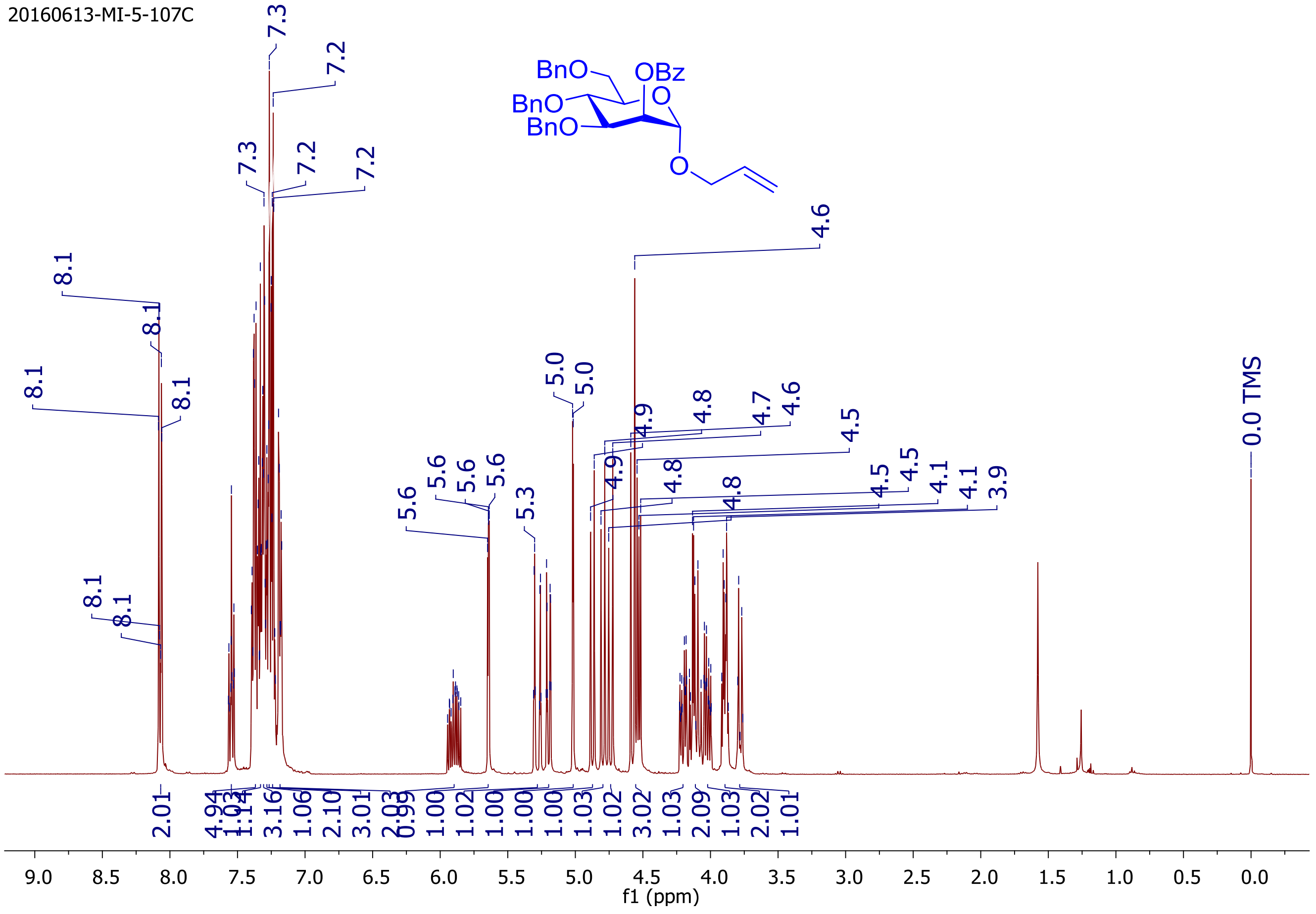

^{13}C NMR Spectrum (100.53 MHz, $CDCl_3$) of Compound **17**

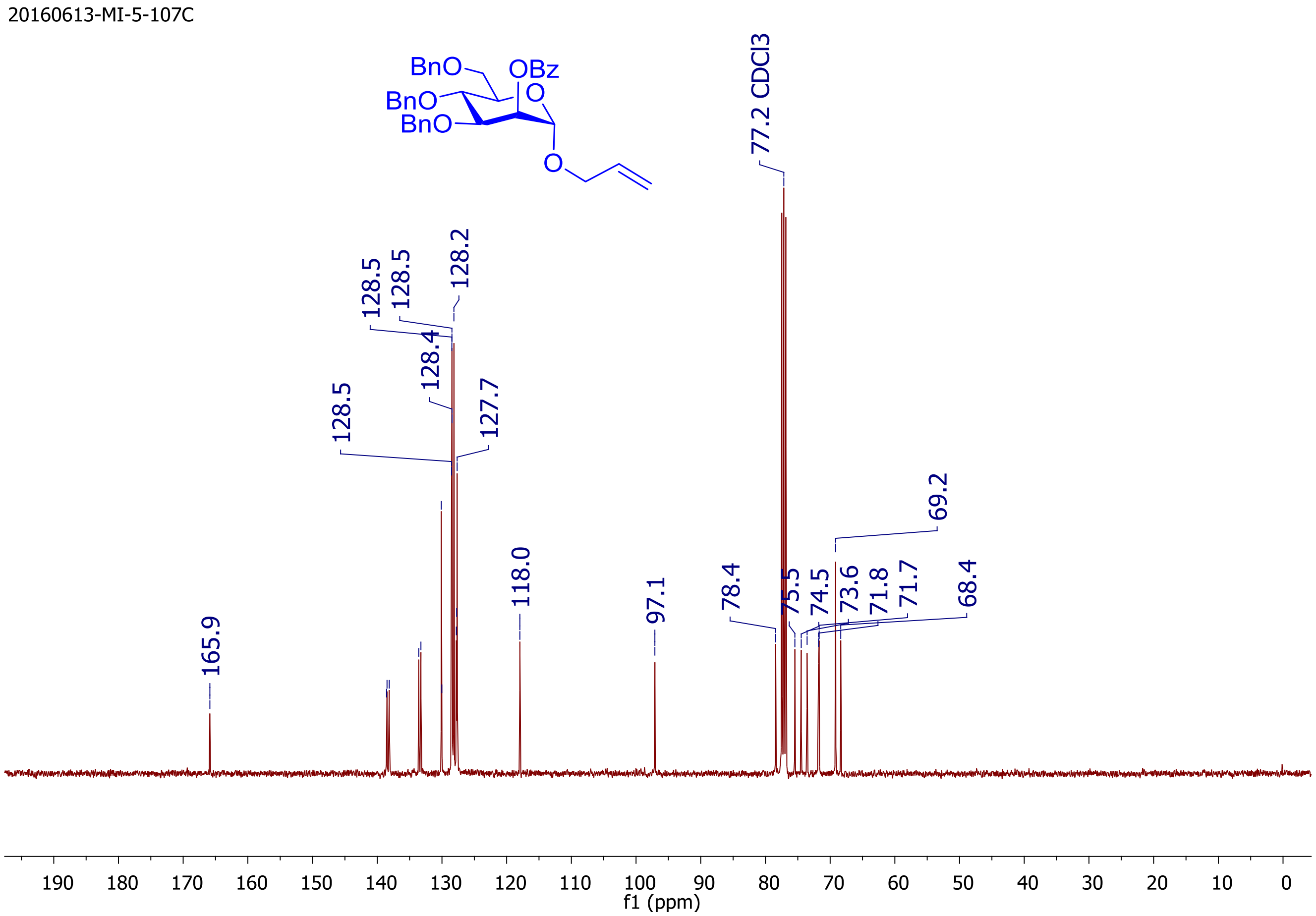

DEPT Spectrum (100.53 MHz, $CDCl_3$) of Compound **17**

20160613-MI-5-107C

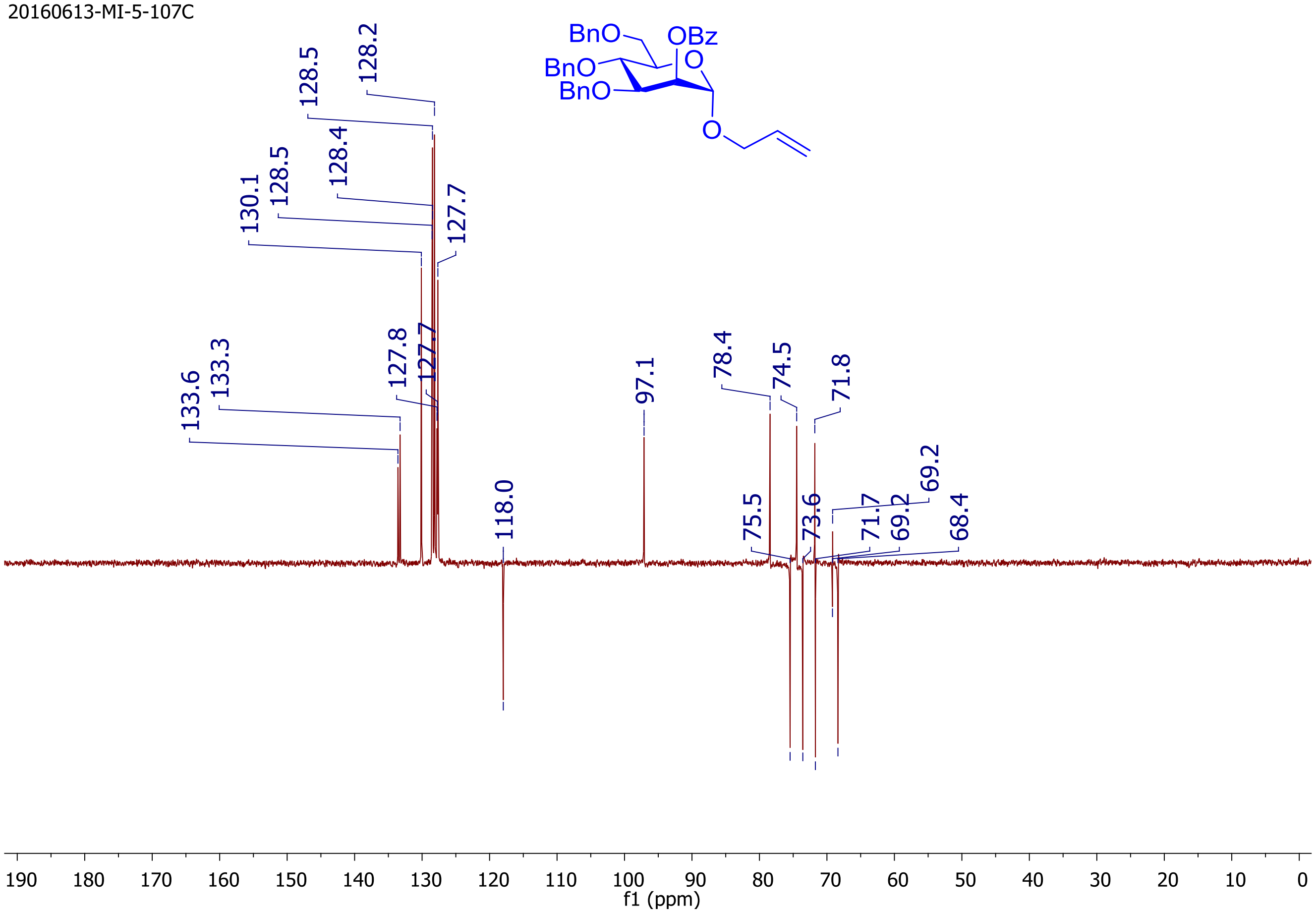

^1H NMR Spectrum (399.78 MHz, $CDCl_3$) of Compound **18**

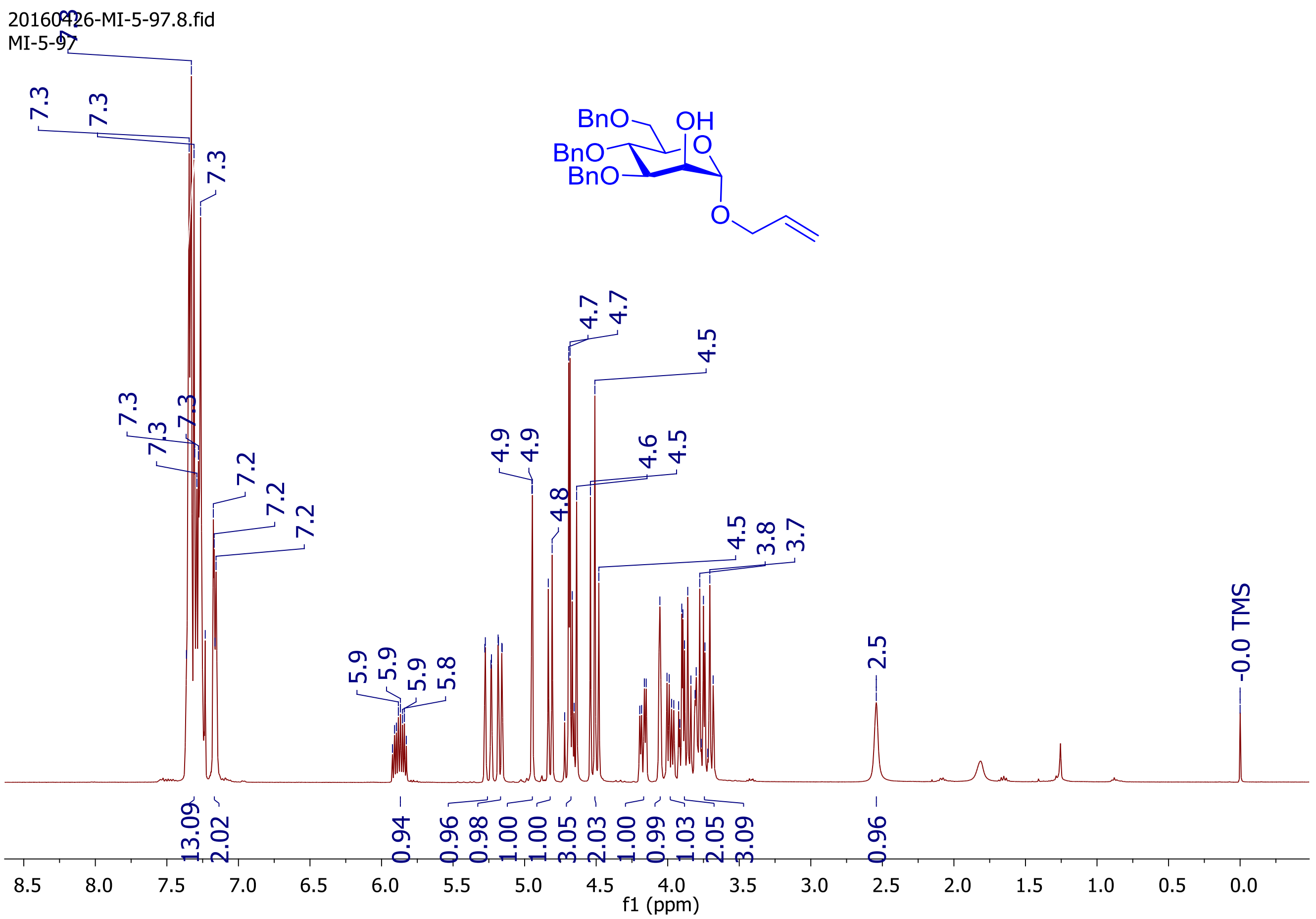

^{13}C NMR Spectrum (100.53 MHz, $CDCl_3$) of Compound **18**

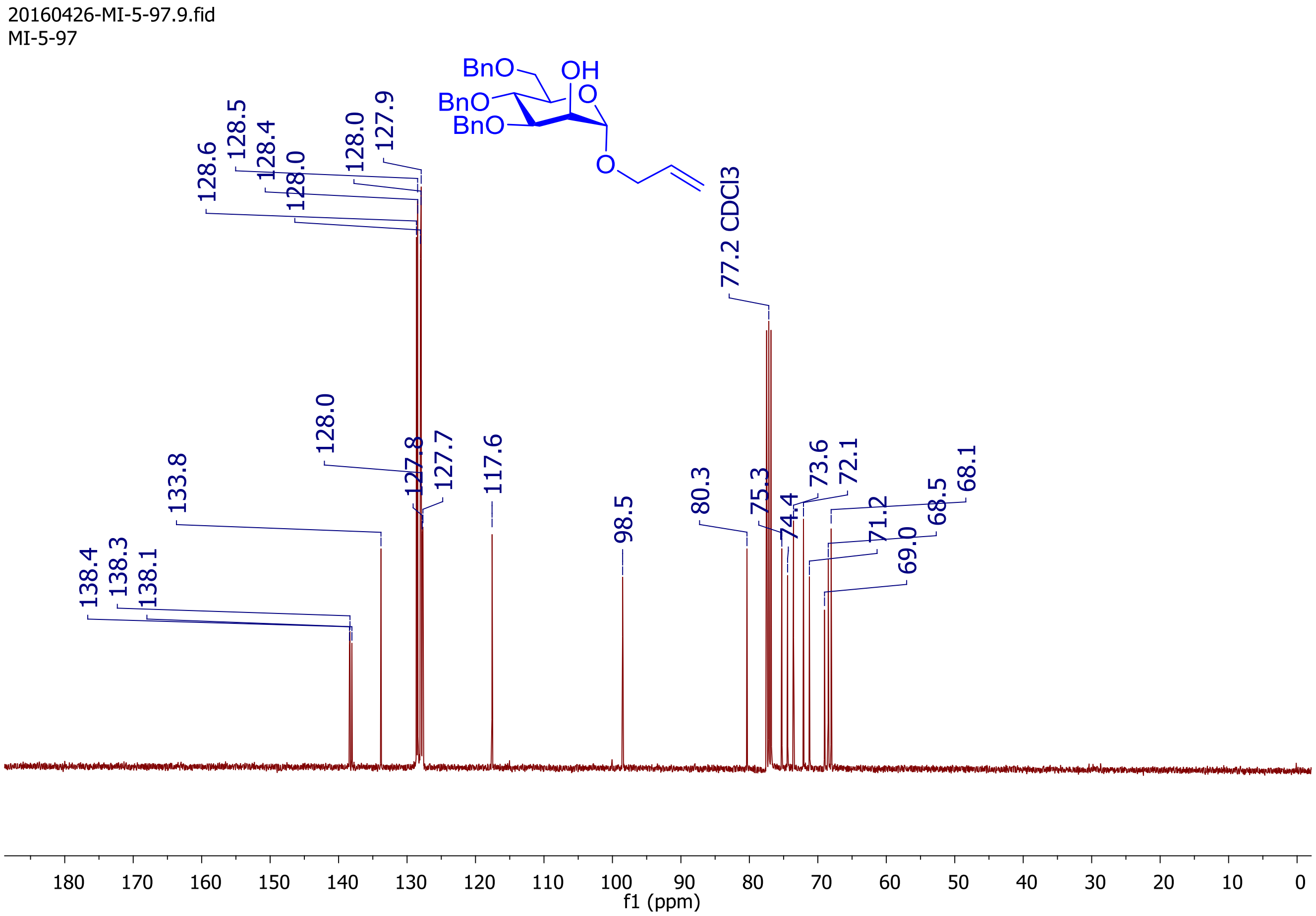

DEPT Spectrum (100.53 MHz, $CDCl_3$) of Compound **18**

20160426-MI-5-97.10.fid
MI-5-97

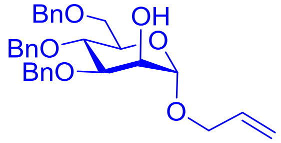

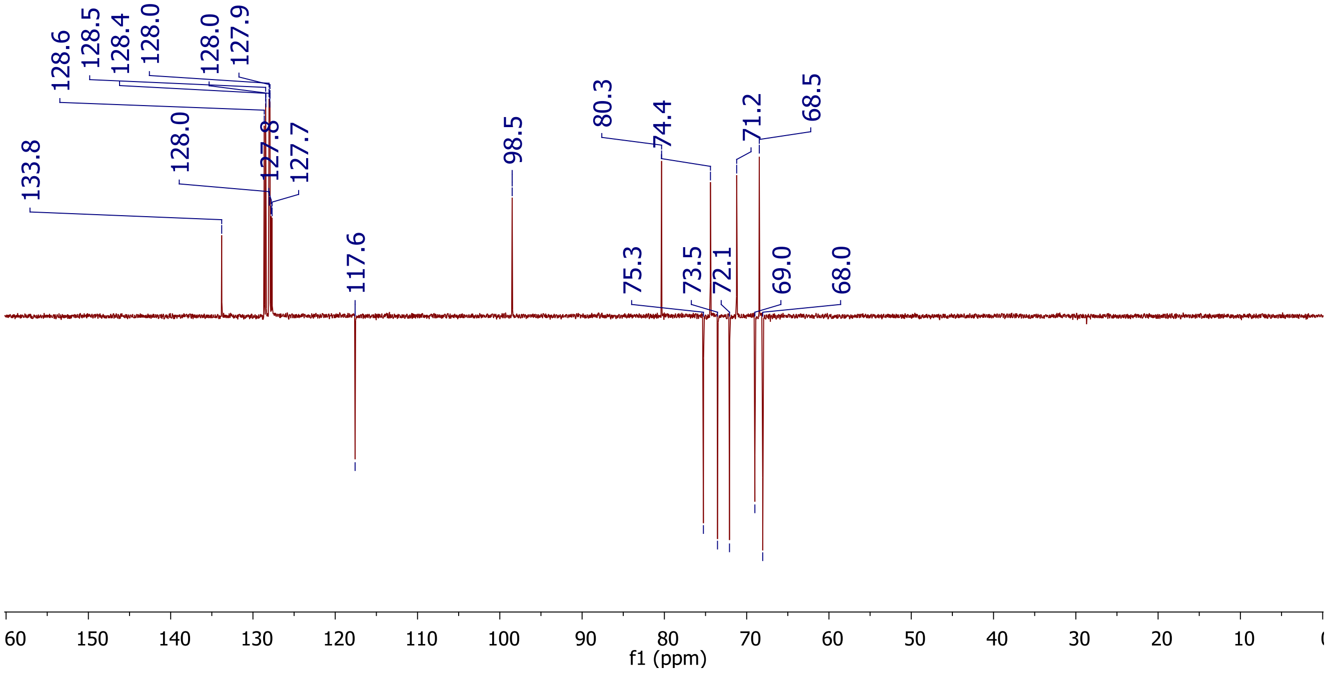

^{1}H NMR Spectrum (399.78 MHz, $CDCl_3$) of Compound **19**

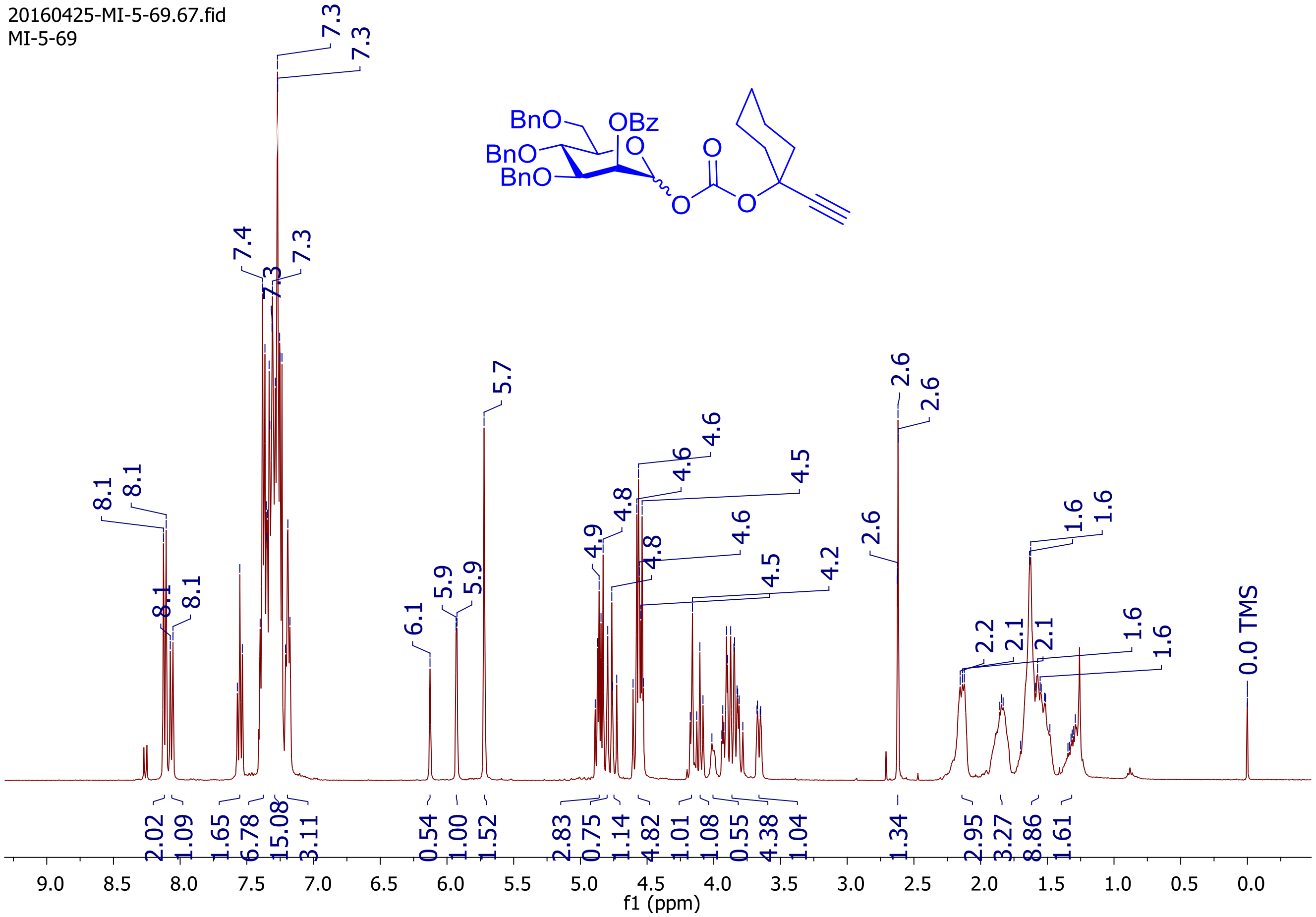

^{13}C NMR Spectrum (100.53 MHz, $CDCl_3$) of Compound **19**

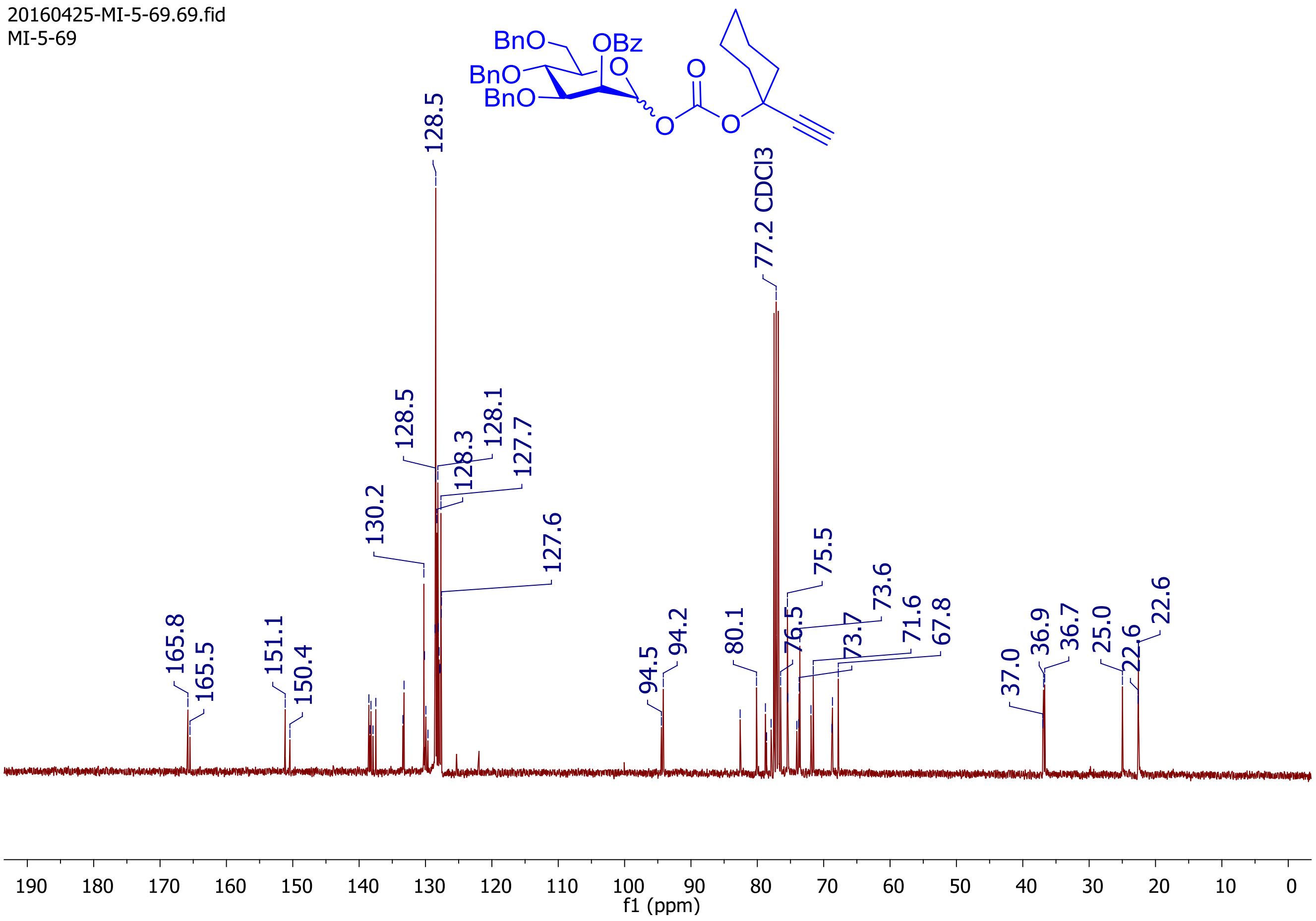

DEPT Spectrum (100.53 MHz, $CDCl_3$) of Compound **19**

20160425-MI-5-69.70.fid
MI-5-69

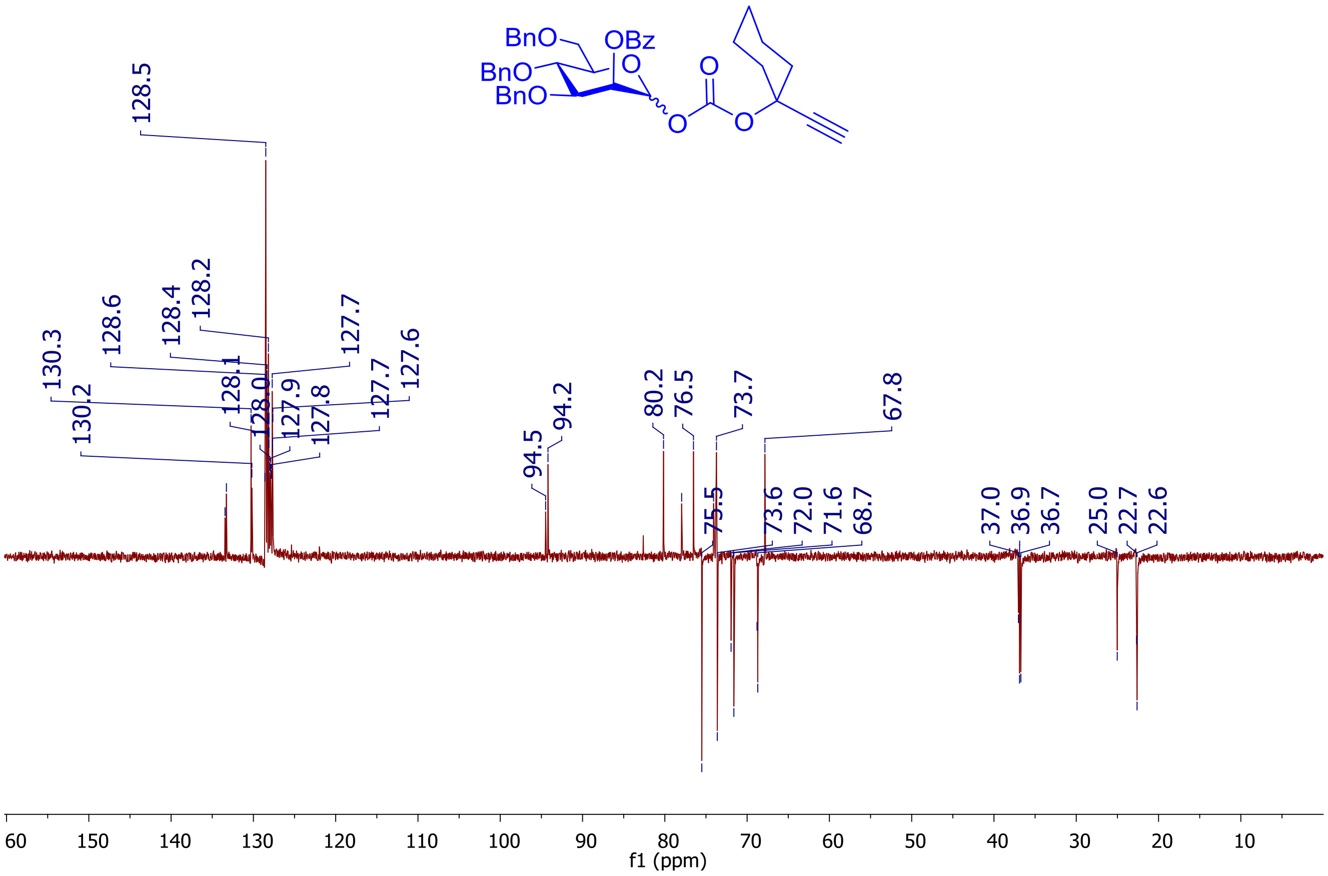

^{1}H NMR Spectrum (399.78 MHz, $CDCl_3$) of Compound **20**

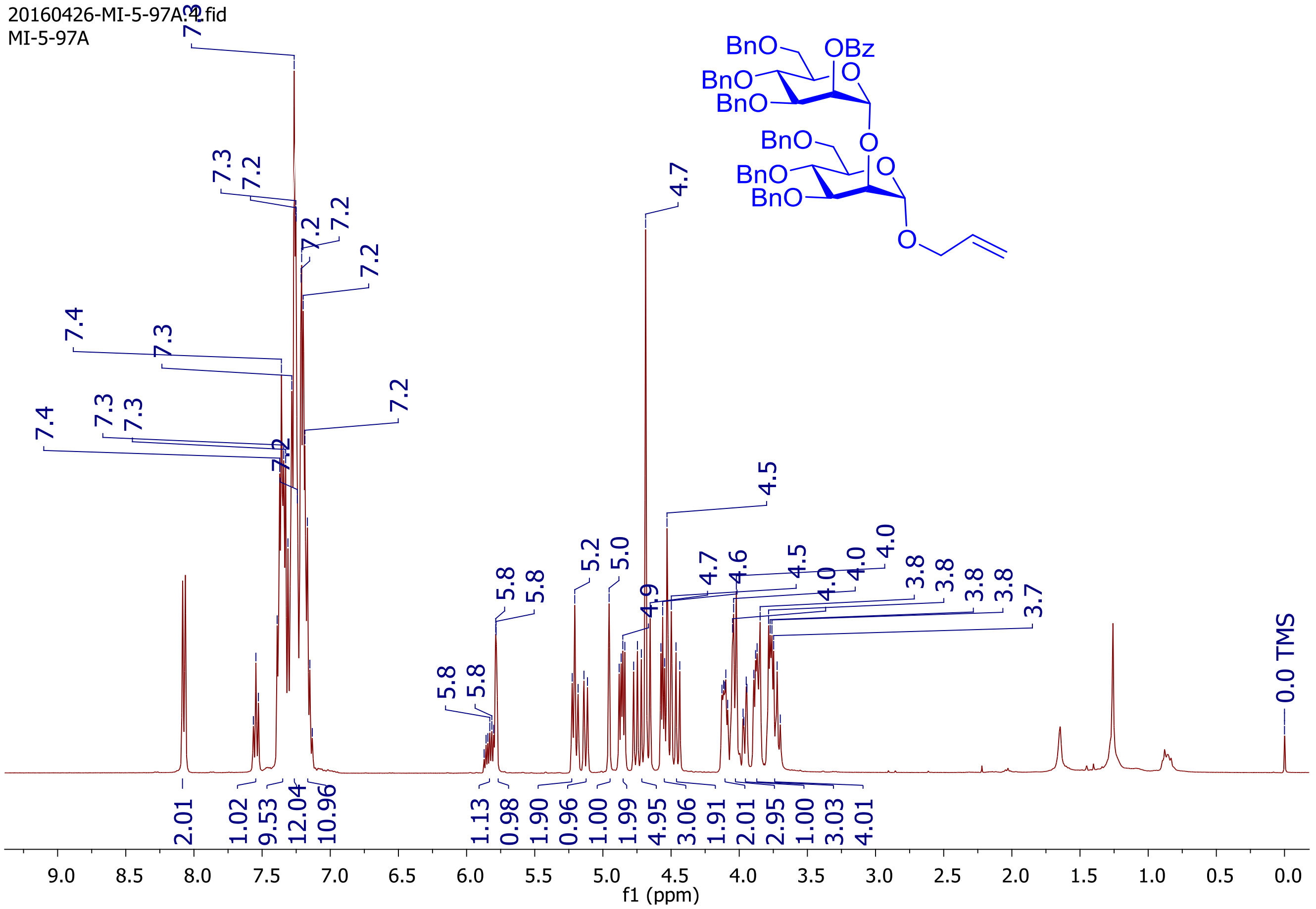

^{13}C NMR Spectrum (100.53 MHz, $CDCl_3$) of Compound **20**

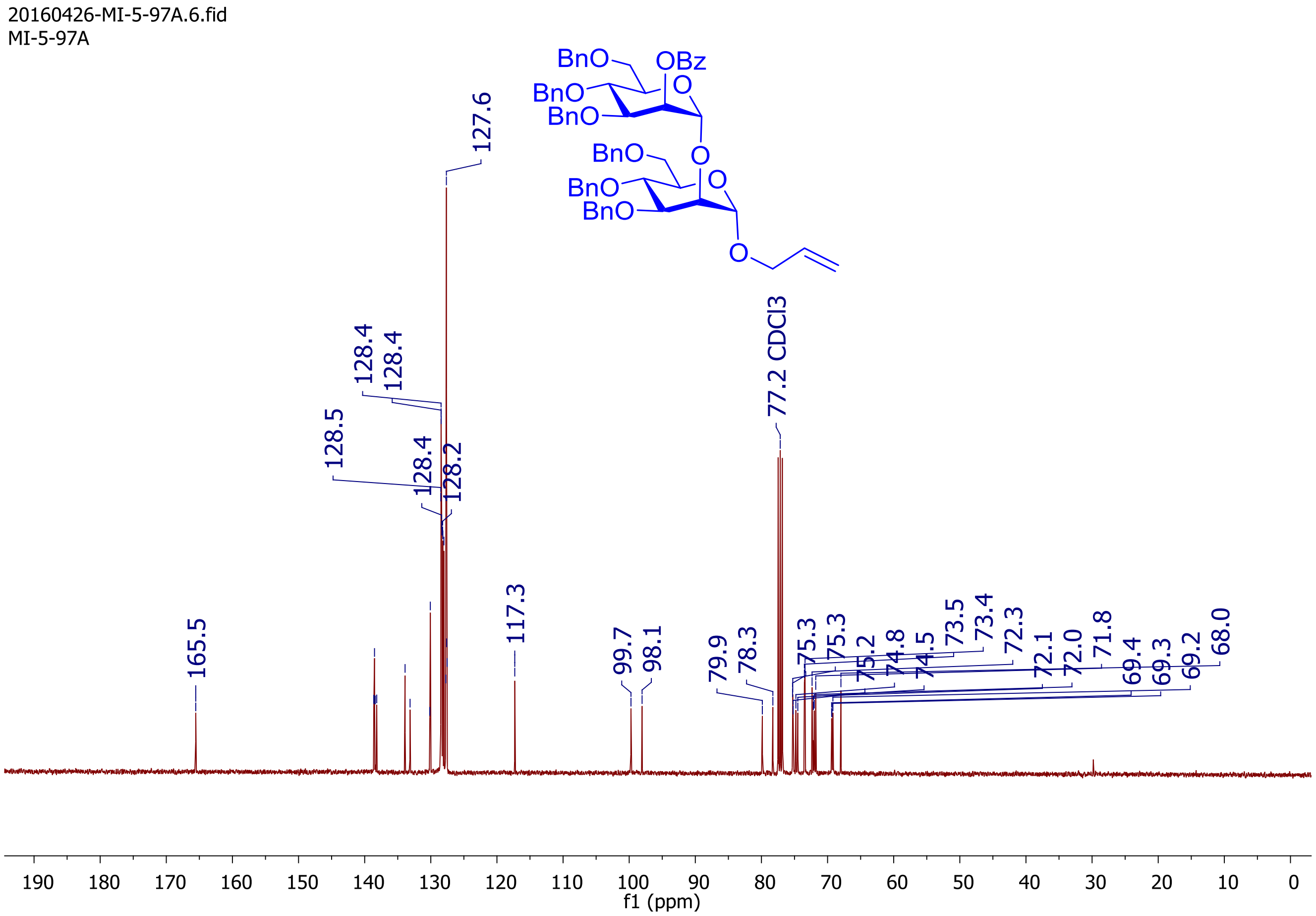

DEPT Spectrum (100.53 MHz, $CDCl_3$) of Compound **20**

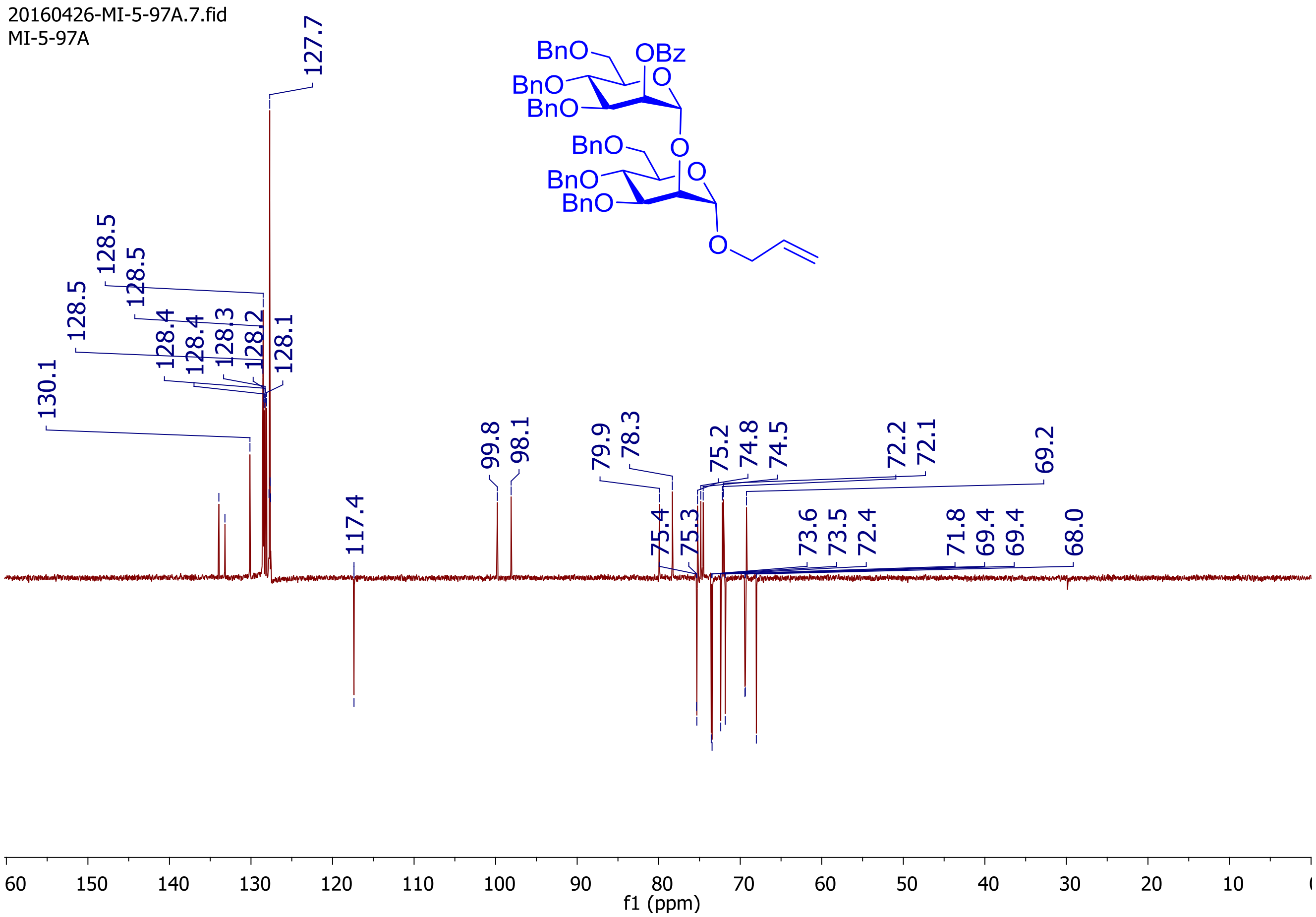

^{1}H NMR Spectrum (399.78 MHz, $CDCl_3$) of Compound **2**

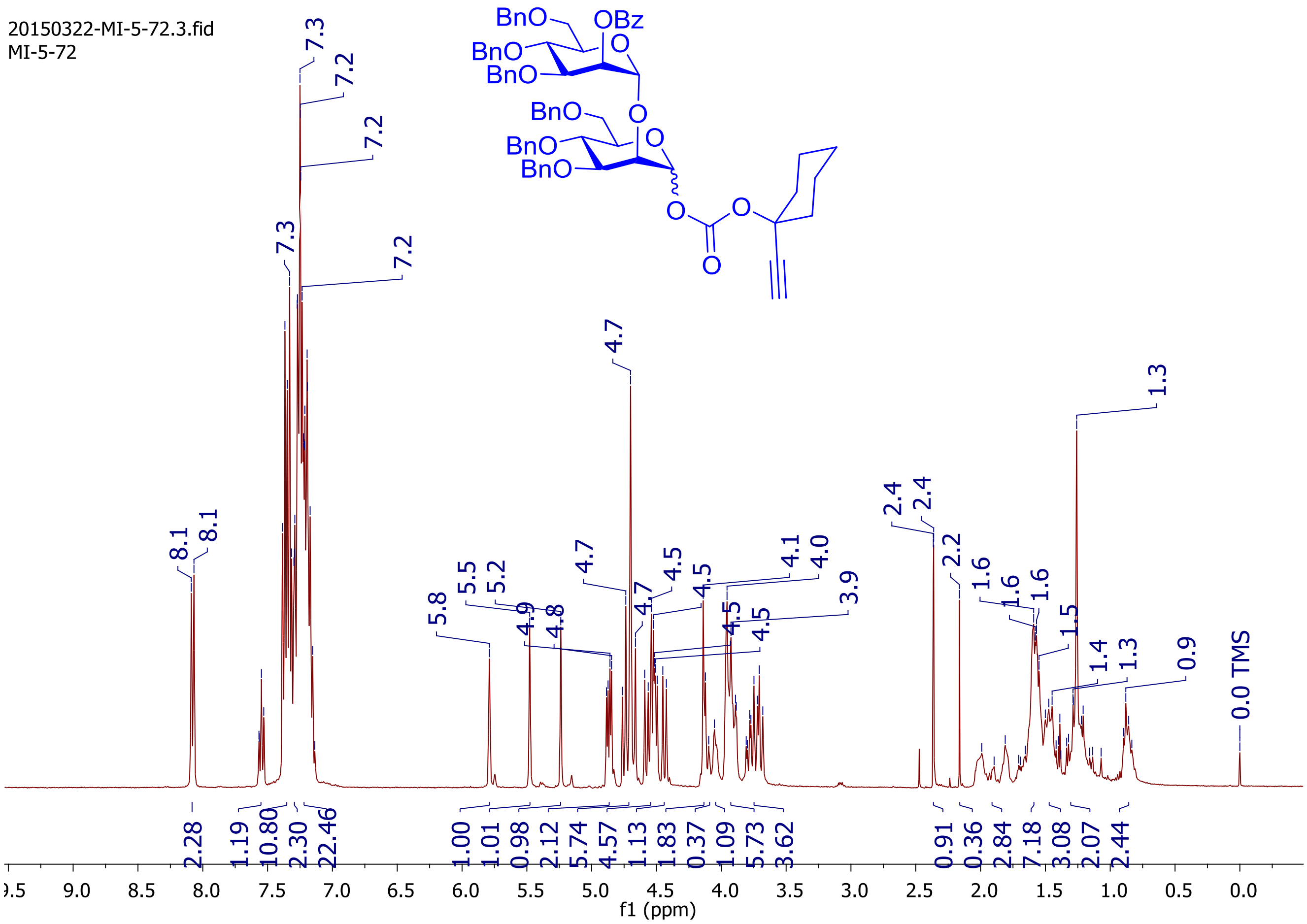

^{13}C NMR Spectrum (100.53 MHz, $CDCl_3$) of Compound **2**

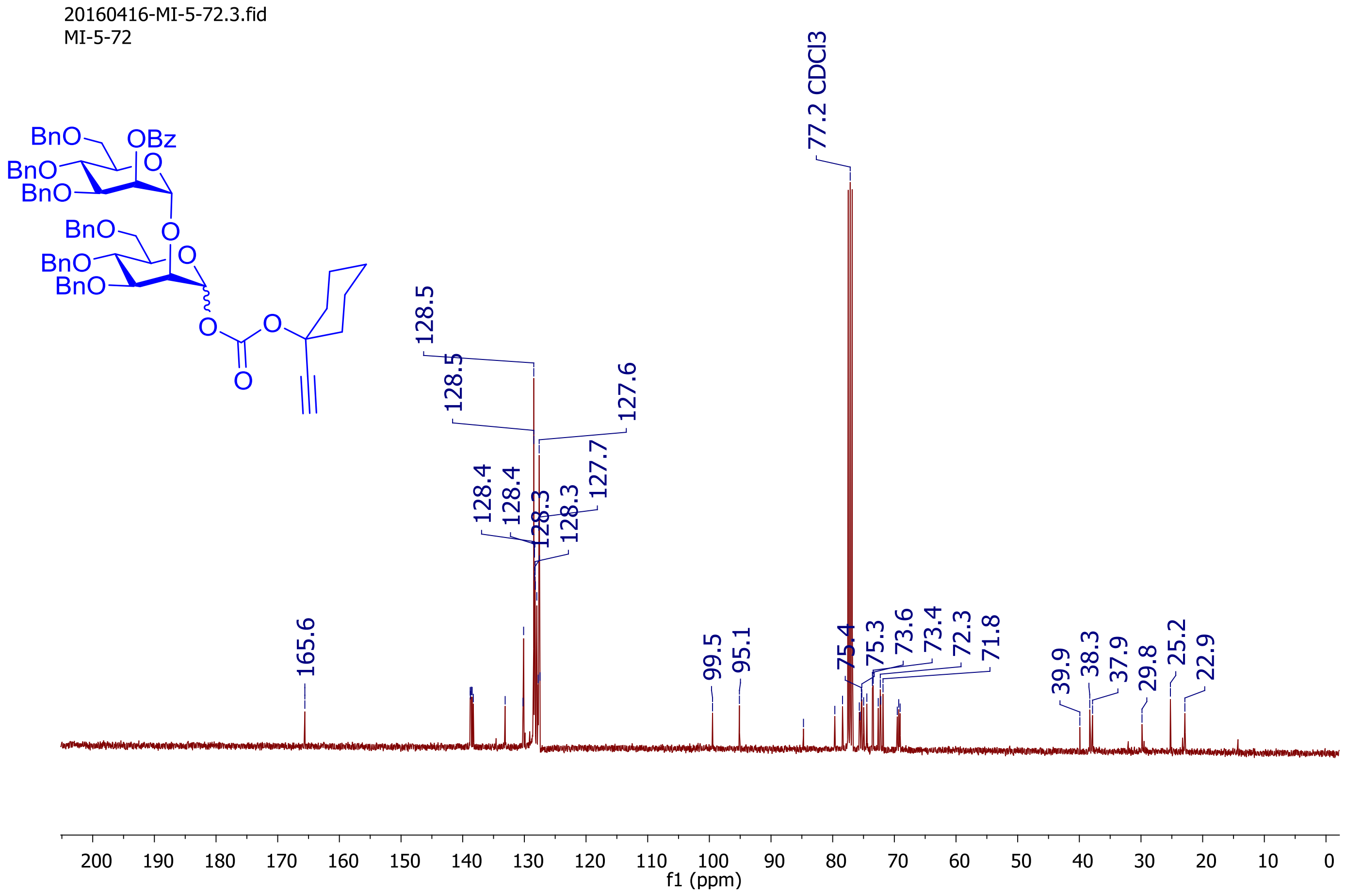

DEPT Spectrum (100.53 MHz, $CDCl_3$) of Compound **2**

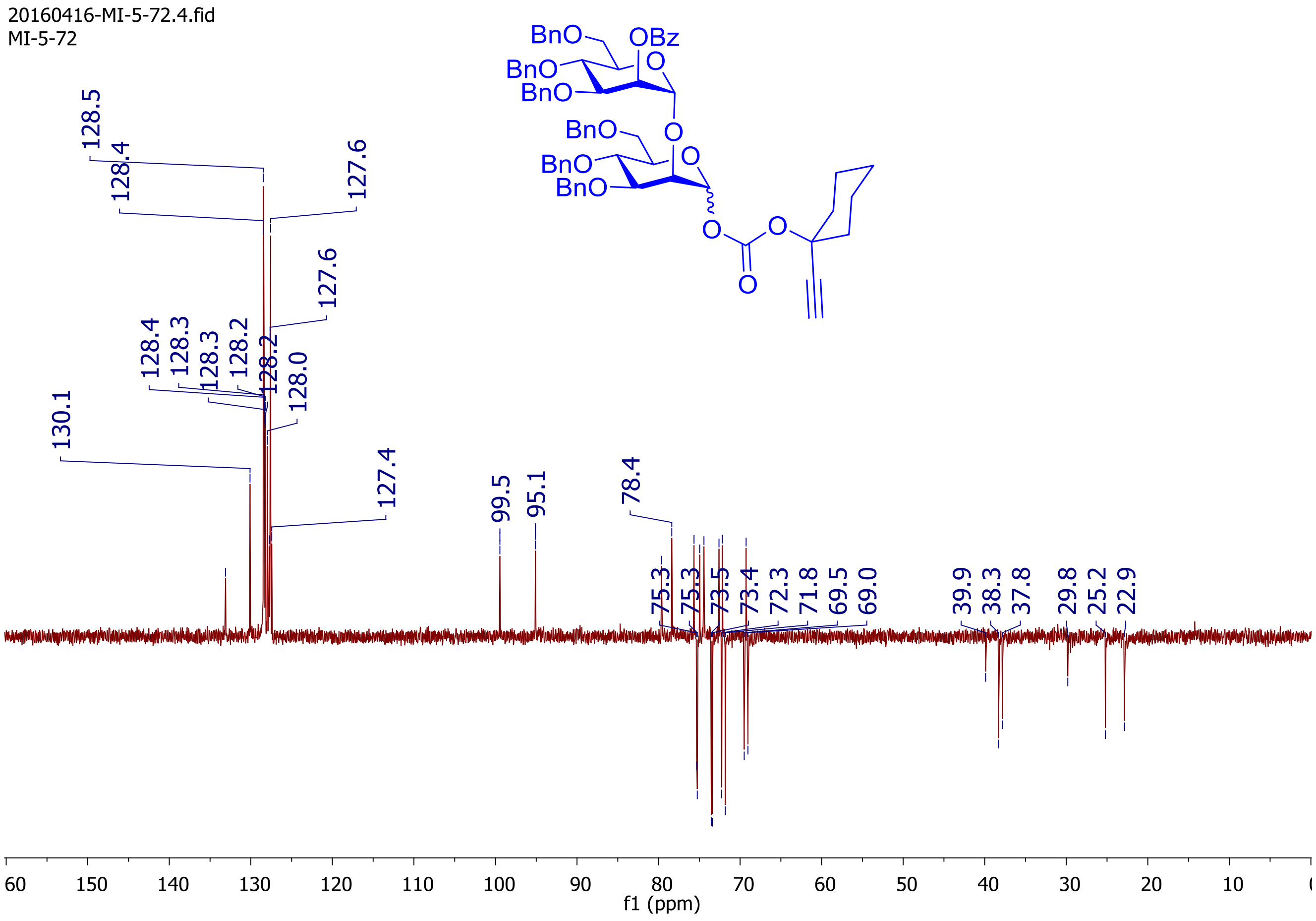

^{1}H NMR Spectrum (399.78 MHz, $CDCl_3$) of Compound **21**

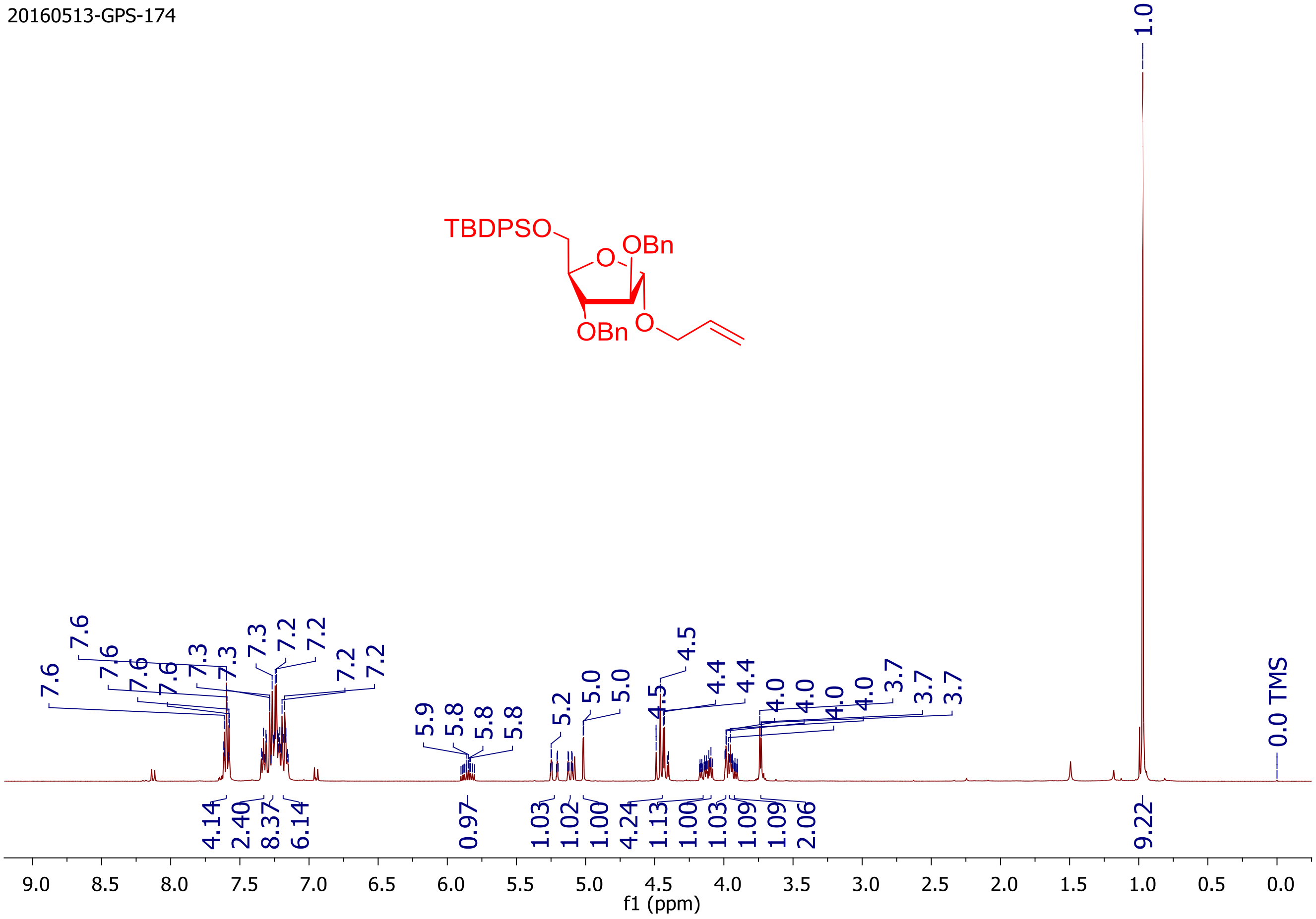

^{13}C NMR Spectrum (100.53 MHz, CDCl$_3$) of Compound **21**

20160513-GPS-174

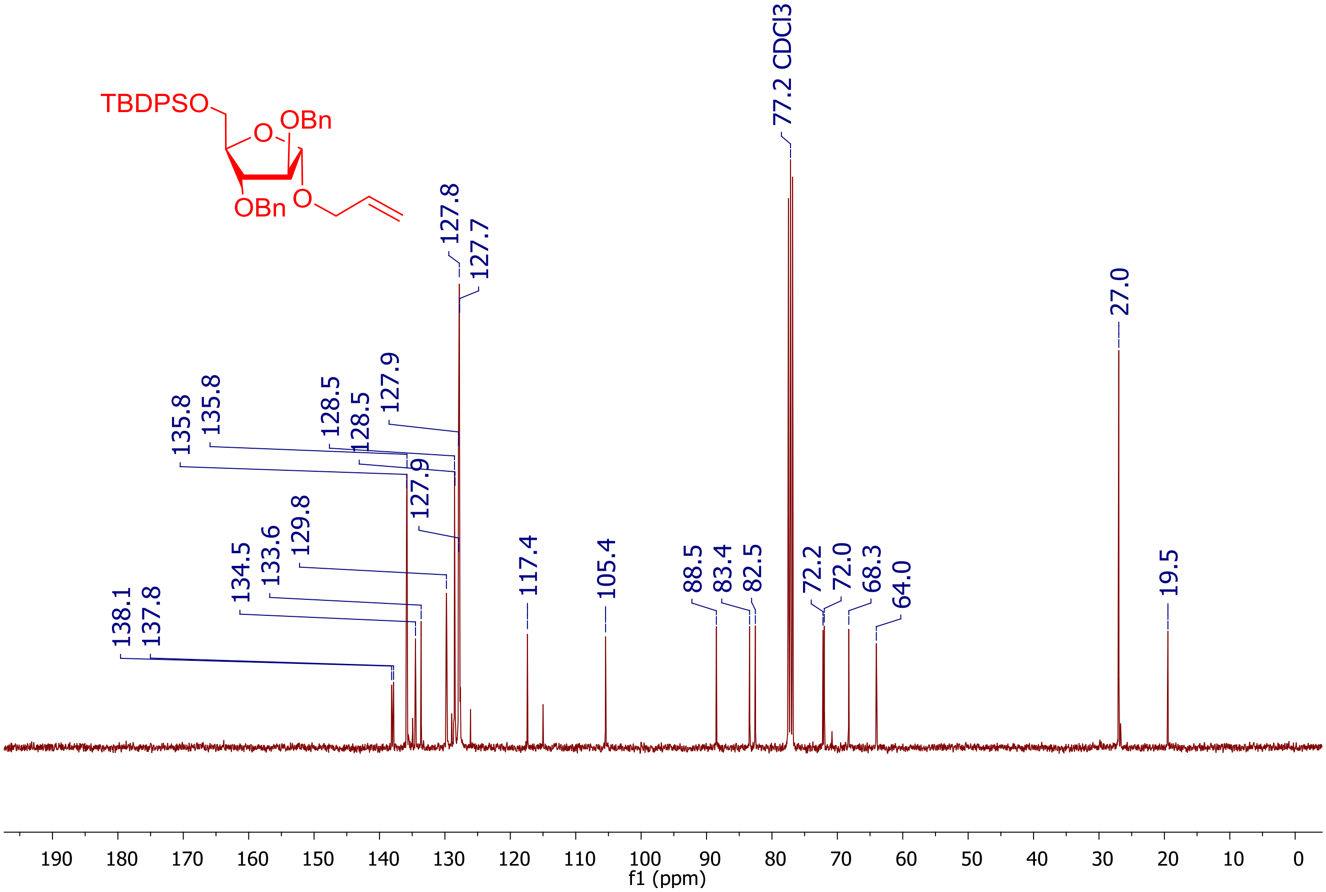

DEPT Spectrum (100.53 MHz, $CDCl_3$) of Compound **21**

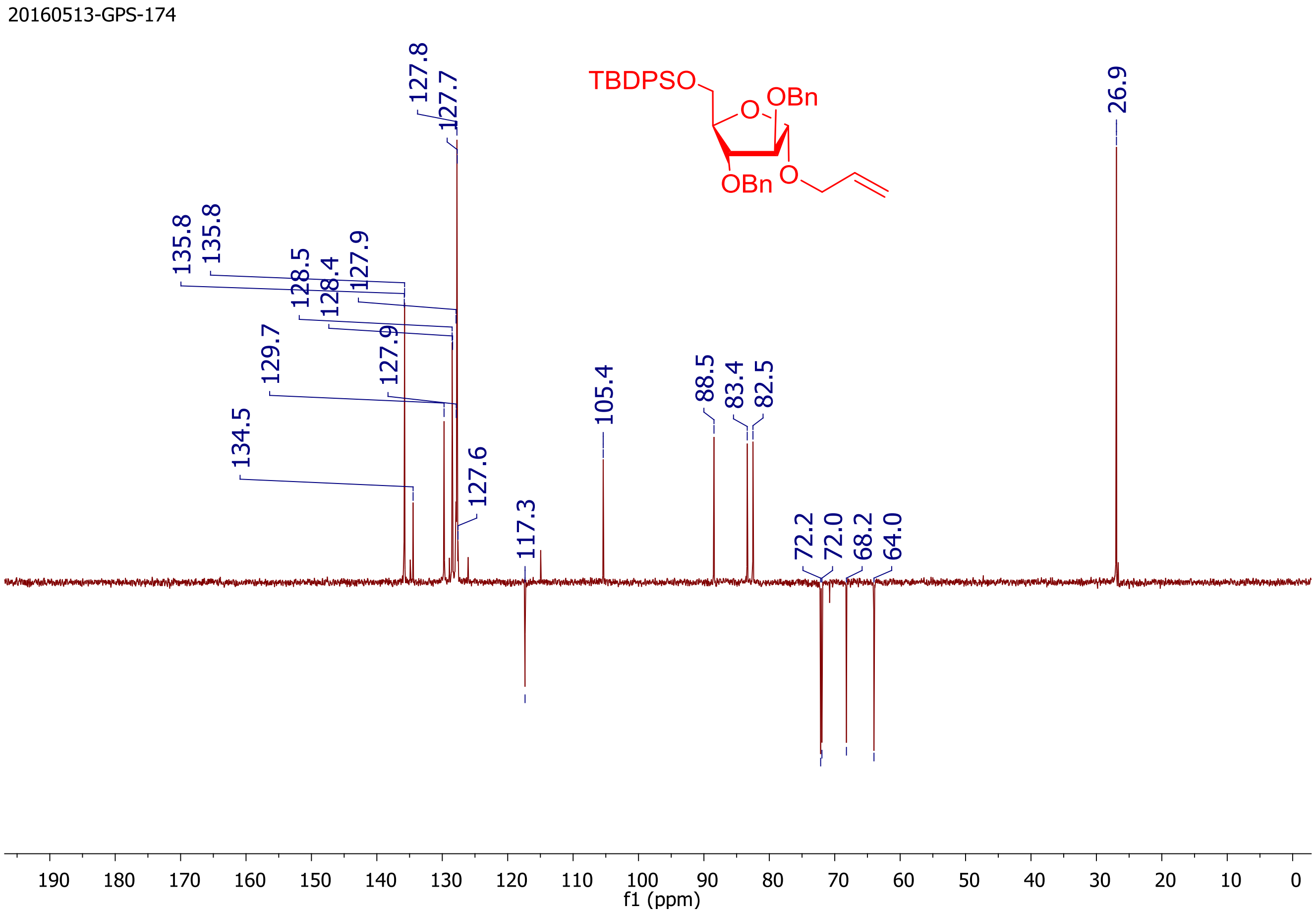

^{1}H NMR Spectrum (399.78 MHz, $CDCl_3$) of Compound **22**

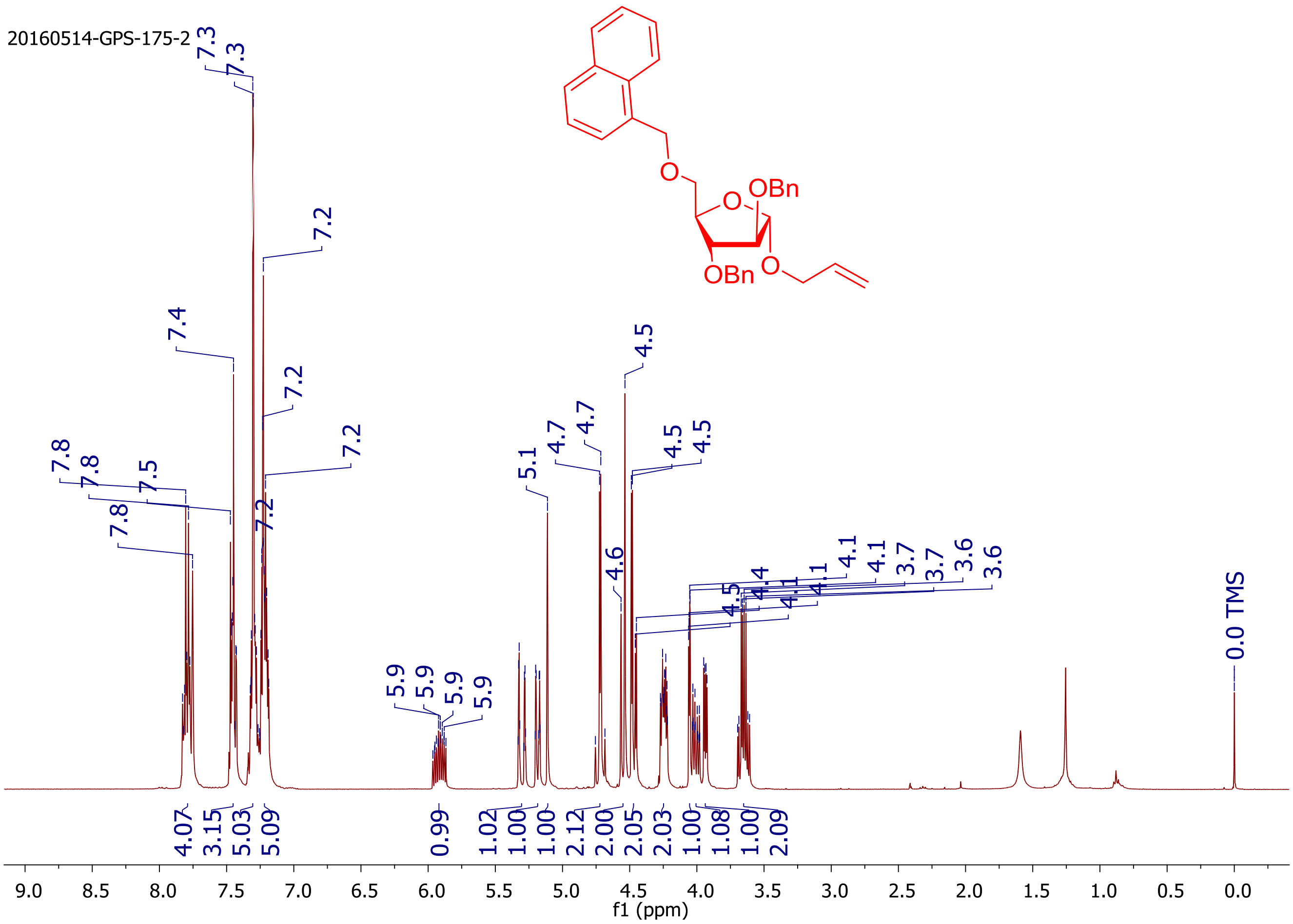

^{13}C NMR Spectrum (100.53 MHz, $CDCl_3$) of Compound **22**

20160514-GPS-175-2

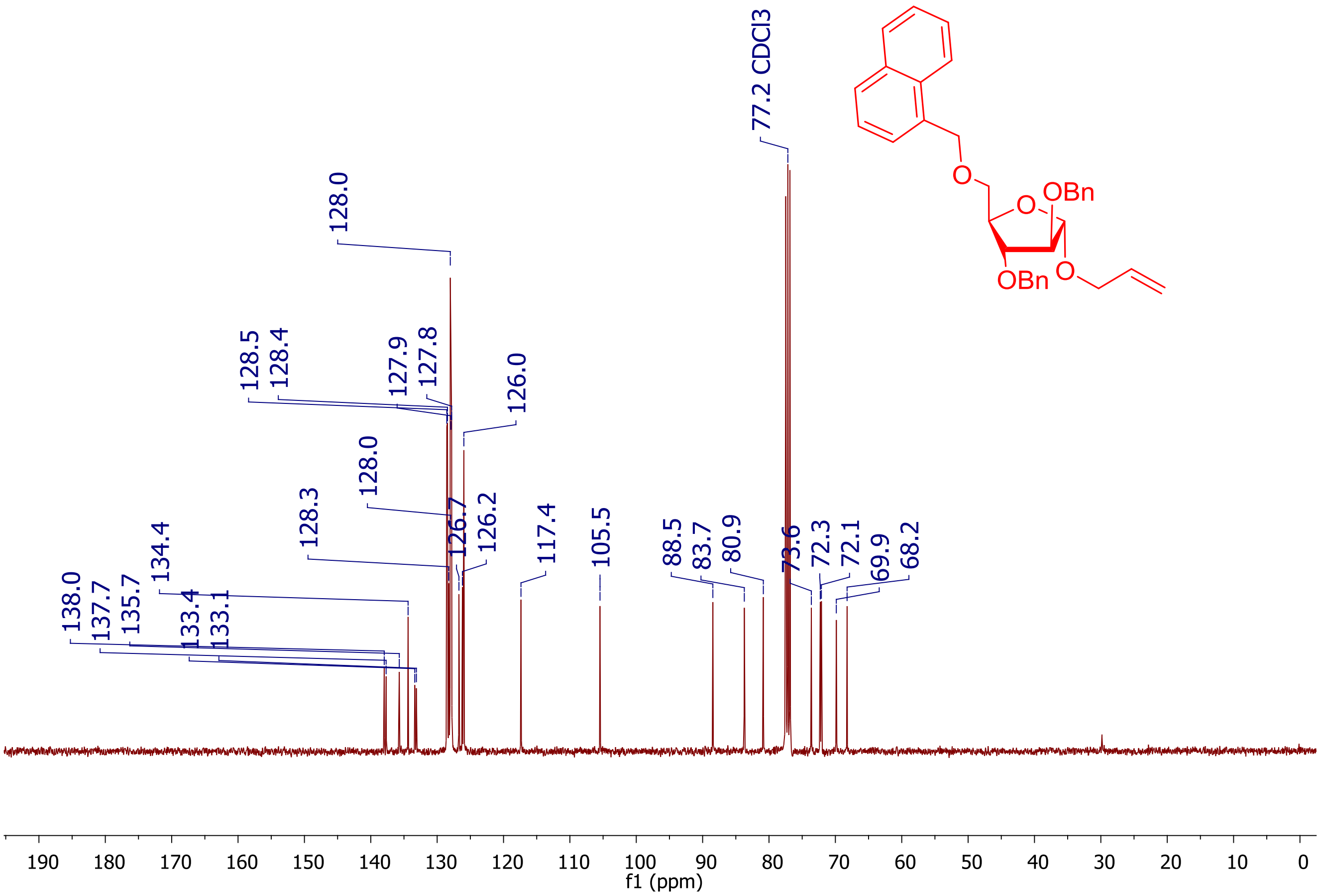

DEPT Spectrum (100.53 MHz, $CDCl_3$) of Compound **22**

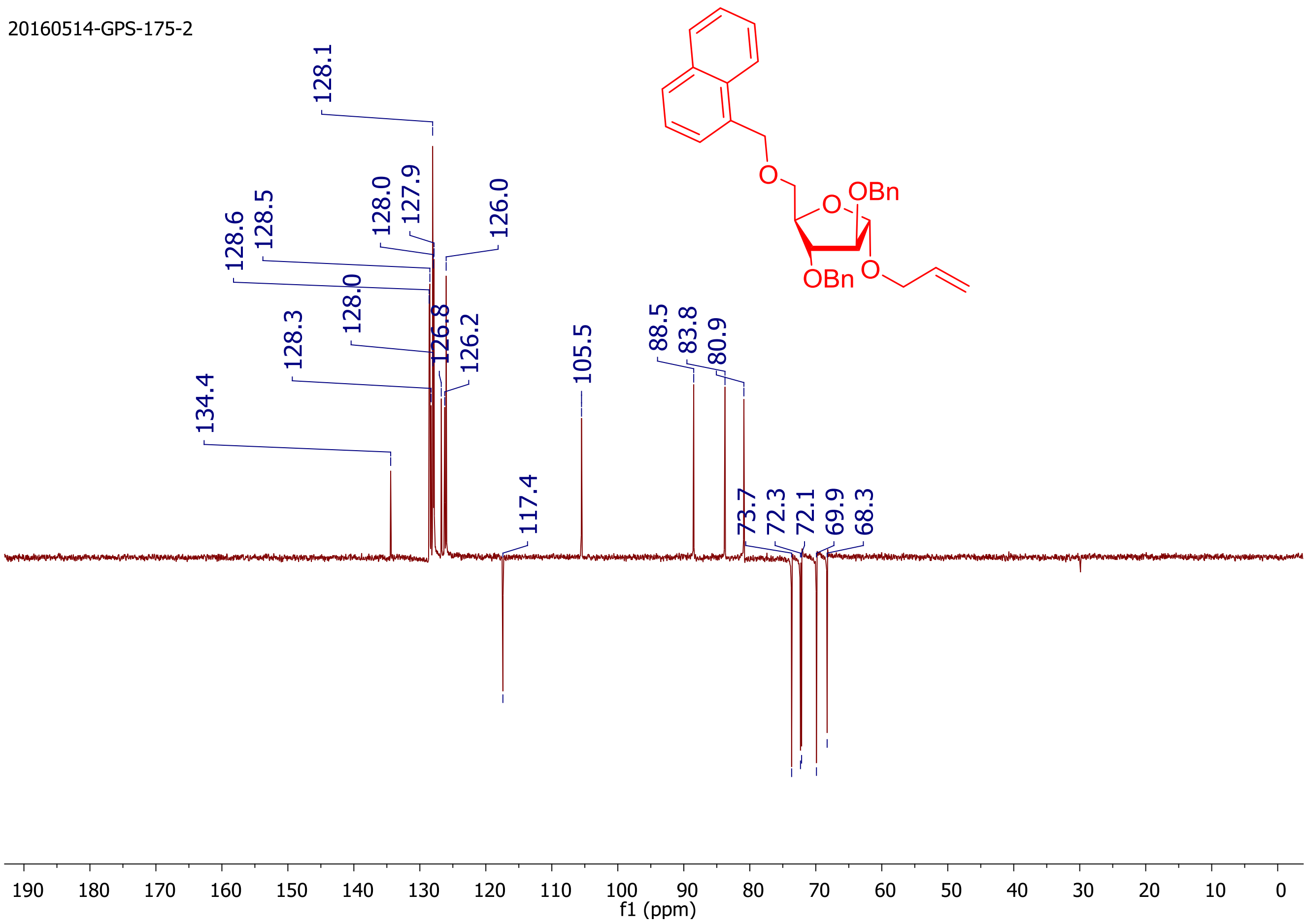

^{1}H NMR Spectrum (399.78 MHz, $CDCl_3$) of Compound **23**

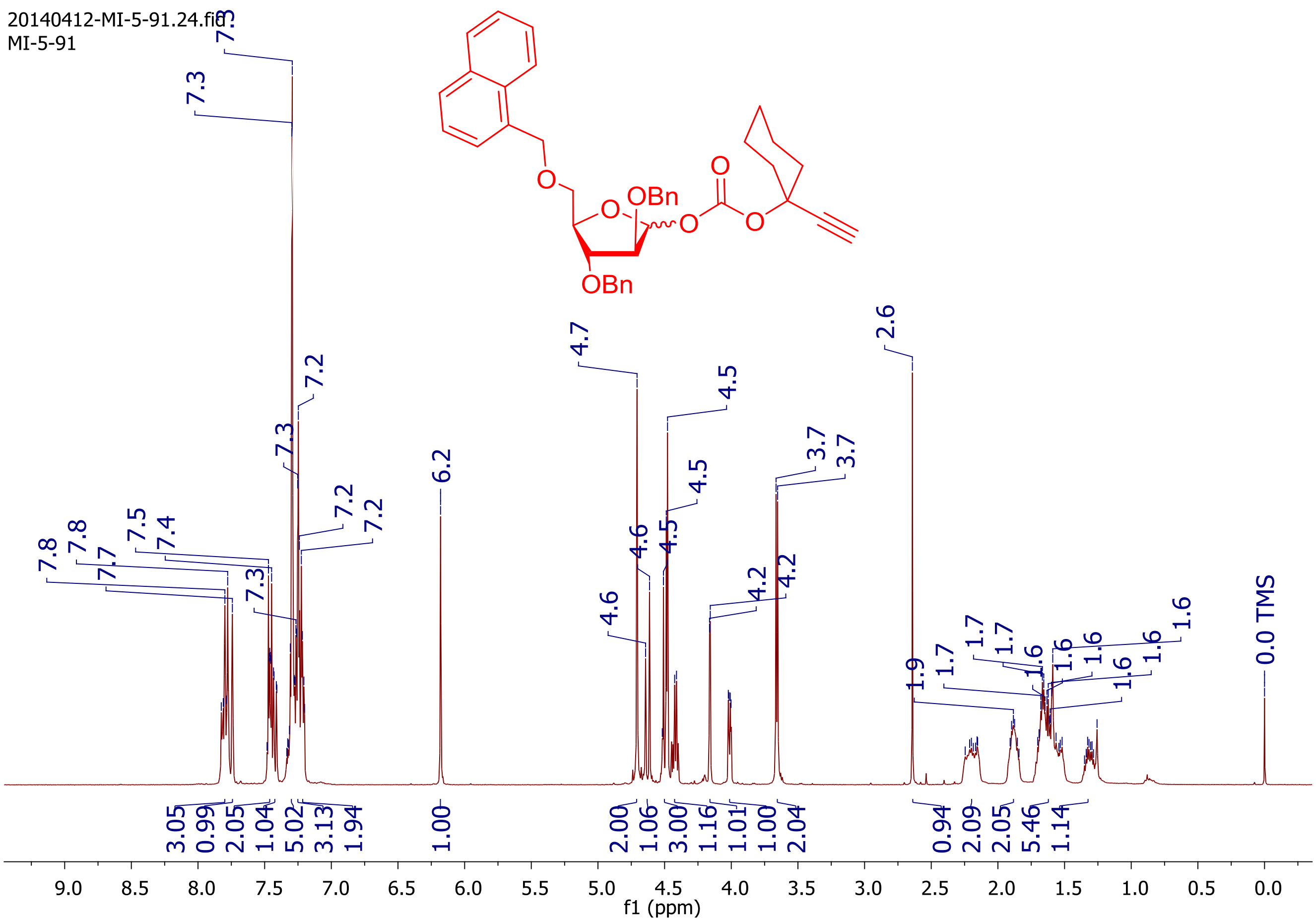

^{13}C NMR Spectrum (100.53 MHz, $CDCl_3$) of Compound **23**

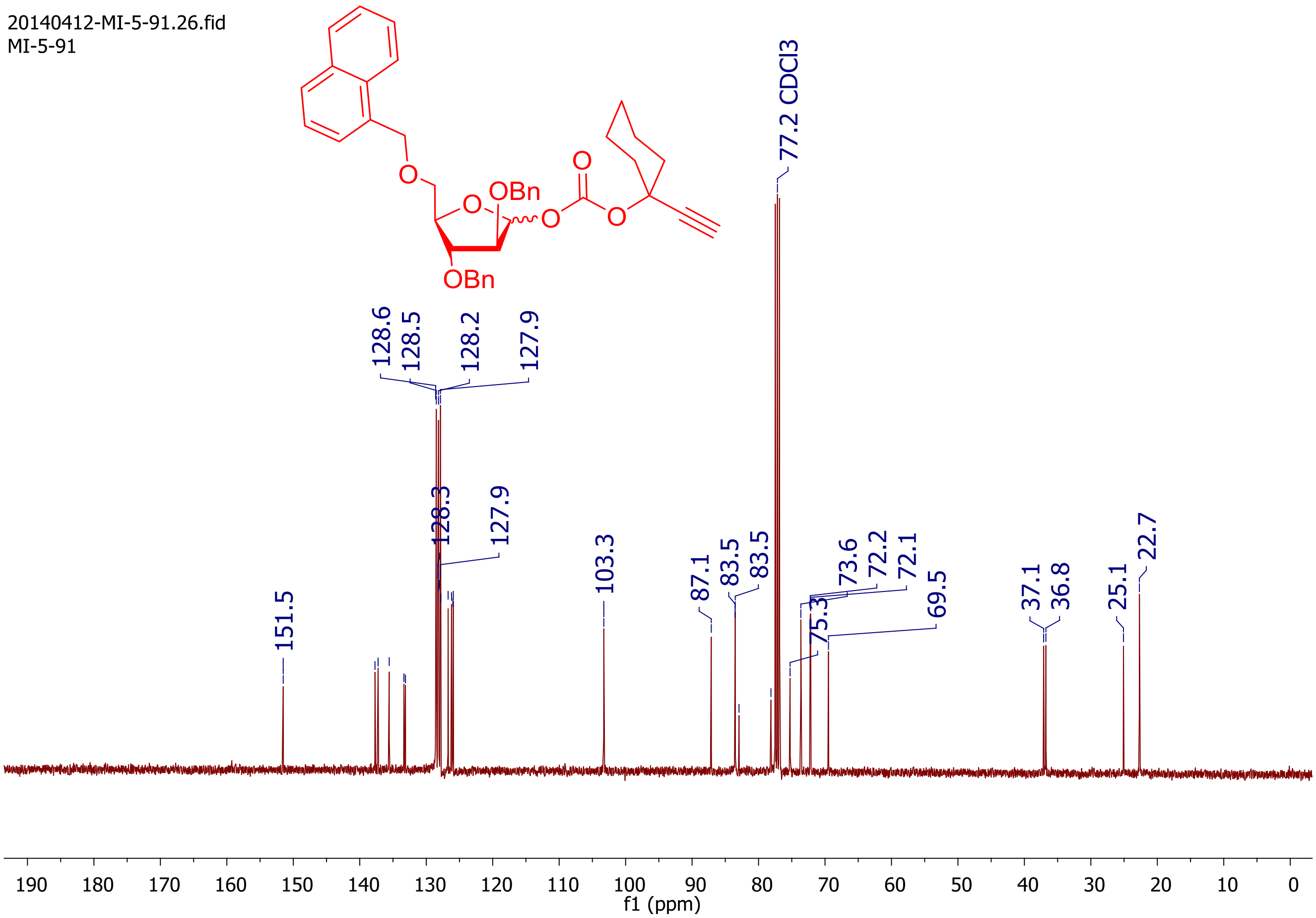

DEPT Spectrum (100.53 MHz, $CDCl_3$) of Compound **23**

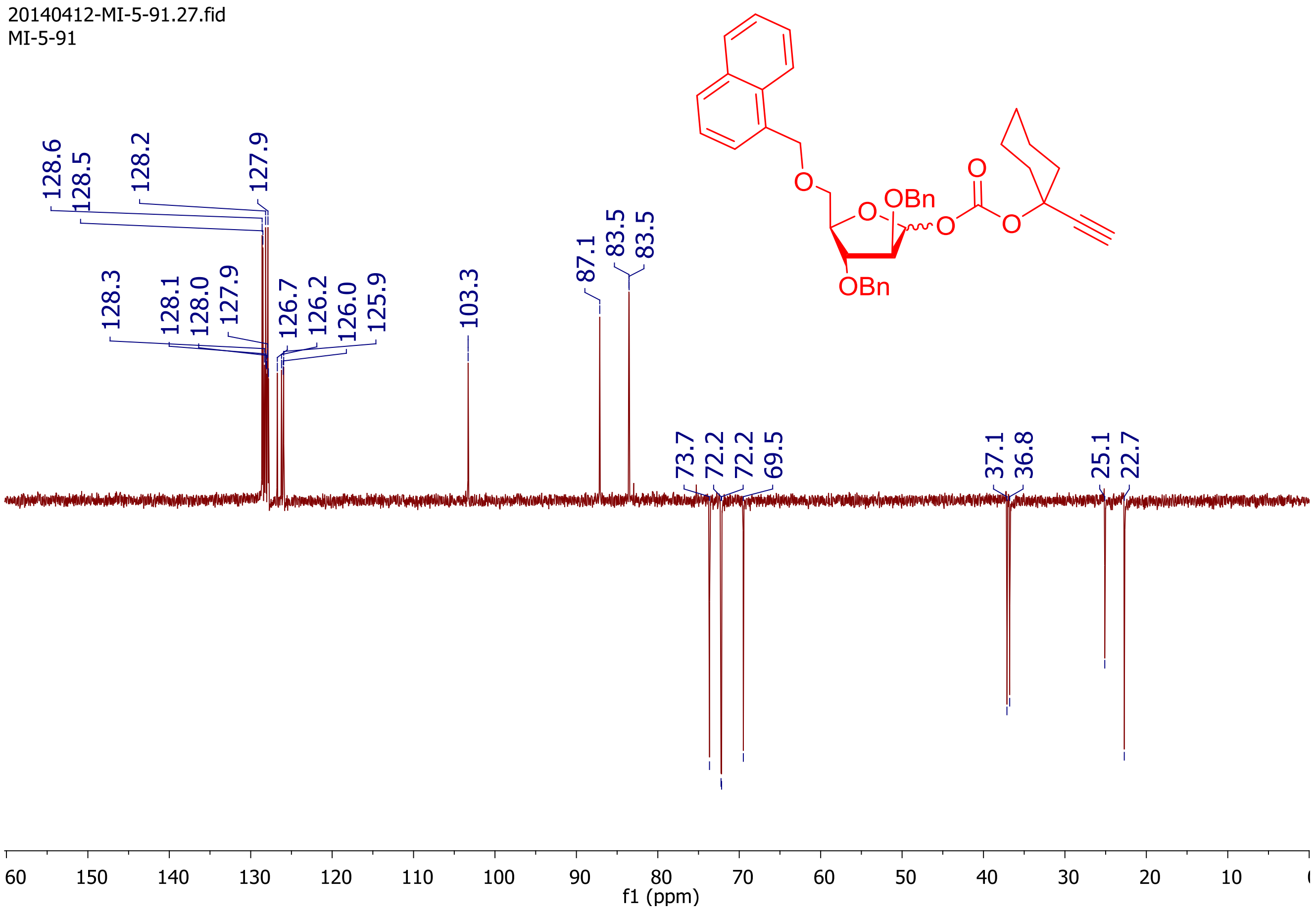

^{1}H NMR Spectrum (399.78 MHz, $CDCl_3$) of Compound **26**

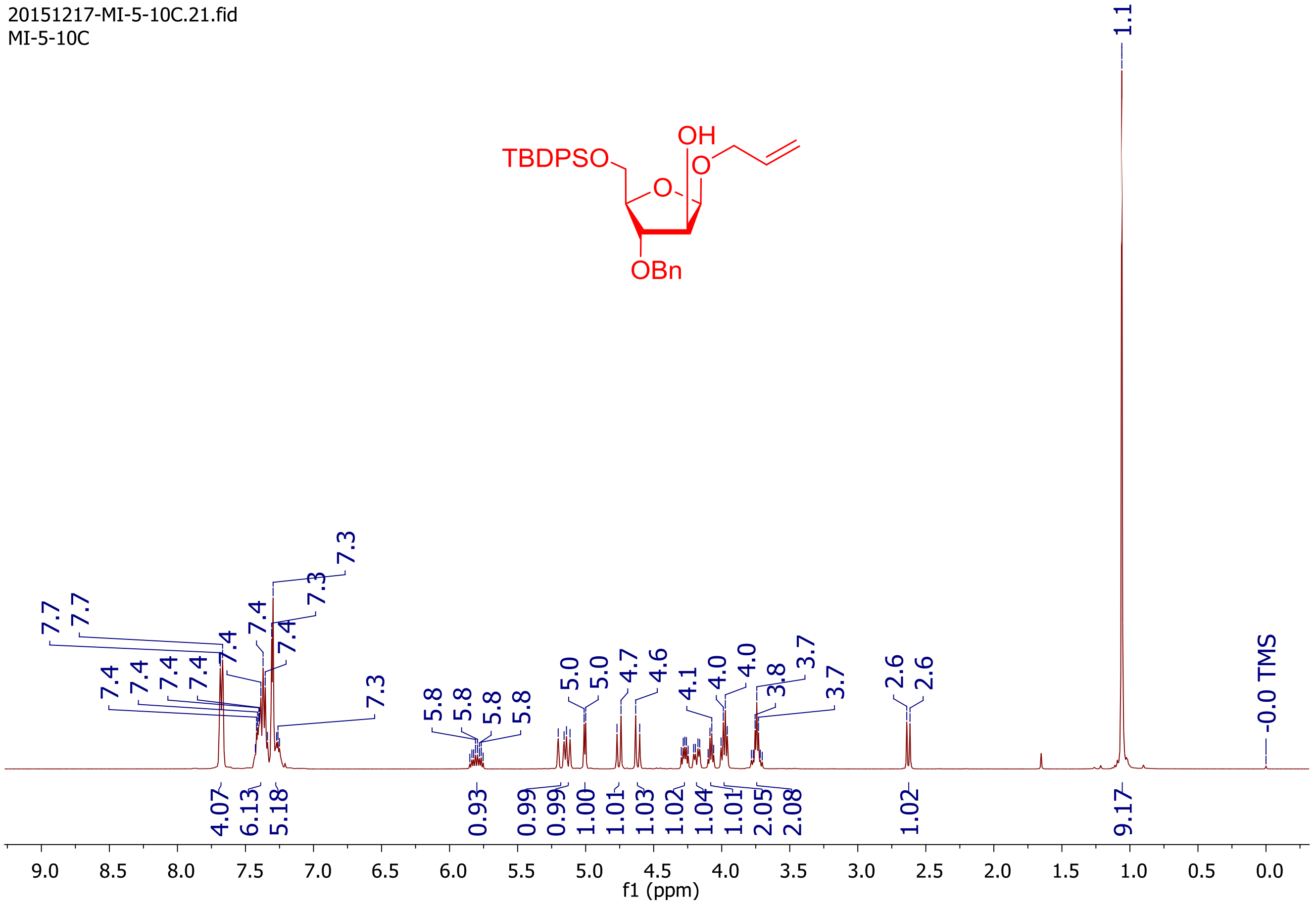

^{13}C NMR Spectrum (100.53 MHz, $CDCl_3$) of Compound **26**

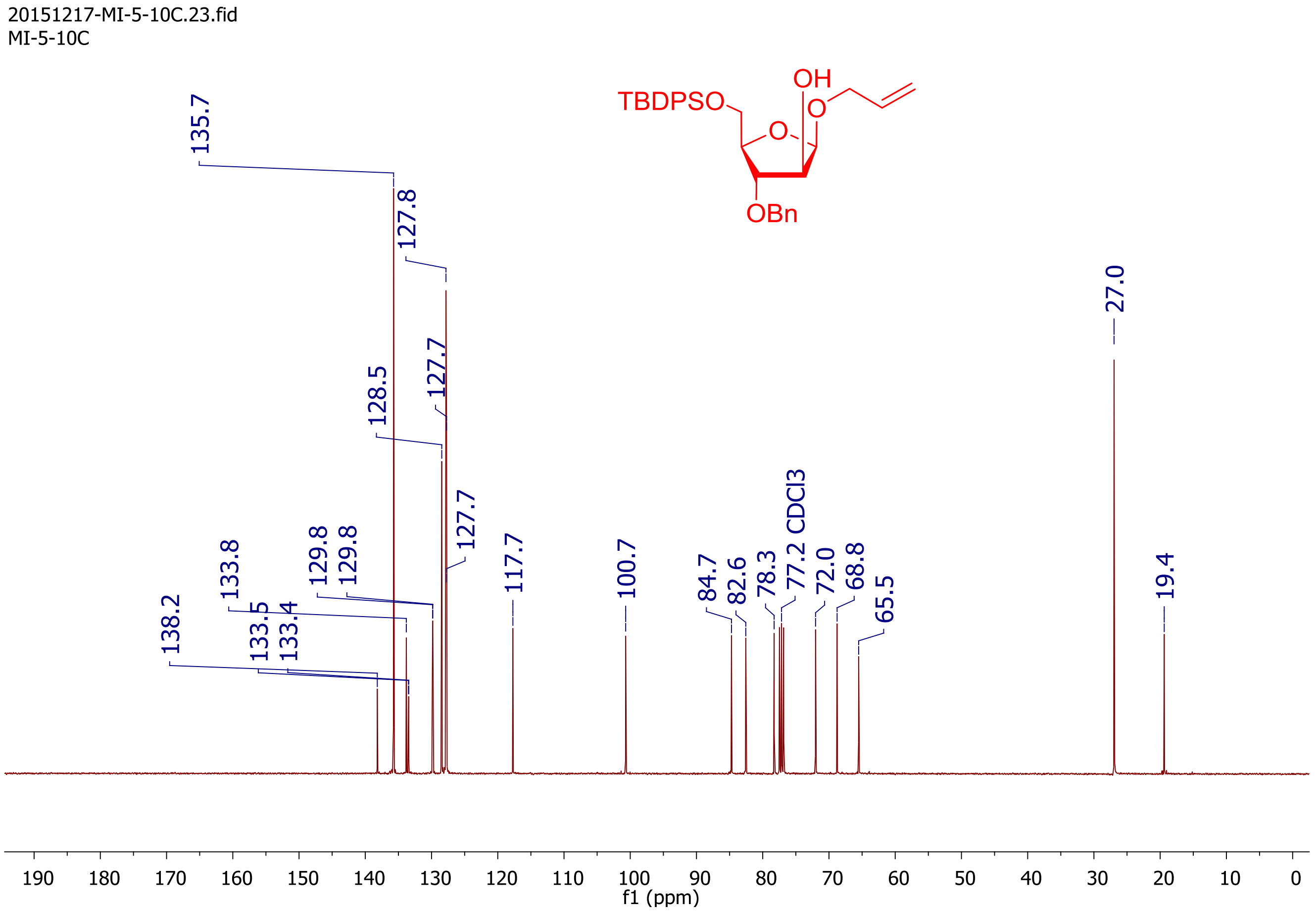

DEPT Spectrum (100.53 MHz, $CDCl_3$) of Compound **26**

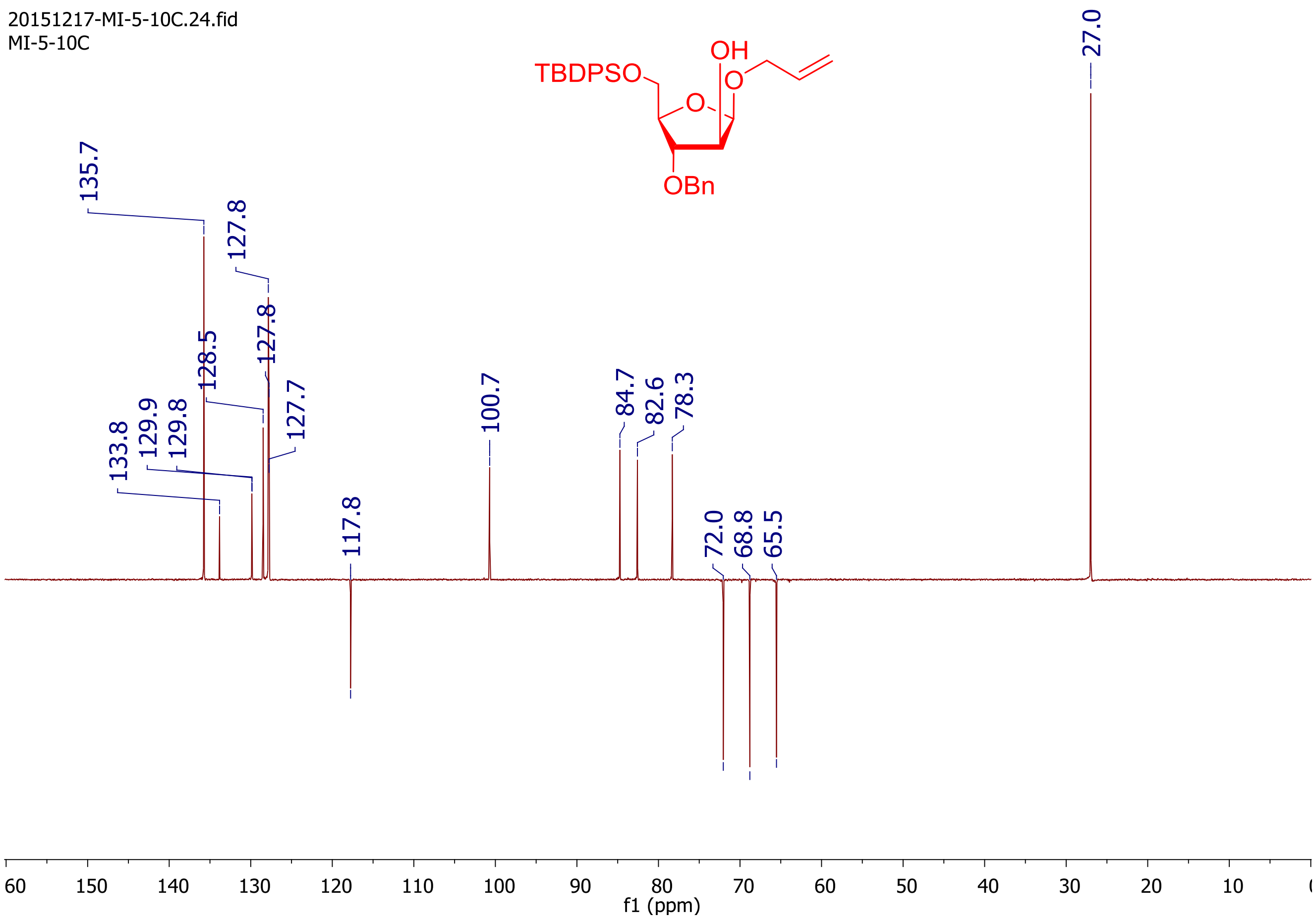

^{1}H NMR Spectrum (399.78 MHz, $CDCl_3$) of Compound **27**

20151126-MI-5-2.40.fid
MI-5-2

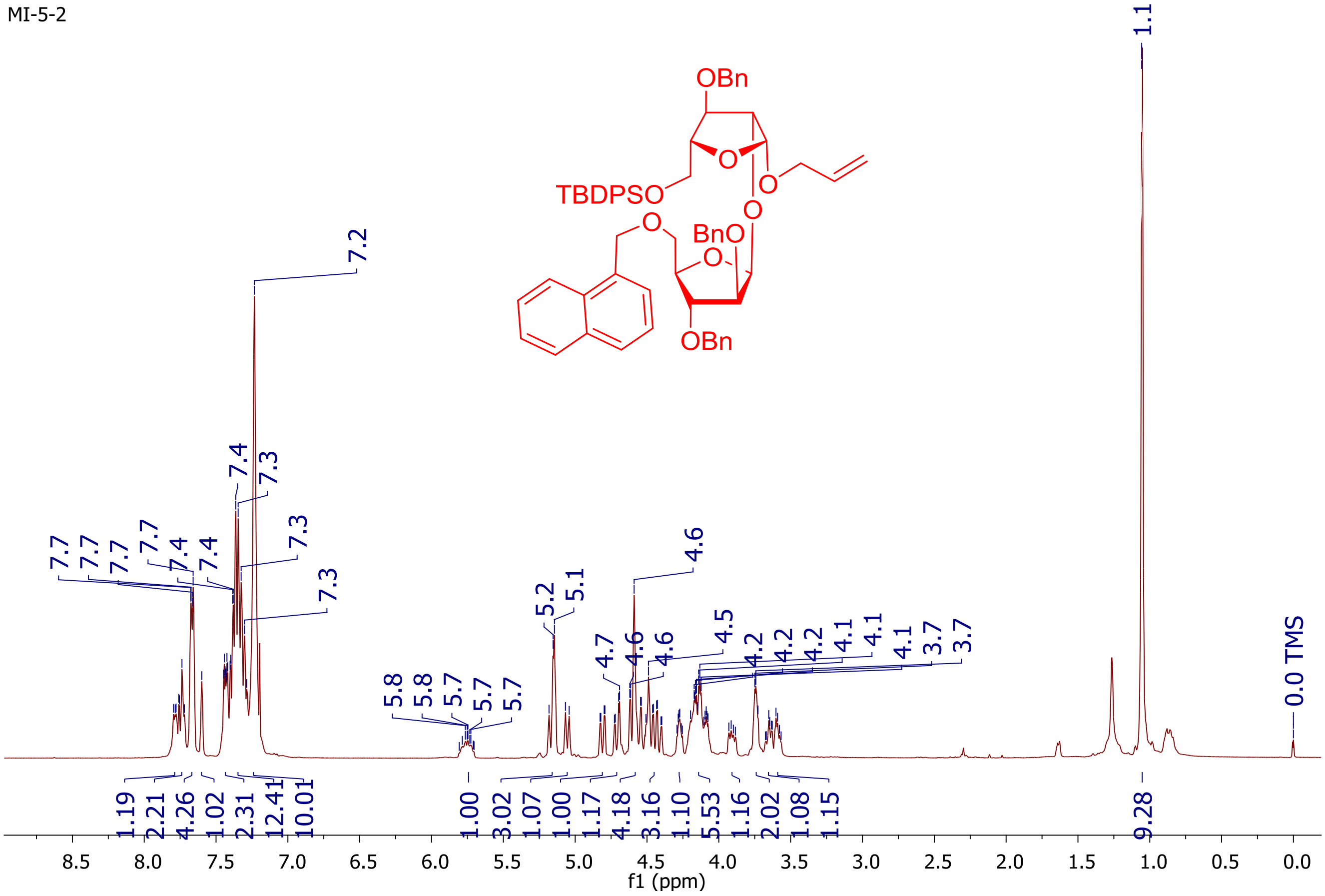

^{13}C NMR Spectrum (100.53 MHz, $CDCl_3$) of Compound **27**

20151126-MI-5-2.42.fid
MI-5-2

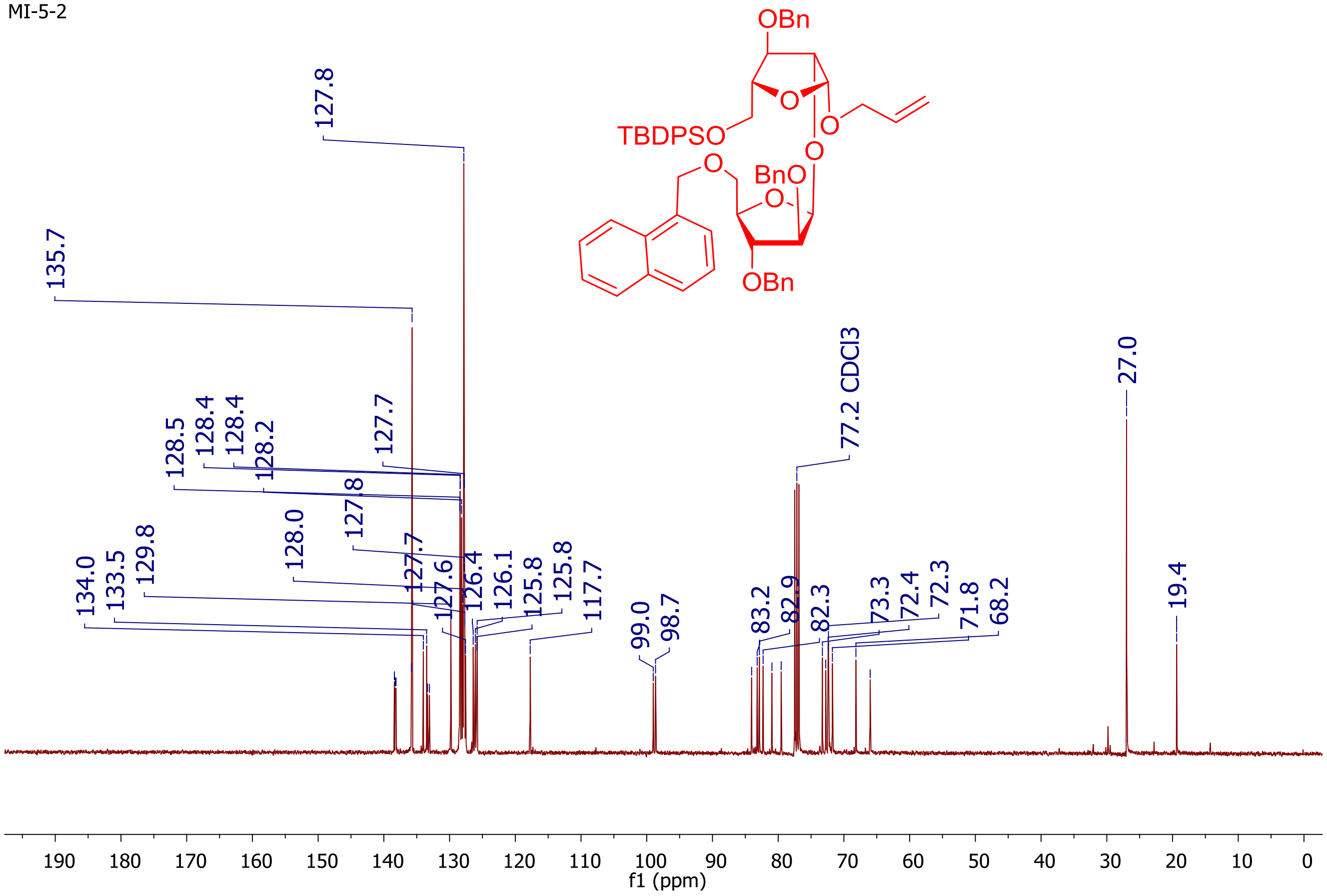

DEPT Spectrum (100.53 MHz, $CDCl_3$) of Compound **27**

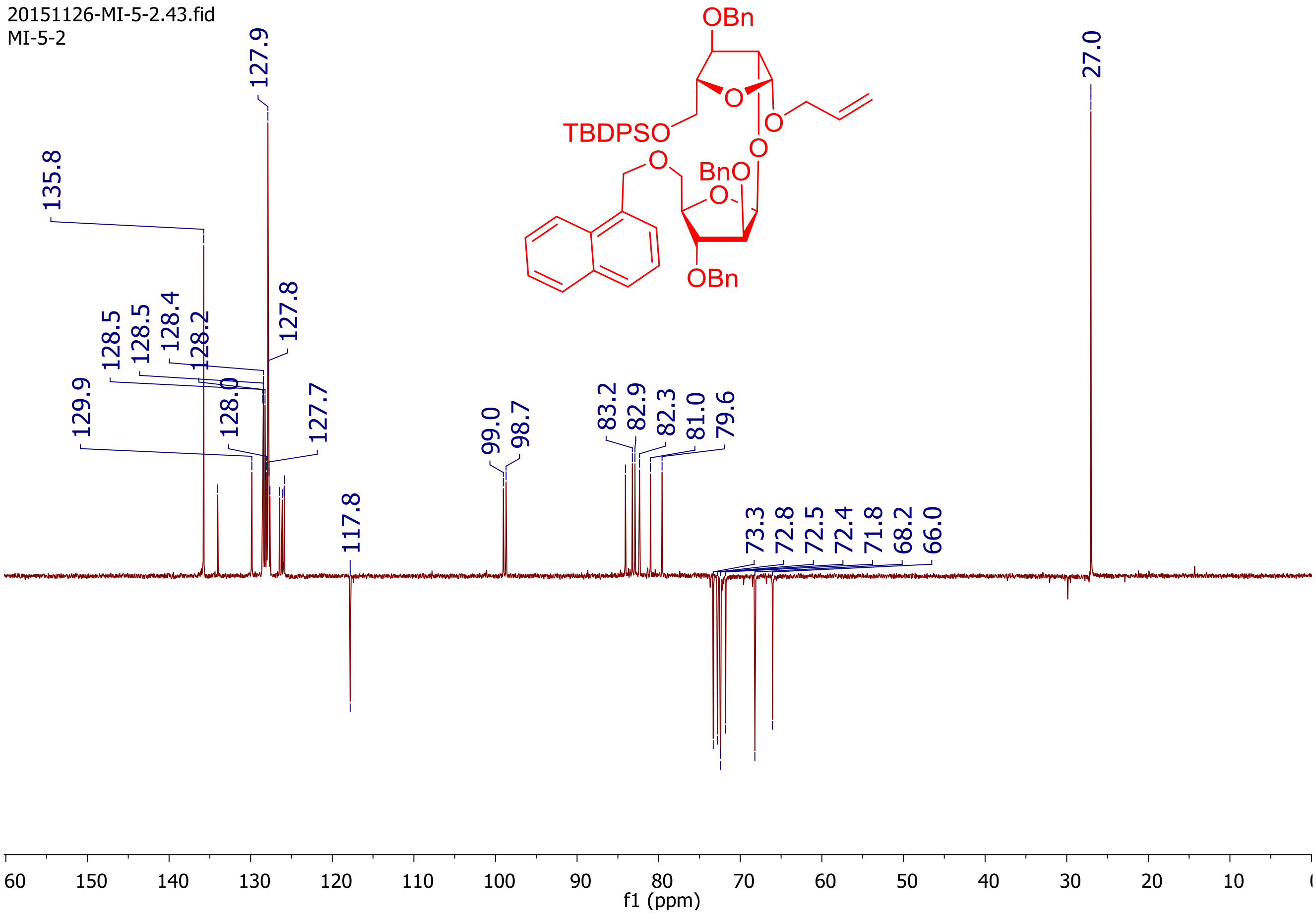

^{1}H NMR Spectrum (399.78 MHz, $CDCl_3$) of Compound **3**

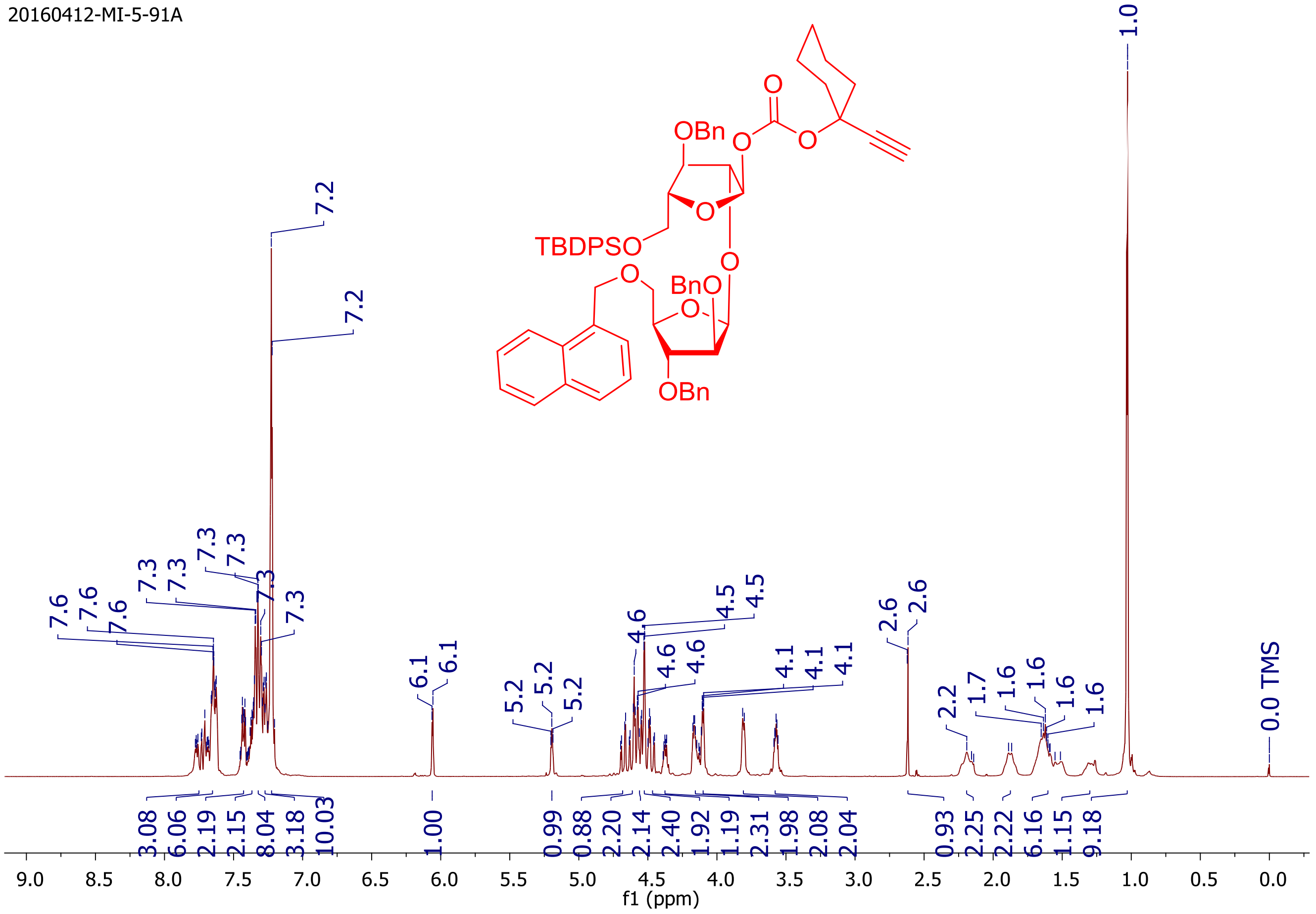

^{13}C NMR Spectrum (100.53 MHz, $CDCl_3$) of Compound **3**

20160412-MI-5-91A

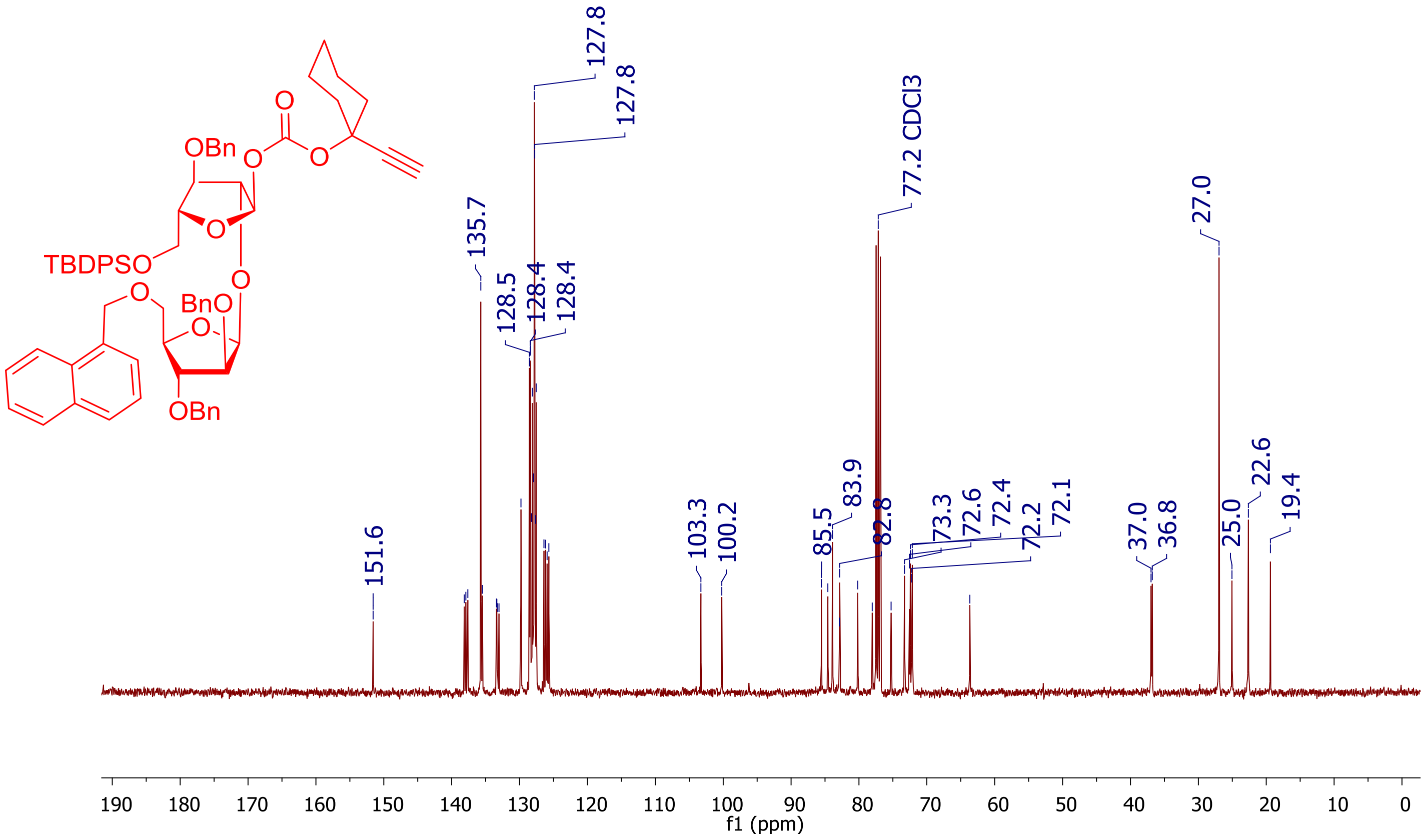

DEPT Spectrum (100.53 MHz, $CDCl_3$) of Compound **3**

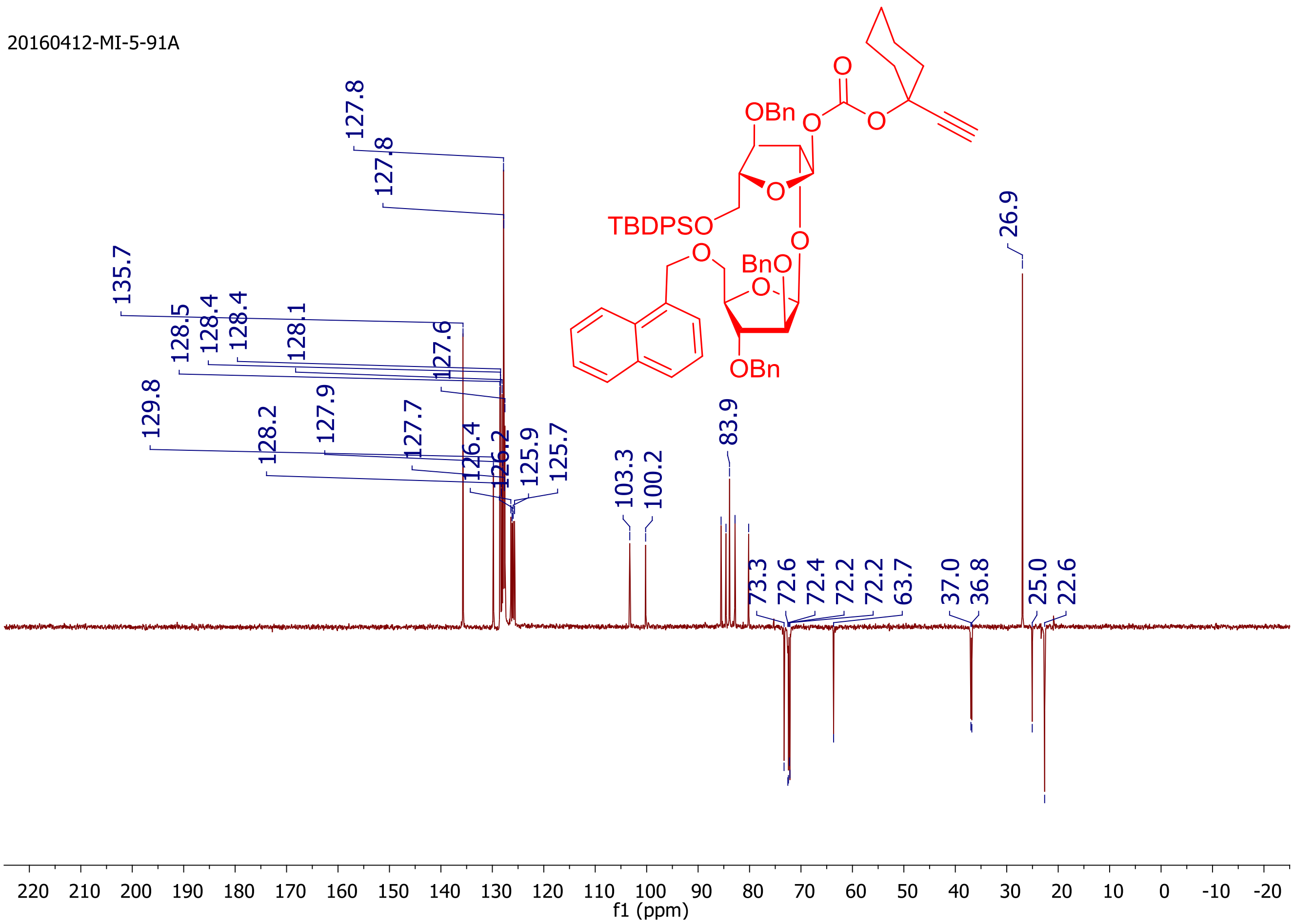

^{1}H NMR Spectrum (399.78 MHz, $CDCl_3$) of Compound **4**

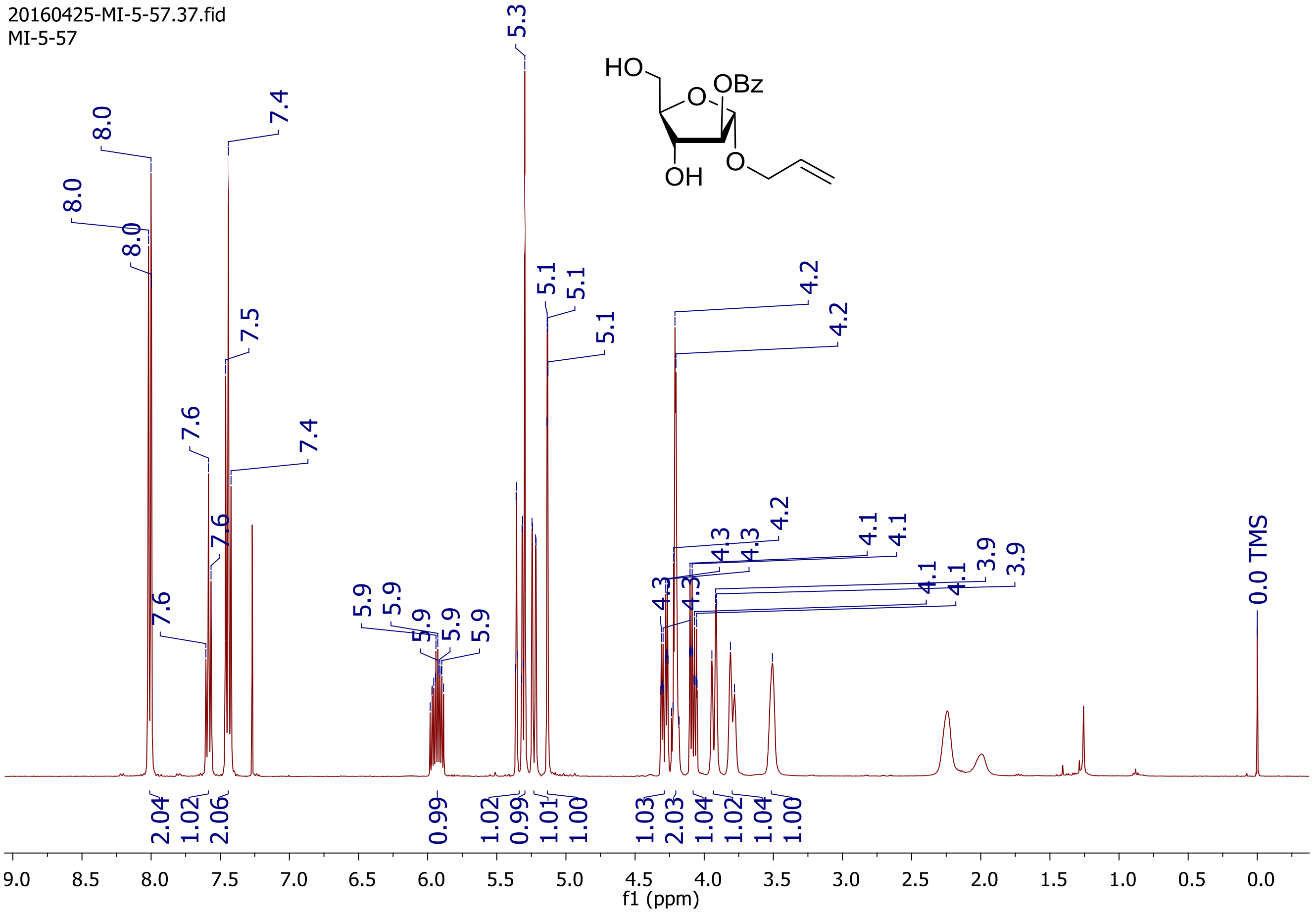

^{13}C NMR Spectrum (100.53 MHz, $CDCl_3$) of Compound **4**

20160425-MI-5-57.39.fid
MI-5-57

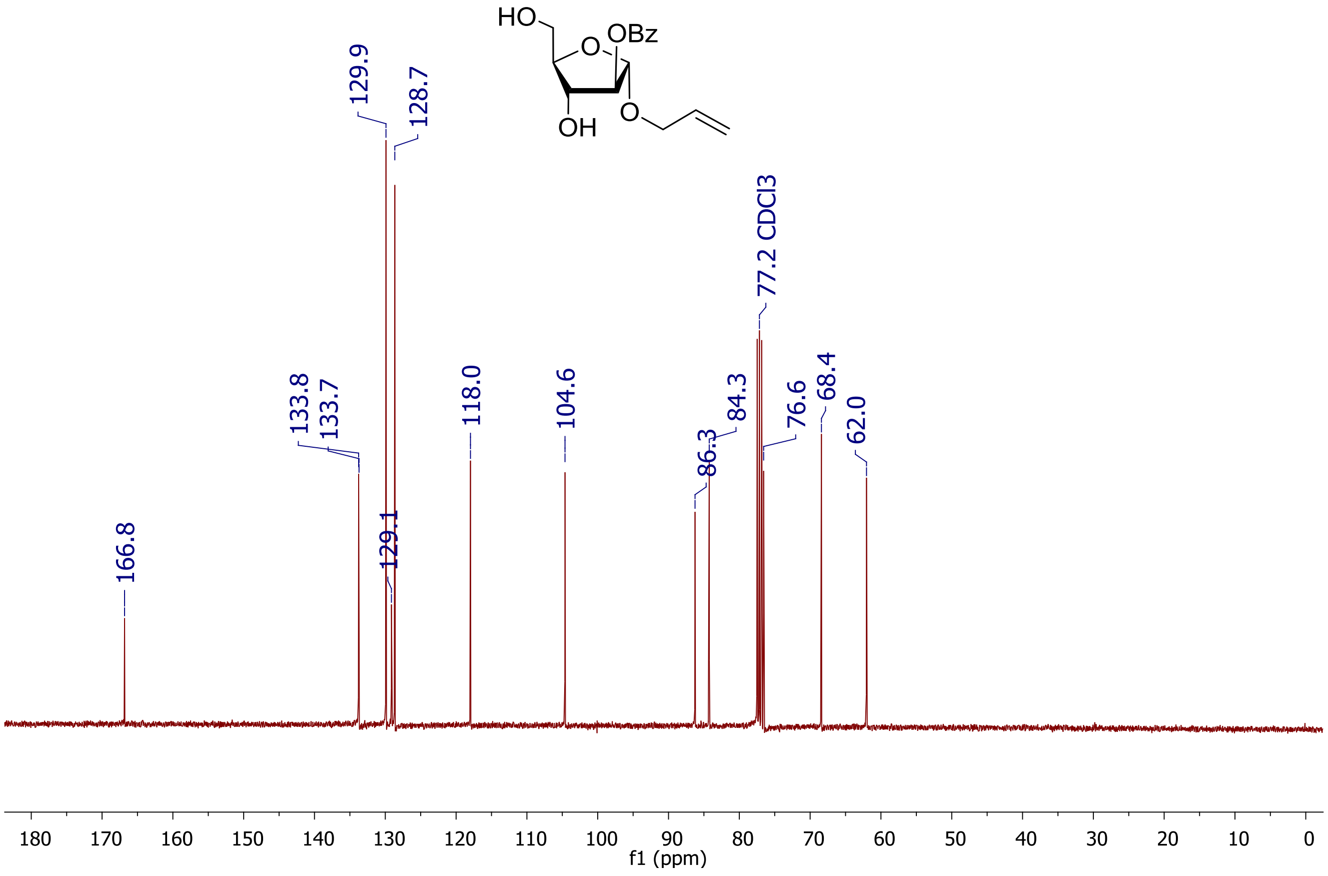

DEPT Spectrum (100.53 MHz, $CDCl_3$) of Compound **4**

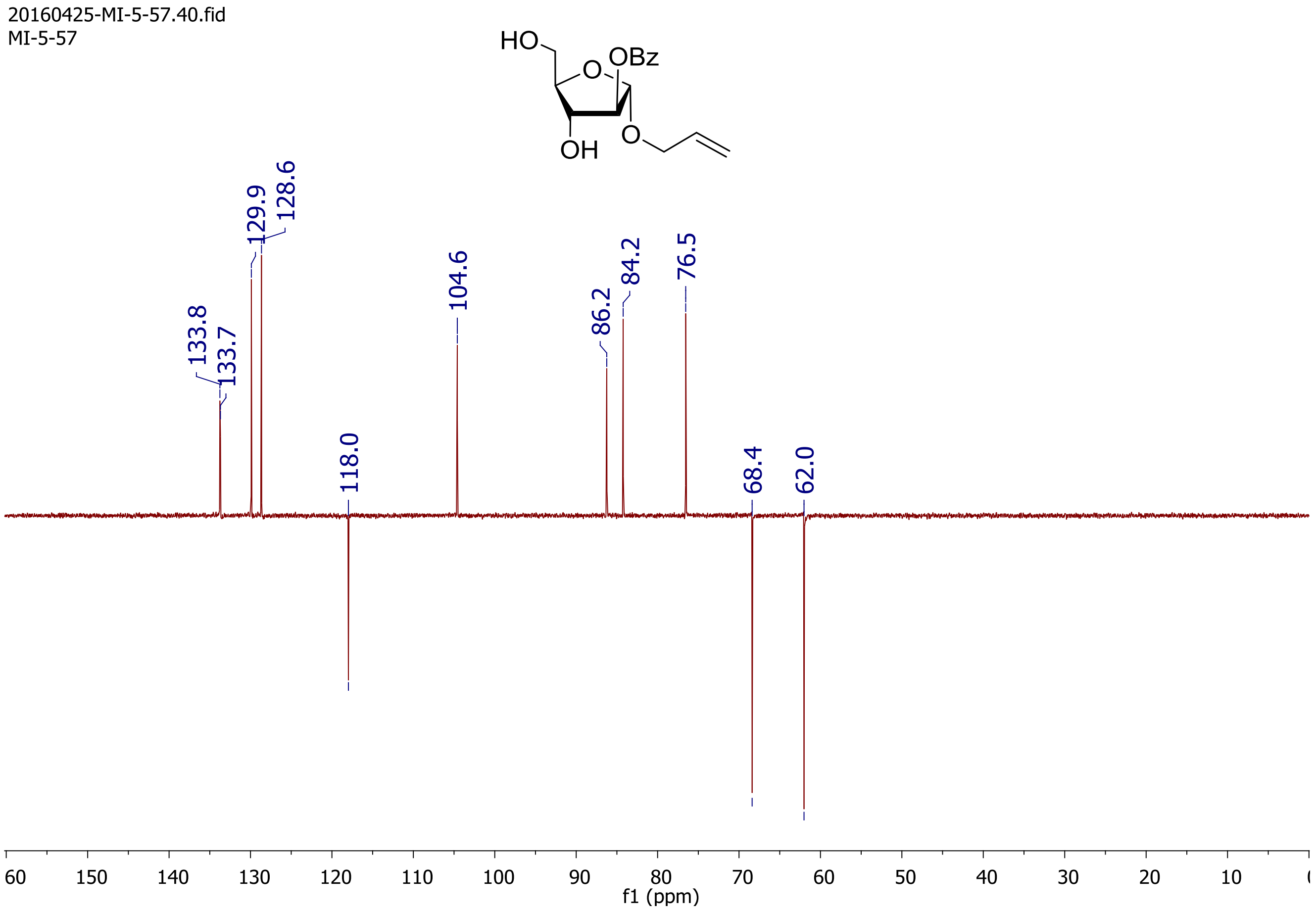

^{1}H NMR Spectrum (399.78 MHz, $CDCl_3$) of Compound **31**

20160330-MI-5-82/2
MI-5-82

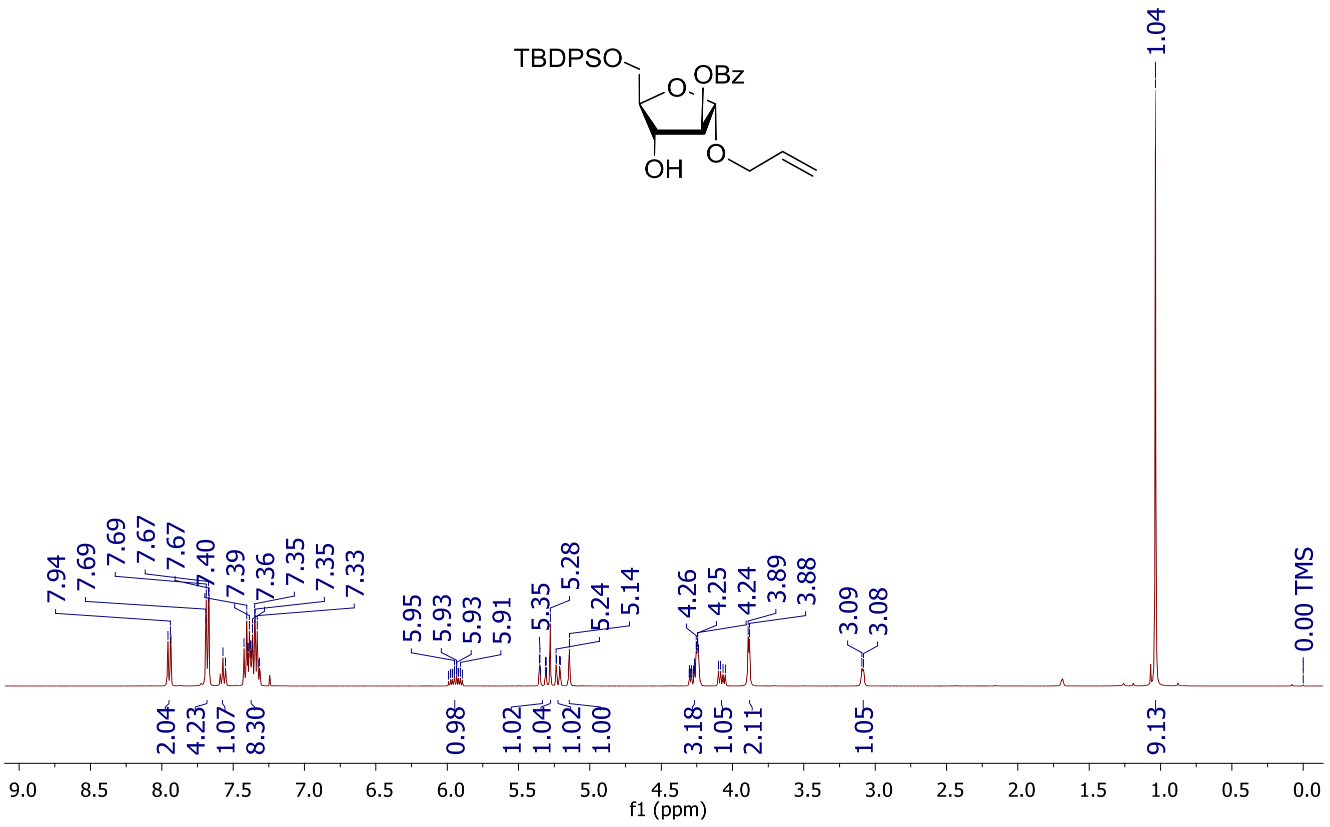

^{13}C NMR Spectrum (100.53 MHz, $CDCl_3$) of Compound **31**

20160330-MI-5-82/3
MI-5-82

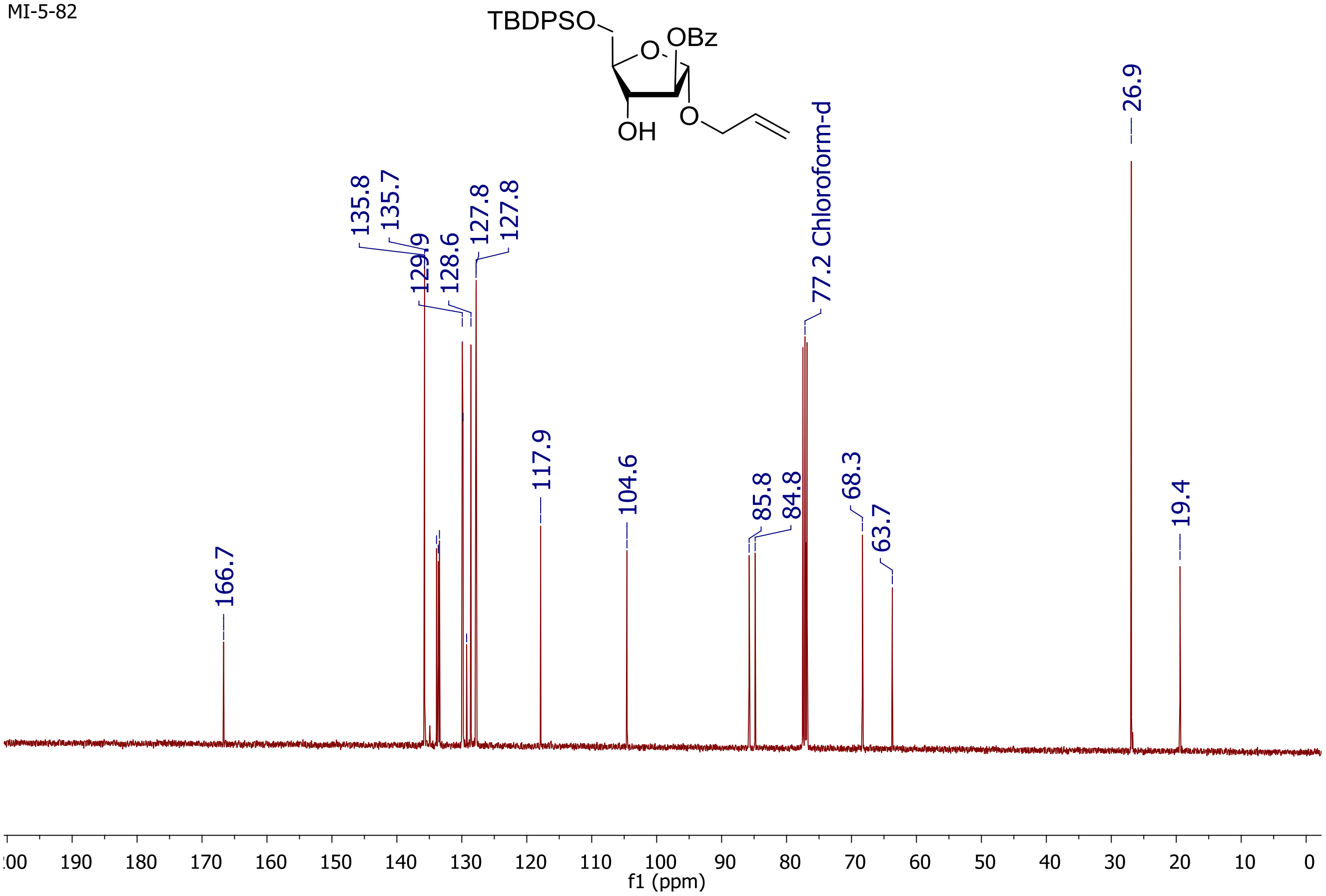

DEPT Spectrum (100.53 MHz, $CDCl_3$) of Compound **31**

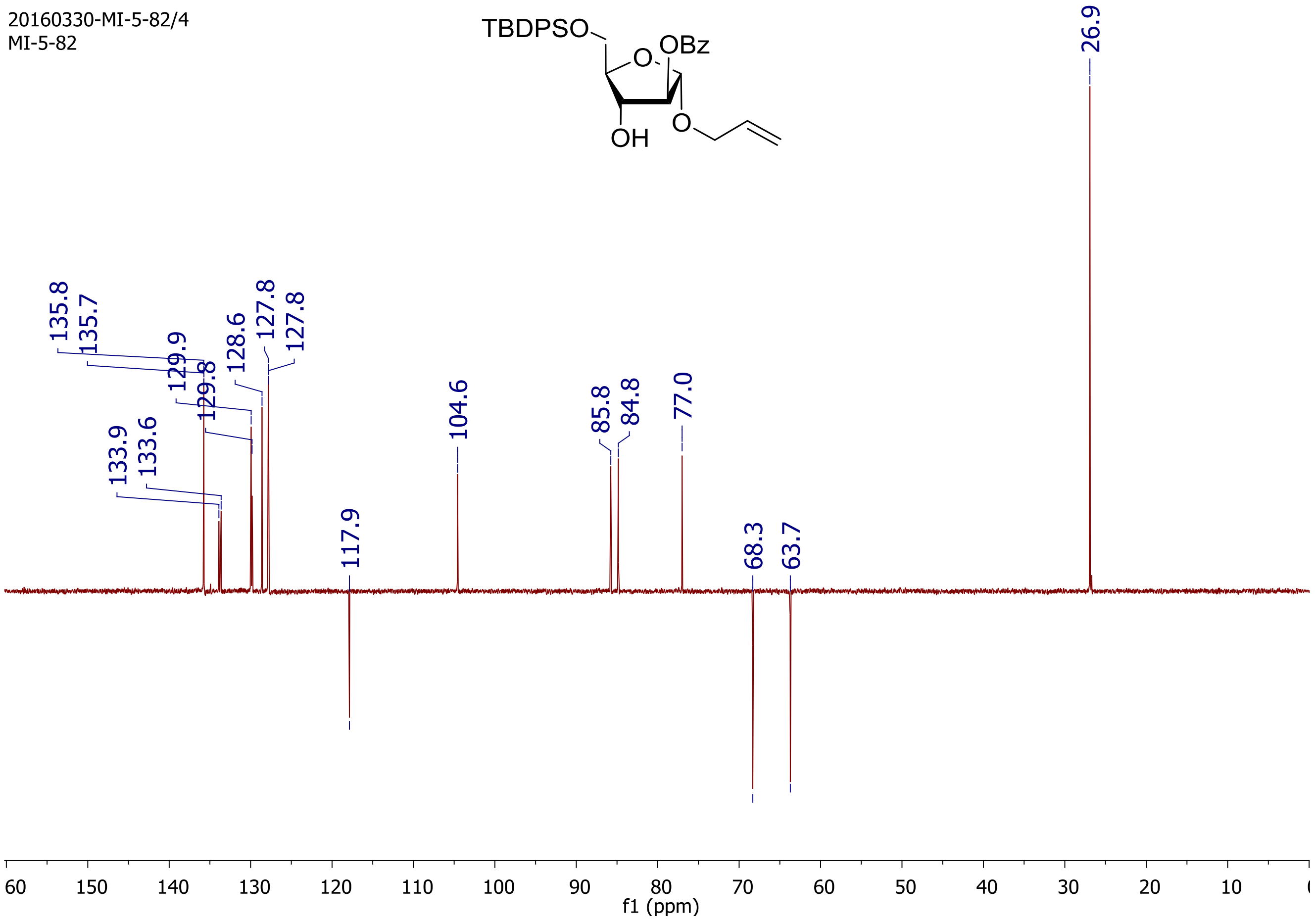

^{1}H NMR Spectrum (399.78 MHz, $CDCl_3$) of Compound **32**

20160330-MI-5-83/5
MI-5-83

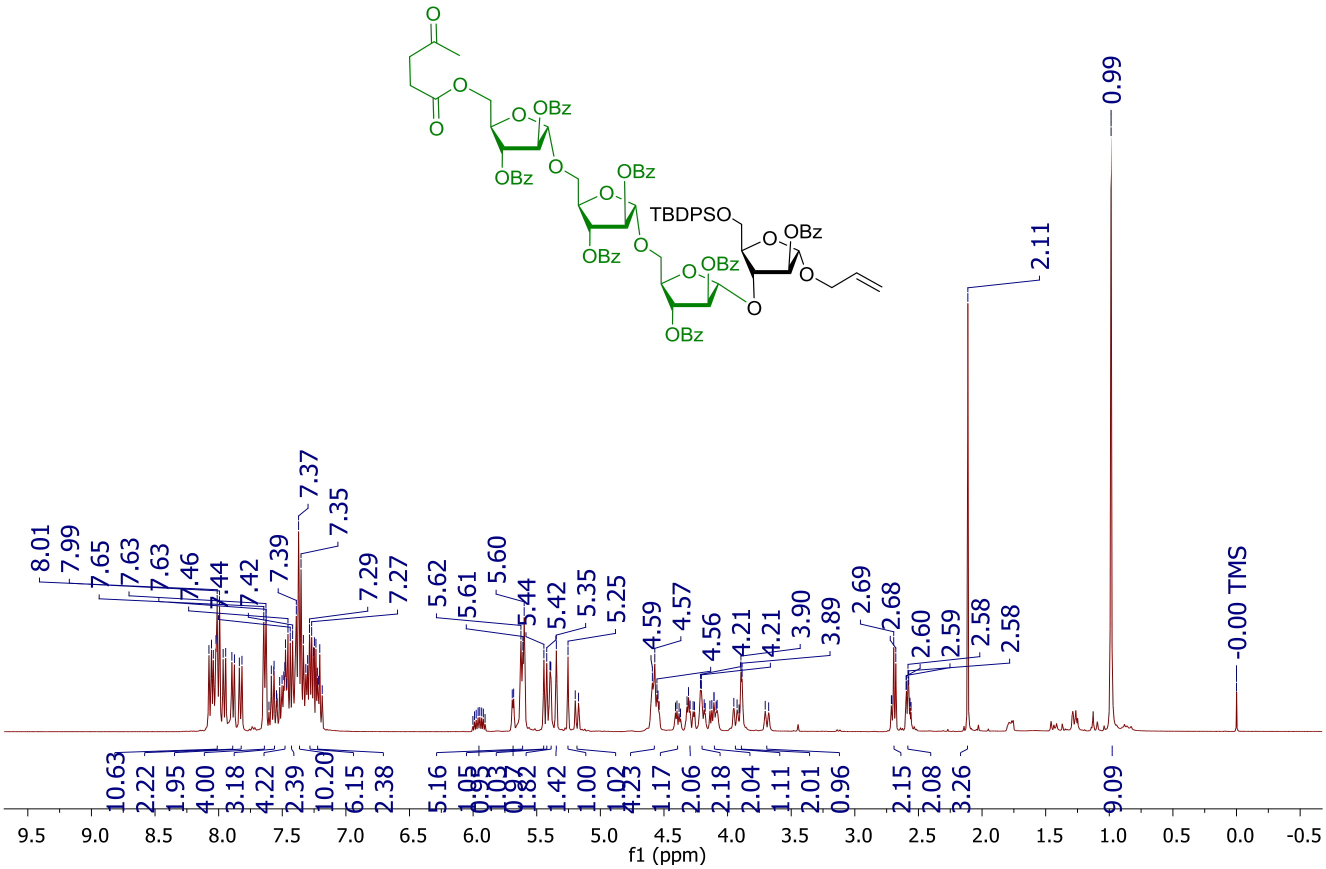

^{13}C NMR Spectrum (100.53 MHz, $CDCl_3$) of Compound 3**2**

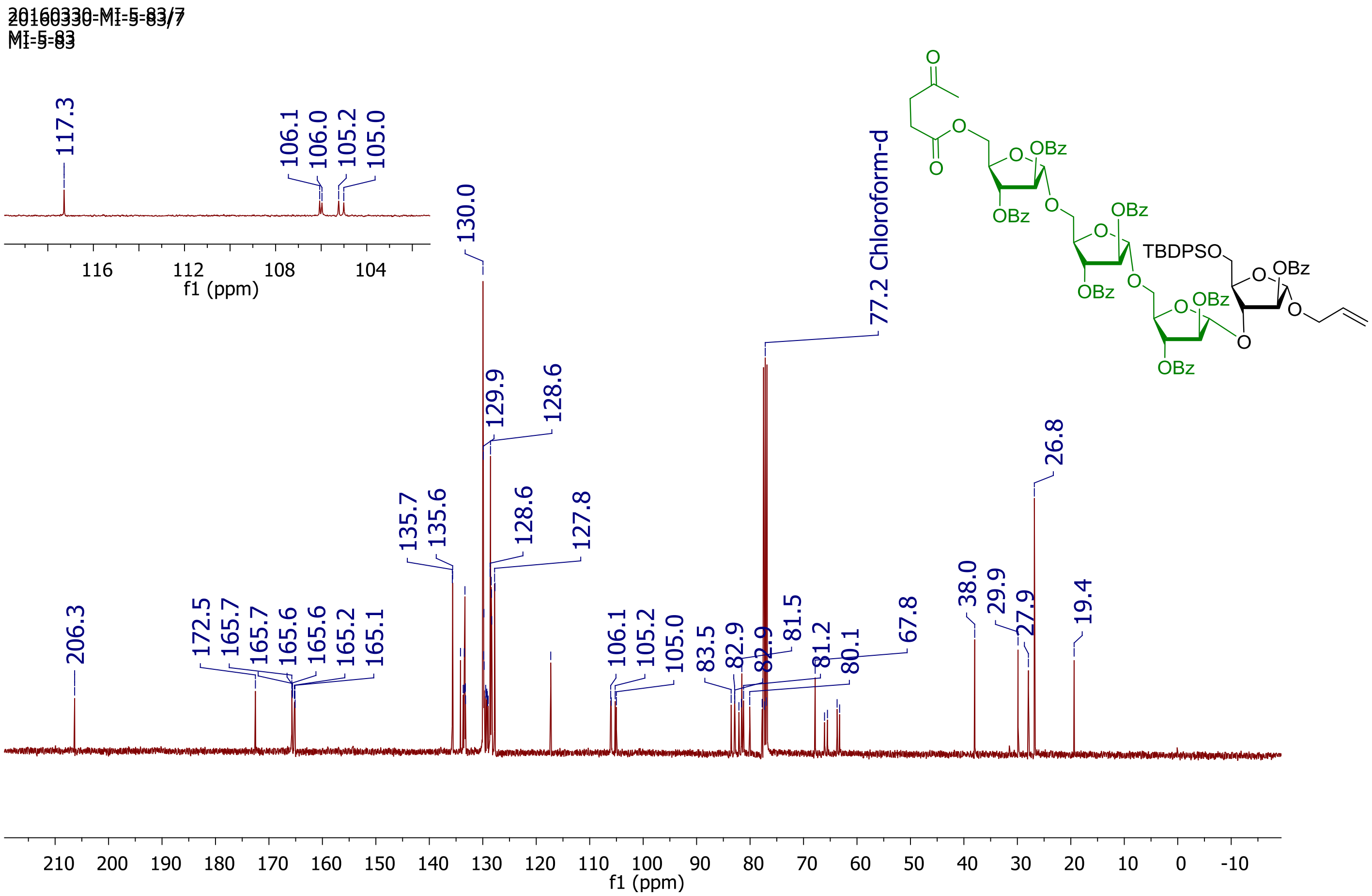

DEPT Spectrum (100.53 MHz, $CDCl_3$) of Compound 3**2**

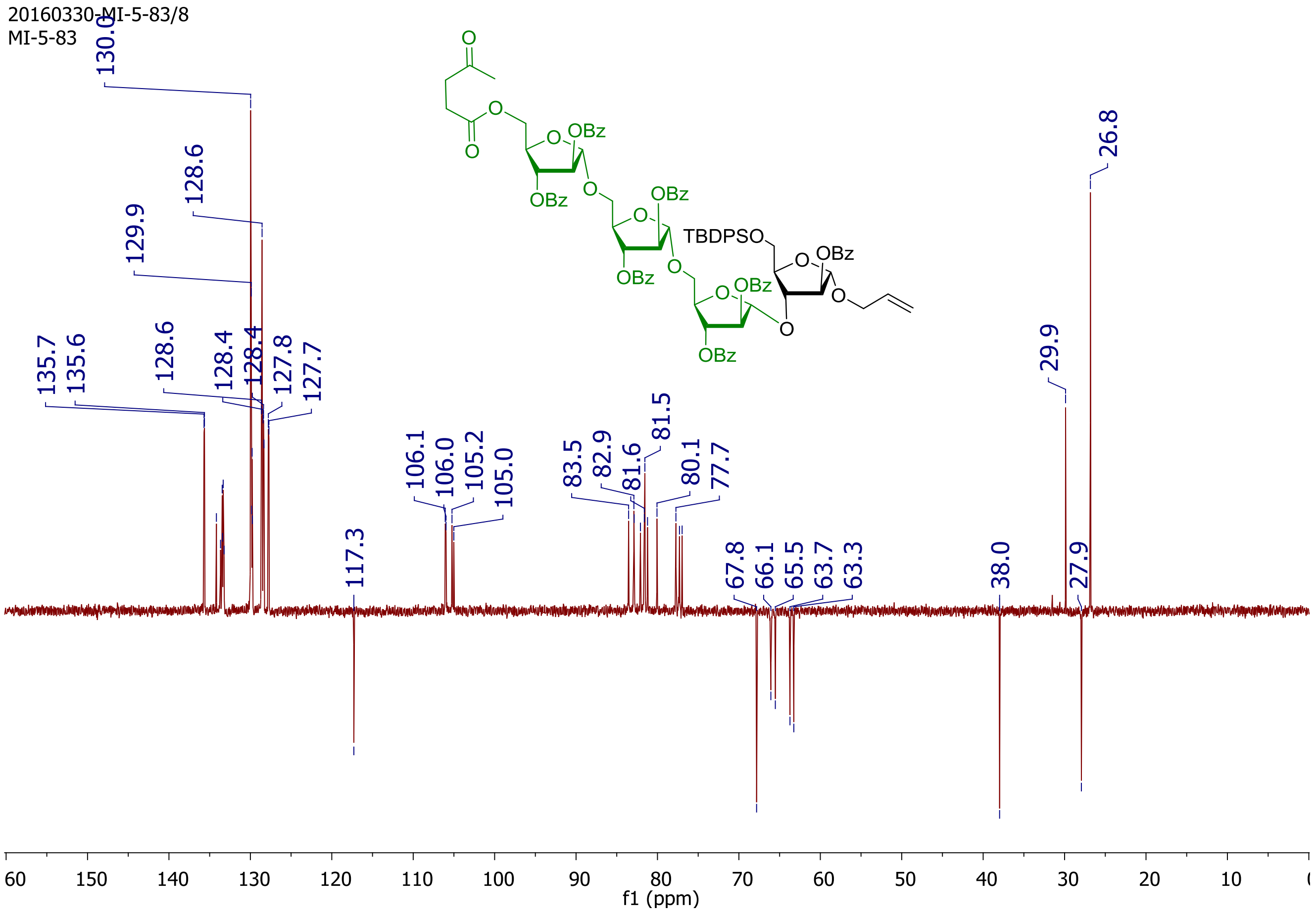

^{1}H NMR Spectrum (399.78 MHz, $CDCl_3$) of Compound **33**

20160401-MI-5-85

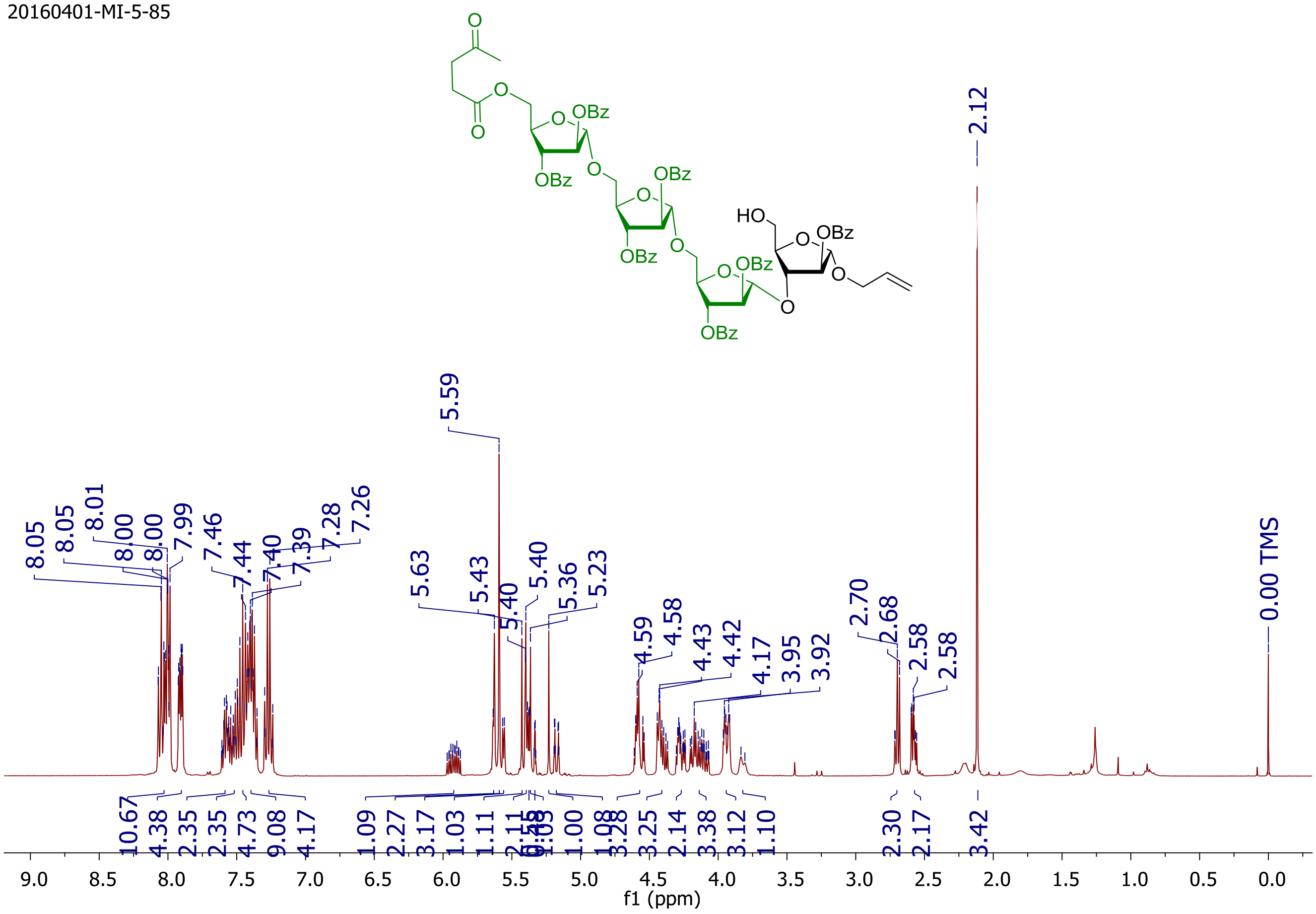

^{13}C NMR Spectrum (100.53 MHz, $CDCl_3$) of Compound **33**

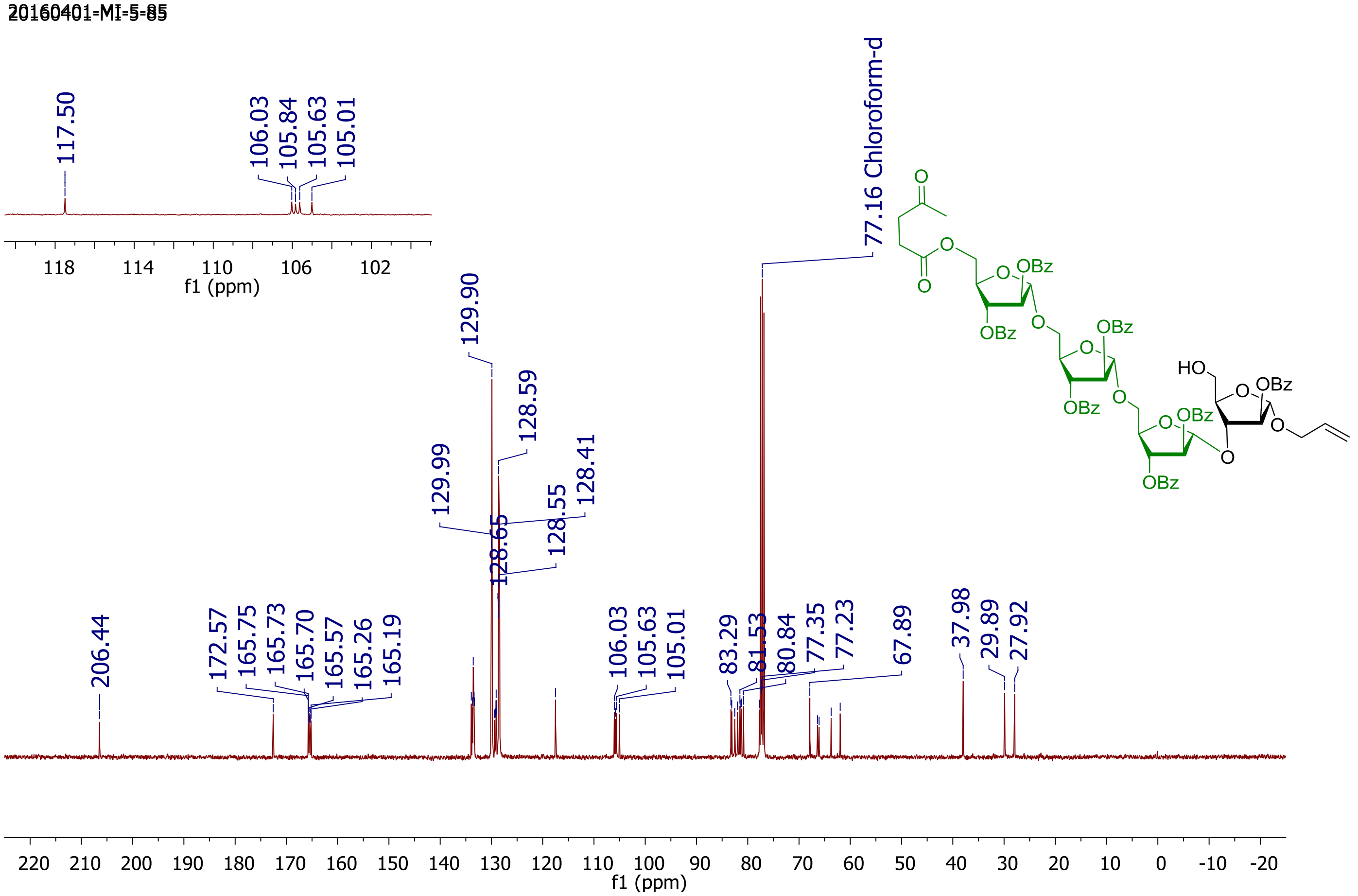

DEPT Spectrum (100.53 MHz, $CDCl_3$) of Compound **33**

20160401-MI-5-85

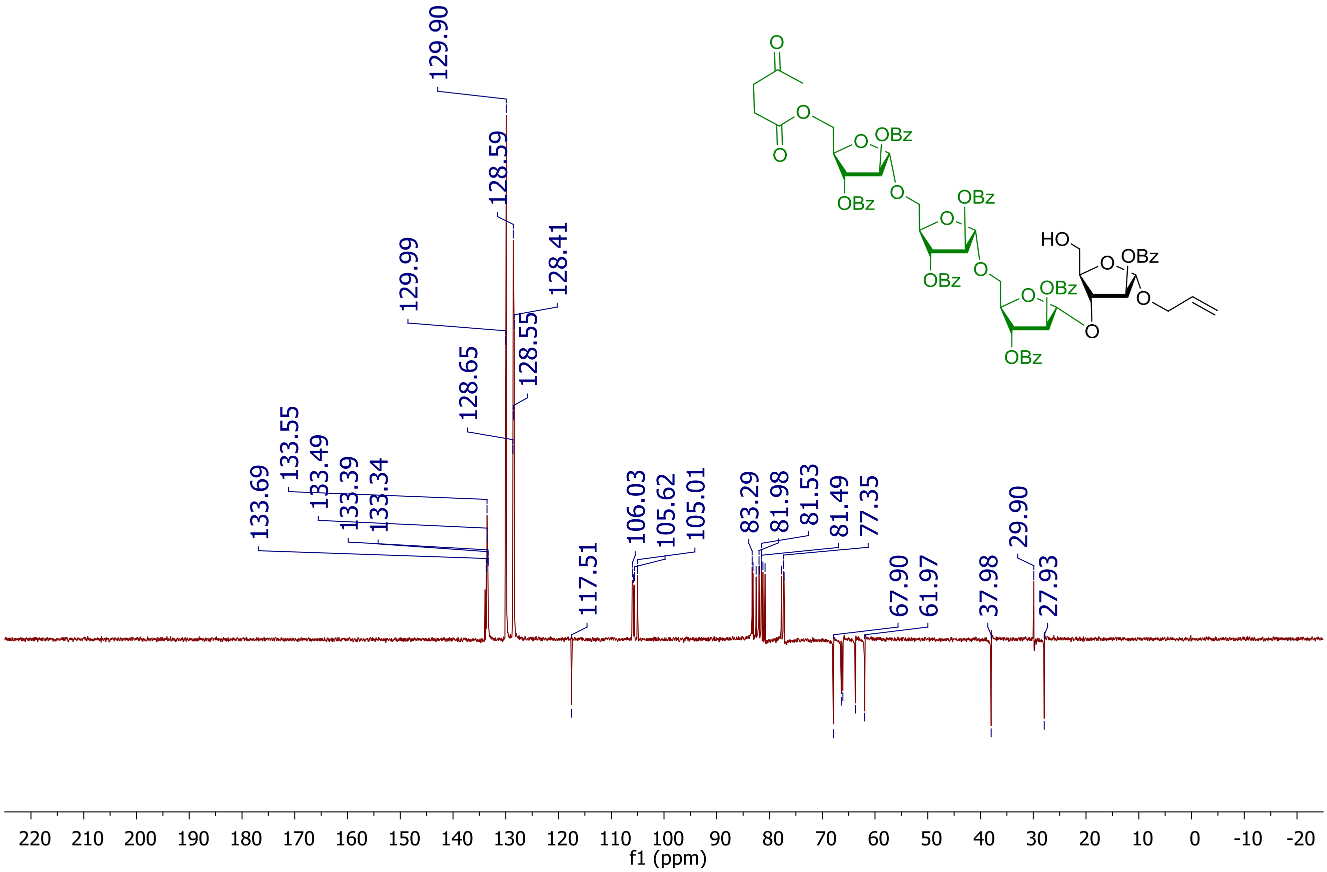

^{1}H NMR Spectrum (399.78 MHz, $CDCl_3$) of Compound **34**

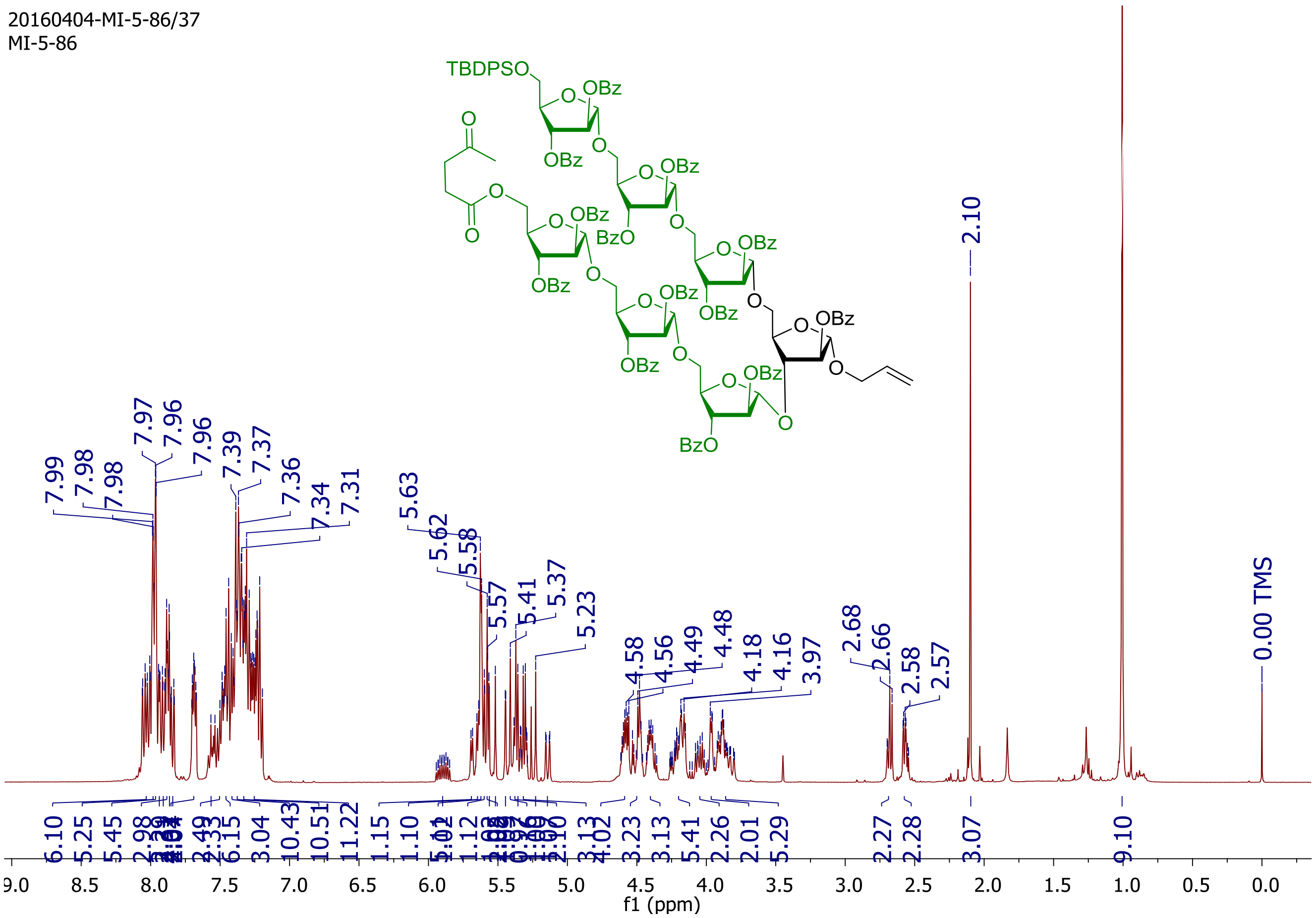

^{13}C NMR Spectrum (100.53 MHz, $CDCl_3$) of Compound **34**

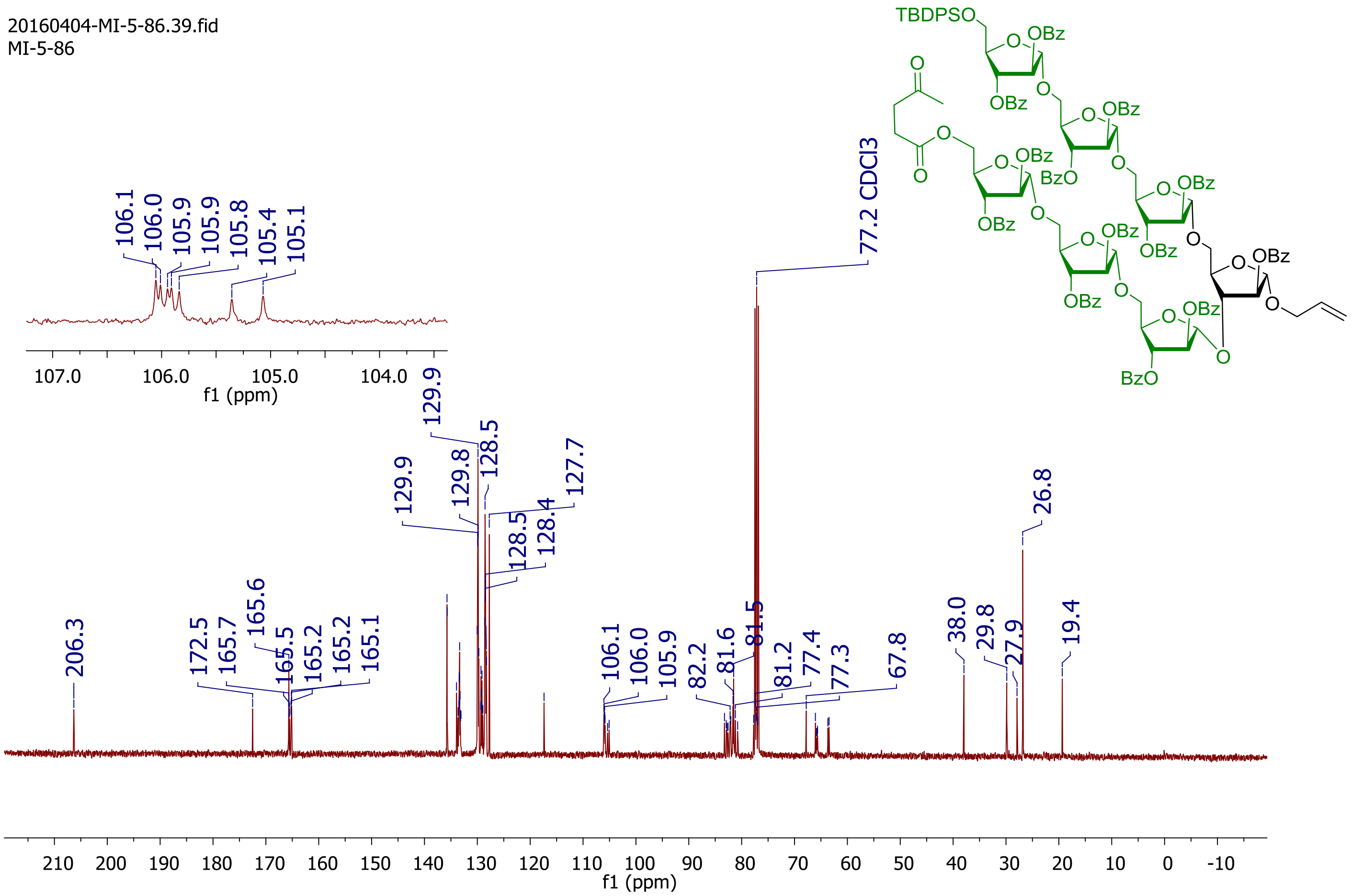

DEPT Spectrum (100.53 MHz, $CDCl_3$) of Compound **34**

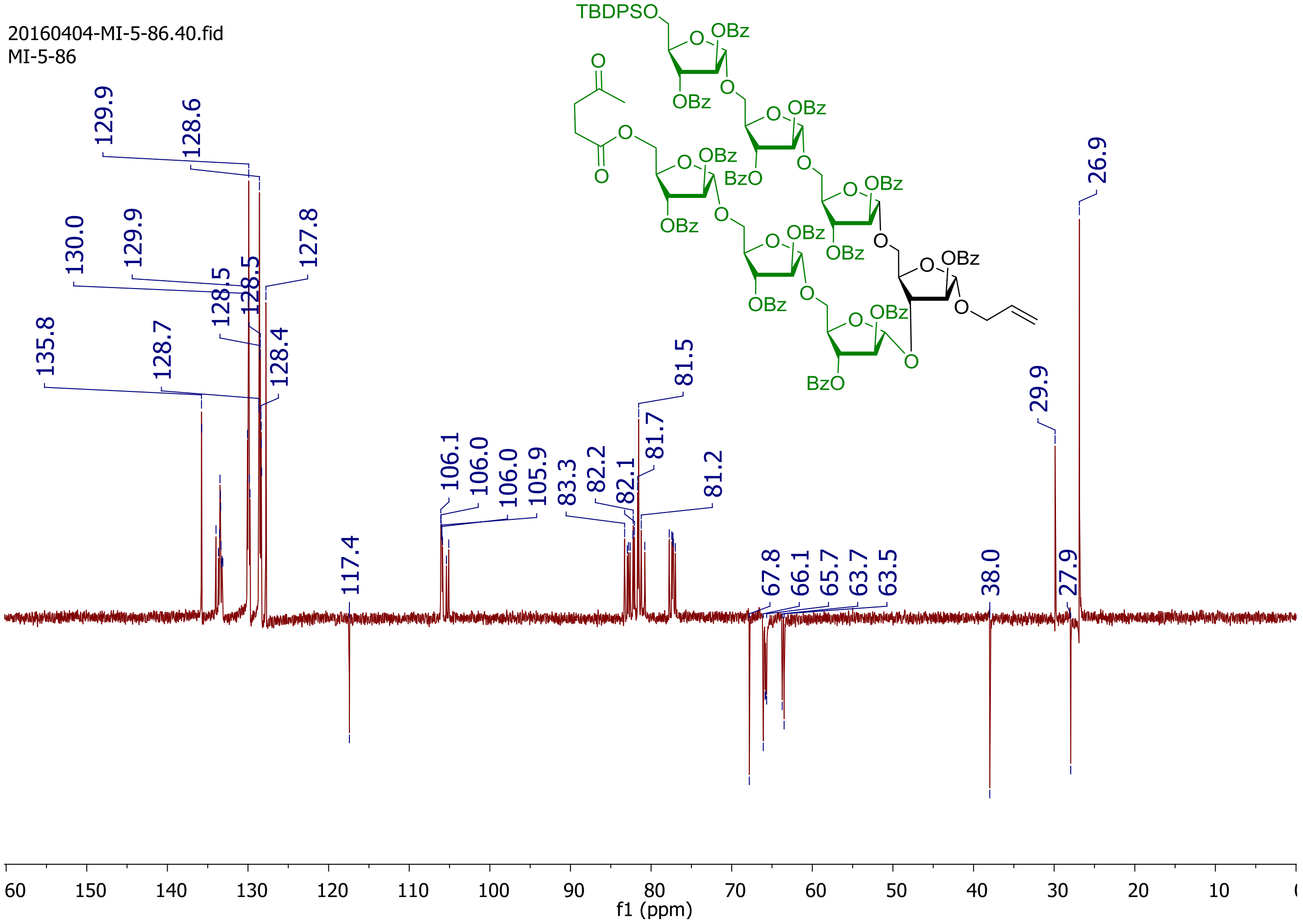

^1H NMR Spectrum (399.78 MHz, $CDCl_3$) of Compound **6**

20160513-MI-5-105A

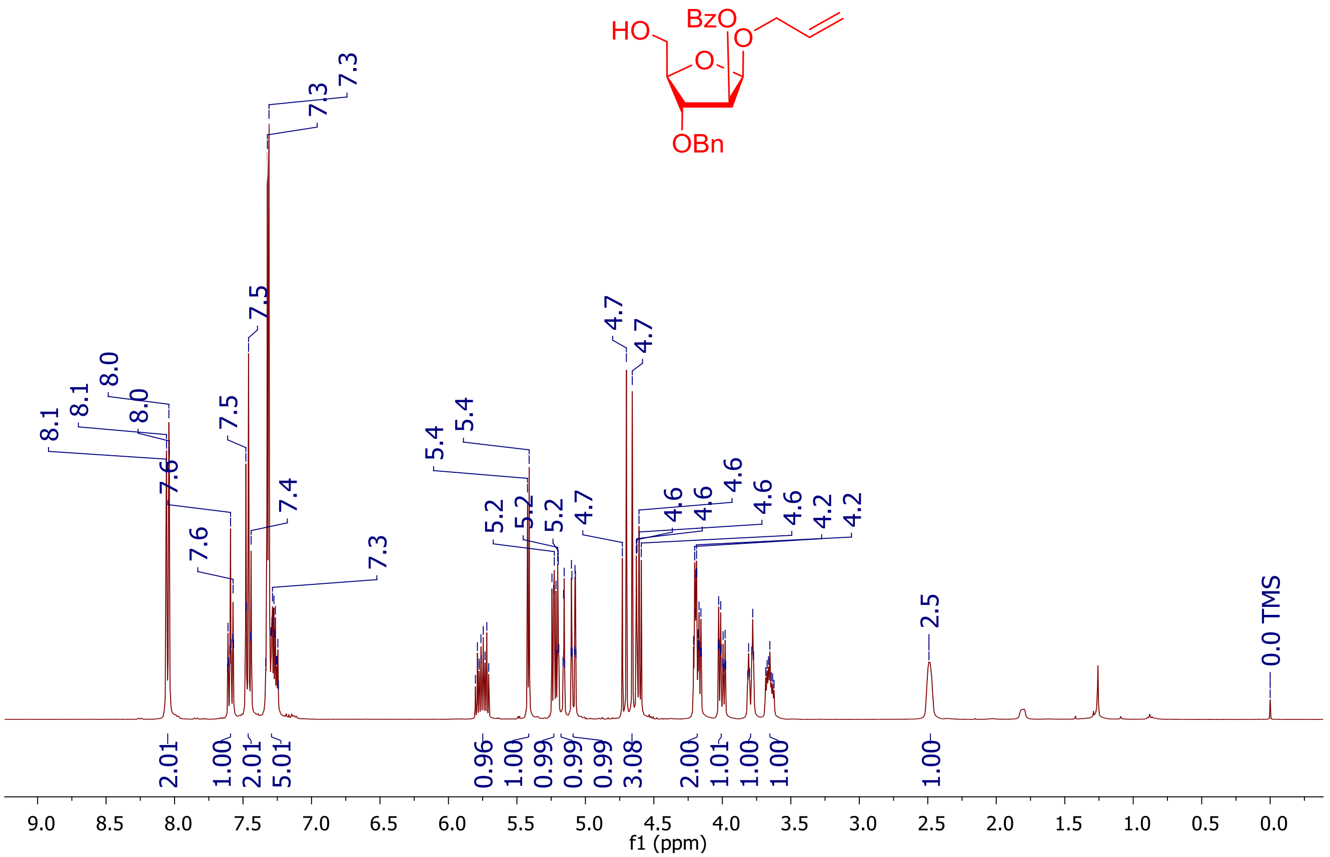

^{13}C NMR Spectrum (100.53 MHz, $CDCl_3$) of Compound **6**

20160513-MI-5-105A

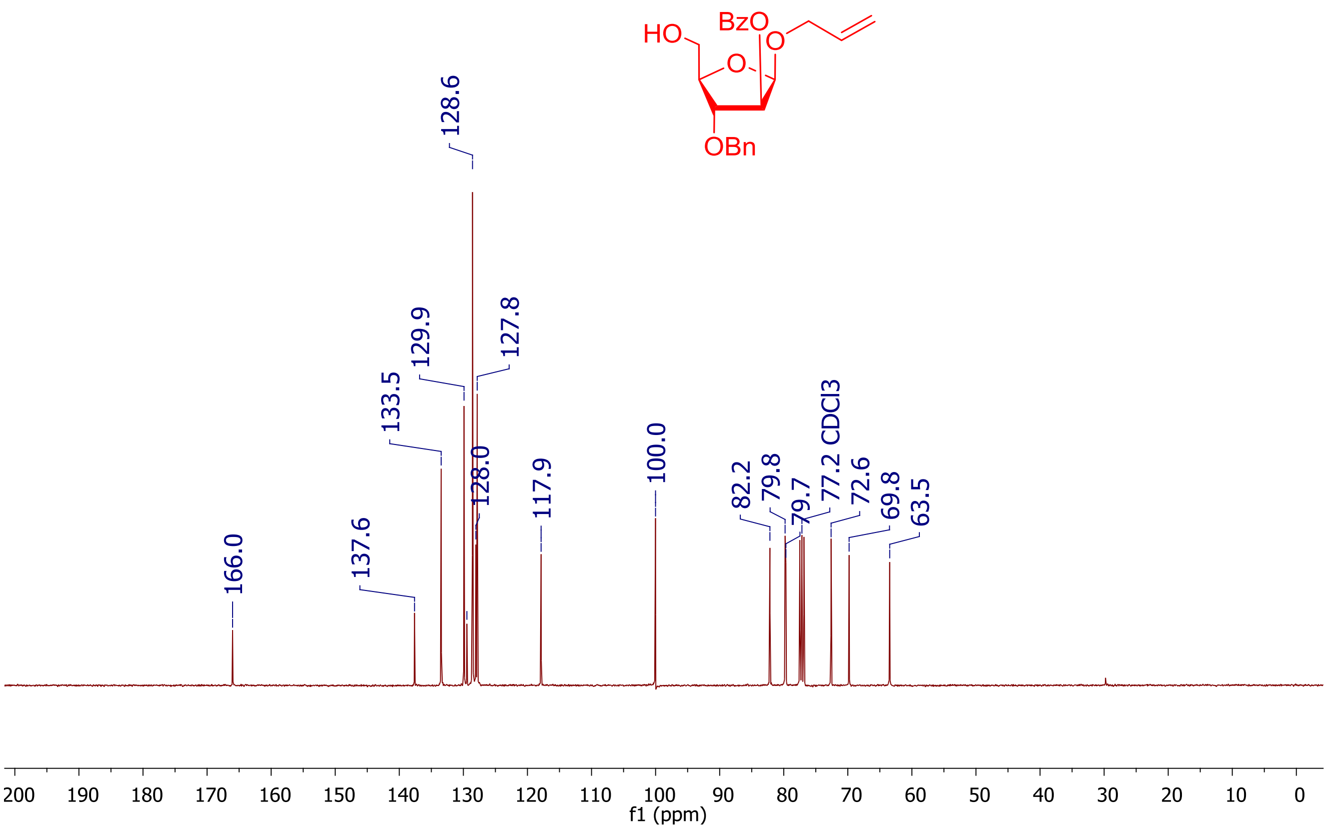

DEPT Spectrum (100.53 MHz, $CDCl_3$) of Compound **6**

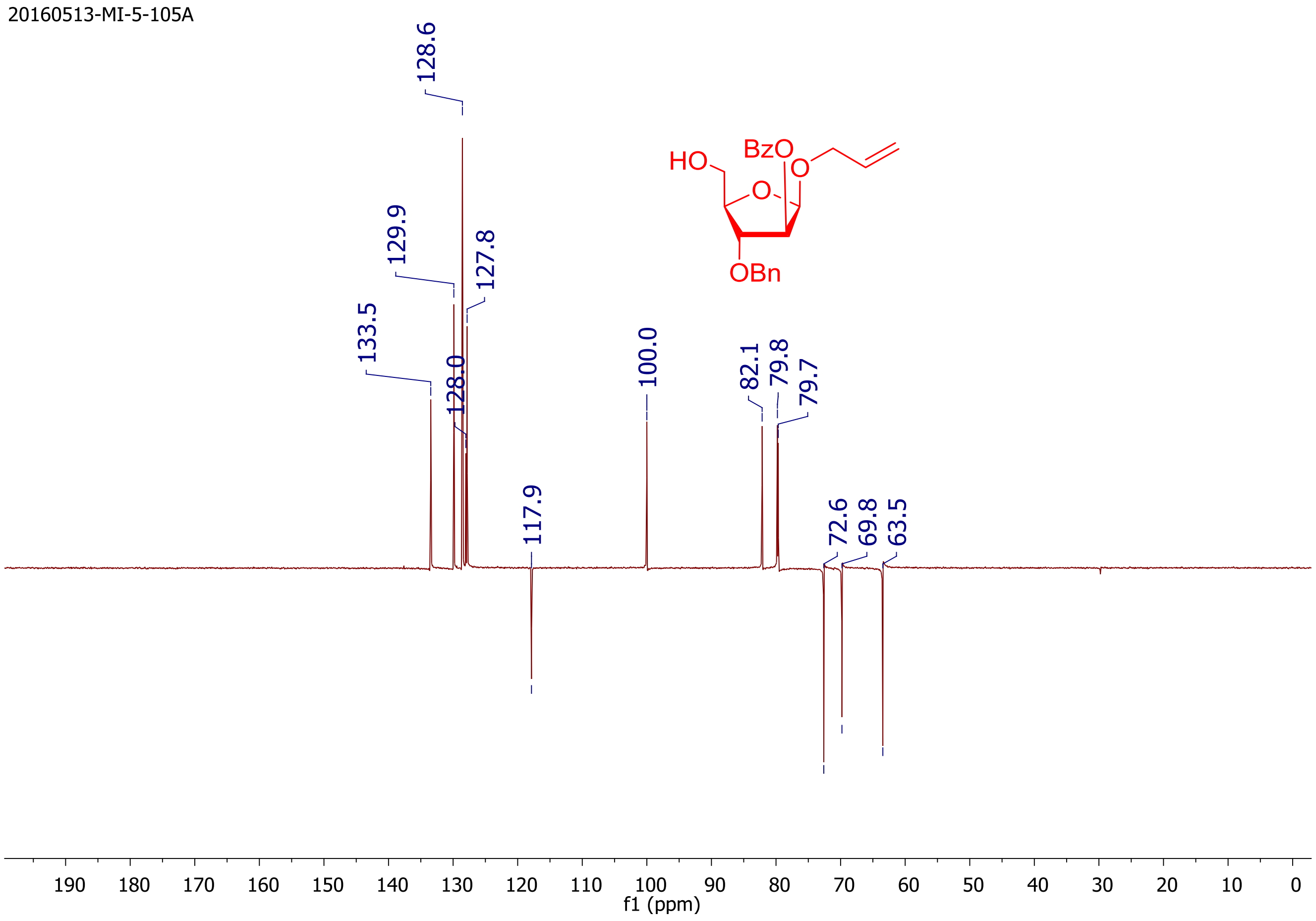

^{1}H NMR Spectrum (399.78 MHz, $CDCl_3$) of Compound **35**

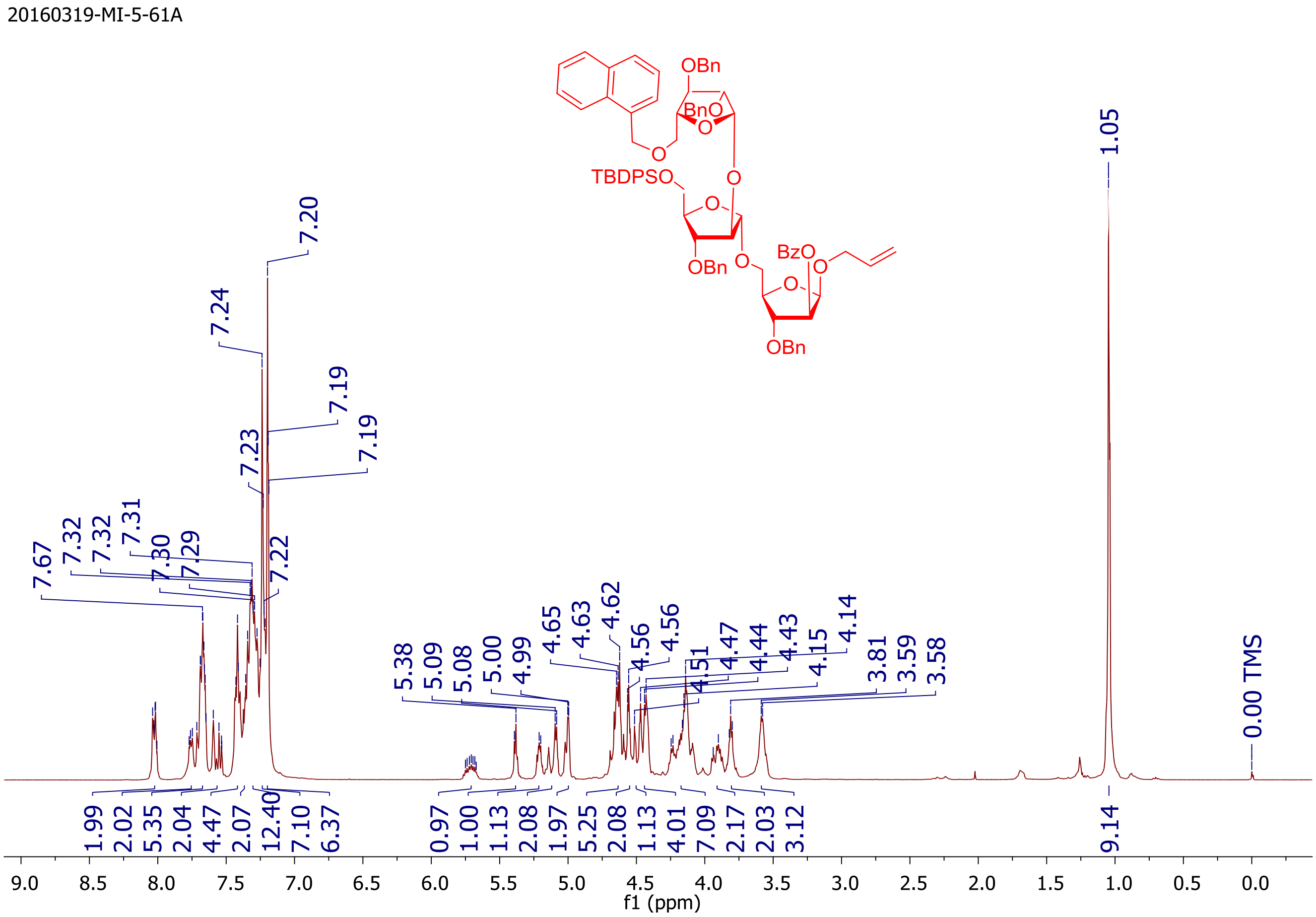

^{13}C NMR Spectrum (100.53 MHz, $CDCl_3$) of Compound **35**

20160319-MI-5-61A

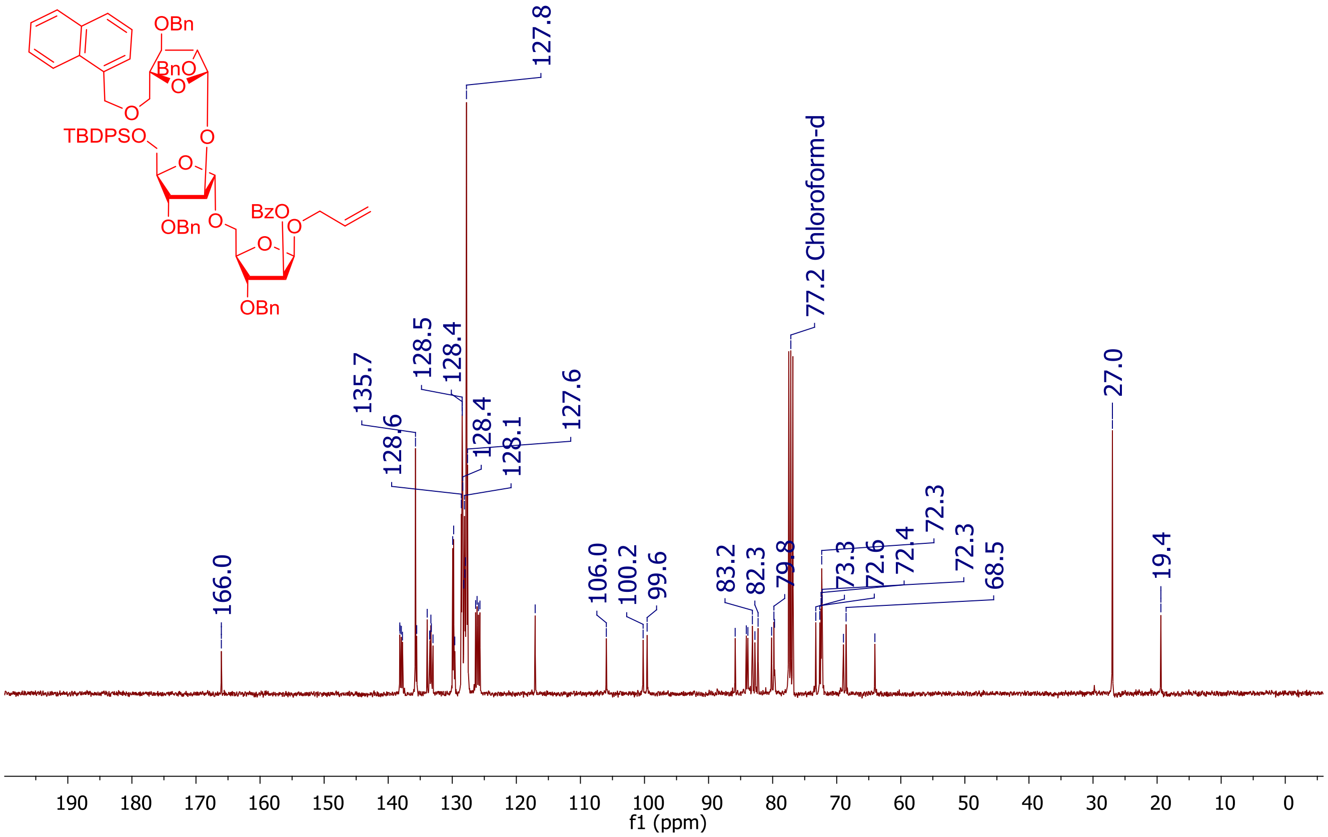

DEPT Spectrum (100.53 MHz, $CDCl_3$) of Compound **35**

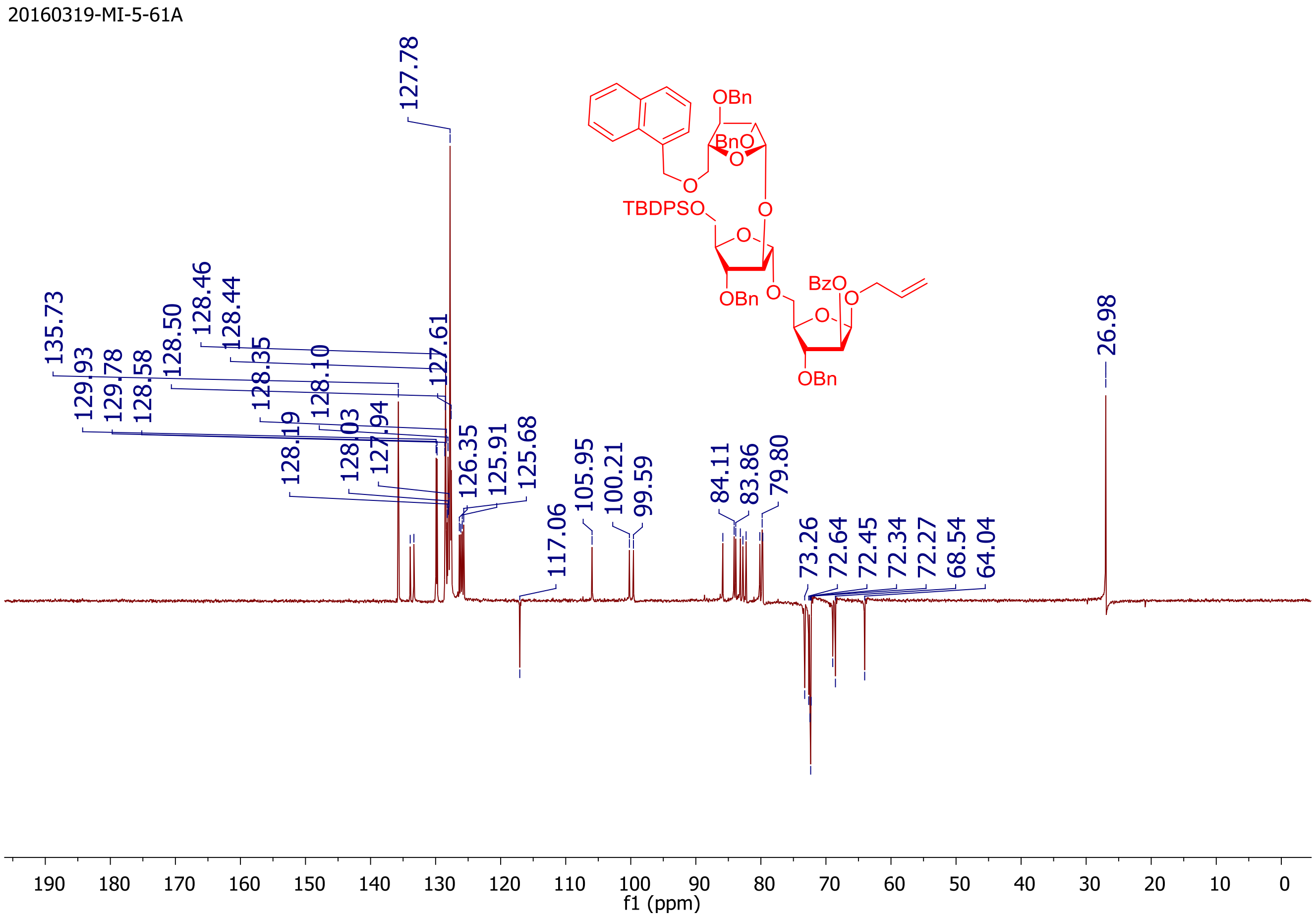

^{1}H NMR Spectrum (399.78 MHz, $CDCl_3$) of Compound **37**

20160326-MI-5-75/1
MI-5-75

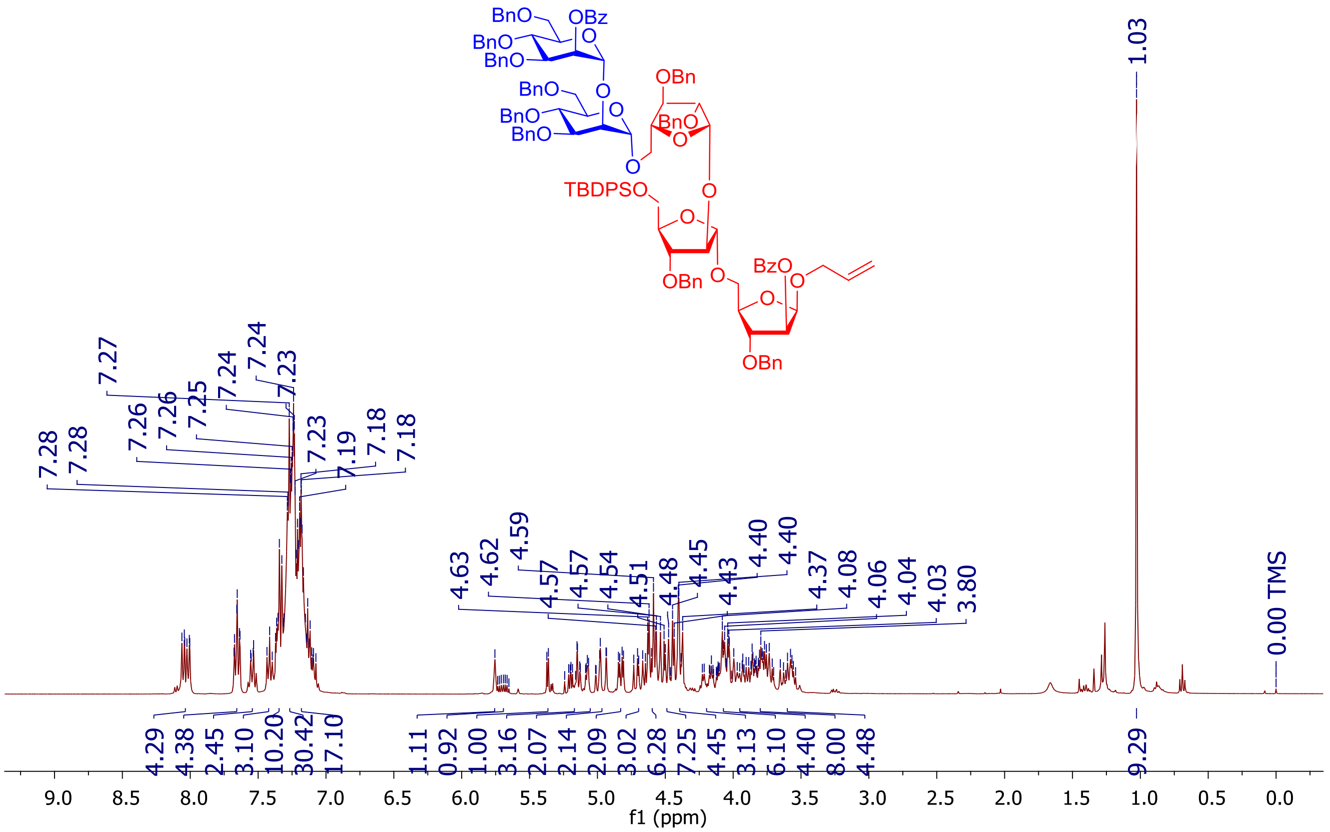

^{13}C NMR Spectrum (100.53 MHz, $CDCl_3$) of Compound **37**

20160326-MI-5-75/2
MI-5-75

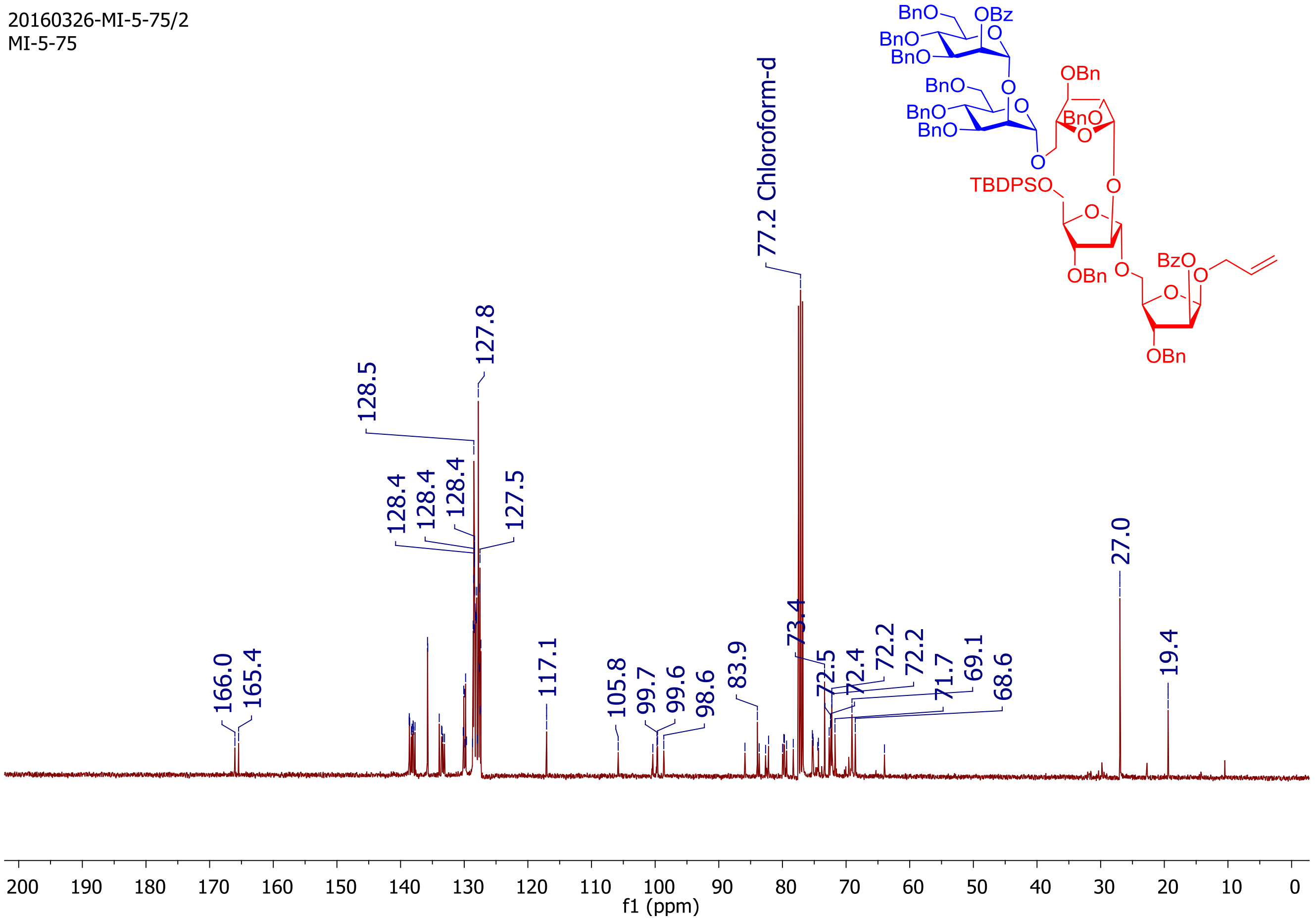

DEPT Spectrum (100.53 MHz, $CDCl_3$) of Compound **37**

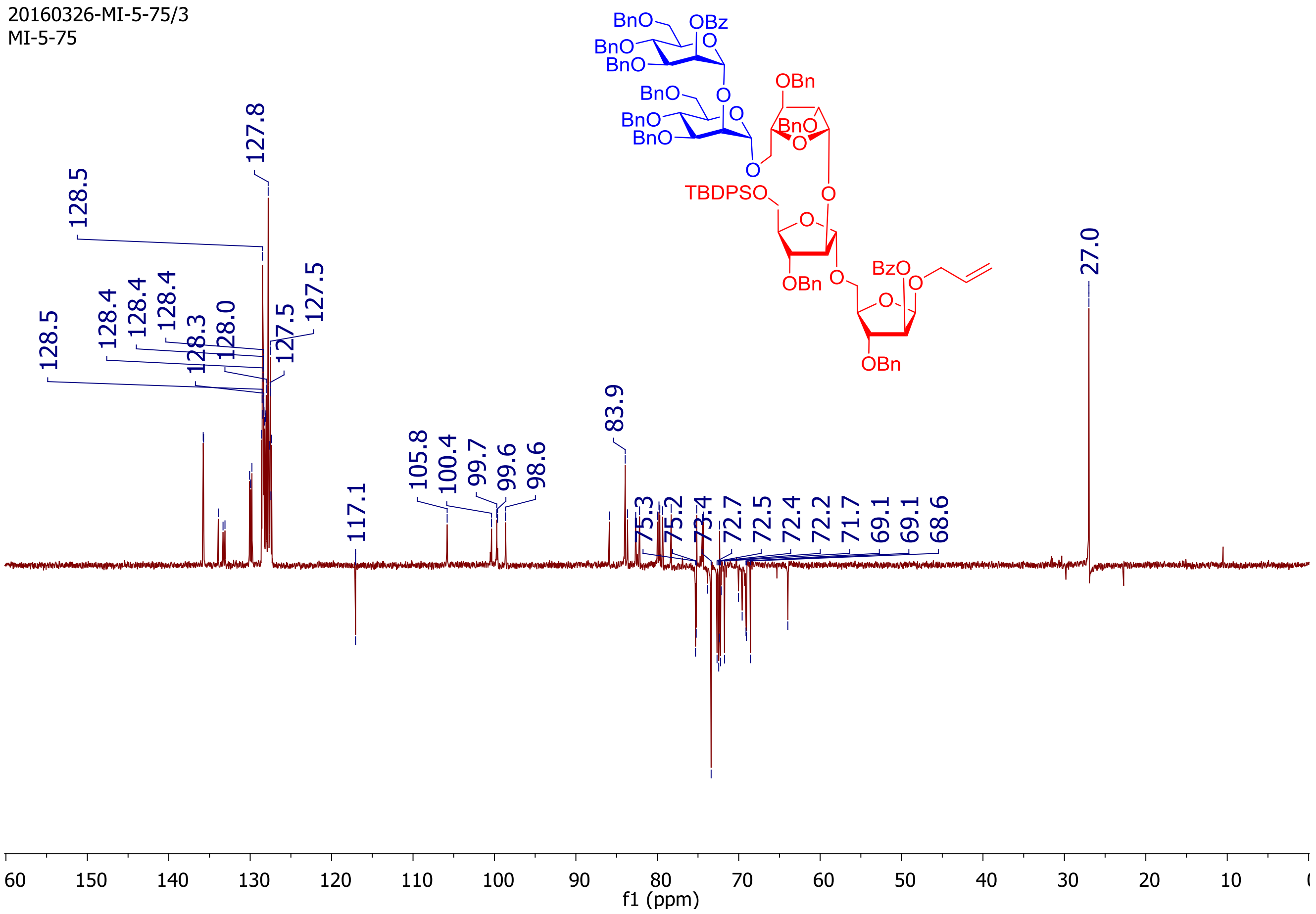

^{1}H NMR Spectrum (399.78 MHz, CDCl$_3$) of Compound **38**

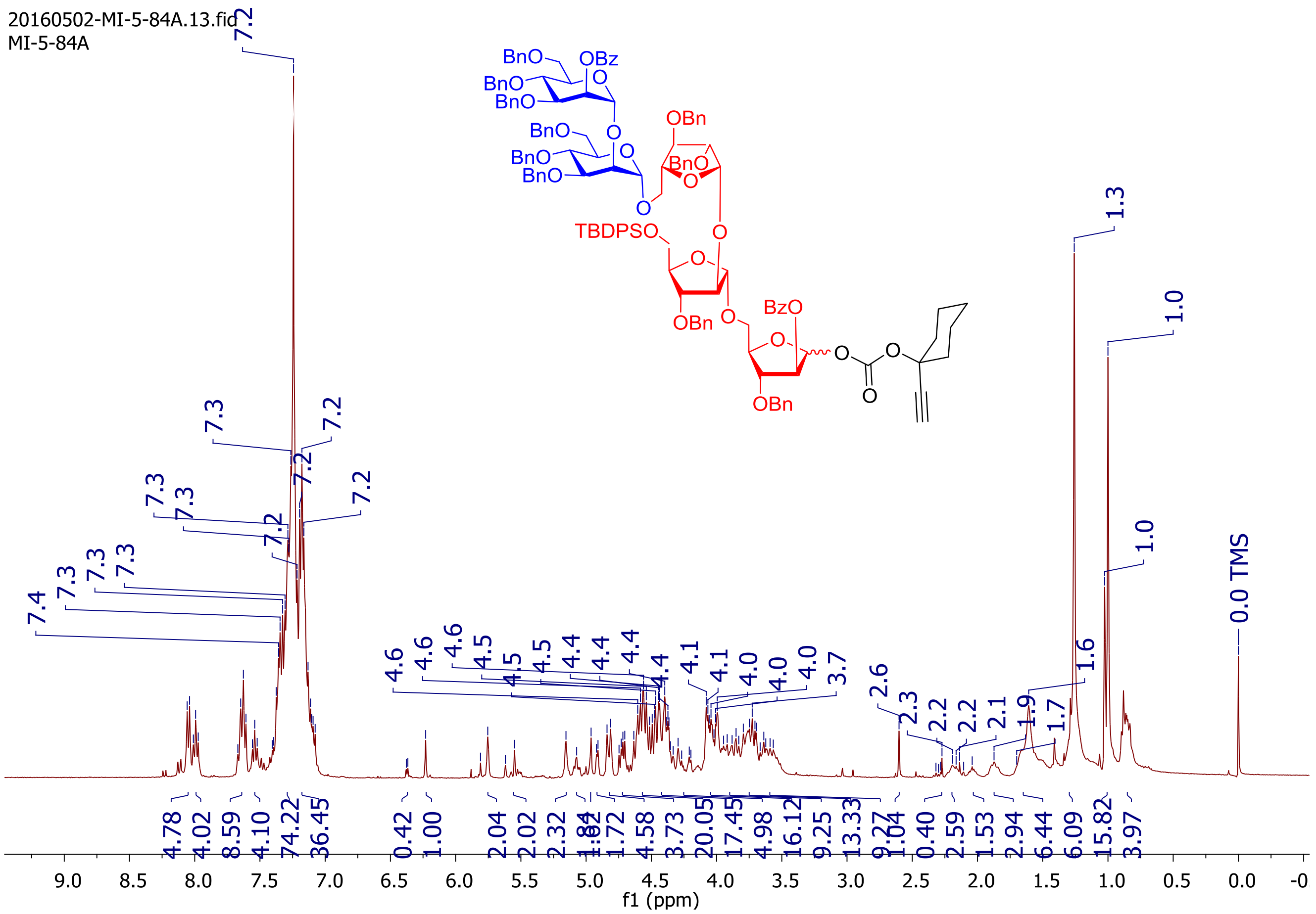

^{13}C NMR Spectrum (100.53 MHz, $CDCl_3$) of Compound **38**

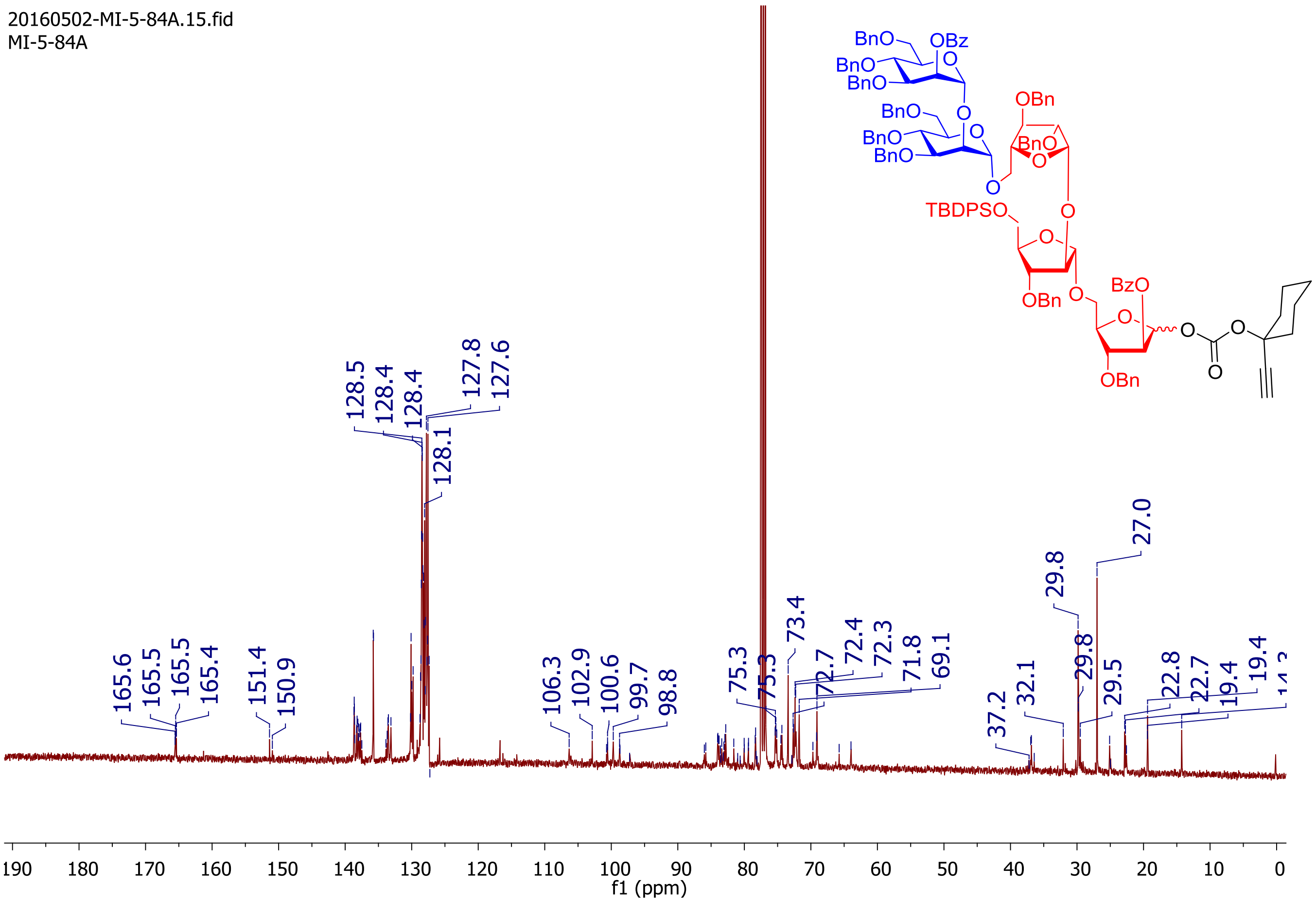

DEPT Spectrum (100.53 MHz, $CDCl_3$) of Compound **38**

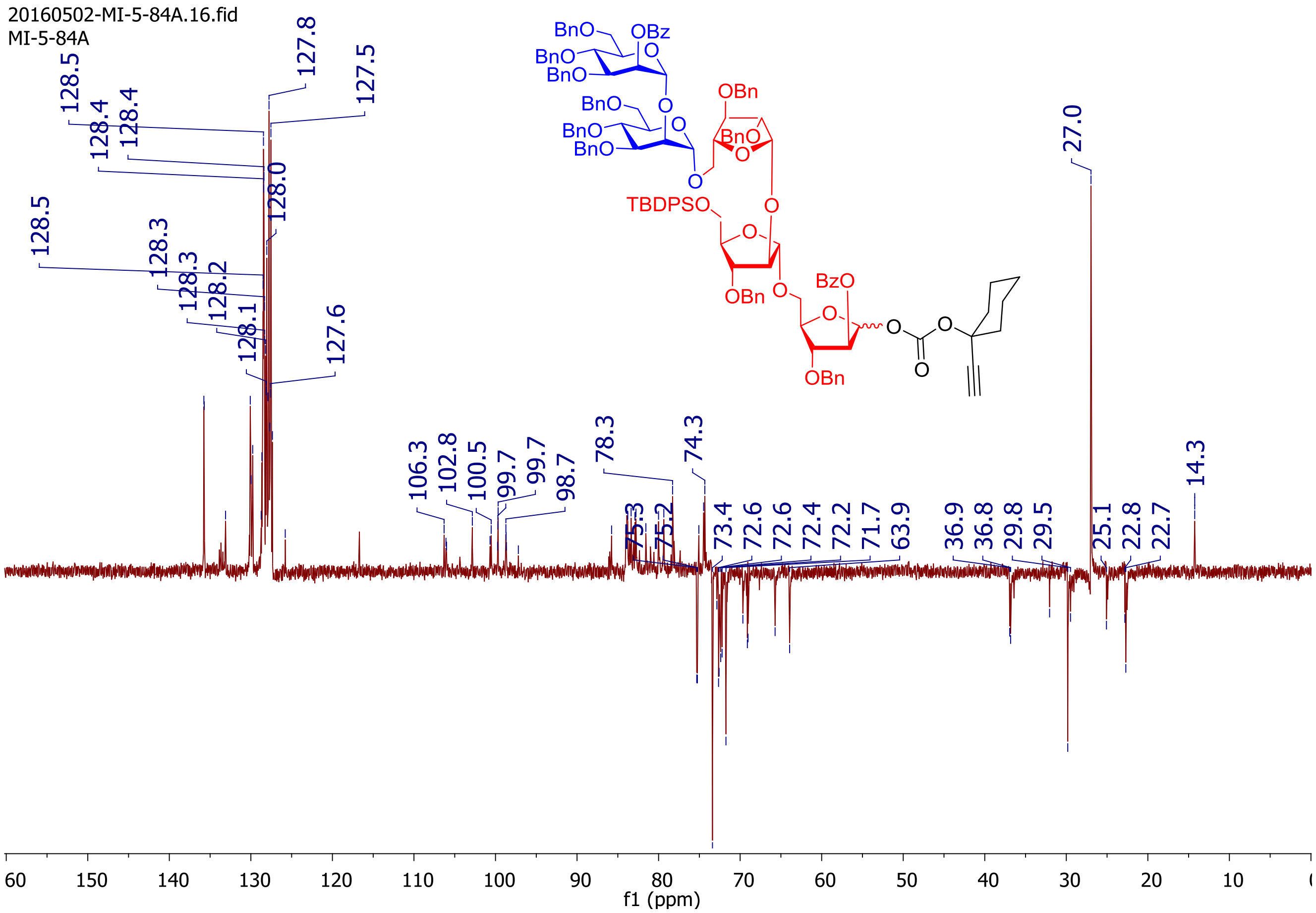

^{1}H NMR Spectrum (399.78 MHz, $CDCl_3$) of Compound **39**

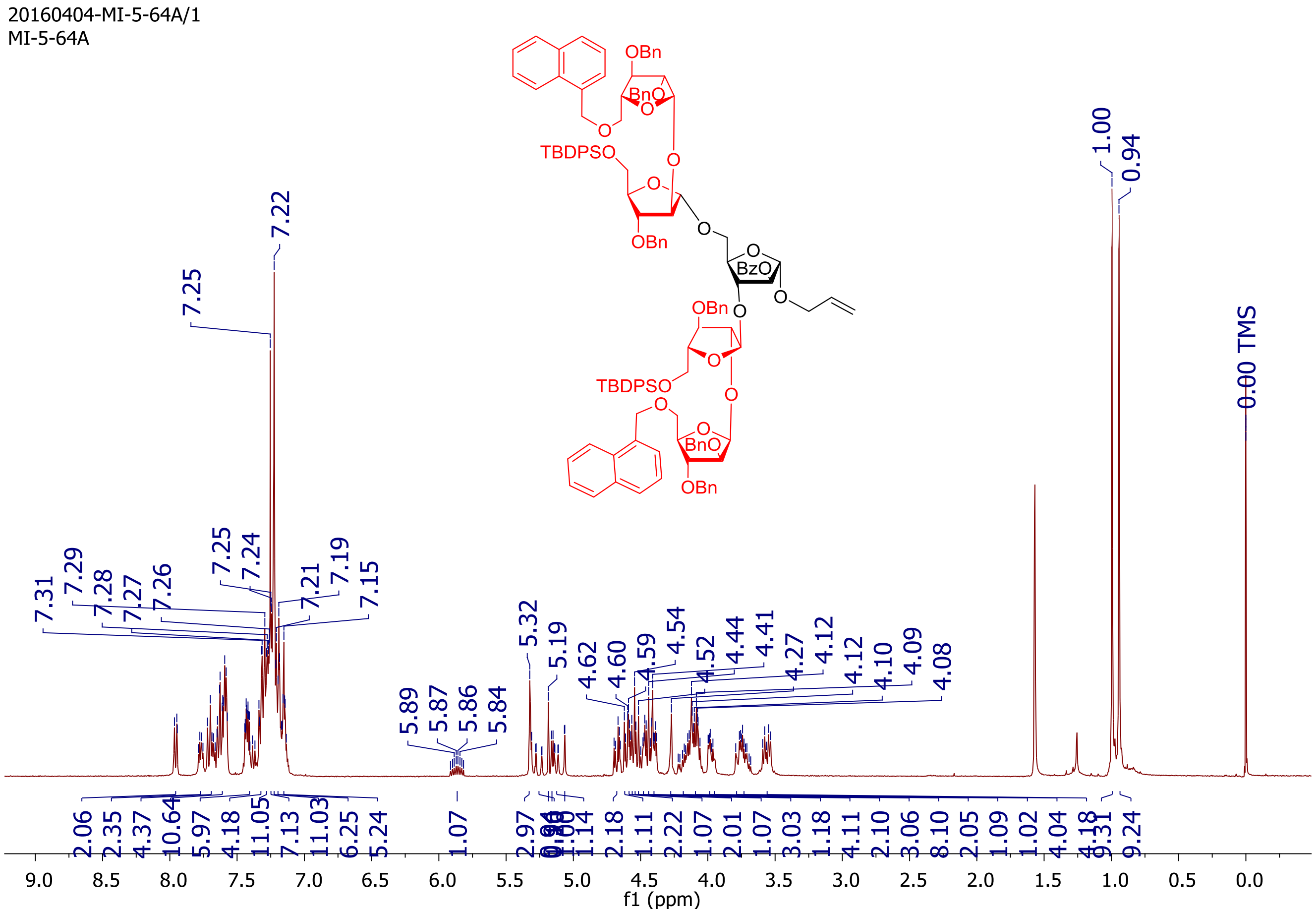

^{13}C NMR Spectrum (100.53 MHz, $CDCl_3$) of Compound **39**

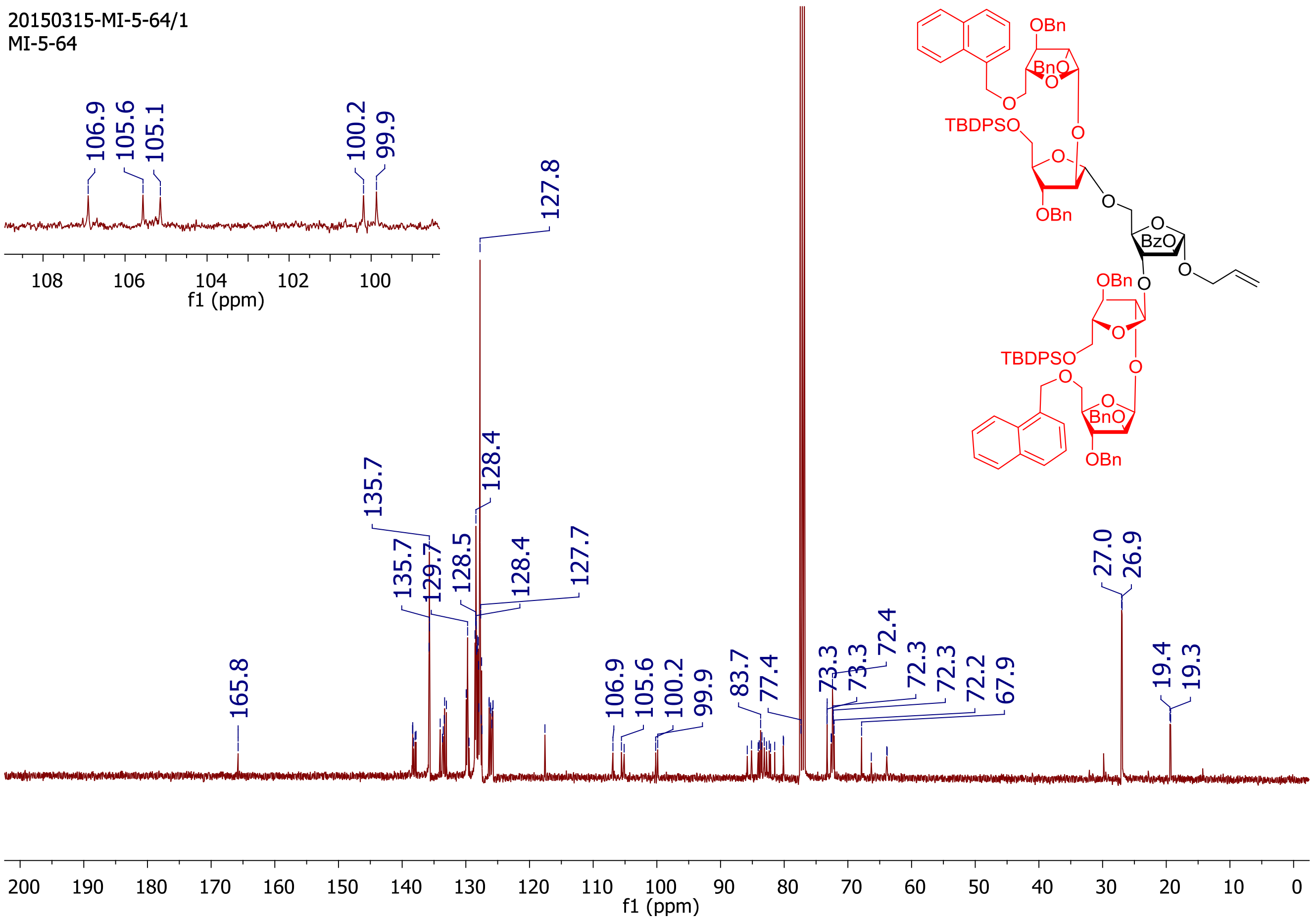

DEPT Spectrum (100.53 MHz, $CDCl_3$) of Compound **39**

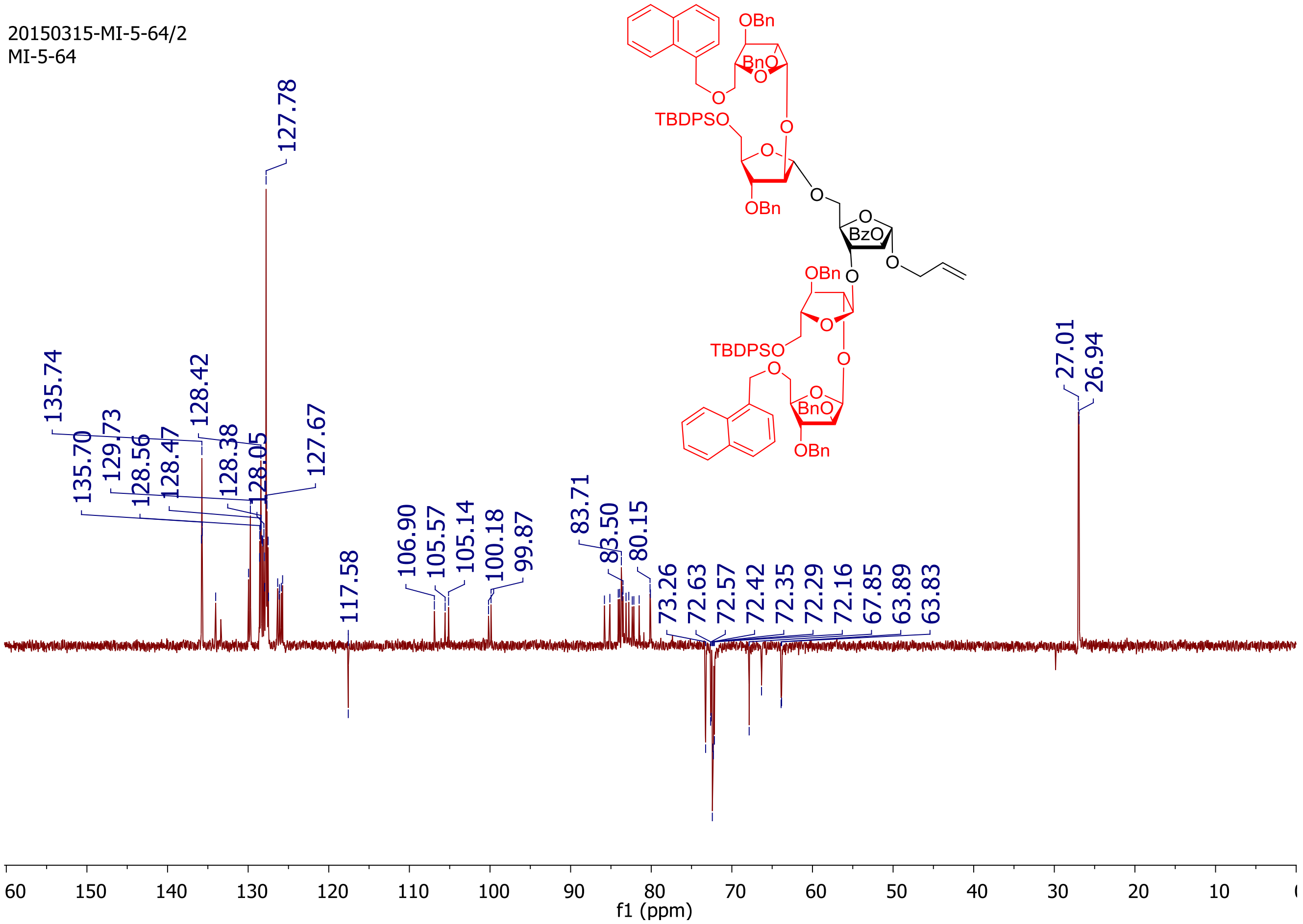

^{1}H-^{1}H COSY Spectrum (399.78 MHz, $CDCl_3$) of Compound **39**

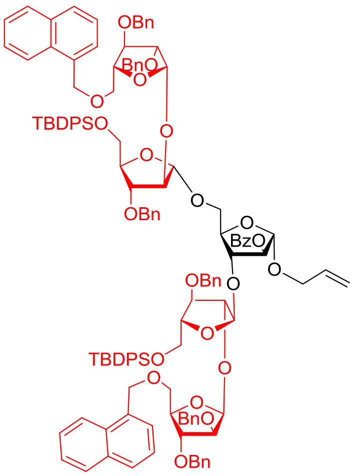

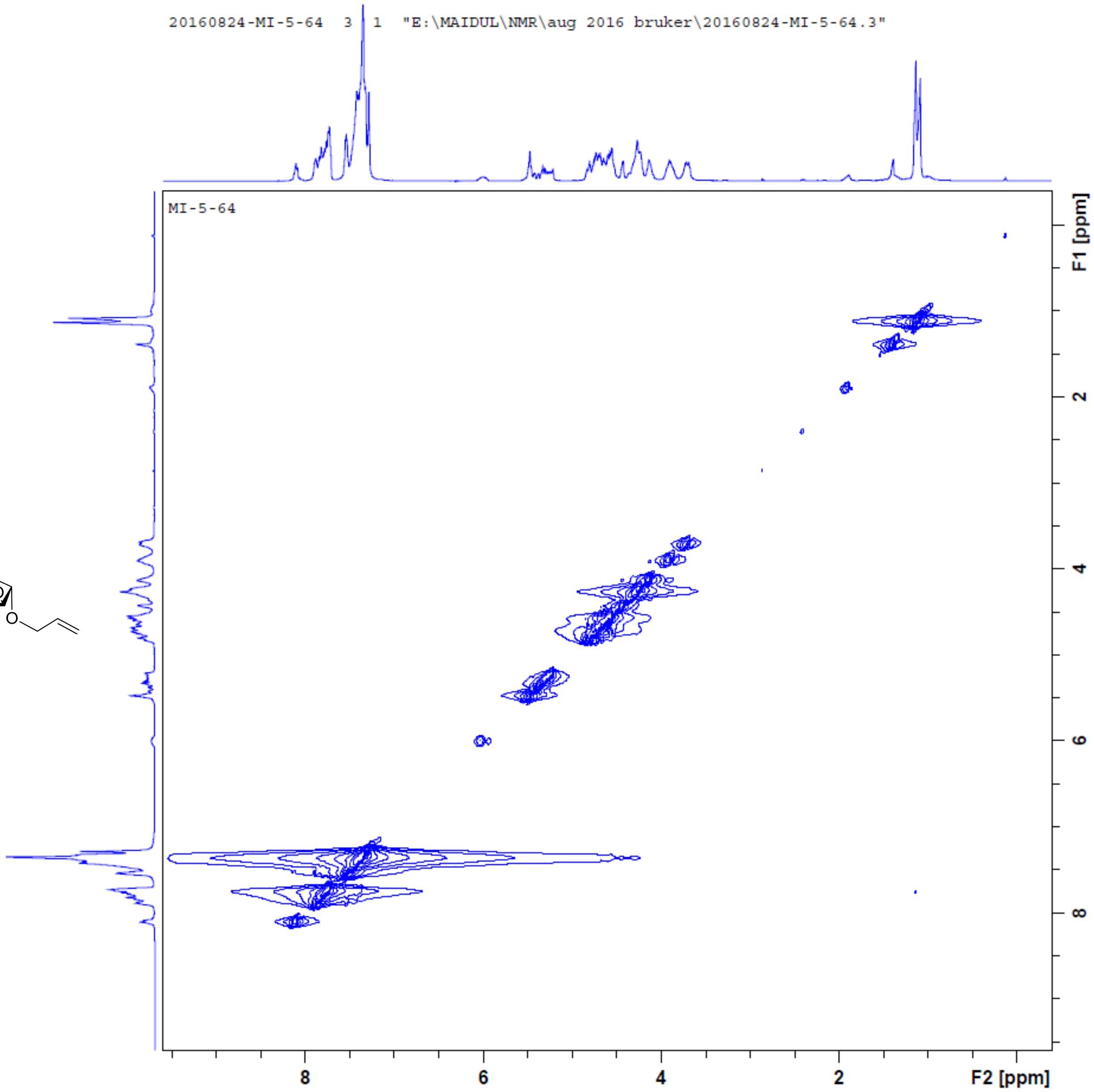

^{1}H-^{13}C HMBC Spectrum (399.78/100.53 MHz, $CDCl_3$) of Compound **39**

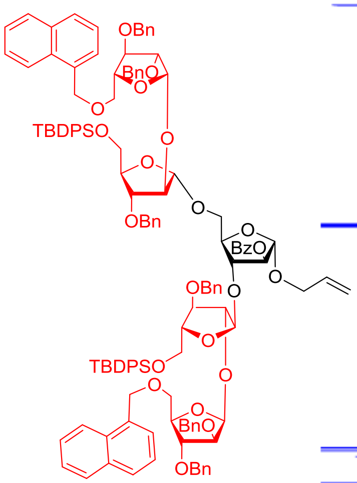

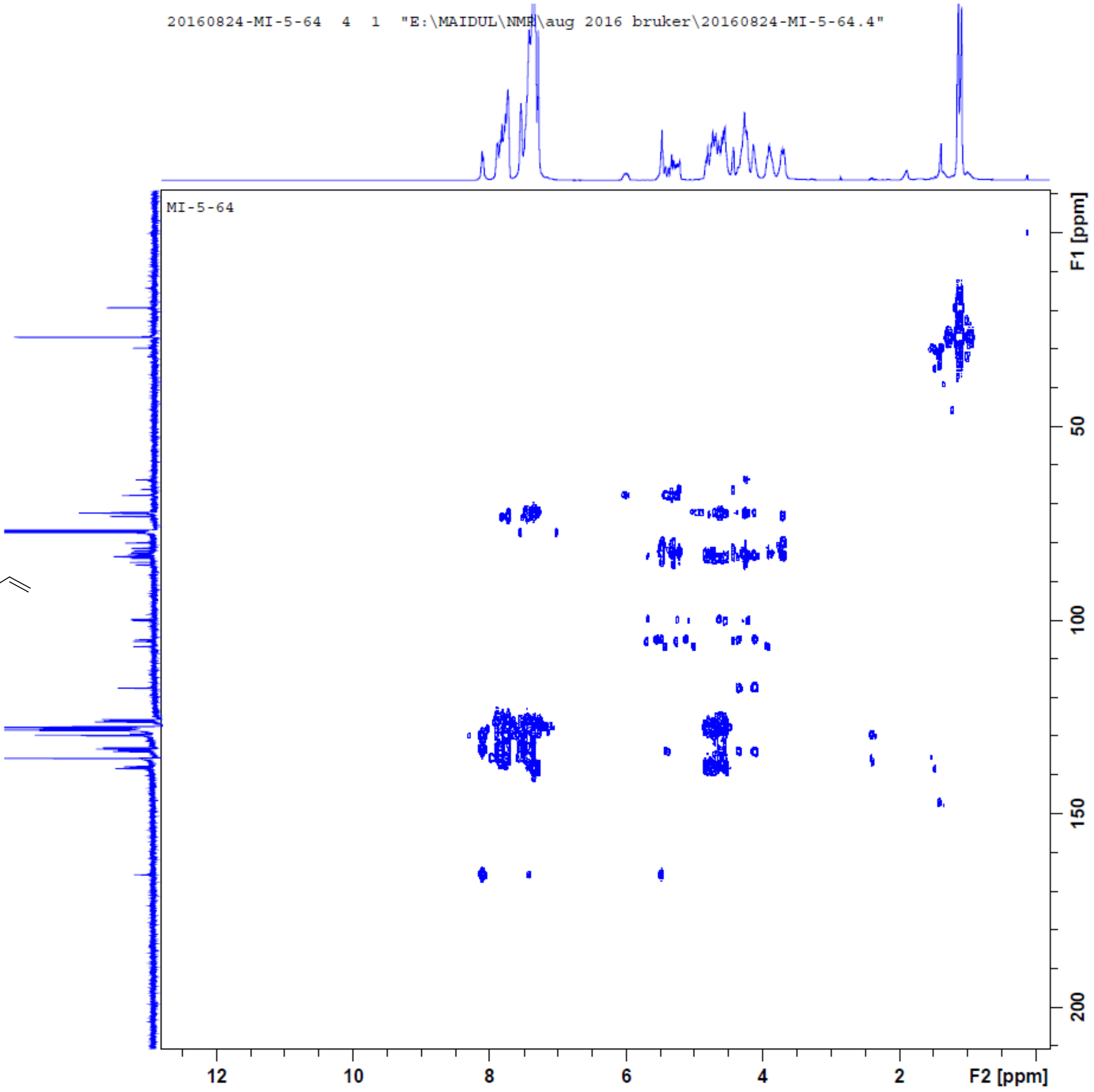

gHSQC Spectrum (399.78/100.53 MHz, $CDCl_3$) of Compound **39**

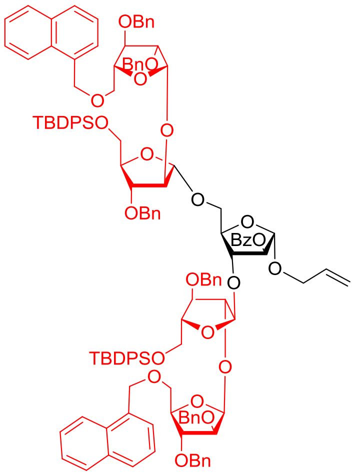

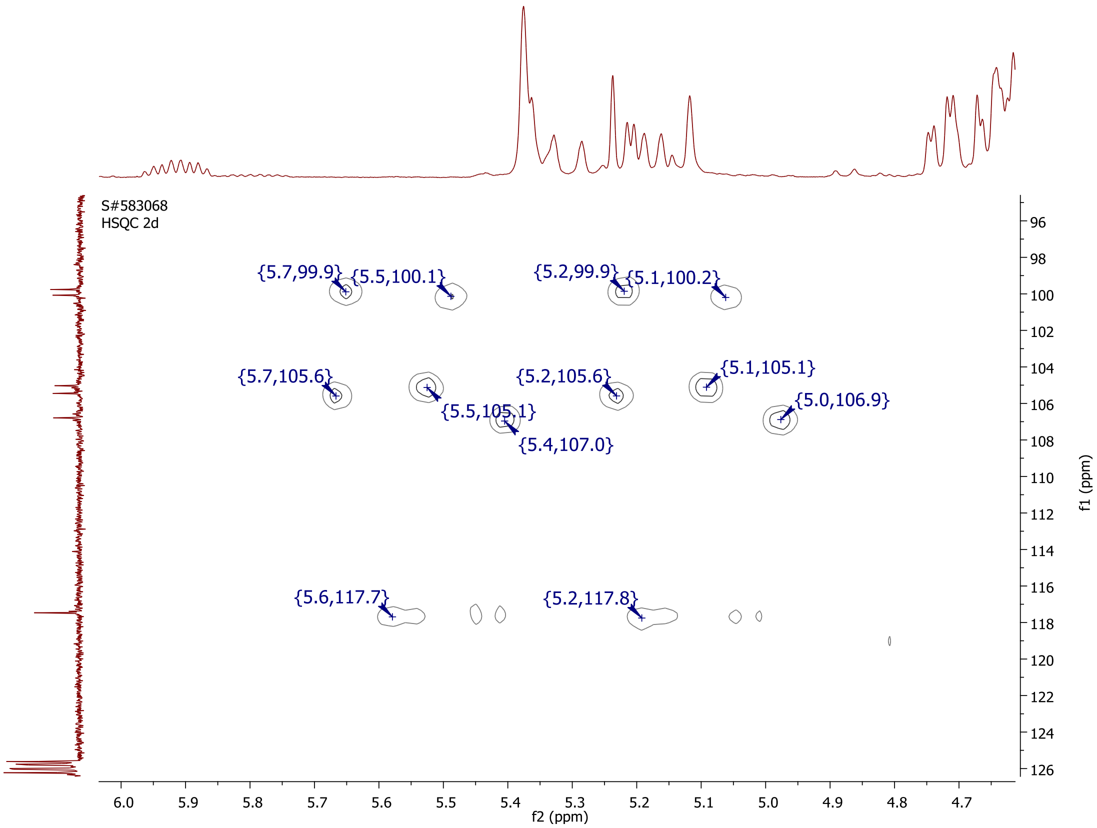

^{1}H NMR Spectrum (399.78 MHz, $CDCl_3$) of Compound **40**

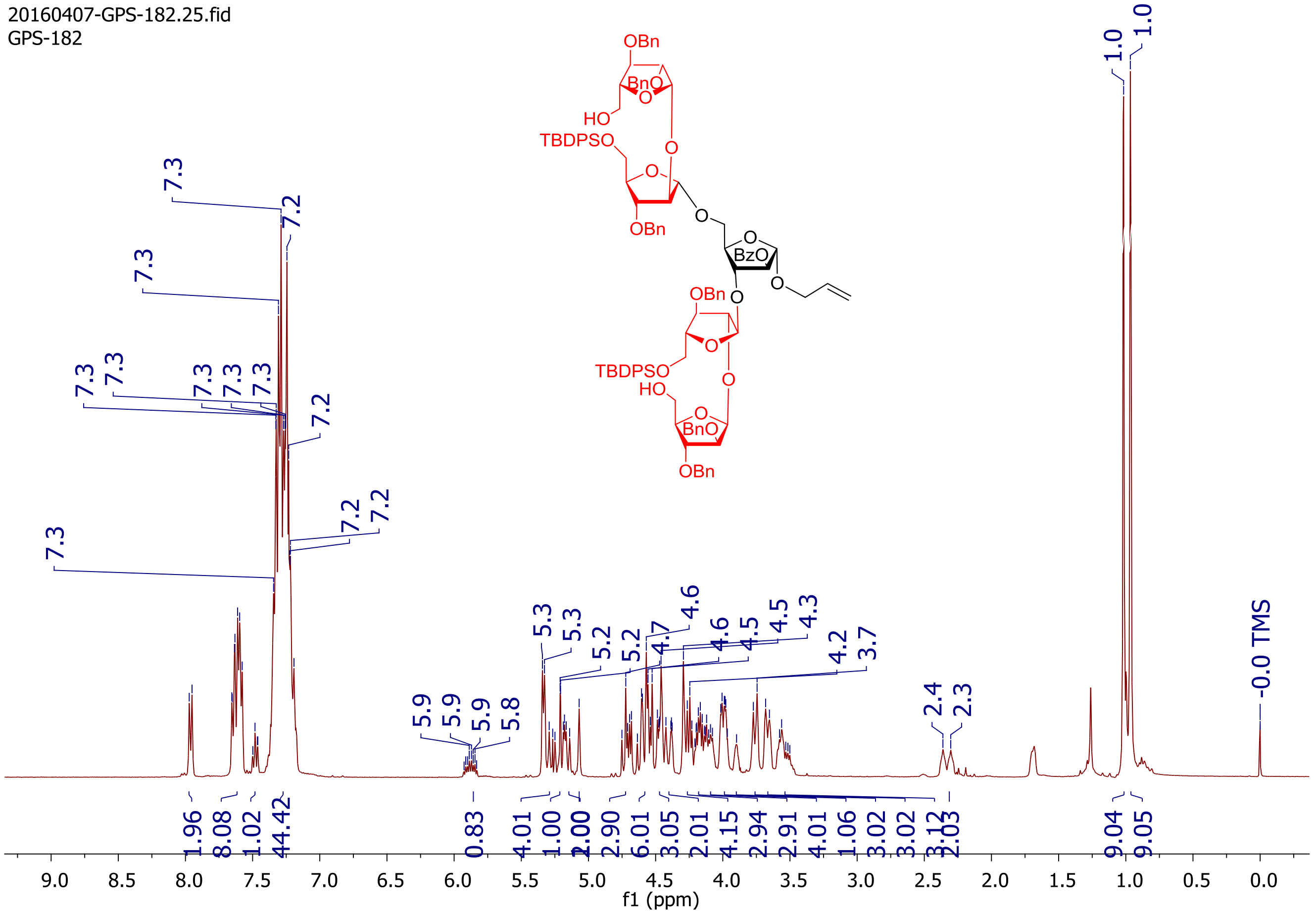

^{13}C NMR Spectrum (100.53 MHz, $CDCl_3$) of Compound **40**

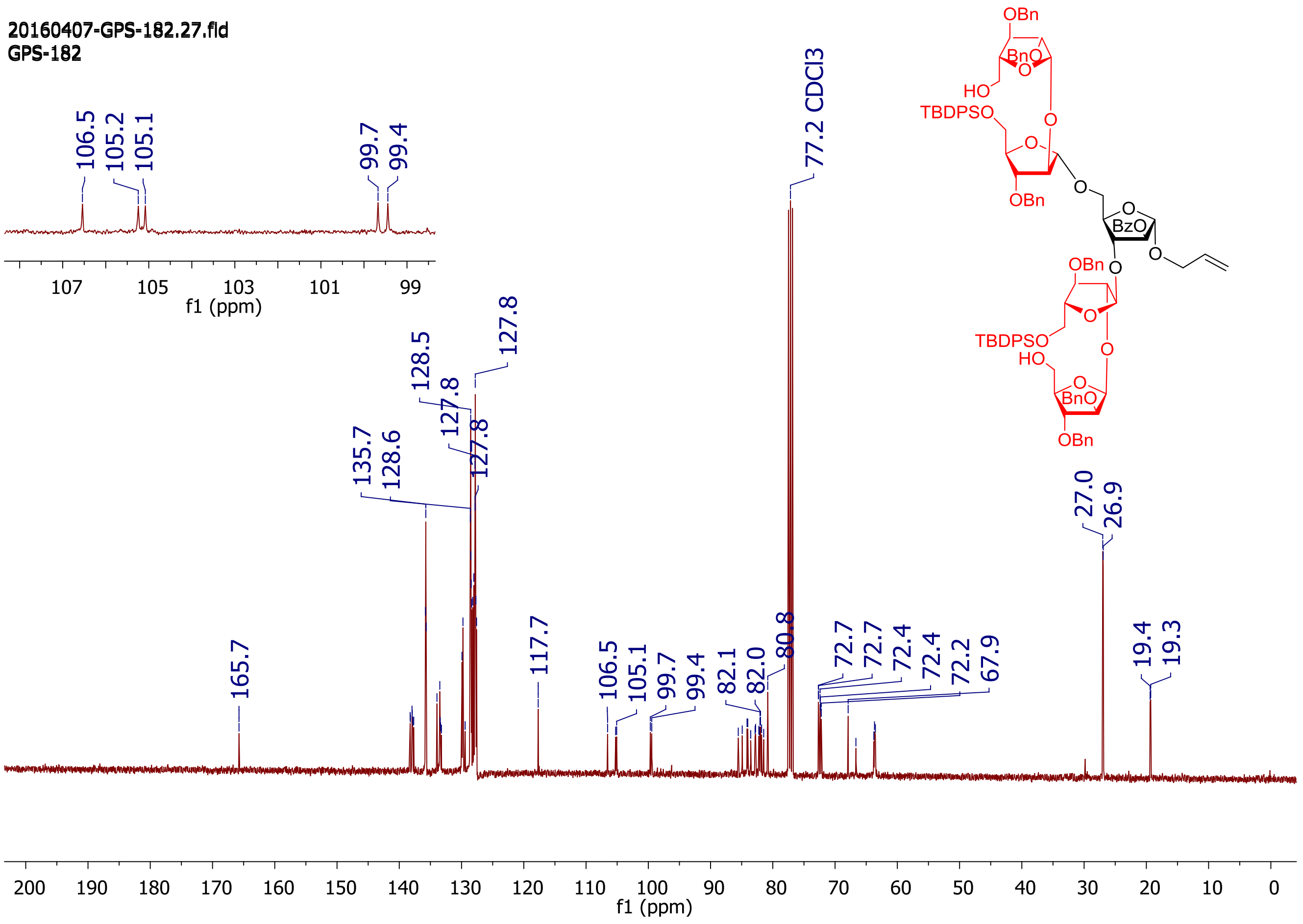

DEPT Spectrum (100.53 MHz, $CDCl_3$) of Compound **40**

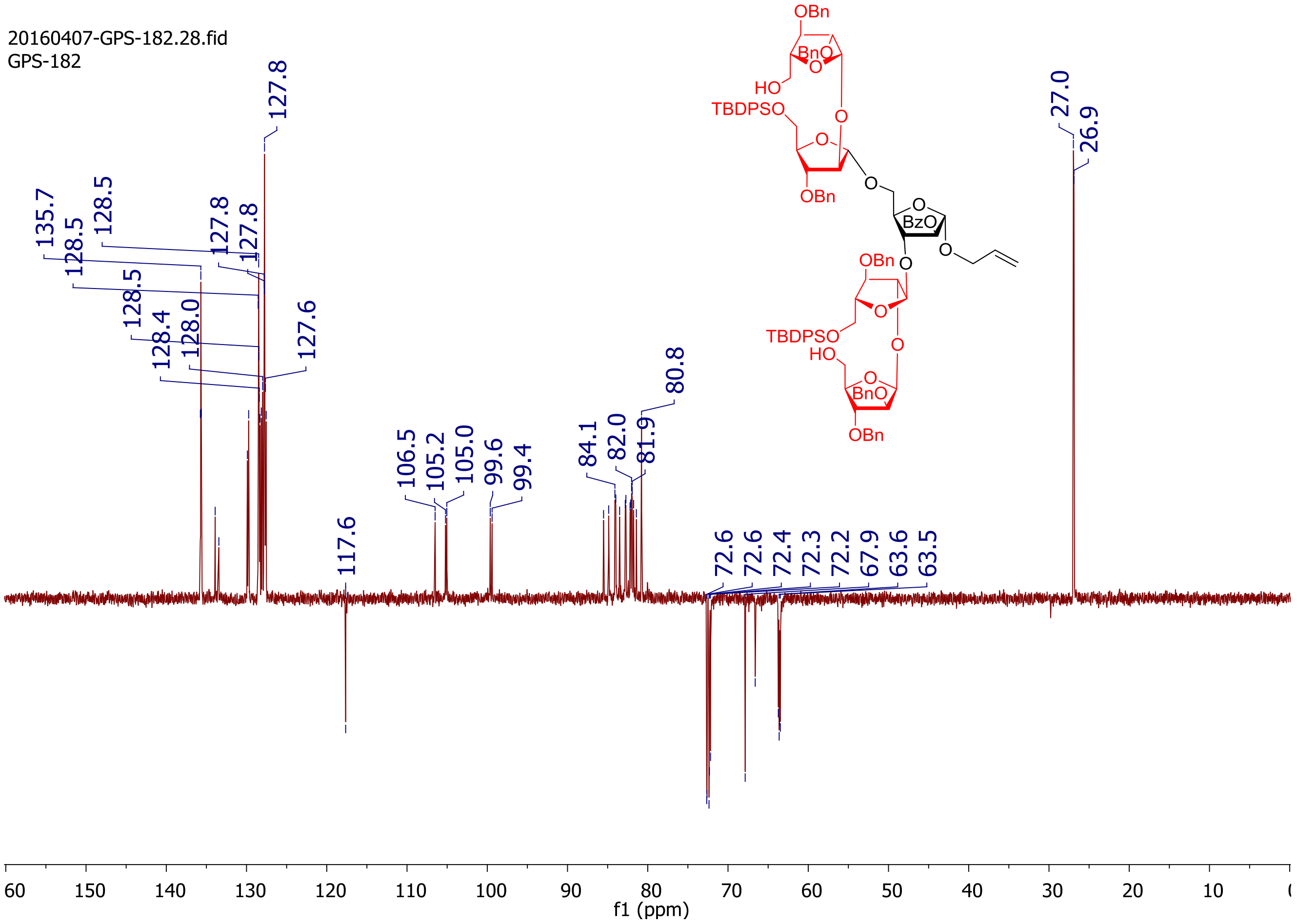

^{1}H-^{1}H COSY Spectrum (399.78 MHz, $CDCl_3$) of Compound **40**

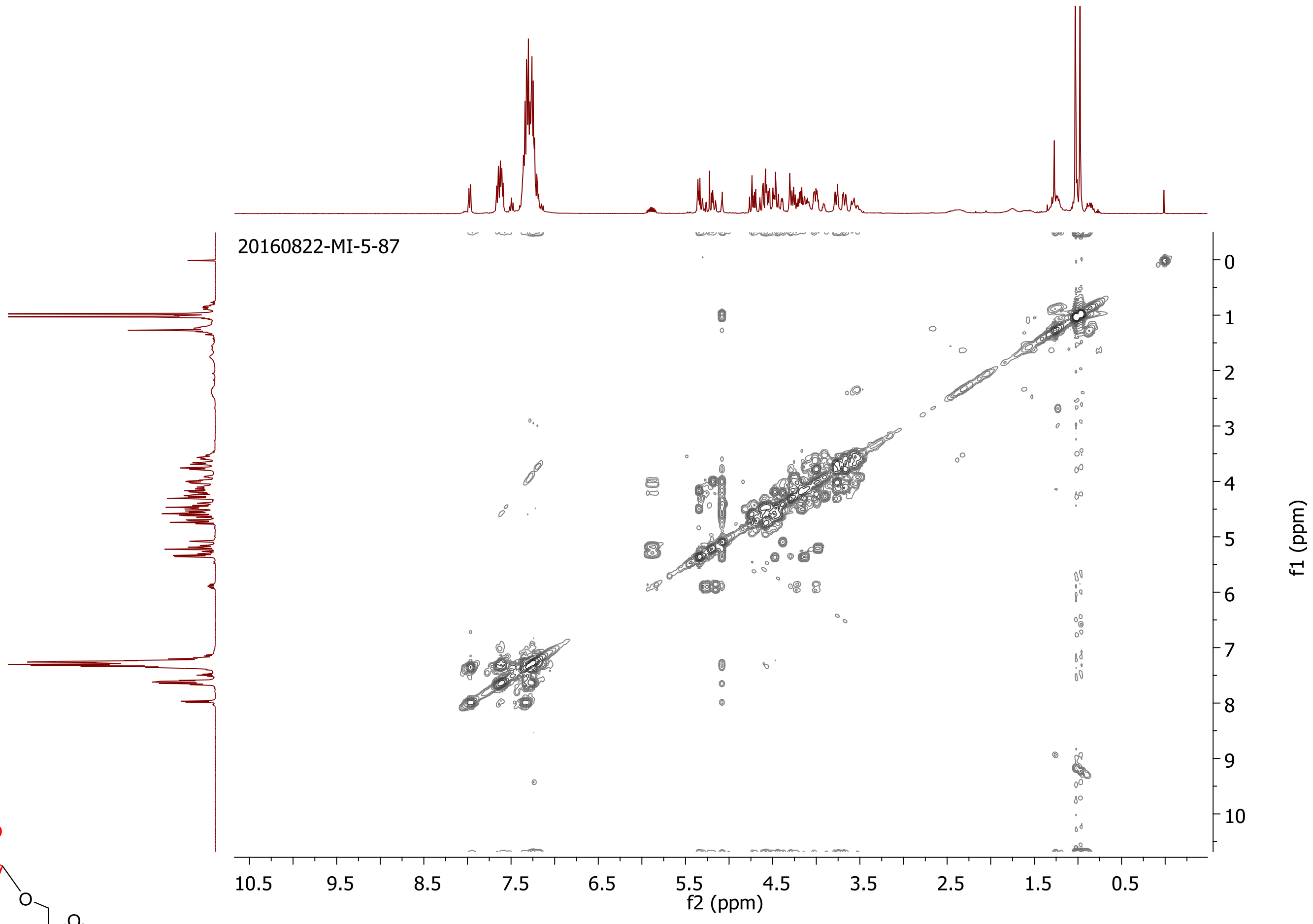

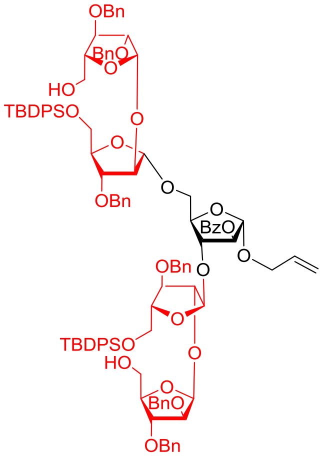

^{1}H-^{13}C HMBC Spectrum (399.78/100.53 MHz, $CDCl_3$) of Compound **40**

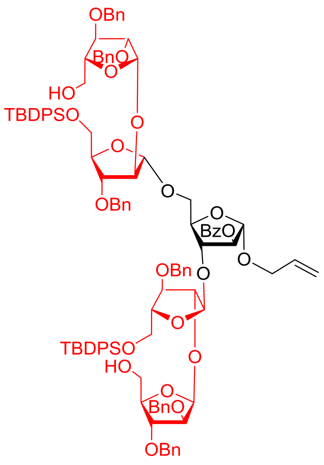

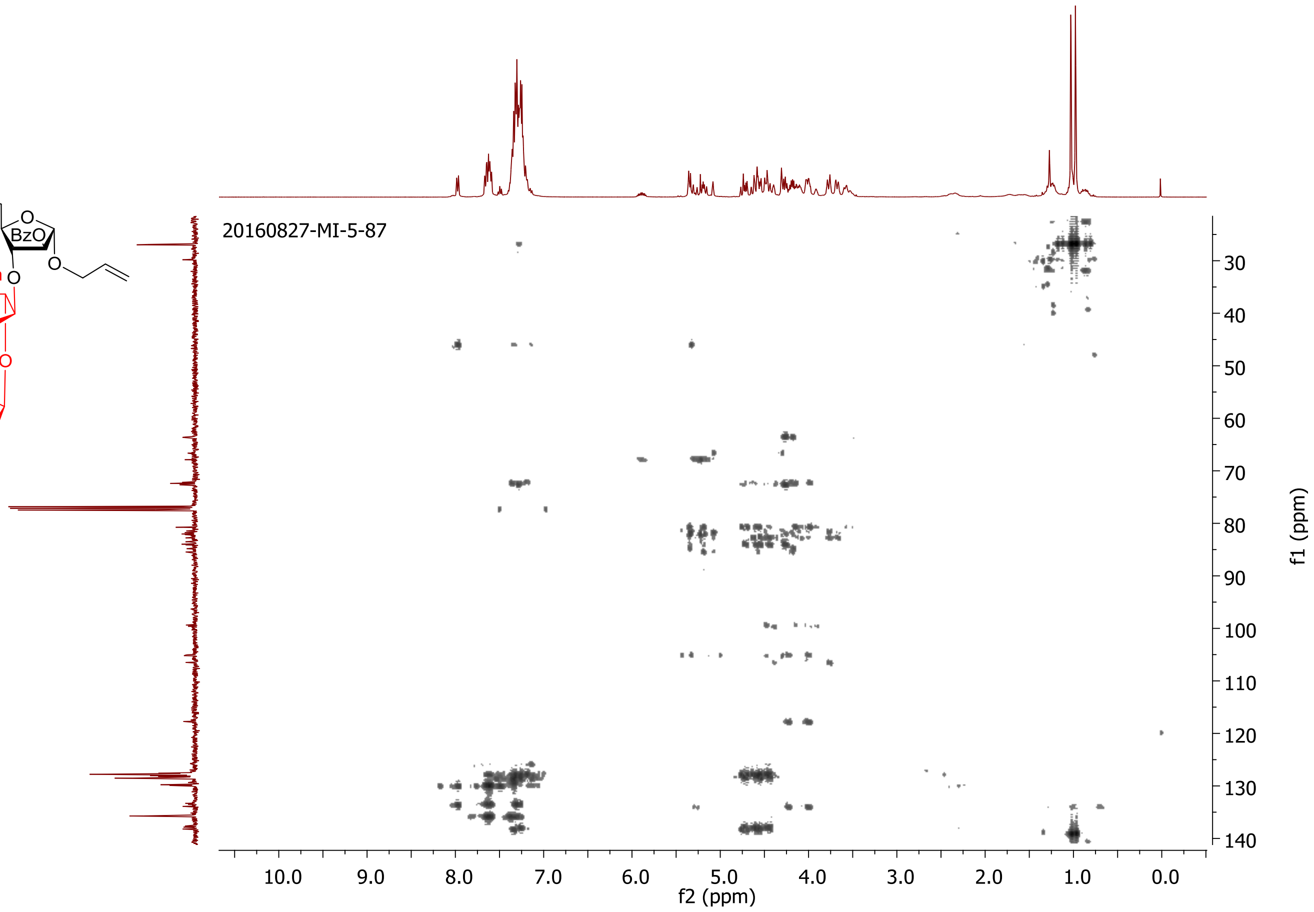

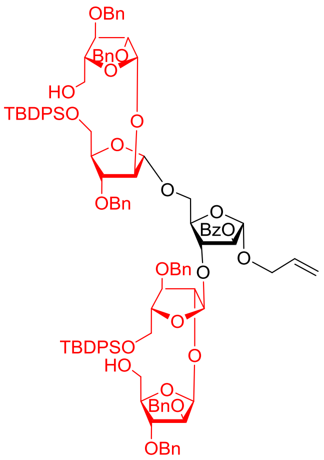

gHSQC Spectrum (399.78/100.53 MHz, $CDCl_3$) of Compound **40**

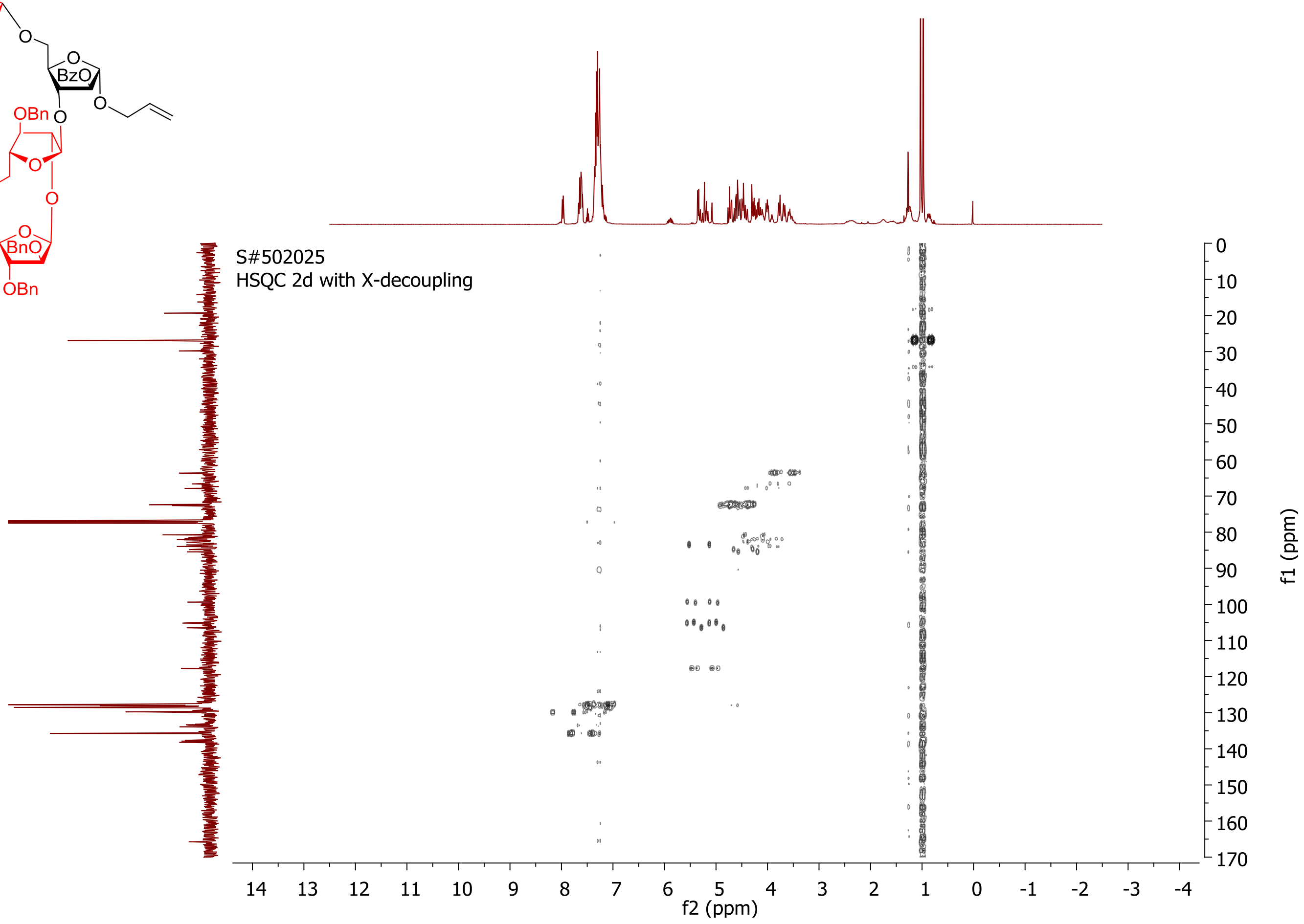

^{1}H NMR Spectrum (399.78 MHz, $CDCl_3$) of Compound **41**

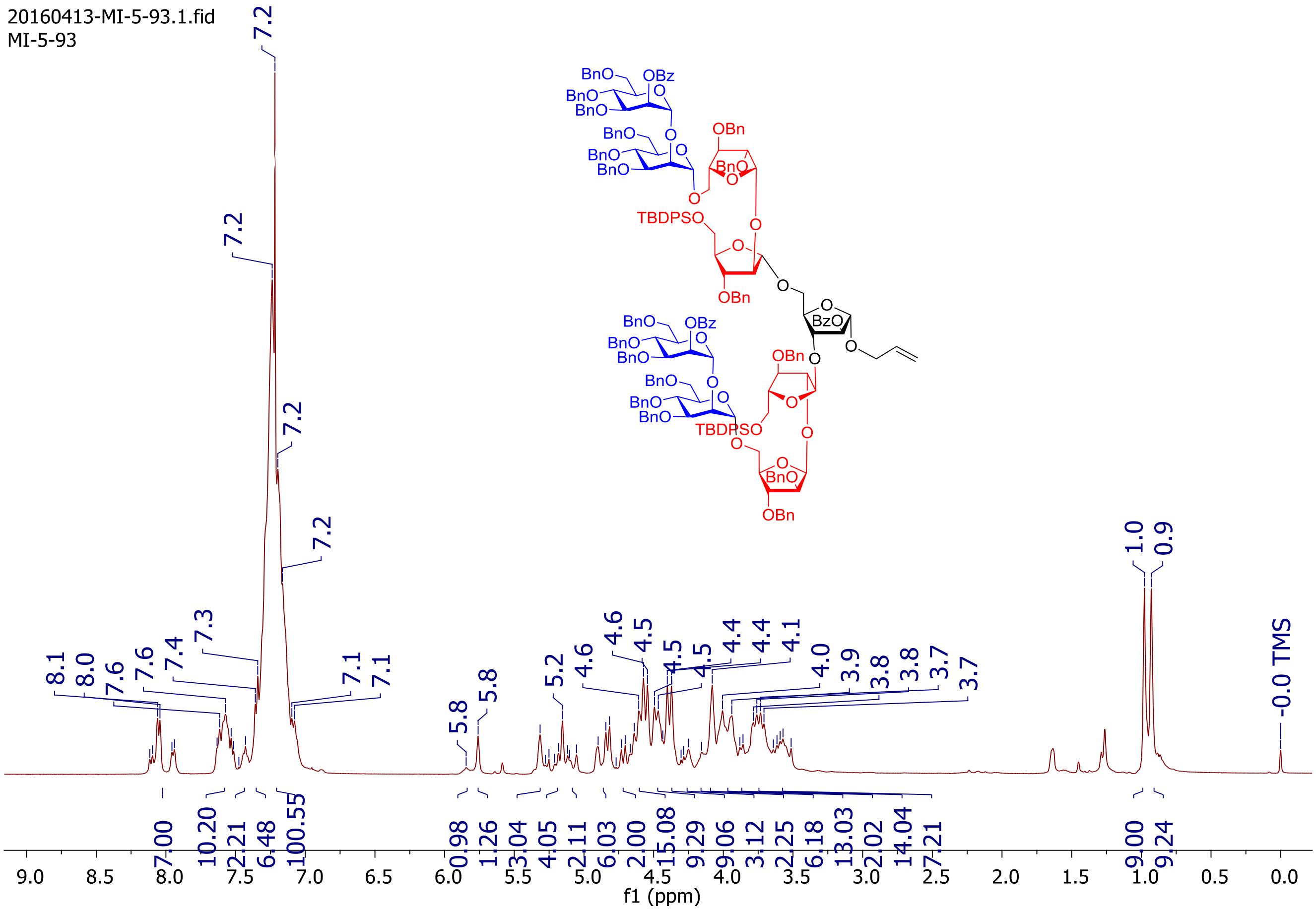

^{13}C NMR Spectrum (100.53 MHz, $CDCl_3$) of Compound **41**

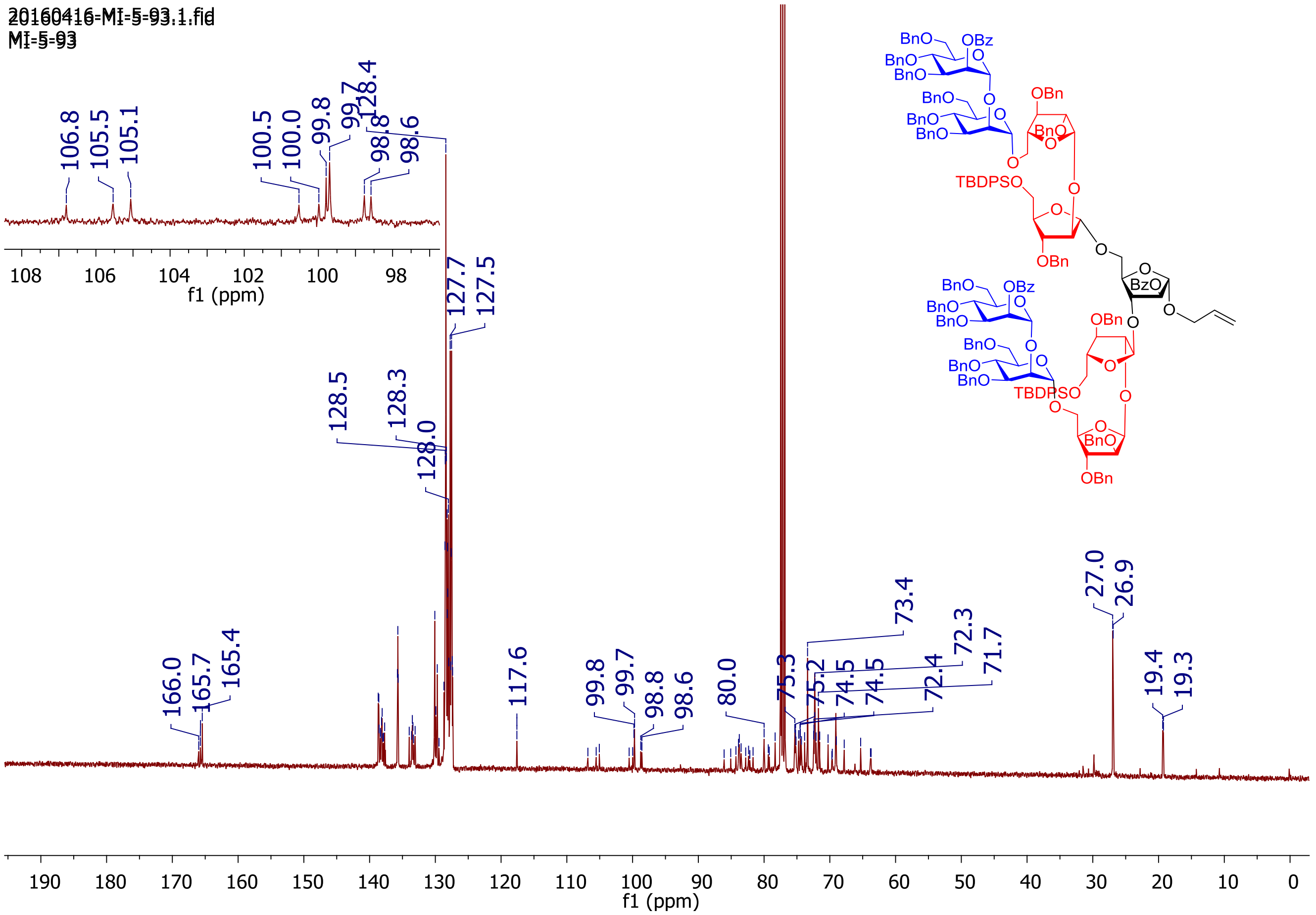

DEPT Spectrum (100.53 MHz, $CDCl_3$) of Compound **41**

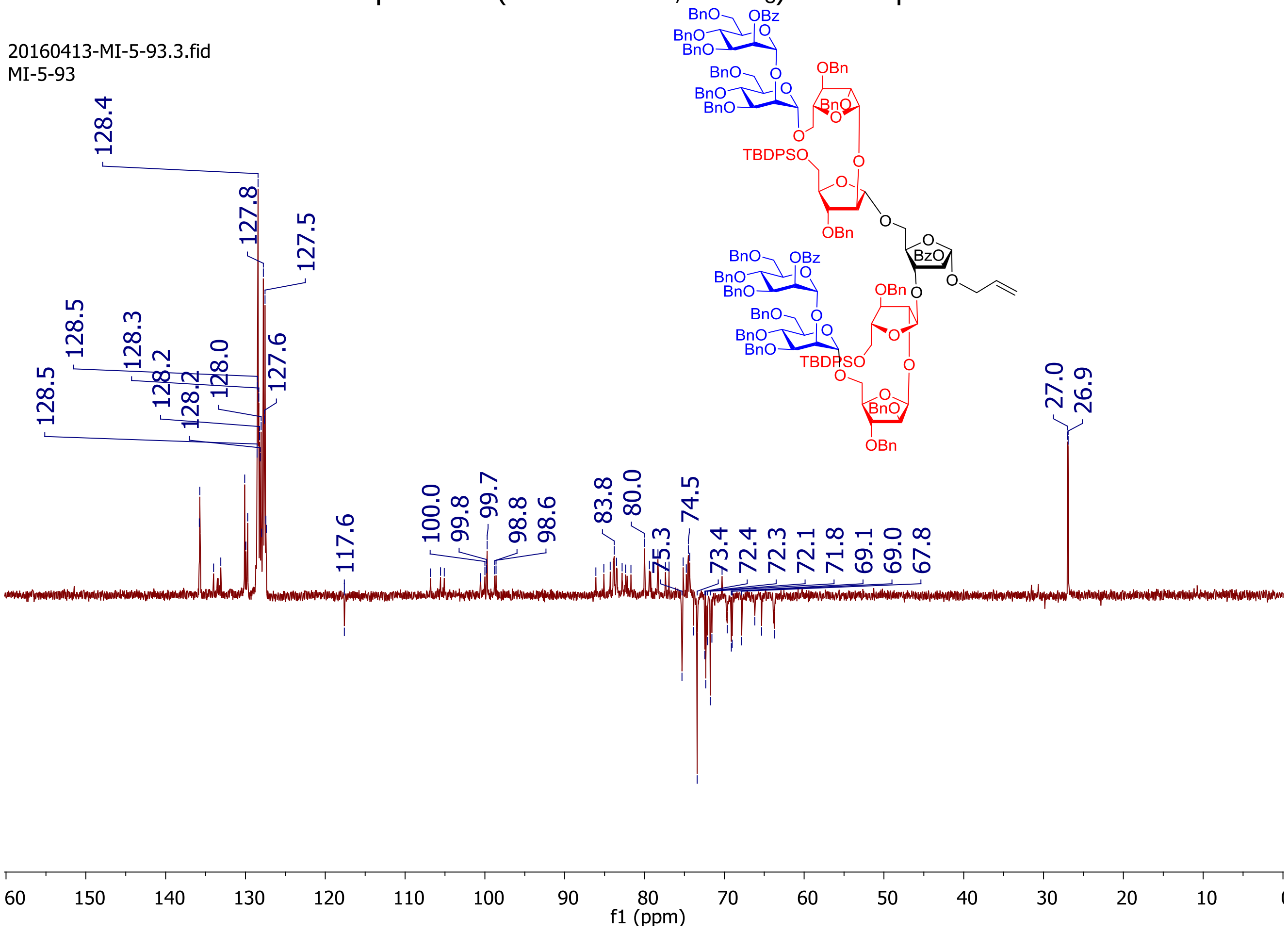

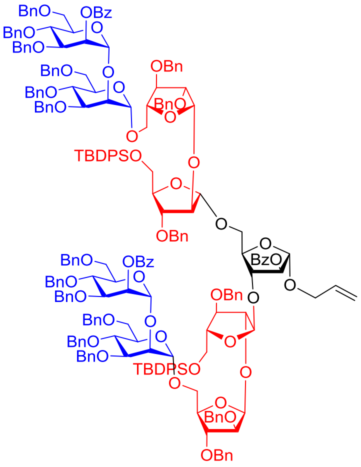

^{1}H-^{1}H COSY Spectrum (399.78 MHz, $CDCl_3$) of Compound **41**

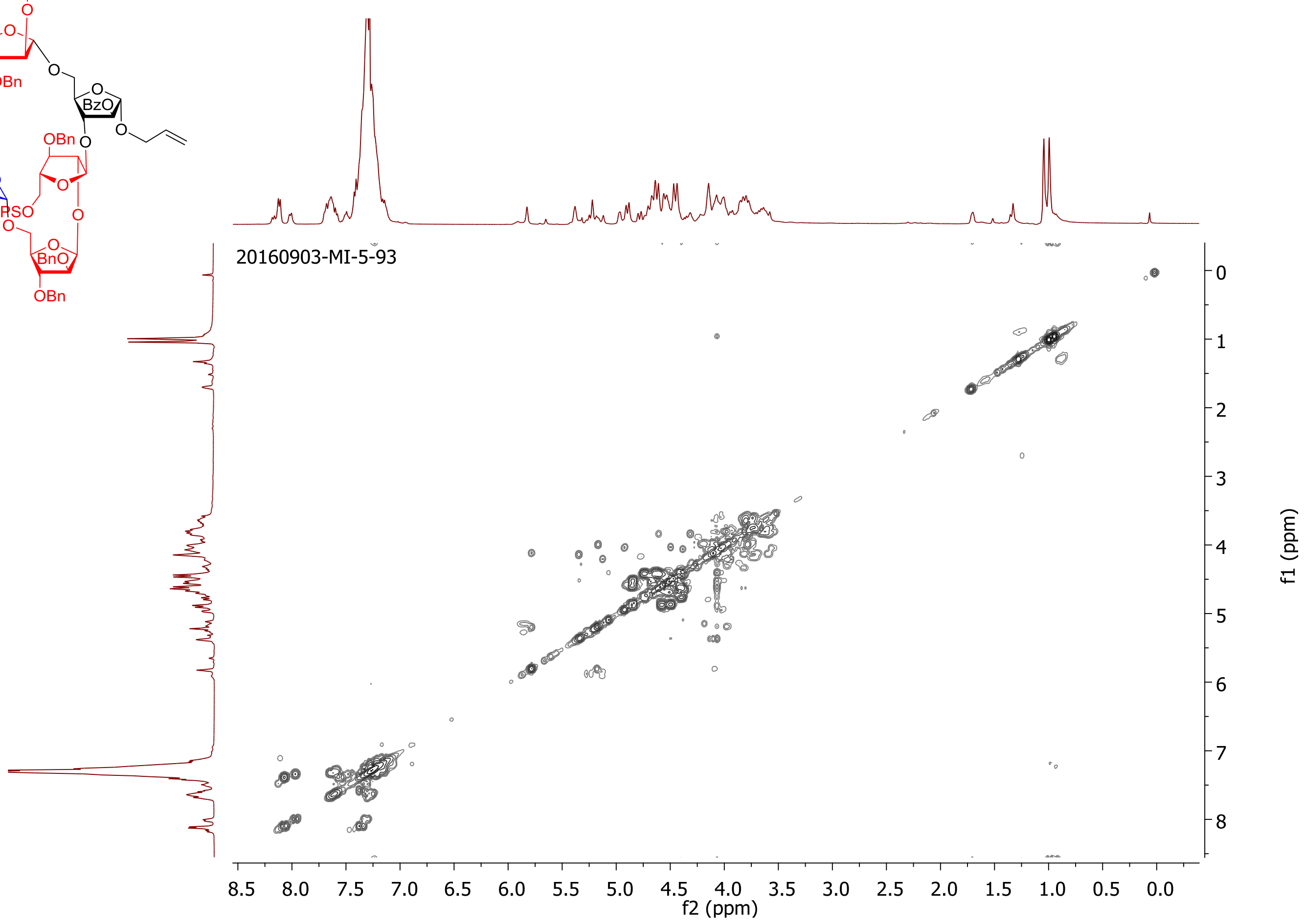

^{1}H-^{13}C HMBC Spectrum (399.78/100.53 MHz, $CDCl_3$) of Compound **41**

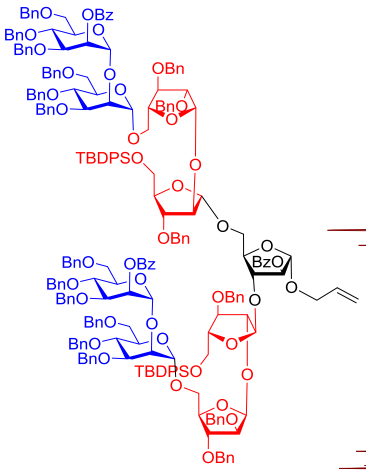

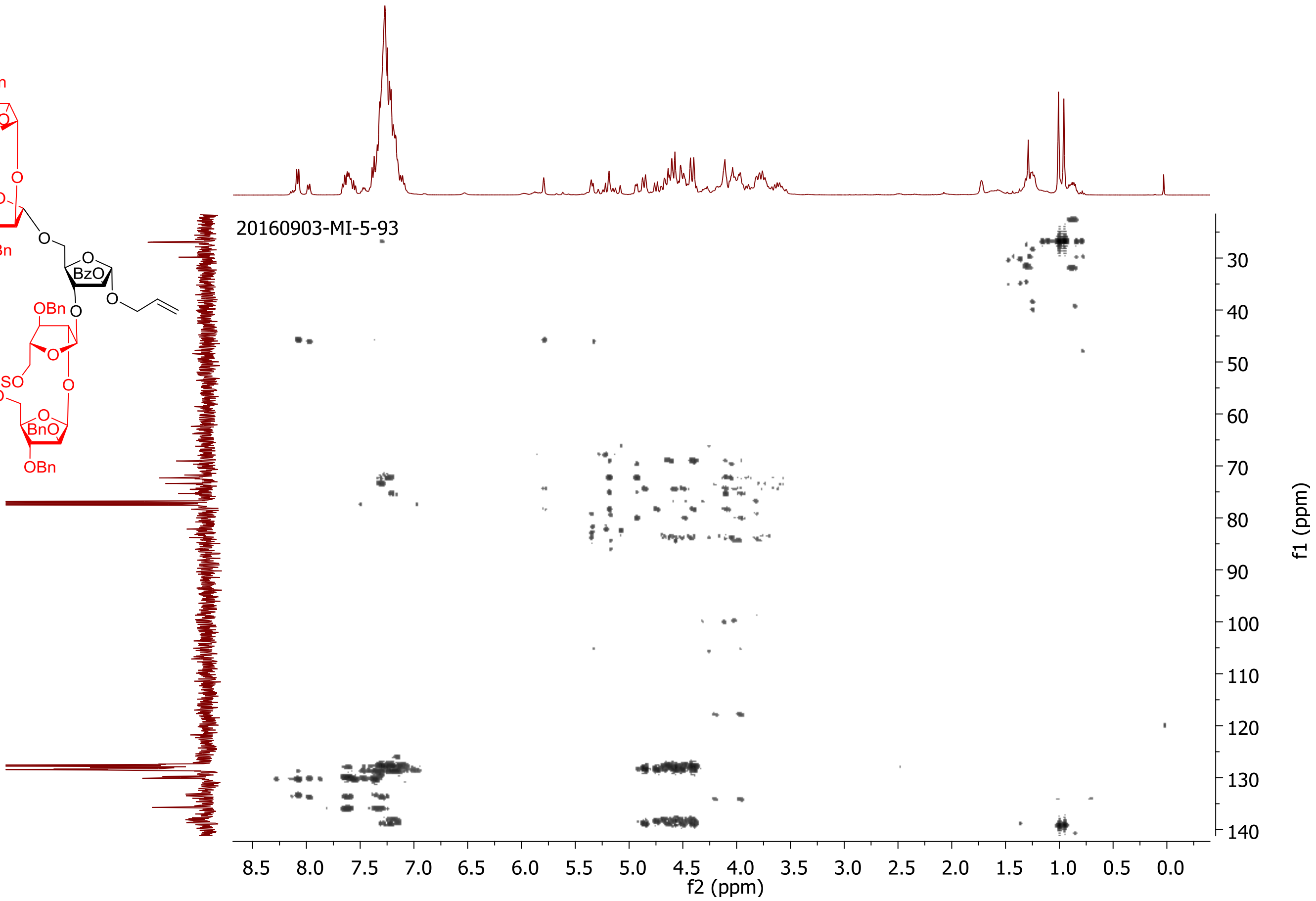

gHSQC Spectrum (399.78/100.53 MHz, $CDCl_3$) of Compound **41**

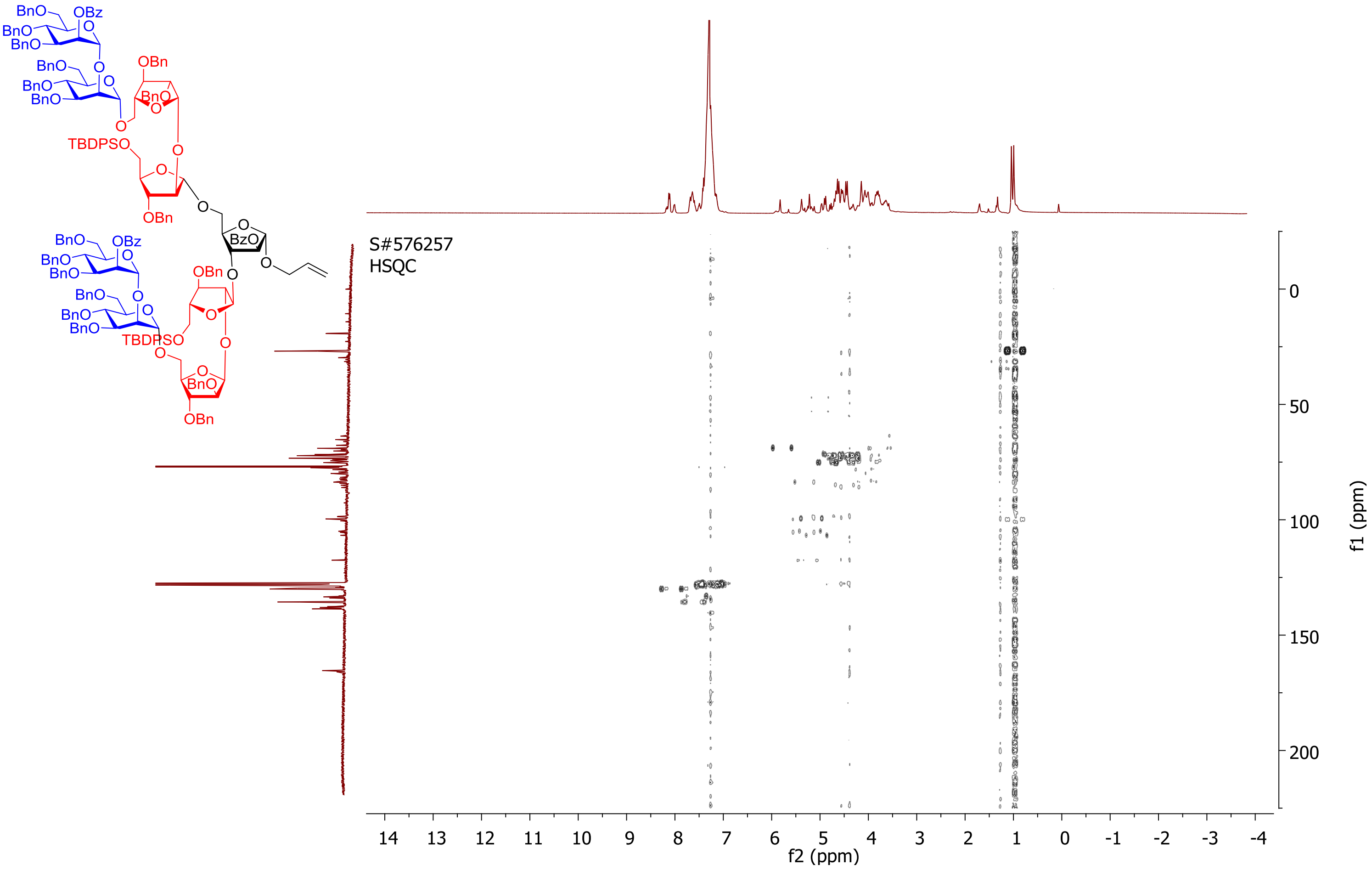

^{1}H NMR Spectrum (399.78 MHz, $CDCl_3$) of Compound **42**

20160903-MI-5-95
MI-5-95

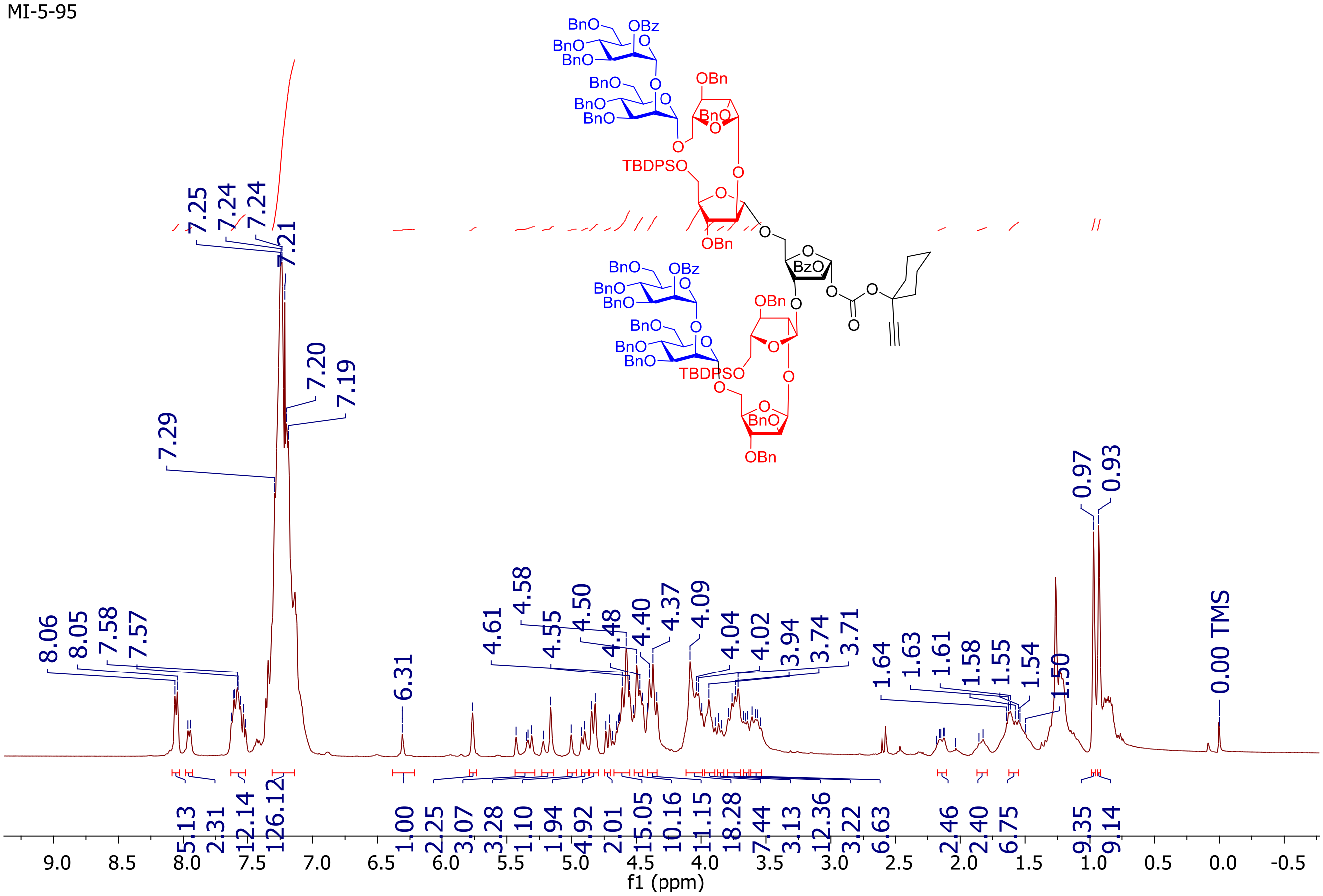

^{13}C NMR Spectrum (100.53 MHz, $CDCl_3$) of Compound **42**

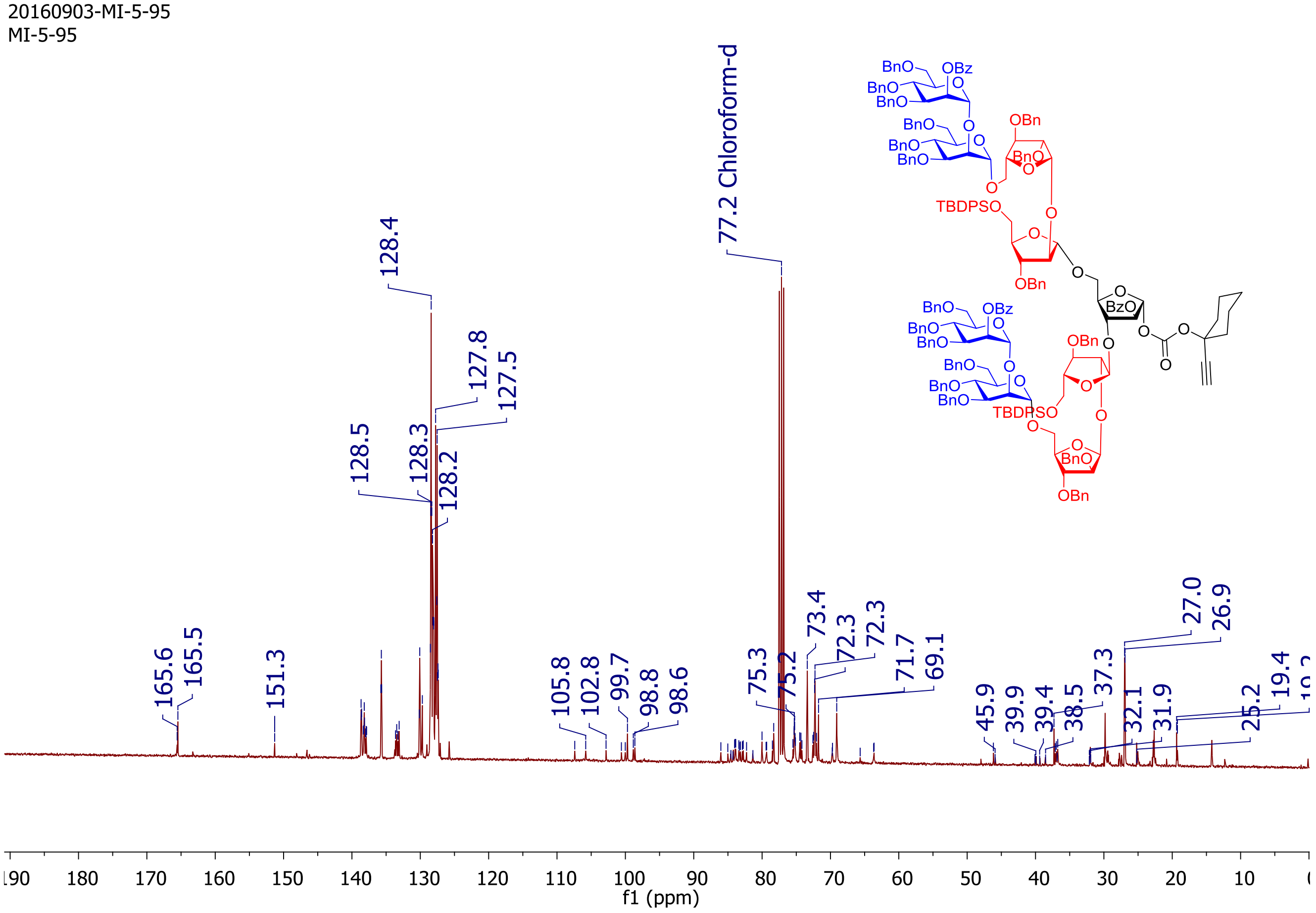

DEPT Spectrum (100.53 MHz, $CDCl_3$) of Compound **42**

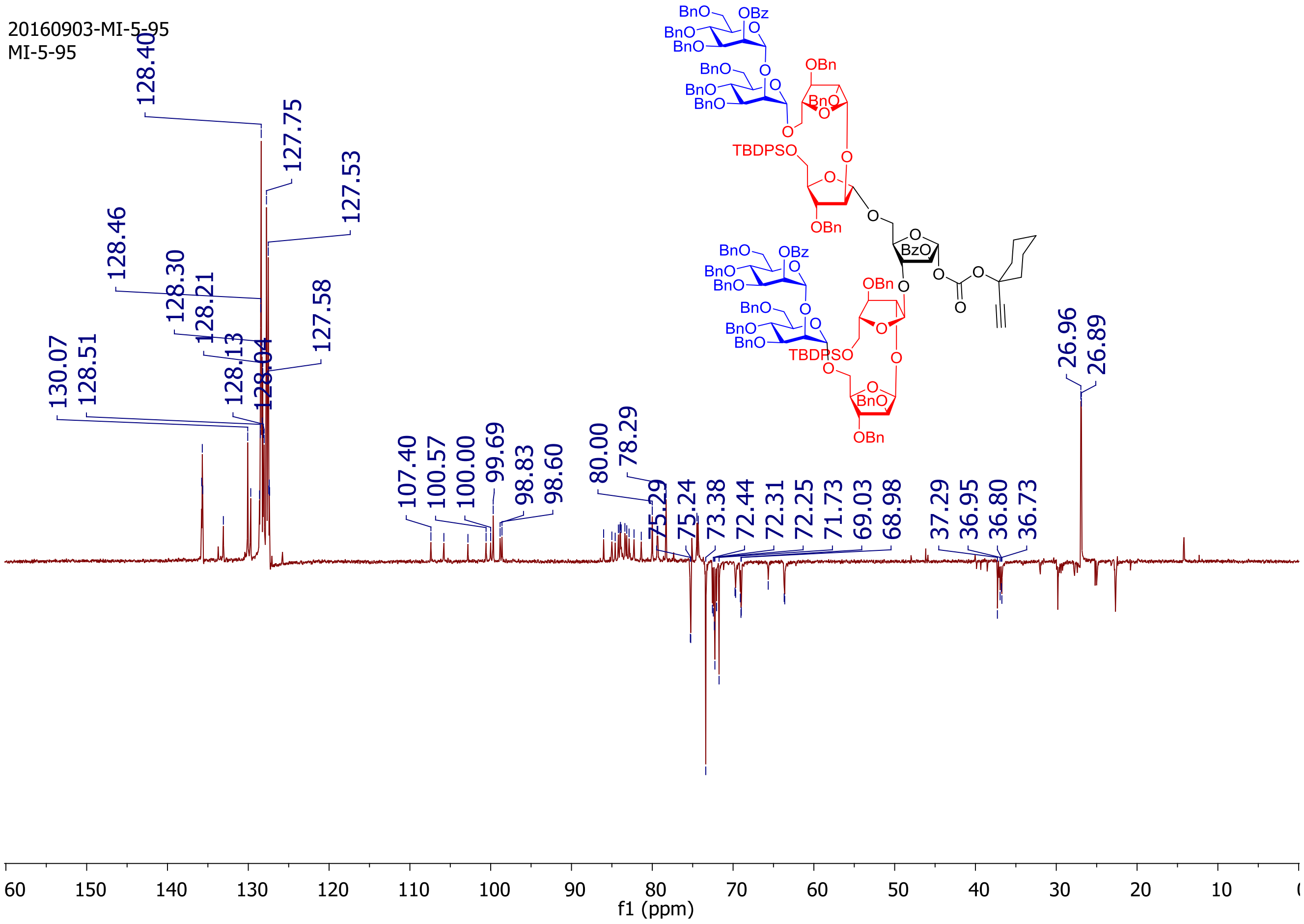

^{1}H NMR Spectrum (399.78 MHz, $CDCl_3$) of Compound **43**

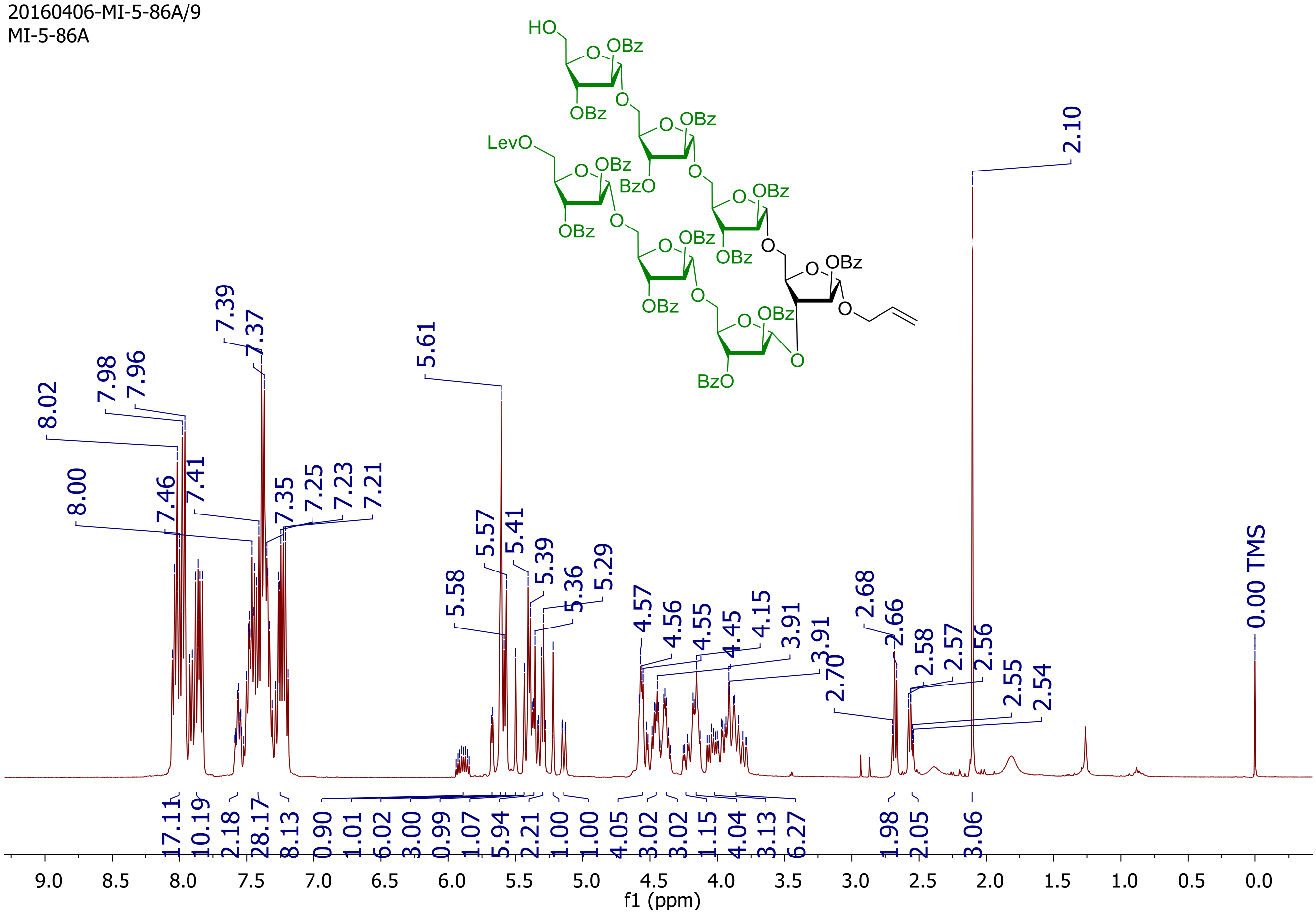

^{13}C NMR Spectrum (100.53 MHz, $CDCl_3$) of Compound **43**

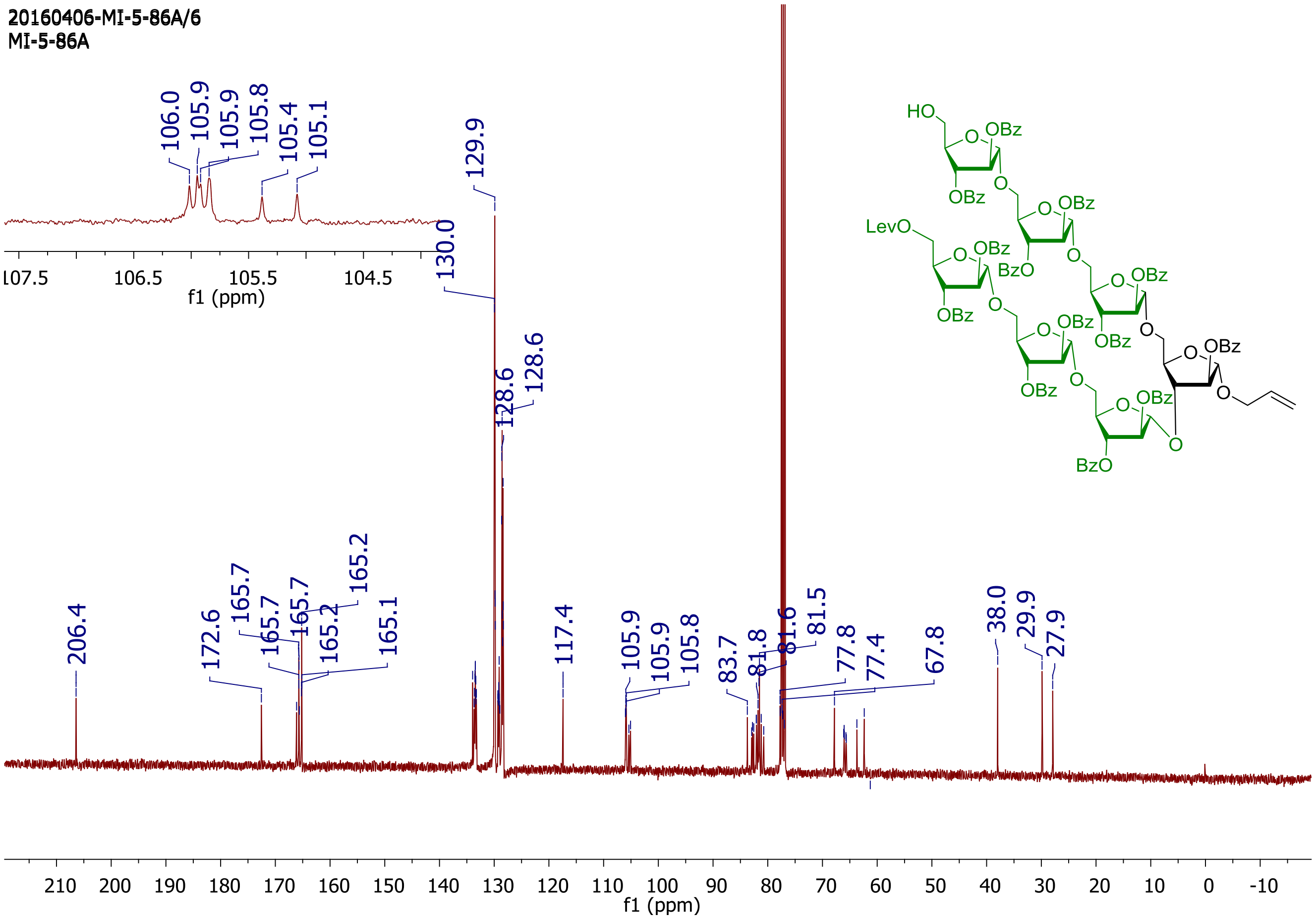

DEPT Spectrum (100.53 MHz, $CDCl_3$) of Compound **43**

20160406-MI-5-86A/8
MI-5-86A

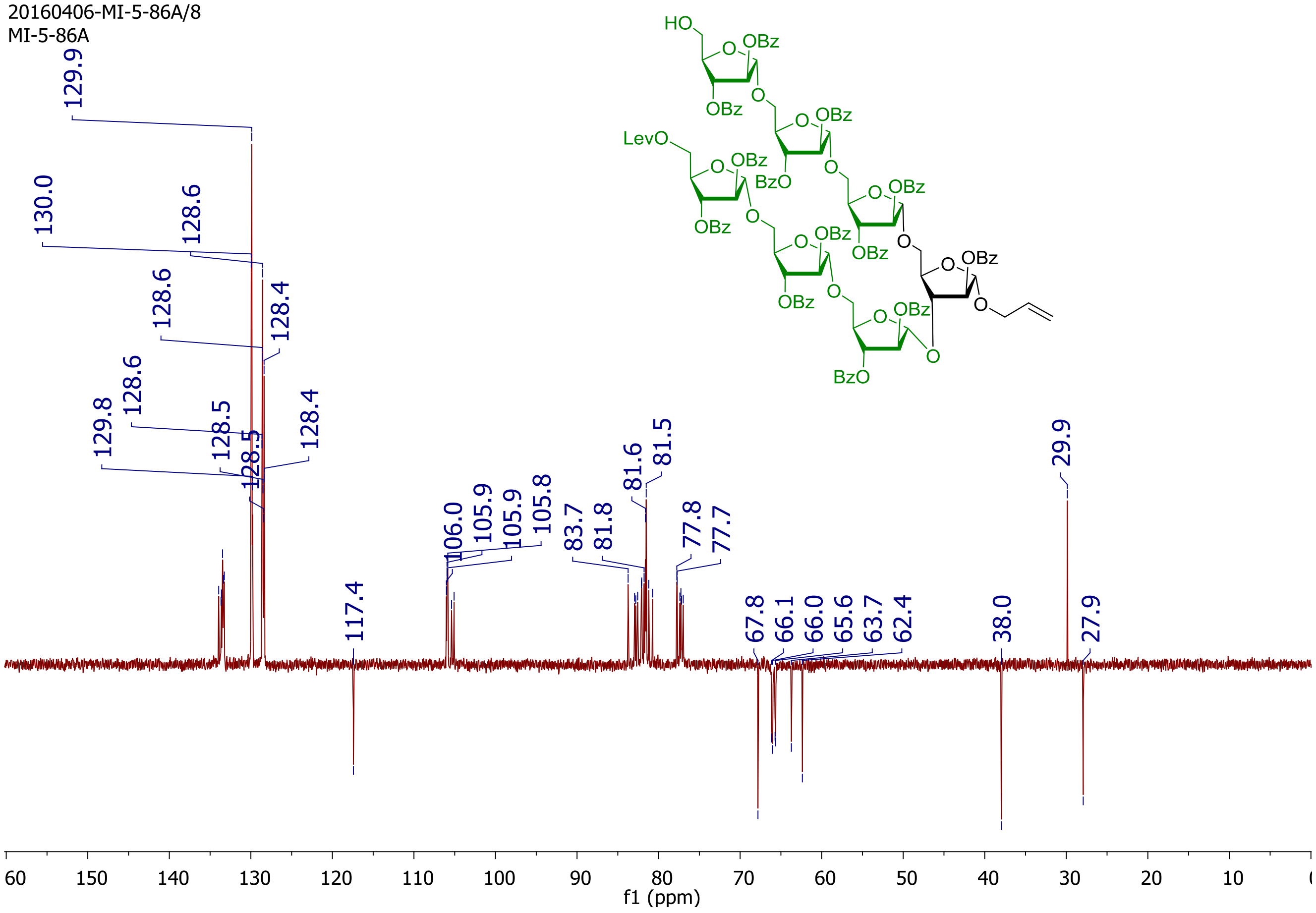

^{1}H NMR Spectrum (399.78 MHz, $CDCl_3$) of Compound **44**

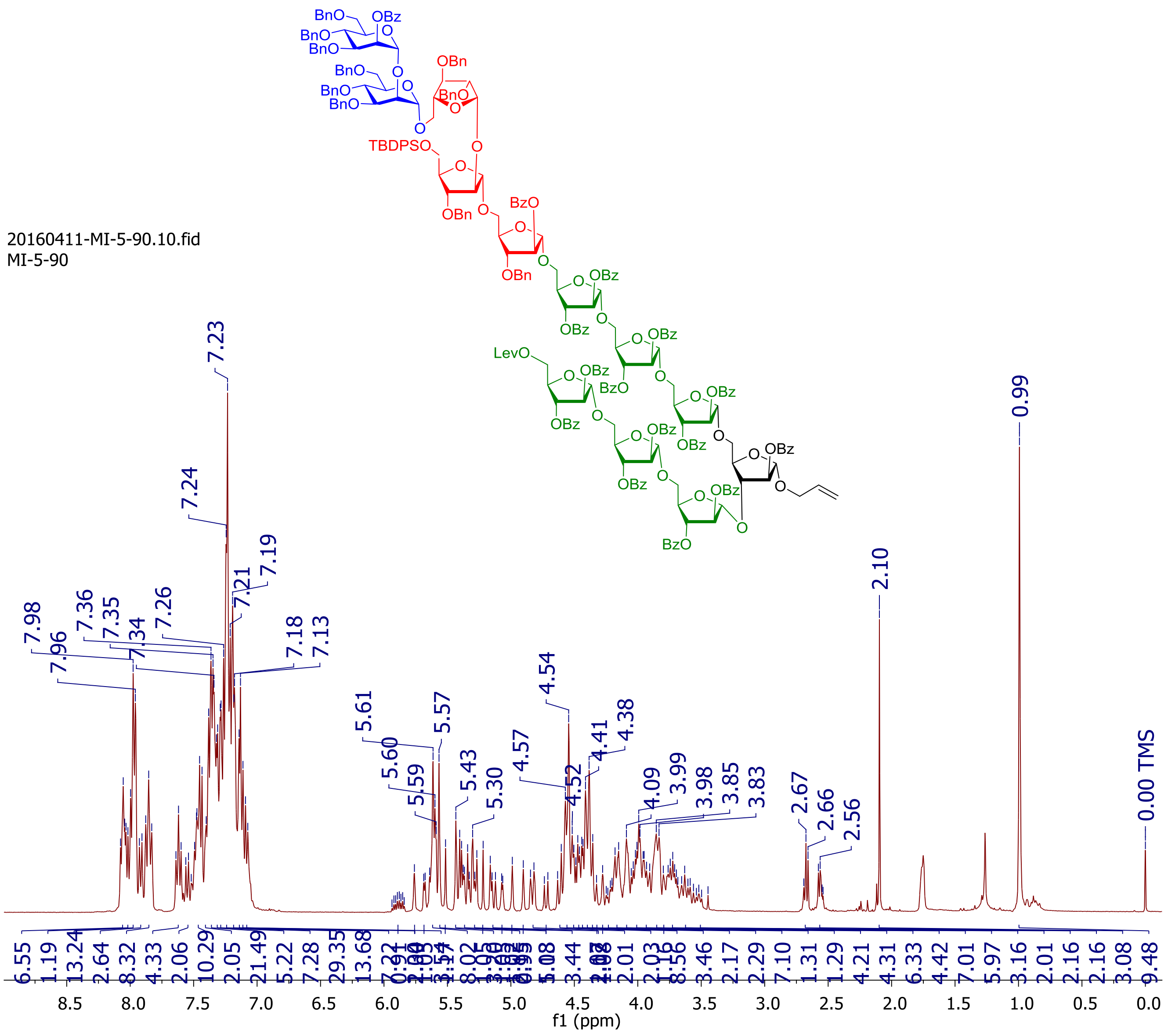

^{13}C NMR Spectrum (100.53 MHz, $CDCl_3$) of Compound **44**

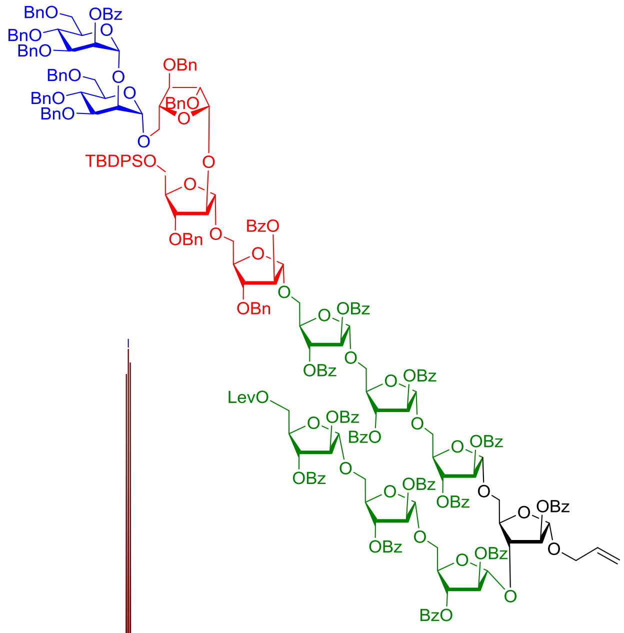

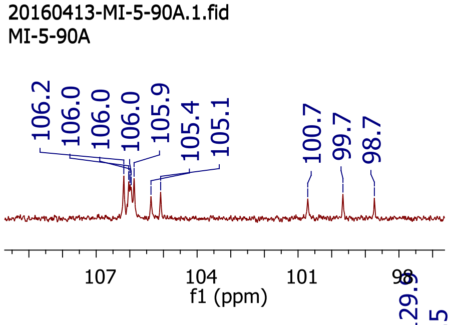

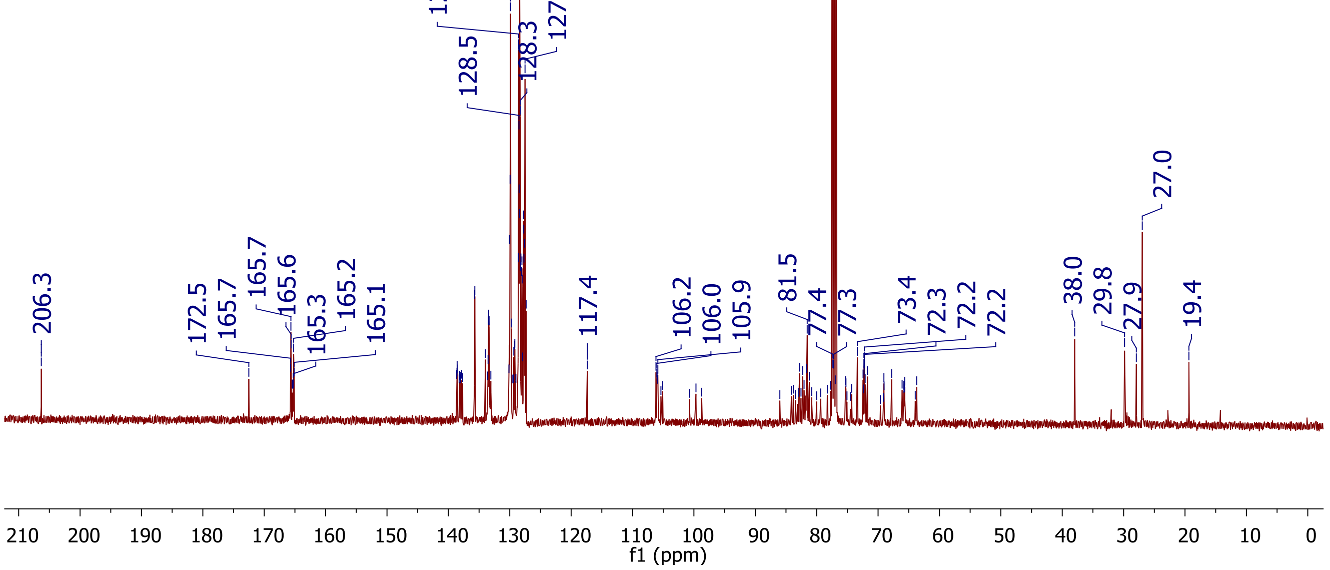

DEPT Spectrum (100.53 MHz, $CDCl_3$) of Compound **44**

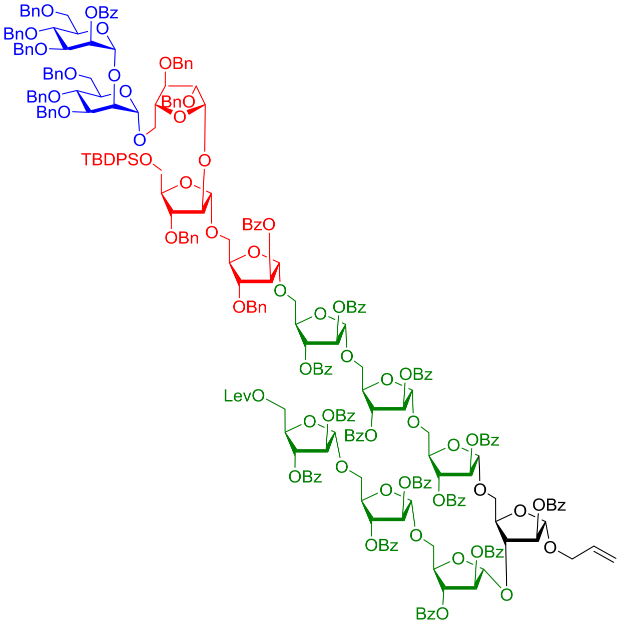

20160411-MI-5-90.13.fid
MI-5-90

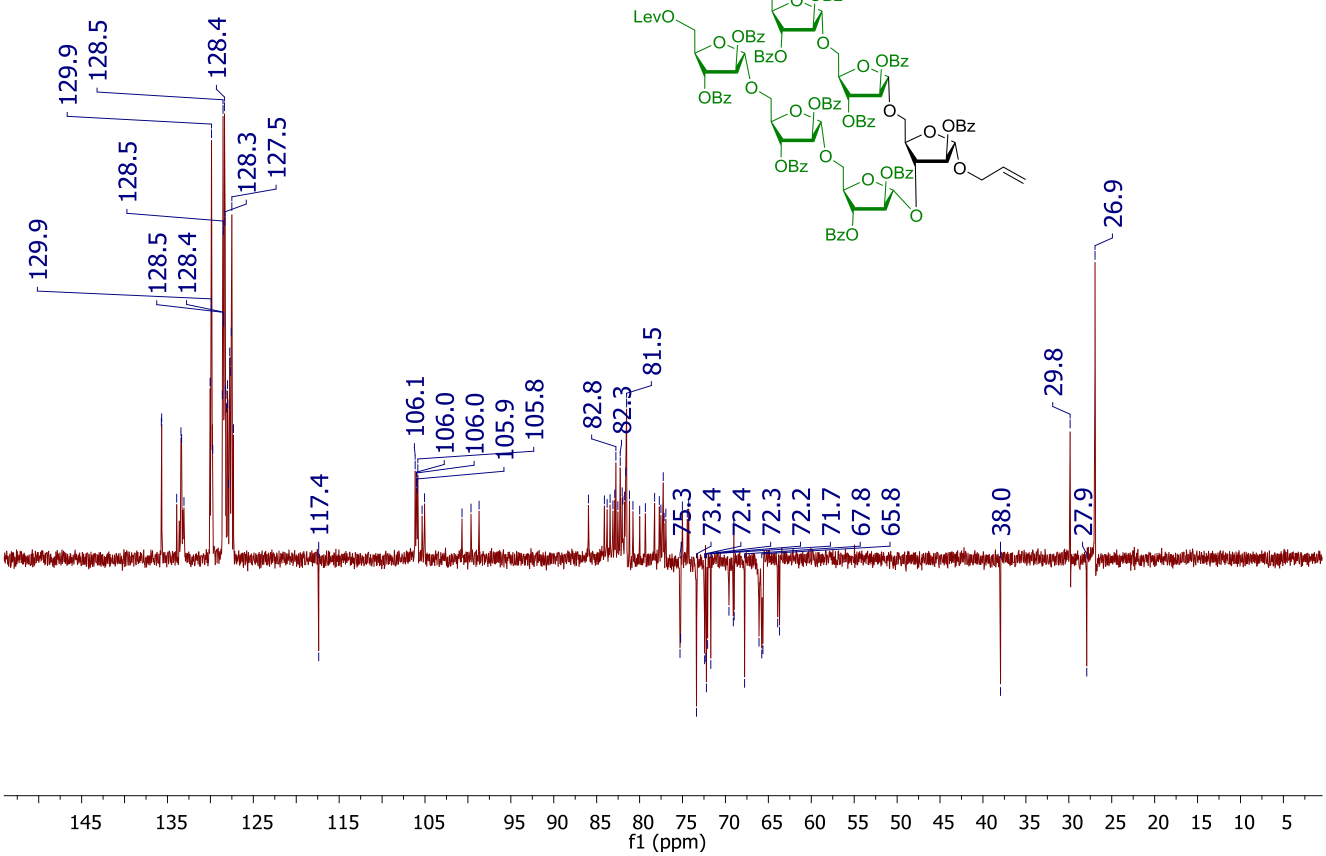

^{1}H NMR Spectrum (399.78 MHz, $CDCl_3$) of Compound **45**

20160418-MI-5-94.1.fid
MI-5-94

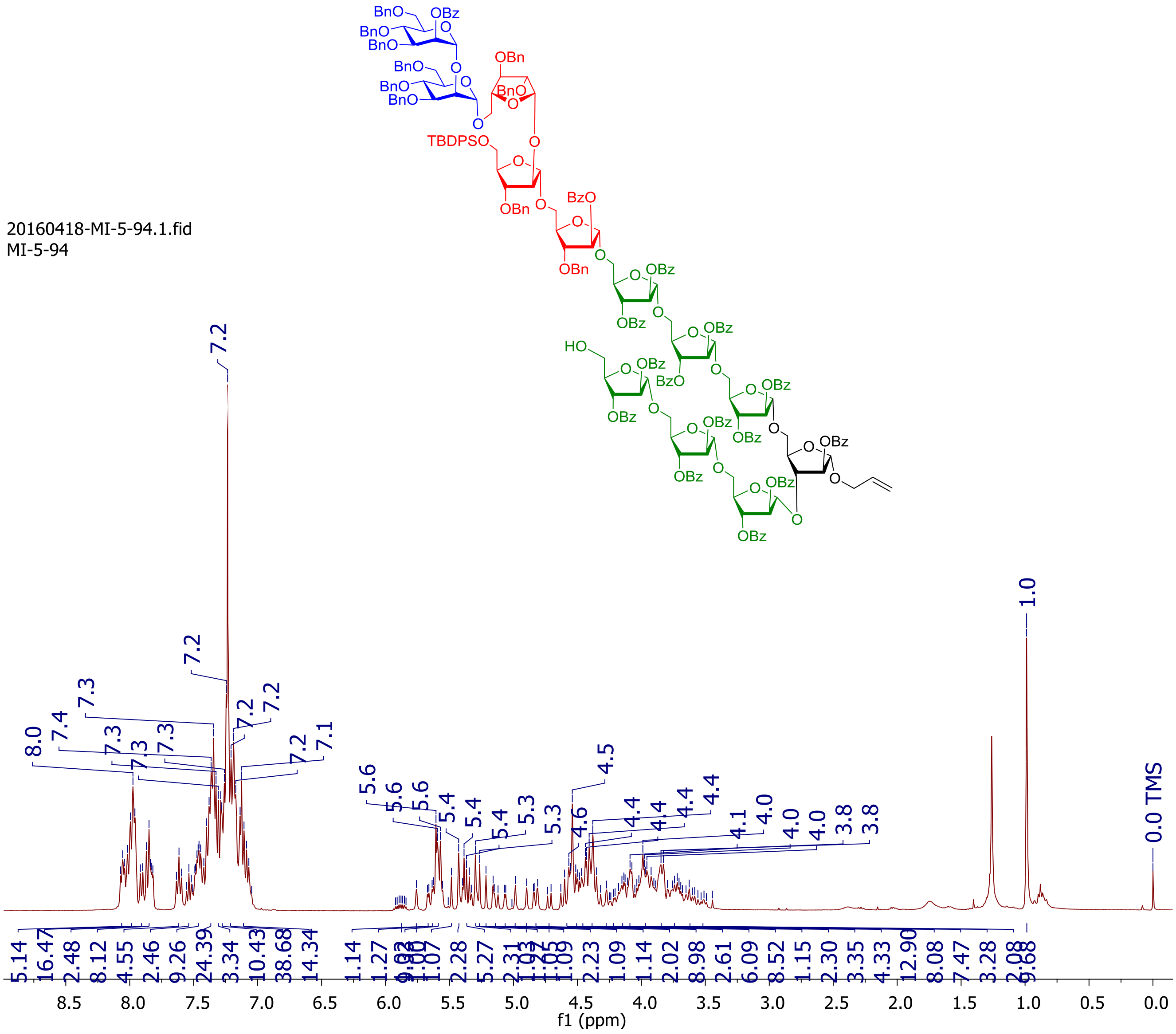

^{13}C NMR Spectrum (100.53 MHz, $CDCl_3$) of Compound **45**

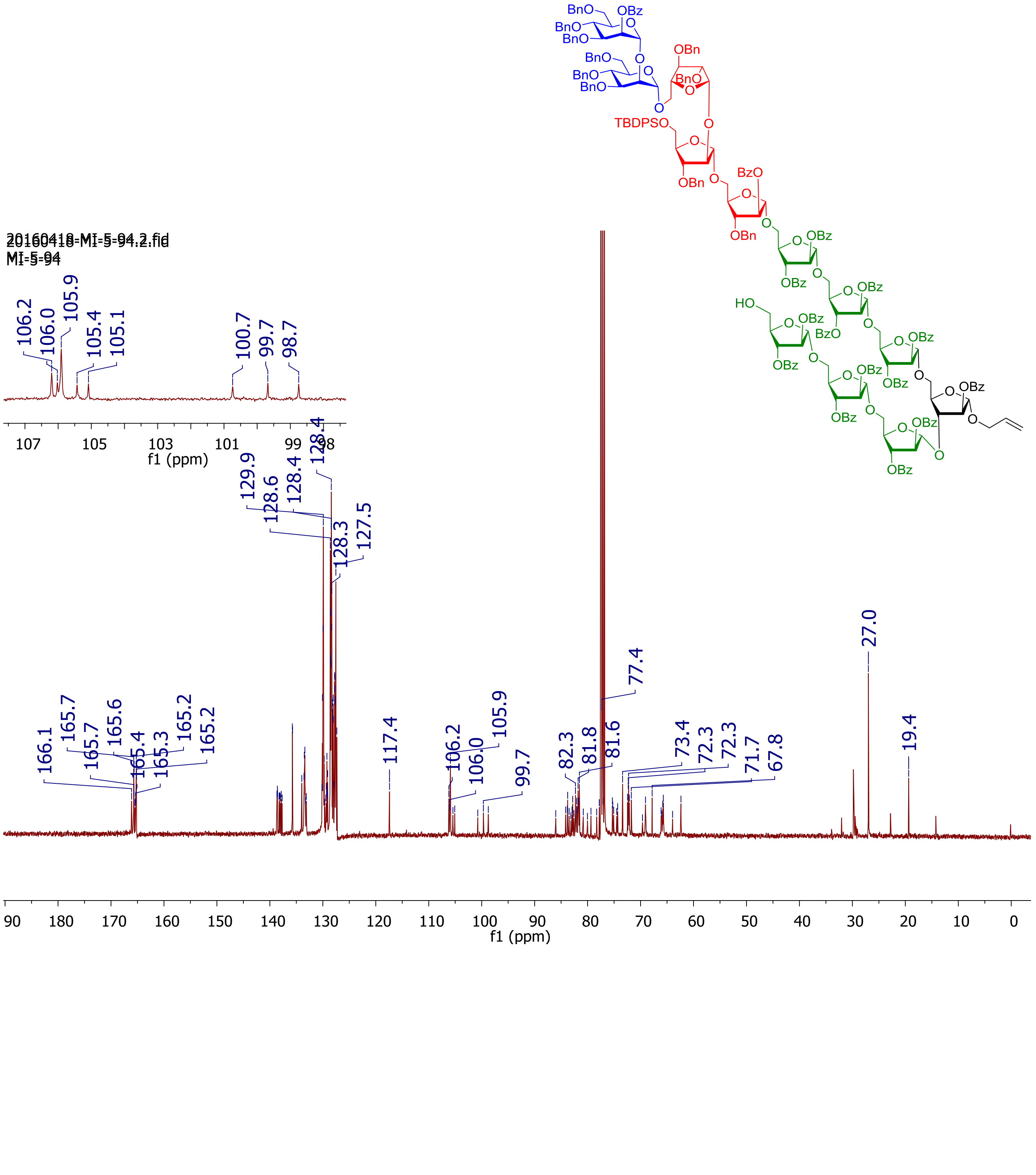

DEPT Spectrum (100.53 MHz, $CDCl_3$) of Compound **45**

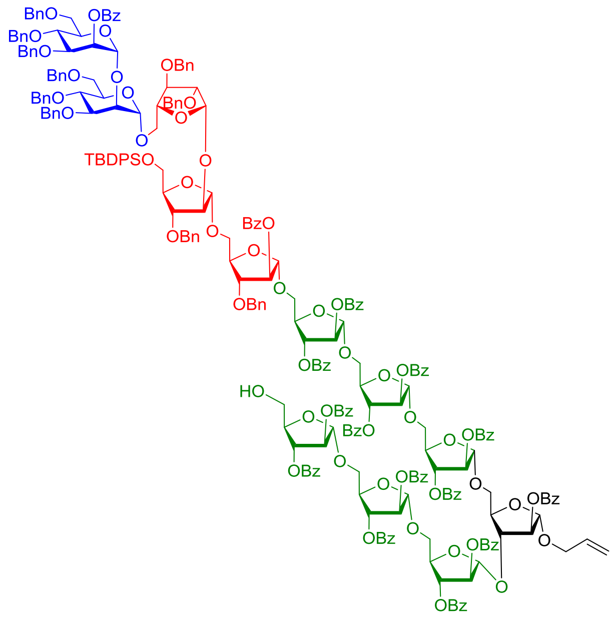

20160418-MI-5-94.3.fid
MI-5-94

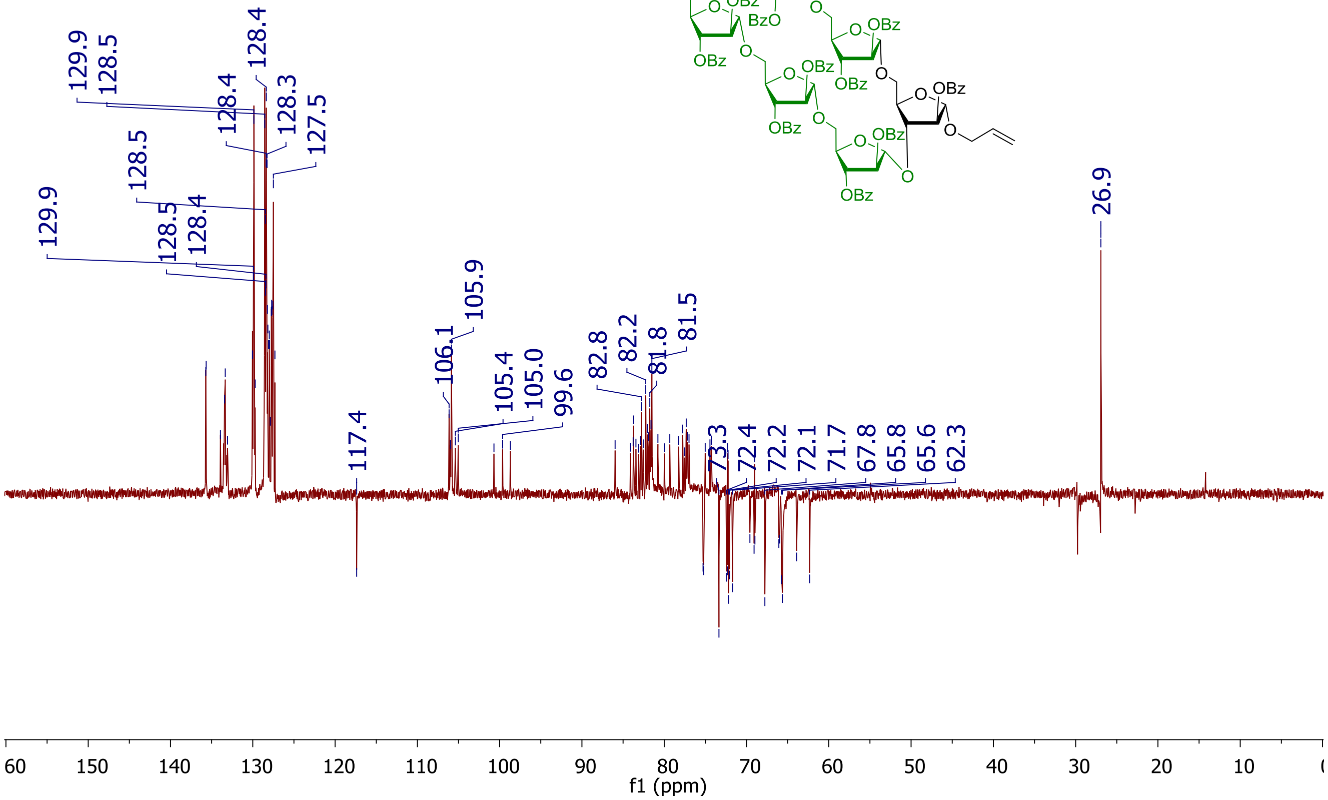

^{1}H-^{1}H COSY Spectrum (399.78 MHz, $CDCl_3$) of Compound **45**

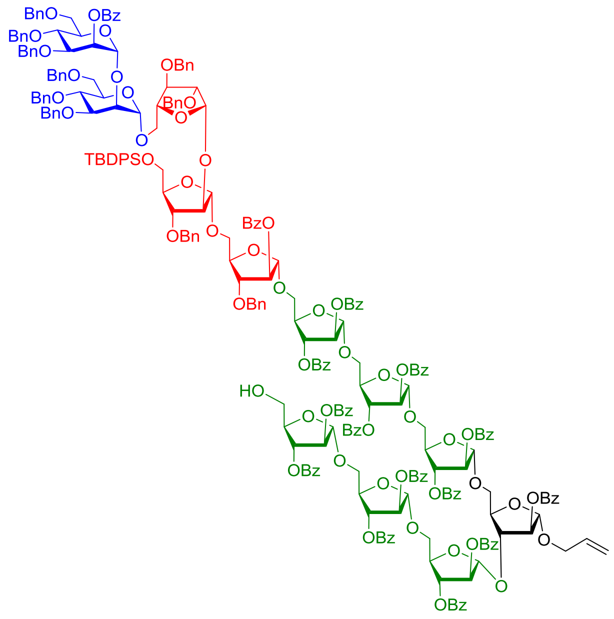

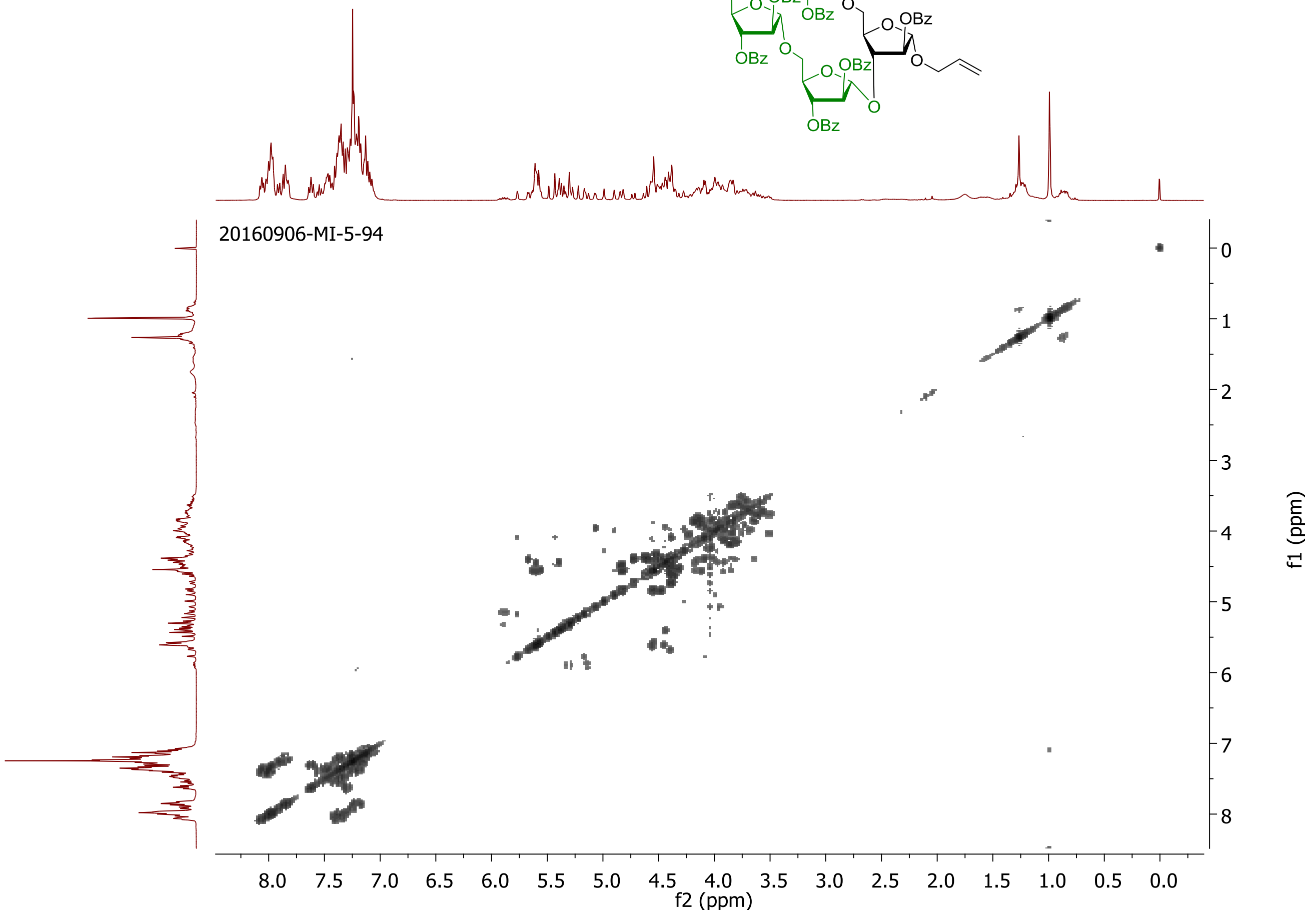

^{1}H-^{13}C HSQC Spectrum (600/150.99 MHz, $CDCl_3$) of Compound **45**

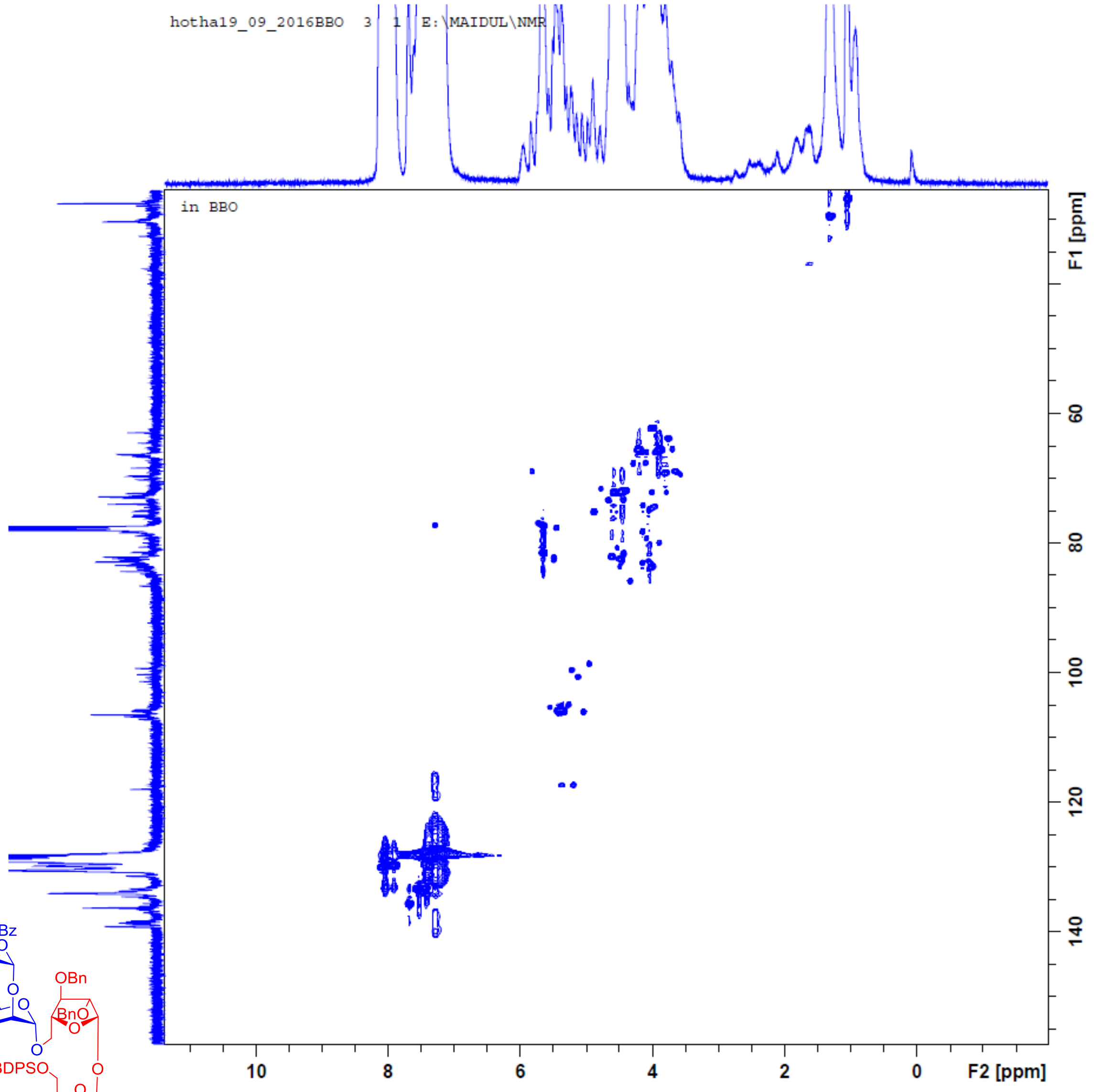

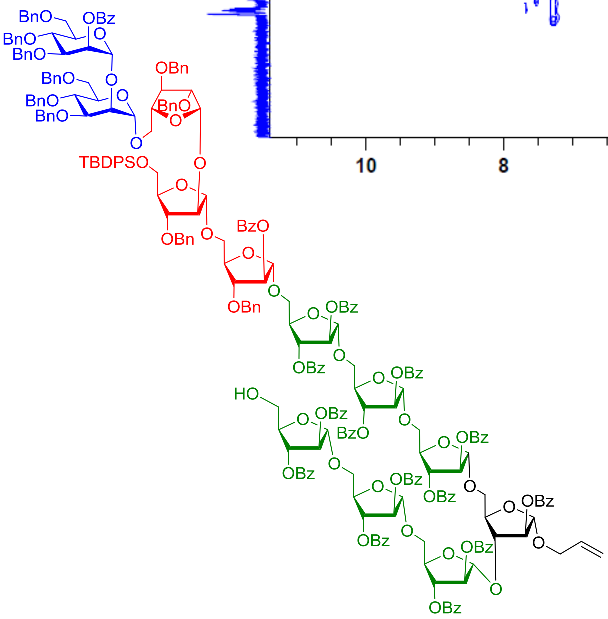

gHSQC Spectrum (600/150.99 MHz, $CDCl_3$) of Compound **45**

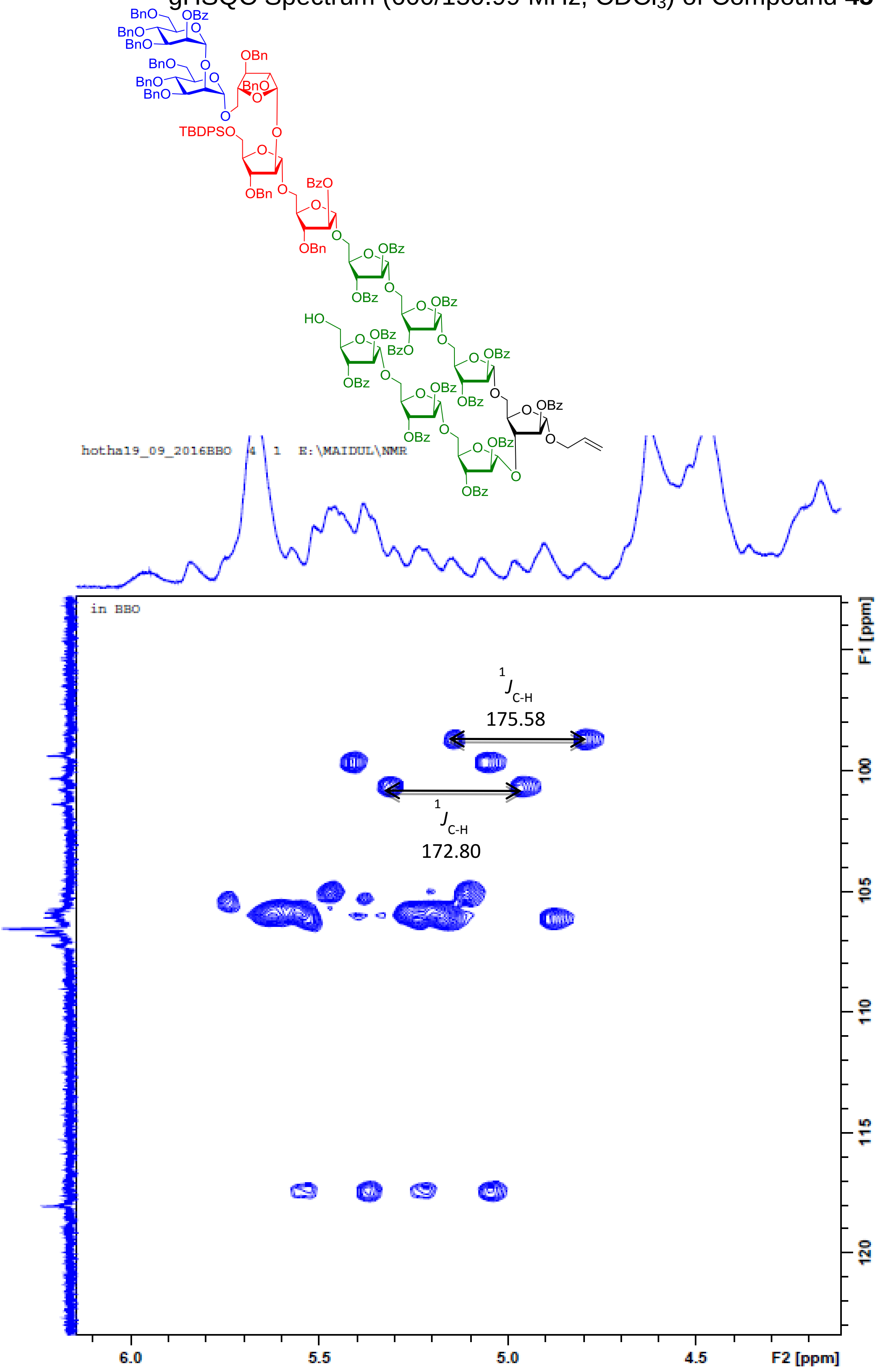

^{1}H NMR Spectrum (399.78 MHz, $CDCl_3$) of Compound **46**

2016-05-02-hota.1.fid
MI-5-98 in CDCl3

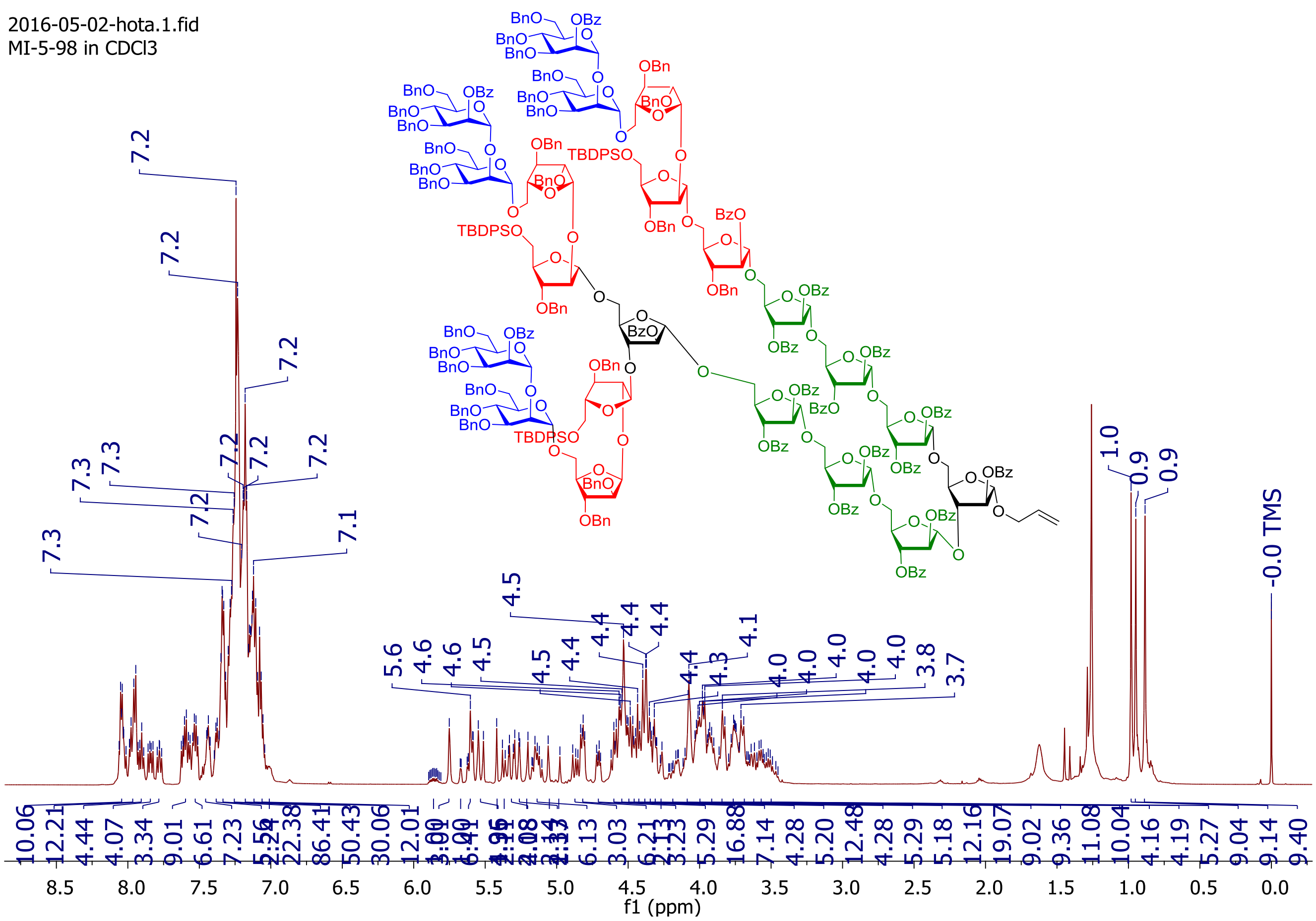

^{13}C NMR Spectrum (100.53 MHz, $CDCl_3$) of Compound **46**

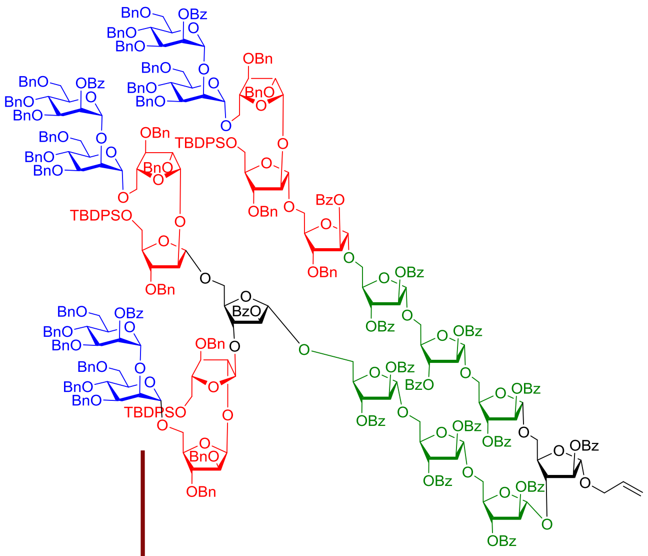

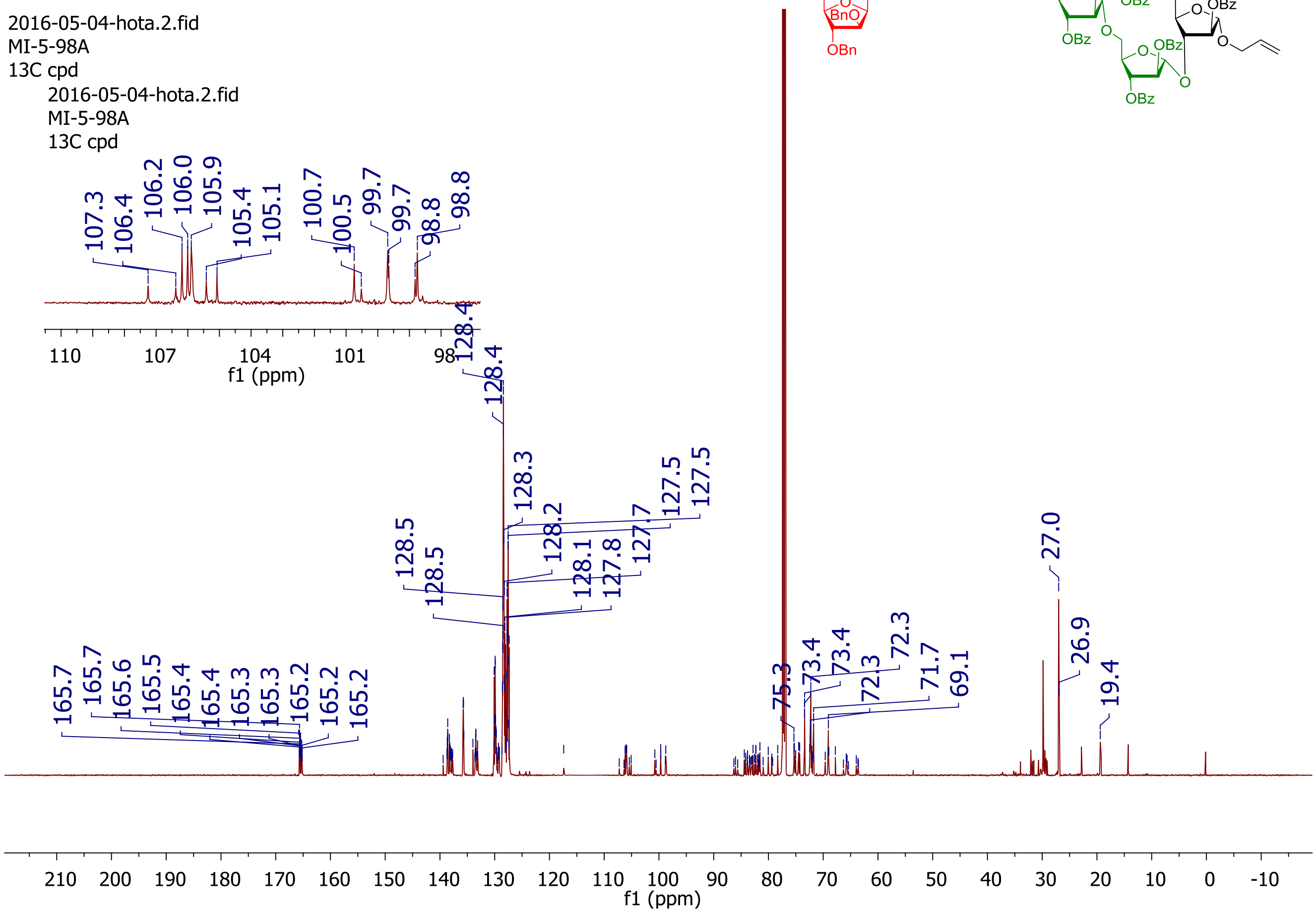

DEPT Spectrum (100.53 MHz, $CDCl_3$) of Compound **46**

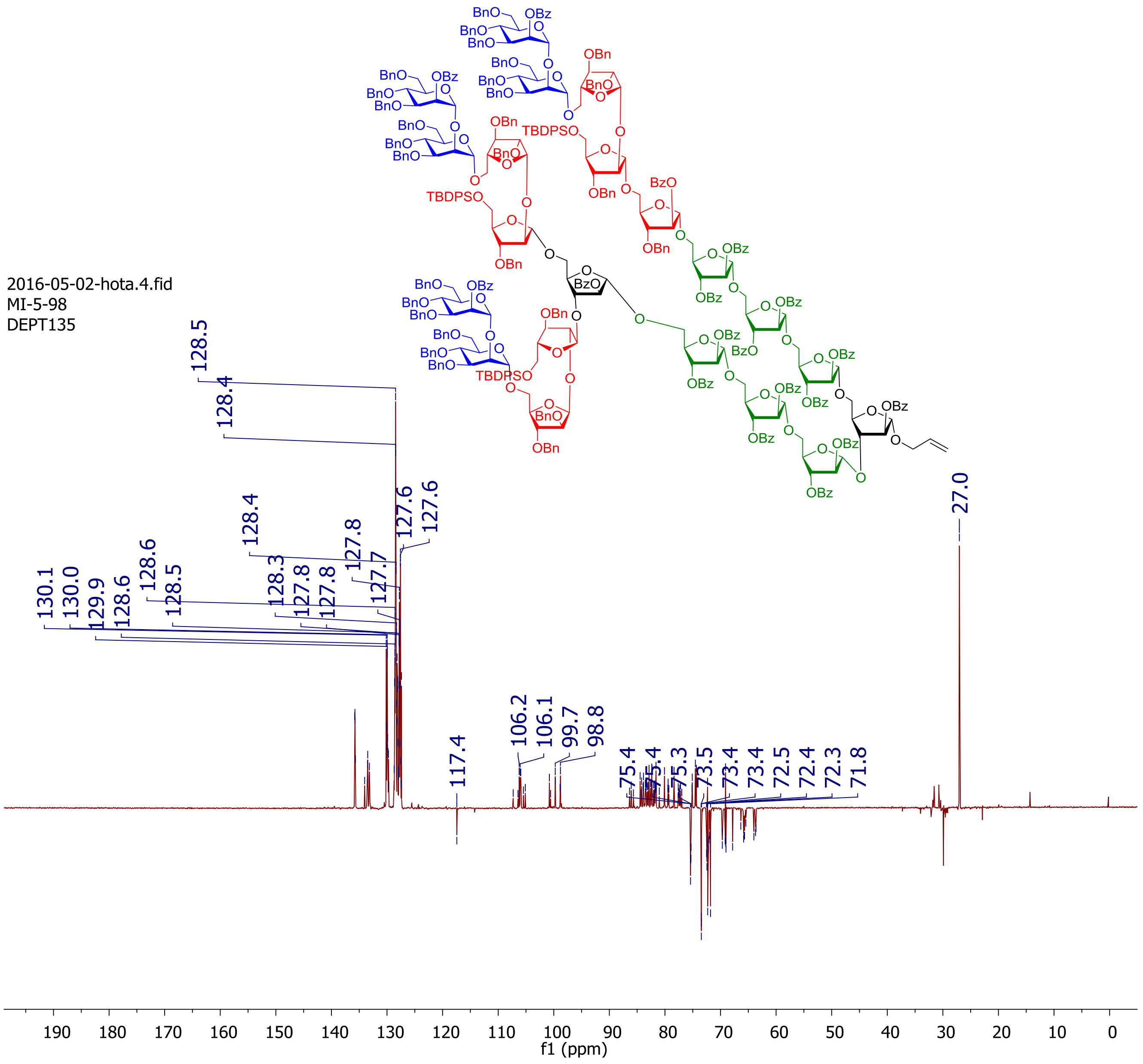

^{1}H-^{1}H COSY Spectrum (600MHz, $CDCl_3$) of Compound **46**

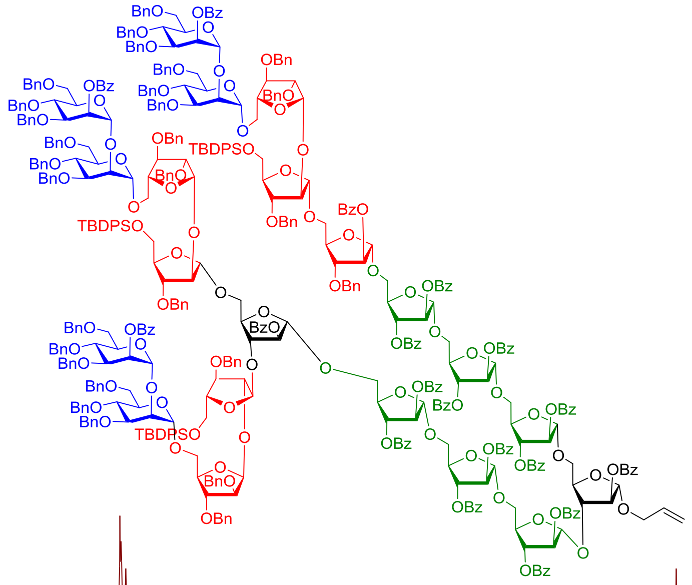

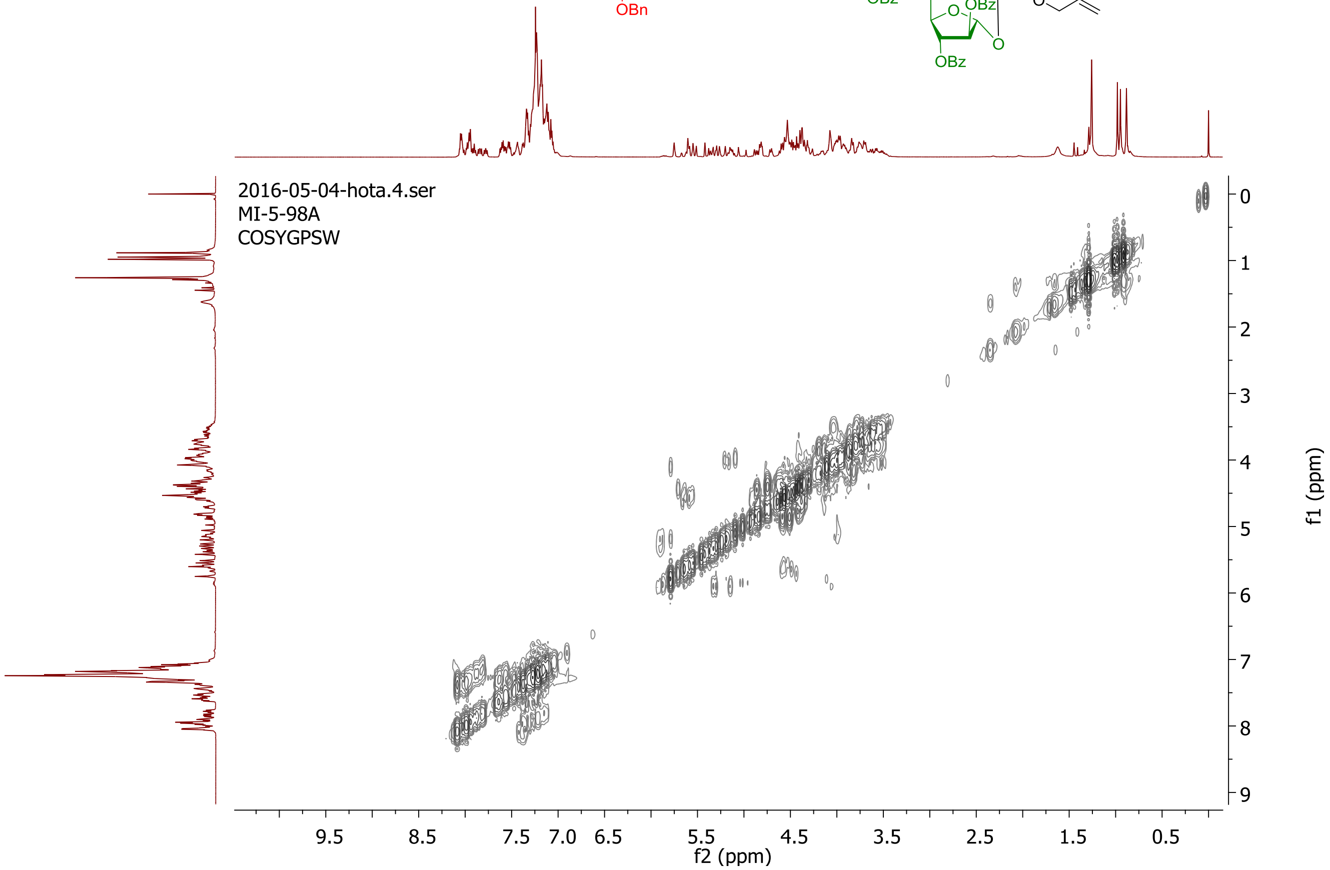

^{1}H-^{13}C HSQC Spectrum (600/150.99 MHz, $CDCl_3$) of Compound **46**

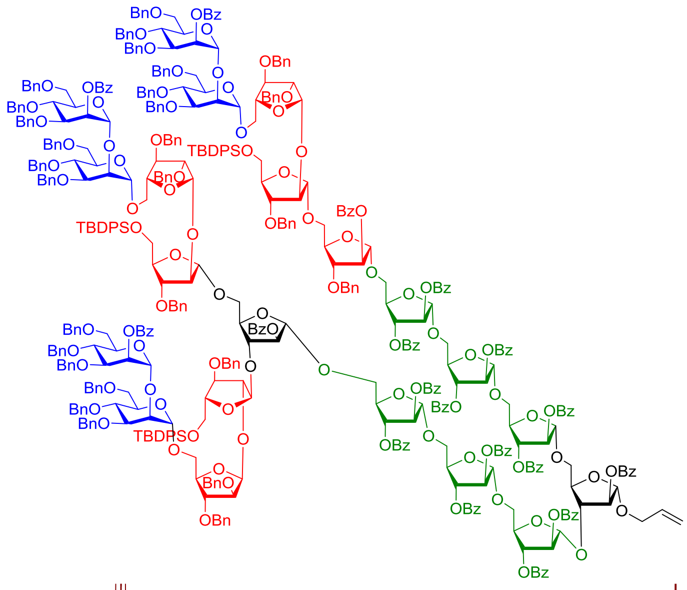

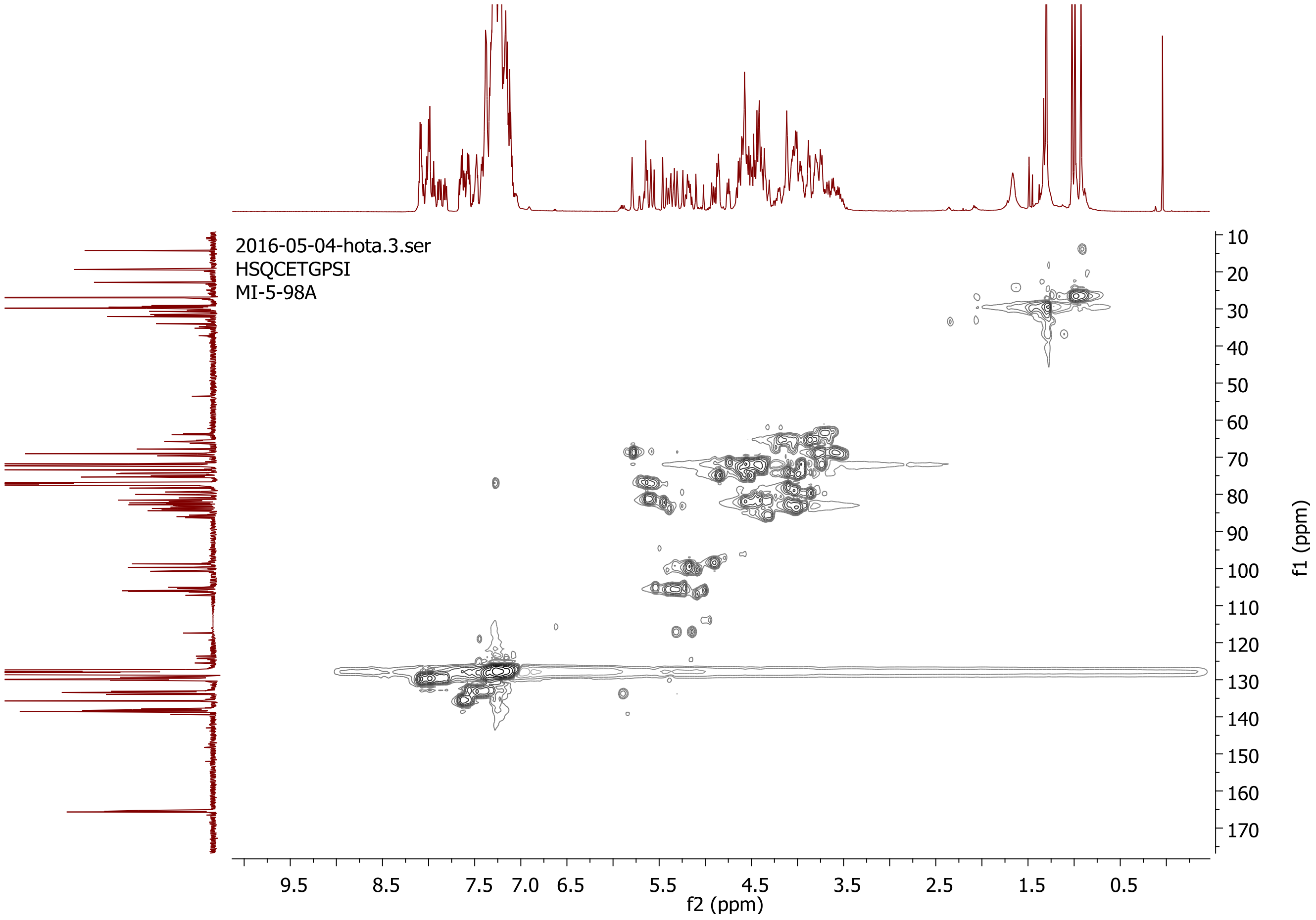

^{1}H NMR Spectrum (600.40 MHz, D_2O) of Compound **47**

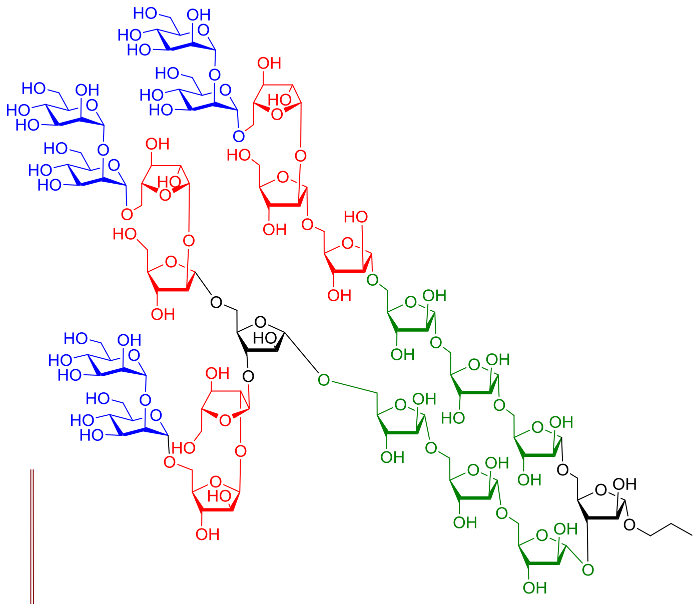

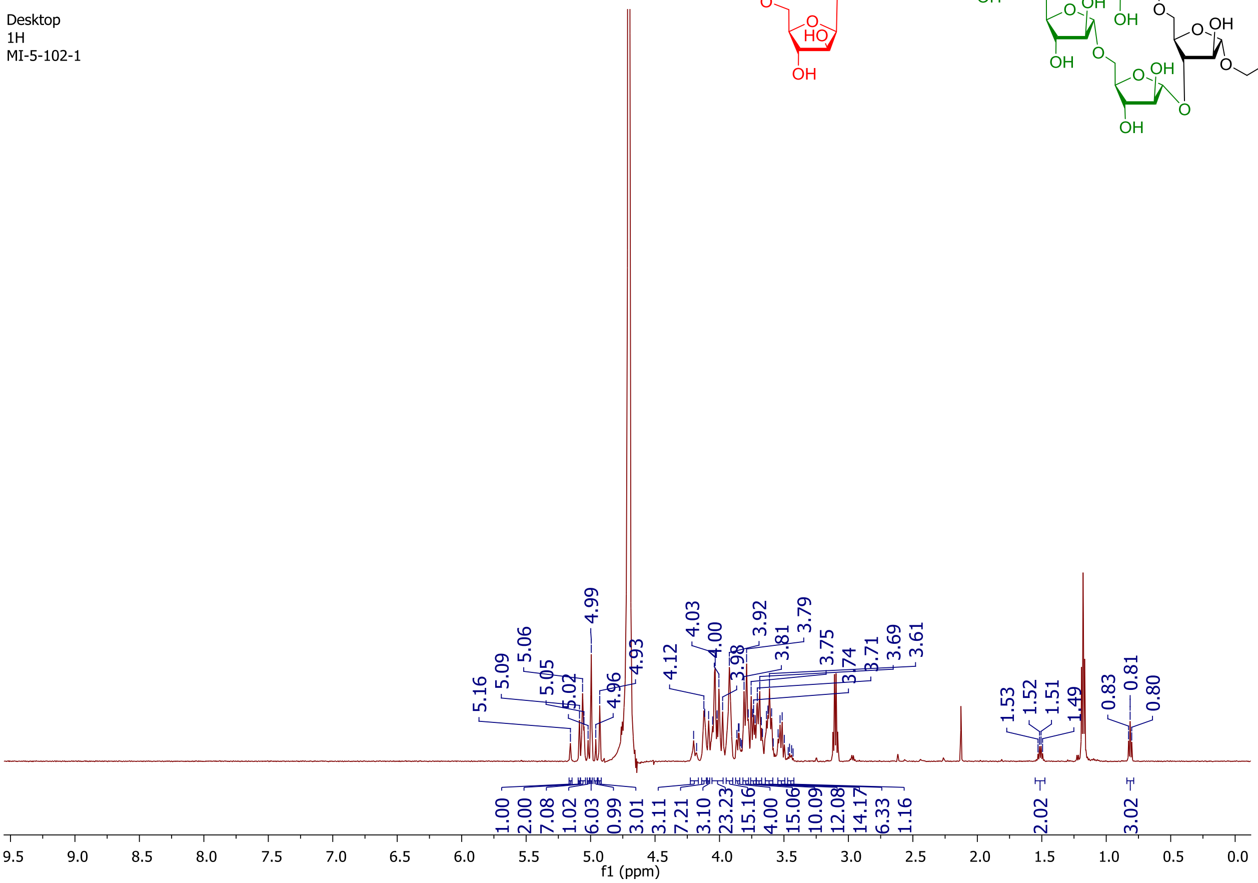

^{13}C NMR Spectrum (150.99 MHz, D_2O) of Compound **47**

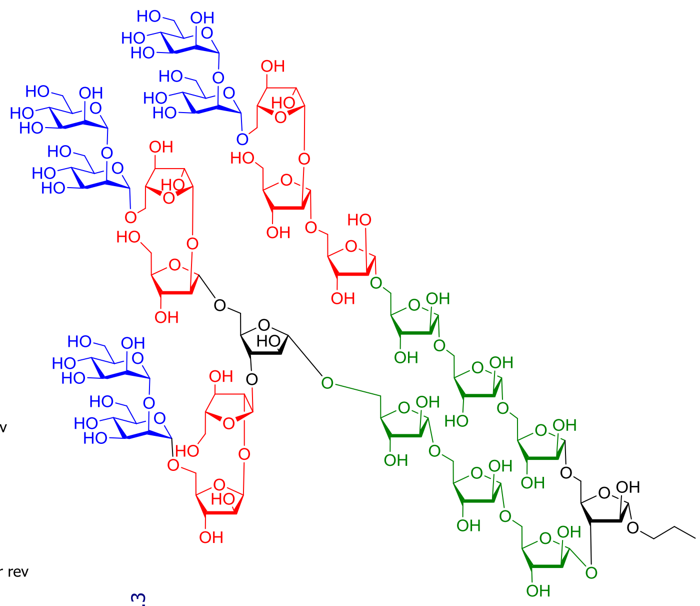

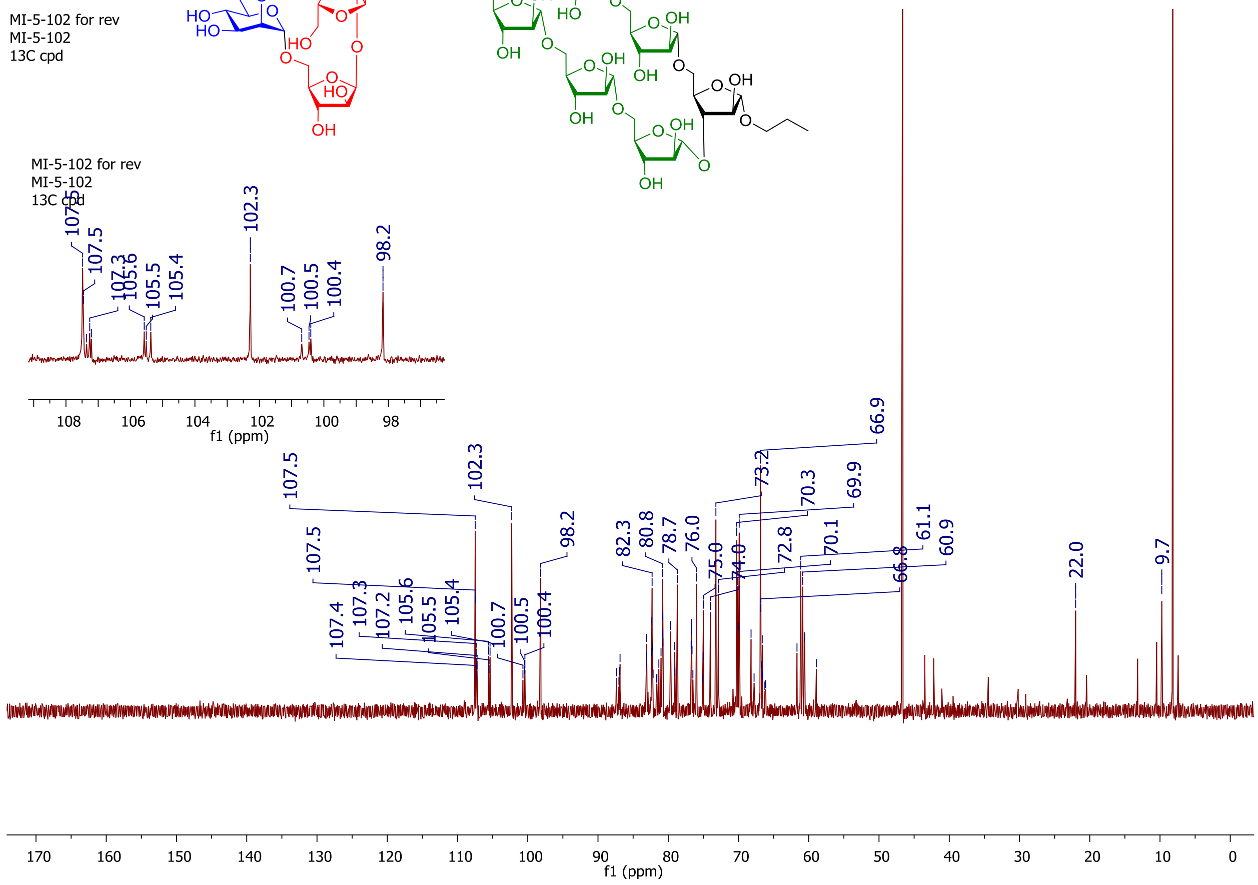

DEPT Spectrum (150.99 MHz, D_2O) of Compound **47**

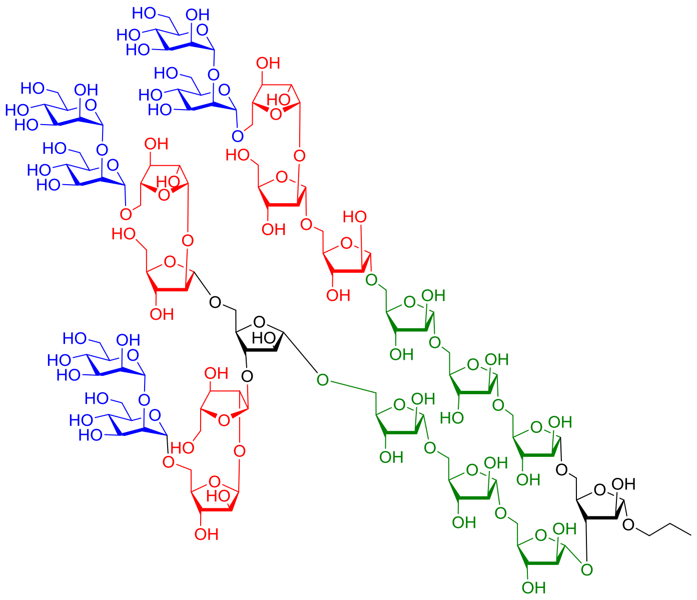

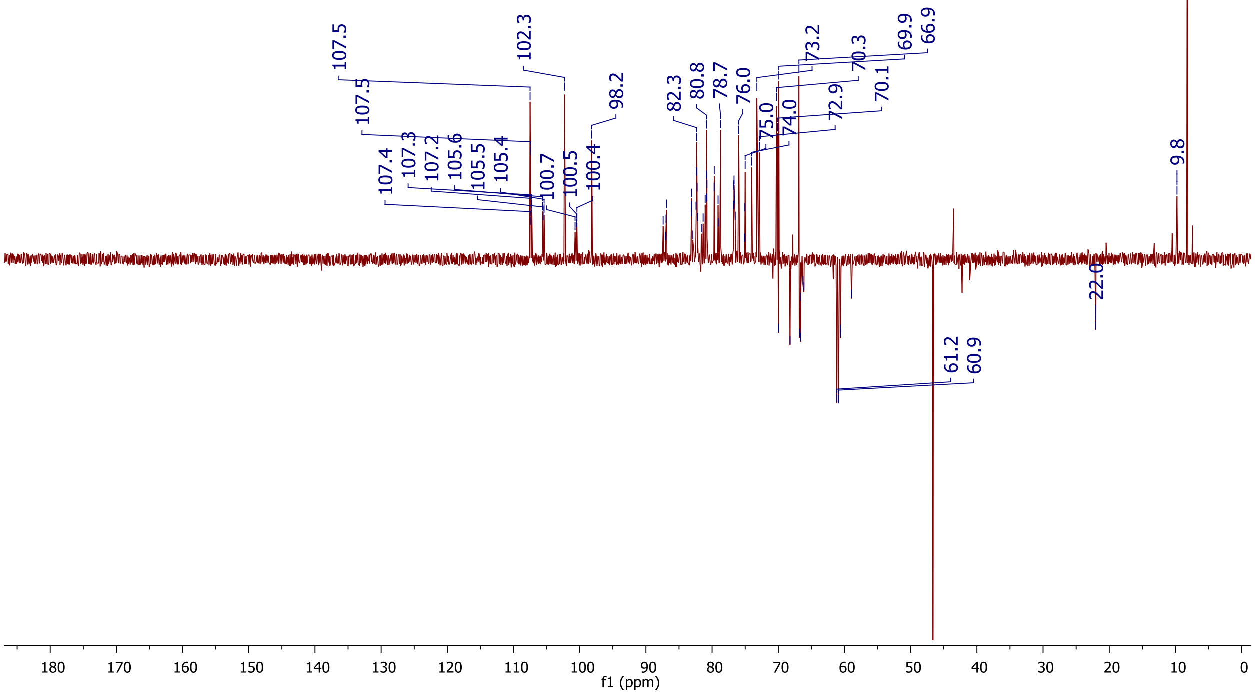

Supplement: Supplementary file 1 [file SC-008-C6SC04866H-s001.pdf]
